# The association between triglyceride-glucose index and its combination with obesity indicators and cardiovascular disease: NHANES 2003–2018

**DOI:** 10.1186/s12933-023-02115-9

**Published:** 2024-01-06

**Authors:** Keke Dang, Xuanyang Wang, Jinxia Hu, Yuntao Zhang, Licheng Cheng, Xiang Qi, Lin Liu, Zhu Ming, Xinmiao Tao, Ying Li

**Affiliations:** 1 Department of Nutrition and Food Hygiene, School of Public Health, Key Laboratory of Precision Nutrition and Health, Ministry of Education, Harbin Medical University, 157 Baojian Road, Heilongjiang, 150081 People’s Republic of China; 2 MED-X Institute, Center for Immunological and Metabolic Diseases (CIMD), First Affiliated Hospital of Xi’an Jiaotong University, Xi’an, 710000 China

**Keywords:** Triglyceride glucose (TyG), Triglyceride glucose-waist circumference (TyG-WC), Triglyceride glucose-waist height ratio (TyG-WHtR), Triglyceride glucose-body mass index (TyG-BMI), Cardiovascular disease (CVD) mortality, Cardiovascular disease, National Health and Nutrition Examination Survey (NHANES)

## Abstract

**Background:**

In the American population, the relationship between the triglyceride-glucose (TyG) index and TYG combined with indicators of obesity and cardiovascular disease (CVD) and its mortality has been less well studied.

**Methods:**

This cross-sectional study included 11,937 adults from the National Health and Nutrition Examination Survey (NHANES) 2003–2018. Cox proportional hazards model, binary logistic regression analyses, restricted cubic spline (RCS), and receiver operating characteristic (ROC) were used to analyze the relationship between TyG and its combined obesity-related indicators and CVD and its mortality. Mediation analysis explored the mediating role of glycated hemoglobin and insulin in the above relationships.

**Results:**

In this study, except for no significant association between TyG and CVD mortality, TyG, TyG-WC, TyG-WHtR, and TyG-BMI were significantly and positively associated with CVD and CVD mortality. TyG-WHtR is the strongest predictor of CVD mortality (HR 1.66, 95% CI 1.21–2.29). The TyG index correlated better with the risk of coronary heart disease (OR 2.52, 95% CI 1.66–3.83). TyG-WC correlated best with total CVD (OR 2.37, 95% CI 1.77–3.17), congestive heart failure (OR 2.14, 95% CI 1.31–3.51), and angina pectoris (OR 2.38, 95% CI 1.43–3.97). TyG-WHtR correlated best with myocardial infarction (OR 2.24, 95% CI 1.45–3.44). RCS analyses showed that most of the above relationships were linear (P-overall < 0.0001, P-nonlinear > 0.05). Otherwise, ROC curves showed that TyG-WHtR and TyG-WC had more robust diagnostic efficacy than TyG. In mediation analyses, glycated hemoglobin mediated in all the above relationships and insulin-mediated in partial relationships.

**Conclusions:**

TyG-WC and TyG-WtHR enhance CVD mortality prediction, diagnostic efficacy of CVD and its mortality, and correlation with some CVD over and above the current hottest TyG. TyG-WC and TyG-WtHR are expected to become more effective metrics for identifying populations at early risk of cardiovascular disease and improve risk stratification.

**Supplementary Information:**

The online version contains supplementary material available at 10.1186/s12933-023-02115-9.

## Introduction

Cardiovascular disease (CVD) is the leading cause of death and years of healthy life lost in humans, the leading cause of the global disease burden, and a significant contributor to lost health and high healthcare cost [1, 2]. Identifying factors that predict CVD risk is essential to promote early disease prevention.

The Triglyceride-Glucose (TyG) index is a measure of insulin resistance that assesses the body's insulin sensitivity by combining two biomarkers, triglyceride and fasting blood glucose [3, 4]. Insulin resistance is a state in which the body’s sensitivity and response to insulin are reduced, resulting in the inability of insulin to efficiently transport glucose into cells, causing metabolic abnormalities such as hyperglycemia [5]. Insulin resistance is considered a significant risk factor for several metabolic diseases such as type 2 diabetes [6], obesity [7], cardiovascular disease [8–10], and so on. The TyG index is calculated using the following formula: TyG = Ln (triacylglycerol (mg/dL) × fasting blood glucose (mg/dL)/2). Recent studies have shown that the TyG index not only predicts the risk and severity of cardiovascular disease but is also associated with the prognosis of cardiovascular disease [11–14]. However, studies on TyG and cardiovascular disease have focused on European and Asian populations, and there are relatively few studies on TyG and cardiovascular disease and CVD mortality in the American population.

Obesity is prevalent worldwide and is closely associated with various health risks, such as poor glucose tolerance, insulin resistance, and metabolic disorders [15–17], which can lead to the onset progression and prognosis of cardiovascular disease [18–20]. TYG combined with obesity indices is closely associated with insulin resistance [21, 22], metabolic syndrome [23], uric acid [24], diabetes mellitus [25], and fatty liver [26]. Some studies have shown that TyG combined with adiposity indices is better than the TyG index for assessing [27]. However, these studies on TyG combined with obesity indices and cardiovascular disease were few and mainly focused on Asian and European populations [28]. The relationship between TyG combined with obesity indices and cardiovascular disease and CVD mortality in the American population is unclear. Our study used the National Health and Nutrition Examination Survey database to analyse the association of the TyG index and its combination of obesity indices with CVD mortality, total CVD, congestive heart failure, myocardial infarction, angina pectoris, and coronary heart disease.

## Materials and methods

### Data source and study population

**Fig. 1:**
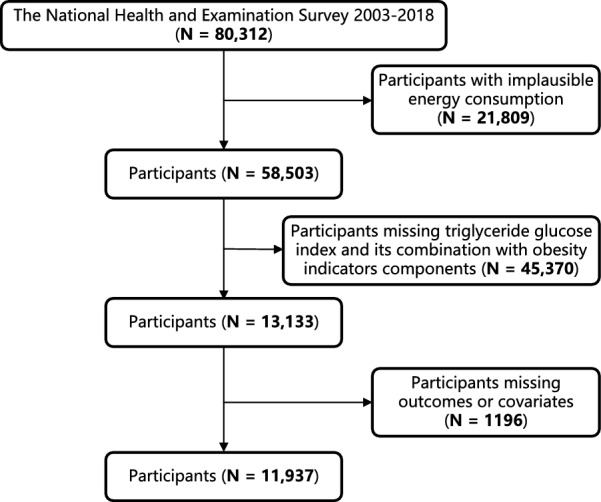
Flowchart depicting the participants’ selection

This cross-sectional study included 11,937 adult participants from the National Health and Nutrition Examination Survey (NHANES) 2003–2018. The study procedure is illustrated in Fig. 1. Exclusion criteria comprised the following: (1) dietary energy intake below 800 or over 4200 kcal/day for male and below 500 or more than 3500 kcal/day for female [29]; (2) participants with missing triglyceride-glucose index and its combinations with indicators of obesity; (3) participants lacking the outcome or covariates. The NCHS Research Ethics Review Board approved the NHANES study protocol, and all participants provided written informed consent.

### Definitions of TyG, TyG-WC, TyG-WHtR, and TyG-BMI

The TyG index quantifies insulin resistance by combining fasting glucose with triglyceride levels. Fasting blood glucose (FSG) and triglycerides were measured at baseline when the participants provided their blood samples. Body weight, height, and waist were obtained when people participated in the physical examinations at a mobile examination center. Furthermore, the body mass index along with the waist-to-height ratio were calculated. The participants were classified into four groups (Q1, Q2, Q3, Q4) by the quartiles of the TyG index, TyG-WC, TyG-WHtR, and TyG-BMI, respectively, and the Q1 group was used as the reference group.

TyG, TyG-WC, TyG-WHtR, and TyG-BMI were calculated according to the following formulas: (1) TyG = ln [triglycerides (mg/dl) × glucose (mg/dl)/2]; (2) BMI = body mass (kg)/height^2^(m^2^); (3) WHtR = waist circumference/height; (4) TyG-WC = TyG × waist circumference; TyG-WHtR = TyG × WHtR; TyG-BMI = TyG × BMI.

### Total CVD, congestive heart failure, myocardium infarction, angina pectoris, and coronary heart disease ascertainment

The diagnosis of CVD was established by self-reported physician diagnoses obtained during an individual interview using a standardized medical condition questionnaire. The participants were asked, “Has a doctor or other health expert ever informed you that you have congestive heart failure/coronary heart disease/angina pectoris/myocardial infarction /stroke?” A person was regarded as having CVD if he or she replied “yes” to any of the above questions. Congestive heart failure, myocardial infarction, angina pectoris, and coronary heart disease are also defined according to the problems of the corresponding diseases mentioned above.

According to the International Statistical Classification of Diseases and Related Health Problems, Tenth Revision (ICD-10) codes, cardiovascular disease (I00-I99), congestive heart failure (I50.0, I50.1, I50.9), coronary artery disease (I20-I25.9), myocardial infarction (I21-I23), and angina pectoris (I20.0-I20.9), CVD mortality (I00–I09, I11, I13, I20–I51, or I60–I69).

### Assessment of covariates

Age and year were considered as a continuous variable, gender was divided into two groups of males and females, race/ethnicity was divided into five sections of Mexican Hispanics, non-Mexican Hispanics, non-Hispanic whites, non-Hispanic blacks, and other, educational attainment was grouped according to the Questionnaire on Educational Attainment for Adults 20 Years of Age and Older (QEAA), household income was grouped according to annual household income greater or less than $100,000, and smoking was defined as never smoking (participants who smoked less than 100 cigarettes in their lifetime), smoking (participants who smoked more than 100 cigarettes in their lifetime), physical activity (yes/no), drinking was defined as never drinking (less than 12 drinks per year), drinking (at least 12 drinks per year) and use of dietary supplements (yes/no), total energy and AHEI were considered as continuous variables. Family history of heart disease, self-reported cancer, and self-reported diabetes was established by self-reported physician diagnoses obtained during an individual interview using a standardized medical condition questionnaire. The participants were asked: “Close relative had a heart attack?” or “Ever been told you had cancer or malignancy?” or “Doctor told you you had diabetes?” and answered by yes or no. Weight, height, blood pressure, and waist circumference were obtained when people attended a physical examination at a mobile health center and were considered continuous type variables. In addition, fasting blood glucose (FSG), insulin, HbA1c, triglycerides, and total cholesterol were measured at baseline when participants provided blood samples and were considered continuous type variables. The AHEI was developed from the original Healthy Eating Index, which included 11 food components identified through a comprehensive review of studies. More details on the measurement of covariates can be found on the NHANES website (https://www.cdc.gov/nchs/nhanes/index.htm).

### Statistical analysis

All statistical analyses were carried out by the CDC guidelines (https://wwwn.cdc.gov/nchs/nhanes/tutorials/default.aspx). The statistical analyses for this study incorporated sample weights, clustering, and stratification due to the complex multi-stage stratified probability survey design employed in NHANES. Mean values (95% CI) were used to express participant characteristics for continuous variables, while percentages (95% CI) were used for categorical variables. Participants' baseline characteristics were described according to the quartiles of the TyG index, TyG-WC, TyG-WHtR, and TyG-BMI, respectively. They were subjected to homogeneity of variance tests and further Bonferroni tests. The Cox proportional hazards model was used to estimate hazard ratios (HRs) and 95% CIs for the association between TyG index and TyG combined with obesity metrics and CVD mortality. Binary weighted logistic regression analyses assessed the association between the TyG index and TyG combined with obesity metrics and CVD. Correlation results were expressed as odds ratios (OR) and 95% confidence intervals (CI) in the four predefined models. In addition, linear trends between TyG, TyG-WC, TyG-WHtR, and TyG-BMI quartiles were assessed by the median value within each quartile as a continuous variable. Model 1 was adjusted for age, sex, race, and year. Model 2 adjusted for age, sex, race, year, smoking, alcohol use, exercise, education level, income, and family history of cardiovascular disease. Model 3 adjusted for the same variables as Model 2 and for energy intake, alternative healthy eating index, and nutritional supplements. Model 4 adjusted for the same variables as Model 3: SBP, cholesterol, self-reported cancer, and self-reported diabetes.

To account for the dose–response relationship (linear or nonlinear) between TyG index, TyG-WC, TyG-WHtR, and TyG-BMI and the cardiovascular disease mortality and cardiovascular disease, restricted triple spline analyses adjusted for the same variables as in model 4 were performed at the 5, 50, and 95th percentiles of the distributions of TyG, TyG-WC, TyG-WHtR, and TyG-BMI Three nodes were set to exclude the most extreme 5% values to reduce the potential impact of outliers. Non-linearity tests were performed using the likelihood ratio test. Receiver operating characteristic (ROC) curves were used for diagnostic value analysis, and the area under the curve, as measured by the C-statistic, was computed to quantify the predictive power of TyG, obesity-related indices (WC et al.), and their combination for cardiovascular disease and its mortality.

In addition, mediation analyses were used to investigate whether the relevance of TyG and its obesity composite index to cardiovascular disease could be explained by glycated hemoglobin and insulin after adjusting for factors in the primary analysis model 4. Stratified analyses were conducted to assess potential moderating effects of age (> 50/ ≤ 50), sex (male/female), race (non-Hispanic white/other), smoking (yes/no), physical activity (yes/no), alcohol consumption (yes/no), and diabetes (yes/no). The above assessments were conducted using r4.1.1 software, and statistical significance was determined using a two-sided p-value threshold of less than 0.05.

## Results

### Basic characteristics of participants according to the quartile of TyG, TyG-WC, TyG-WHtR, and TyG-BMI indicators

The characteristics of the participants according to quartiles of TyG, TyG-WC, TyG-WHtR, and TyG-BMI are shown in Table 1 and Additional file 3: Tables S1, S2, and S3. Participants with higher TyG index, TyG-WC, TyG-WHtR, and TyG-BMI were more likely to be male, older, to have a higher physical activity level, and to be higher levels of BMI, waist circumference, triglycerides, fasting glucose, glycated hemoglobin, systolic blood pressure, insulin, cholesterol, self-reported cancer, self-reported diabetes, cardiovascular diseases mortality, cardiovascular disease, congestive heart failure, myocardial infarction, angina pectoris, and coronary heart disease, and lower levels of educational strata, income, and AHEI.

**Table 1:** Baseline characteristics according to triglyceride-glucose (TyG) quartiles: NHANES, 2003–2018^a^

	Triglyceride-glucose (TyG) (N = 11,937)				*P*	*P*_*test*_
	≤ 8.22	8.23–8.62	8.63–9.06	> 9.07		
	N = 2986	N = 2986	N = 2983	N = 2982		
Age, years	42.33 (41.36, 43.29)	47.00 (46.13, 47.86)	50.08 (49.32, 50.85)	51.91 (51.15, 52.66)	< 0.001	< 0.001
Male, %	37.60 (35.70, 39.60)	47.40 (45.20, 49.70)	51.20 (49.20, 53.20)	58.50 (56.30, 60.70)	< 0.001	< 0.001
Non-Hispanic white, %	68.00 (64.80, 71.10)	71.00 (67.80, 73.90)	72.80 (69.70, 75.70)	73.10 (69.60, 76.30)	< 0.001	< 0.001
BMI, kg/m^2^	25.95 (25.71, 26.20)	27.95 (27.68, 28.22)	29.54 (29.23, 29.86)	31.46 (31.10, 31.81)	< 0.001	< 0.001
Smoke, %	19.80 (17.80, 22.00)	24.20 (21.80, 26.70)	25.90 (23.80, 28.20)	27.20 (25.30, 29.30)	< 0.001	< 0.001
Drink, %	74.00 (71.30, 76.50)	73.90 (71.60, 76.10)	74.60 (72.10, 76.90)	73.20 (70.90, 75.40)	< 0.001	< 0.001
Regular exercise, %	32.90 (30.30, 35.70)	42.20 (39.90, 44.60)	43.60 (41.20, 46.00)	50.90 (48.10, 53.70)	< 0.001	< 0.001
College graduate or above, %	36.90 (34.10, 39.80)	30.80 (27.60, 34.10)	27.10 (24.30, 30.10)	21.20 (18.80, 23.80)	< 0.001	< 0.001
> 100,000 annual household income, %	19.60 (16.80, 22.60)	16.50 (13.80, 19.60)	13.80 (11.90, 15.90)	10.40 (8.10, 13.30)	0.078	0.336
Dietary supplements use, %	51.00 (48.90, 53.10)	51.30 (48.90, 53.60)	50.20 (48.00, 52.50)	52.70 (50.10, 55.20)	< 0.001	< 0.001
Total energy, kcal/day	2045 (2015, 2027)	2090 (2057, 2124)	2060 (2018, 2102)	2111 (2075, 2148)	< 0.001	< 0.001
AHEI sore	50.86 (50.31, 51.41)	50.03 (49.47, 50.59)	50.21 (49.72, 50.71)	49.58 (49.06, 50.09)	< 0.001	< 0.001
Family history of heart disease, %	13.00 (11.60, 14.70)	16.70 (14.70, 18.90)	17.70 (15.90, 19.60)	18.80 (16.70, 21.10)	0.078	0.033
Triglyceride, mmol/L	0.68 (0.67, 0.69)	1.07 (1.06, 1.07)	1.53 (1.52, 1.54)	2.91 (2.82, 3.00)	< 0.001	< 0.001
Fasting glucose, mmol/L	5.15 (5.12, 5.18)	5.46 (5.43, 5.49)	5.76 (5.71, 5.80)	6.93 (6.80, 7.05)	< 0.001	< 0.001
Waist circumference, cm	89.93 (89.33, 90.52)	96.67 (95.87, 97.47)	101.12 (100.46, 101.78)	107.34 (106.49, 108.19)	< 0.001	< 0.001
Glycohemoglobin, %	5.35 (5.34, 5.37)	5.52 (5.50, 5.54)	5.65 (5.63, 5.68)	6.34 (6.28, 6.40)	< 0.001	< 0.001
Standing height, cm	167.49 (167.14, 167.83)	167.86 (167.50, 168.22)	167.41 (167.04, 167.79)	168.01 (167.64, 168.38)	< 0.001	< 0.001
Insulin, pmol/L	49.54 (48.07, 51.01)	65.07 (63.15, 67.00)	81.76 (79.41, 84.11)	118.02 (112.65, 123.39)	< 0.001	< 0.001
Systolic blood pressure, mmHg	115.63 (114.88, 116.38)	120.18 (119.44, 120.91)	122.24 (121.52, 122.96)	127.11 (126.20, 128.02)	< 0.001	< 0.001
Cholesterol, mmol/L	4.67 (4.63, 4.72)	5.00 (4.95, 5.05)	5.21 (5.17, 5.26)	5.50 (5.44, 5.55)	< 0.001	< 0.001
Self-reported cancer, %	7.10 (5.90, 8.60)	9.50 (8.00, 11.20)	10.70 (9.40, 12.30)	11.20 (10.00, 12.60)	< 0.001	< 0.001
Self-reported diabetes, %	2.20 (1.60, 2.80)	4.20 (3.50, 5.10)	7.70 (6.70, 8.90)	21.40 (19.10, 23.80)	< 0.001	< 0.001
Death of cardiovascular diseases, %	2.00 (1.50, 2.60)	3.40 (2.90, 4.10)	4.20 (3.50, 4.90)	6.60 (5.70, 7.80)	< 0.001	< 0.001
Cardiovascular diseases, %	4.80 (4.10, 5.60)	7.30 (6.20, 8.60)	10.00 (8.70, 11.50)	13.70 (12.10, 15.50)	< 0.001	< 0.001
Congestive heart failure, %	1.20 (0.90, 1.70)	1.80 (1.30, 2.40)	2.60 (2.00, 3.40)	4.40 (3.60, 5.30)	< 0.001	< 0.001
Myocardial infarction, %	1.70 (1.30, 2.40)	3.20 (2.40, 4.20)	4.00 (3.40, 4.80)	5.50 (4.50, 6.60)	< 0.001	< 0.001
Angina pectoris, %	1.10 (0.80, 1.60)	1.60 (1.10, 2.20)	3.10 (2.40, 0.40)	3.80 (0.30, 4.80)	< 0.001	< 0.001
Coronary heart disease, %	1.70 (1.30, 2.40)	2.80 (2.10, 3.60)	4.40 (3.50, 5.50)	6.30 (5.30, 7.50)	< 0.001	< 0.001

^a^Continuous variables were listed as weighted mean (95% CI). Categorical variables were listed as weighted percentage (95% CI). After adjusting for age, general linear models and chi-square tests were conducted to compare continuous and categorical baseline characteristics, respectively. And *P*_test_ was the result of Bonfreni correction

### Relationship between TyG, TyG-WC, TyG-WHtR, TyG-BMI, and cardiovascular disease mortality, total CVD, congestive heart failure, myocardial infarction, angina pectoris, and coronary heart disease

**Fig. 2:**
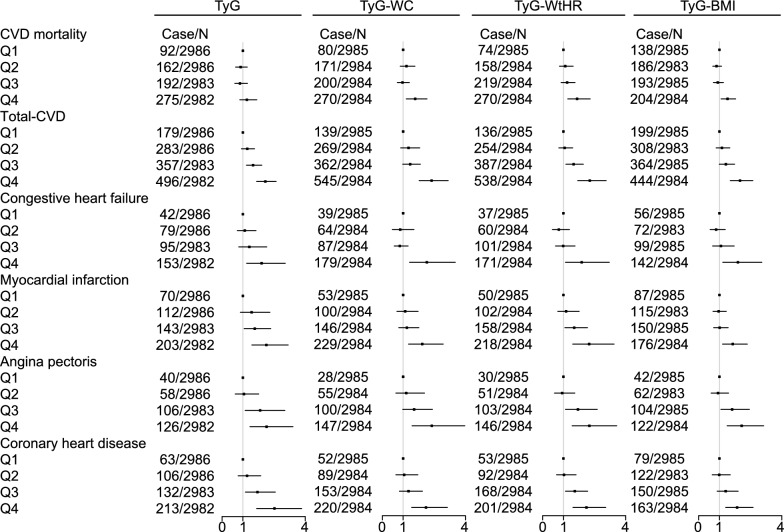
Forest plot of the TyG, TyG-WC, TyG-WHtR, and TyG-BMI association with CVD mortality, total CVD, congestive heart failure, myocardial infarction, angina pectoris, and coronary heart disease calculated using binomial logistic regression models/Cox proportional hazards model. The adjustments involved the covariables selected in the full binomial logistic regression model/Cox proportional hazards model. Case/N, the number of case subjects/total. *Q* quartile

Figure 2 demonstrates the association of TyG and its combined obesity indicators with cardiovascular disease. Additional file 3: Tables S4–S9 provide detailed information on all associations. After adjustment for covariates, the results showed that except for no significant association between TyG and CVD mortality, TyG, TyG-WC, TyG-WHtR, and TyG-BMI were significantly and positively associated with CVD mortality, total cardiovascular disease, congestive heart failure, myocar-dial infarction, angina pectoris, and coronary heart disease (P trend < 0.05, P_test_ < 0.05).

For CVD mortality, TyG-WHtR had the highest predictive power (HR 1.66, 95% CI 1.21–2.29), followed by TyG-WC (HR 1.58, 95% CI 1.15–2.15). For total CVD, TyG-WC had the highest association (OR 2.37, 95% CI 1.77–3.17), followed by TyG-WHTR (OR 2.27, 95% CI 1.69–3.06). For congestive heart failure, TyG-WC had the highest association (OR 2.14, 95% CI 1.31–3.51), followed by TyG (OR 1.90, 95% CI 1.18–3.04). For myocardial infarction, TyG-WHTR had the highest association (OR 2.24, 95% CI 1.45–3.44), followed by TyG (OR 2.13, 95% CI 1.43–3.18). For angina pectoris, TyG-WC had the highest association (OR 2.38, 95% CI 1.43–3.97), followed by TyG-WHTR (OR 2.25, 95% CI 1.42–3.57). For coronary heart disease, TyG had the highest association (OR 2.52, 95% CI 1.66–3.83), followed by TyG-WHTR (OR 2.11, 95% CI 1.46–3.04).

### Restricted cubic splines (RCS) analysis investigating the relationship between TyG, TyG-WC, TyG-WHtR, and TyG-BMI and cardiovascular disease mortality, total CVD, congestive heart failure, myocardial infarction, angina pectoris, and coronary heart disease

**Fig. 3:**
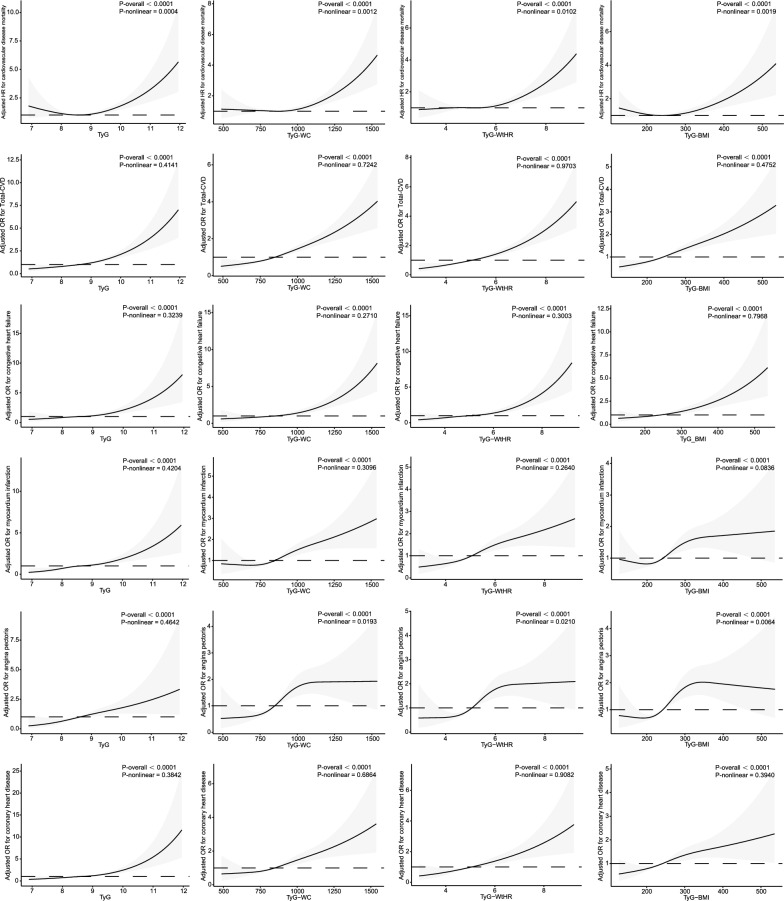
Associations between TyG, TyG-WC, TyG-WHtR, and TyG-BMI with cardiovascular mortality, total CVD, congestive heart failure, myocardial infarction, angina pectoris, and coronary heart disease were evaluated by RCS after adjustment for the covariables. The solid black lines correspond to the central estimates, and the gray-shaded regions indicate the 95% confidence intervals

In Fig. 3, We employed restricted cubic spline to flexibly model and visualize the associations between TyG, TyG-WC, TyG-WHtR, and TyG-BMI, and cardiovascular mortality, total cardiovascular disease, congestive heart failure, myocardial infarction, angina pectoris, and coronary heart disease. After adjusting for all covariates in the master analytical model 4 above, a linear correlation was observed between TyG and total CVD, congestive heart failure, myocardium infarction, angina pectoris, and coronary heart disease (P-overall < 0.0001, P-nonlinear > 0.05). Similarly, TyG-WC, TyG-WHtR, and TyG-BMI exhibited linear correlations with total CVD, myocardial infarction, and coronary heart disease (P-overall < 0.0001, P-nonlinear > 0.05). TyG, TyG-WC, TyG-WHtR, and TyG-BMI with CVD mortality exhibited nonlinear associations (P-overall < 0.0001 and P-nonlinear < 0.05), as well as TyG-WC, TyG-WHtR, and TyG-BMI with angina pectoris (P-overall < 0.0001 and P-nonlinear < 0.05).

### Mediation analysis of TyG, TyG-WC, TyG-WHtR, and TyG-BMI with CVD mortality, total CVD, congestive heart failure, myocardial infarction, angina pectoris, and coronary heart disease

Mediation analyses indicated that glycated hemoglobin partially mediated the association between TyG and its combined obesity indicators and CVD and CVD mortality (Additional file 1: Fig. S1). Glycated hemoglobin mediated a more significant proportion of indirect effects in the following relationships. For TyG, the proportion of indirect effects of glycated hemoglobin-mediated CVD mortality, total CVD, and congestive heart failure were 49.0, 41.6, and 42.2%. For the TyG-WHtR, the proportion of indirect effects of glycated hemoglobin-mediated coronary heart disease was 43.9%. For TyG-BMI, the ratios of indirect effects of glycated hemoglobin-mediated total CVD, myocardial infarction, and coronary heart disease were 42.3, 55.0, and 67.8%, respectively.

The proportions of indirect effects of insulin-mediated associations between TyG, TyG-WC, TyG-WHtR, and CVD mortality were − 11.2, − 25.1, and − 23.8%, respectively. For TyG, the proportions of indirect effects of insulin-mediated total CVD, congestive heart failure, and angina pectoris were 6.4, 10.2, and 10.6%, respectively. For TyG-BMI, the indirect effects of insulin-mediated total CVD and coronary heart disease were 11.4% and 29.5 (Additional file 1: Fig S1).

### Receiver operating characteristic (ROC) curves of TyG, TyG-WC, TyG-WHtR, TyG-BMI, WC, WHtR, and BMI in relation to cardiovascular disease mortality, total CVD, congestive heart failure, myocardial infarction, angina pectoris, and coronary heart disease

The ROC curve showed that TyG-WHtR or TyG-WC had the highest diagnostic efficacy for CVD and its mortality, followed by WHtR or WC, TyG, TyG-BMI, and BMI (Additional file 2: Fig S2).

For CVD mortality, TyG-WHtR had the highest diagnostic efficacy (AUC: 0. 628, 95% CI 0.608–0.647), followed by TyG-WC (AUC: 0.614, 95% CI 0.595–0.634). For total CVD, TyG-WHtR had the highest diagnostic efficacy (AUC: 0. 655, 95% CI 0.640–0.670), followed by TyG-WC (AUC: 0.650, 95% CI 0.635–0.665). For congestive heart failure, TyG-WHtR had the highest diagnostic efficacy (AUC: 0.675, 95% CI 0.648–0.702), followed by TyG-WC (AUC: 0.669, 95% CI 0.641–0.696). For angina pectoris, TyG-WHtR had the highest diagnostic efficacy (AUC: 0.649, 95% CI 0.627–0.671), followed by TyG-WC (AUC: 0.648, 95% CI 0.625–0.670). For myocardial infarction, TyG-WHtR had the highest diagnostic efficacy (AUC: 0.665, 95% CI 0.639–0.692), followed by TyG-WC (AUC: 0.663, 95% CI 0.636–0.690). For coronary heart disease, TyG-WC had the highest diagnostic efficacy (AUC: 0.654, 95% CI 0.632–0.677), followed by TyG-WHtR (AUC: 0.642, 95% CI 0.620–0.664).

### Stratification of TyG, TyG-WC, TyG-WHtR, and TyG-BMI in relation to CVDmortality, total CVD, congestive heart failure, myocardial infarction, angina pectoris, and coronary heart disease

After controlling for variables, segmented analyses according to age, sex, race, exercise, smoking, alcohol consumption and diabetes (Additional file 3: Tables S10–S31) identified that significant correlation between TyG, TyG-WC, TyG-WHtR, and TyG-BMI with total CVD, congestive heart failure, myocardial infarction, angina pectoris, and coronary heart disease were more regularly observed within individuals who were ≤ 50 years of age, male, did not smoke, and no diabetes. Significant associations between TyG, TyG-WC, TyG-WHtR, and TyG-BMI and CVD mortality were more likely to occur among individuals aged ≤ 50 years, women, drinkers, and no diabetes.

## Discussion

Our primary discoveries revealed that except for no significant association between TyG and CVD mortality, TyG, TyG-WC, TyG-WHtR, and TyG-BMI were significantly and positively associated with CVD mortality, total cardiovascular disease, congestive heart failure, myocardial infarction, angina pectoris, and coronary heart disease. Among the above indicators, TyG-WHtR had the best predictive ability for CVD mortality and the best correlation for myocardial infarction. TyG-WC had the highest correlation with total cardiovascular disease, congestive heart failure, and angina pectoris. TyG had the highest correlation with coronary heart disease. (2) TyG-WHtR and TyG-WC had more robust diagnostic efficacy than TyG for CVD and CVD mortality. (3) These associations were mainly mediated by hemoglobin. (4) Associations between TyG, TyG-WC, TyG-WHtR, and TyG-BMI and CVD were found to be more prevalent among individuals who were ≤ 50 years of age, male, did not smoke and no diabetes. Significant associations be-tween TyG, TyG-WC, TyG-WHtR, and TyG-BMI and CVD mortality were more likely to occur among individuals aged ≤ 50 years, women, drinkers, and no diabetes.

TyG, a biomarker derived from fasting glucose and triglyceride levels, has gained popularity as a substitute for insulin resistance owing to its user-friendly calculation and high degree of sensitivity and specificity [30–32]. Our research demonstrates a signifcant and positive connection between TyG and total CVD, congestive heart failure, myocardial infarction, angina pectoris, and coronary heart disease in the United States population, consistent with previous studies [33, 34]. However, current research on TyG and cardiovascular disease has centered on European and Asian populations, with a limited number of studies conducted in the United States [35]. Following the adjustment of nutritional data covariates such as total energy intake, AHEI, and dietary supplements, our investigation determined that TyG showed a significant and affirmative correlation with total CVD, congestive heart failure, myocardial infarction, angina pectoris, and coronary heart disease.

Our study is the inaugural investigation into the correlation between TyG combined with obesity indicators and CVD and CVD mortality in an American population. The findings of our study reveal that except for no significant association between TyG and CVD mortality, TyG, TyG-WC, TyG-WHtR, and TyG-BMI were significantly and positively associated with CVD mortality, total cardiovascular disease, congestive heart failure, myocar-dial infarction, angina pectoris, and coronary heart disease. RCS analyses showed that most of the above relationships were linear, but TyG-WC, TyG-WHtR, and TyG-BMI were non-linearly associated with cardiovascular mortality. This is consistent with previous reports [36]. In a prospective cohort study from the United States population, the TyG index was associated with mortality due to all-cause and cardiovascular disease non-linearly. In Model 3, the positive effect sizes become non-significant with cardiovascular death (HR, 1.37; 95% CI 0.78–2.42) [37]. It has been suggested that excessively high or low TyG, TyG-WC, and TyG-BMI indices may lead to an increased risk of cardiovascular death, which may be related to excessively high or low insulin resistance, inflammatory response, oxidative stress vascular endothelial function, which in turn may lead to an increased risk of cardiovascular death [38, 39]. TyG-WC, TyG-WHtR, and TyG-BMI were non-linearly associated with angina pectoris. The RCS curves do not represent the dispersion of the data. They may be related to the small number of angina patients, the disease's complexity, the angina data's multidimensionality, the setting of the parameters of the RCS curves, and the choice of their models.

Our research found that TyG-WHtR had the best predictive ability for CVD mortality and the best correlation for myocardial infarction. TyG-WC had the highest correlation with total cardiovascular disease, congestive heart failure, and angina pectoris. TyG had the highest correlation with coronary heart disease. The ROC curves showed that TyG-WHtR had the best diagnostic efficacy for cardiovascular disease mortality, total CVD, congestive heart failure, angina pectoris, and myocardial infarction. TyG-WC had the best diagnostic efficacy for coronary heart disease. The diagnostic efficacy of TyG-WC and TyG-WHtR for CVD and CVD mortality was higher than that of TyG, WC, and WHtR. TyG-BMI was more effective than BMI in diagnosing CVD and CVD mortality. The above results suggest that the correlation and diagnostic efficacy of TyG-WC and TyG-WHtR with cardiovascular diseases and their deaths are, to some extent, superior to the TyG index. A study involving 1145 participants from Korea [40], the results also showed that TyG-WC had better diagnostic efficacy for the progression of coronary artery calcification than TyG and TyG-BMI. Among the possible reasons, obesity can contribute directly to the development of cardiovascular risk factors, including dyslipidemia, type 2 diabetes, hypertension, and sleep disorders [41]. Obesity may also contribute to the development of cardiovascular disease and death from cardiovascular disease independently of other cardiovascular risk factors, especially the location of body fat distribution [41, 42]. Furthermore, integrating TyG with obesity indices presents a more accurate insulin resistance evaluation than the Homeostatic Model Assessment of Insulin Resistance (HOMA-IR) or TyG index alone [3, 21, 22, 27]. Our results provide significant proof for the literature on TyG, TyG-BMI, TyG-WC, and TyG-WHtR as predictors of cardiovascular disease risk.

Our research demonstrates that except for no significant association between TyG and CVD mortality, TyG, TyG-WC, TyG-WHtR, and TyG-BMI were significantly and positively associated with CVD and CVD mortality, especially in younger individuals. These findings are consistent with another study conducted among the American population, which revealed that the TyG index is significantly linked to a higher probability of heart failure in a younger age group (less than 60 years) [43]. Another study of an Iranian population showed that the TyG index was significantly associated with an increased risk of developing Cardiovascular disease/coronary heart disease and was more pronounced in younger people [44]. It is plausible that as one ages, there might be more risk factors for cardiovascular disease, making the predictive capabilities of TyG and its combination with obesity indices less potent in older populations. Our study also showed that the correlation of TyG, TyG-WC, TyG-WHtR, and TyG-BMI with CVD was higher in men than women. In contrast, the correlation of TyG-WC and TyG-WHtR with CVD mortality was higher in women than men. Current reports of sex differences in TyG, TyG-WC, TyG-WHtR, and TyG-BMI with CVD and CVD mortality are inconsistent. They may be related to the age and sex composition of the study population. A joint study of a prospective cohort and the Hong Kong Cohort Study showed that the association between the TyG index and the risk of heart failure events was stronger in women than in men [45]. In another study of the TyG index and the risk of a first major hard cardiovascular event within ten years, subgroup analyses showed that the above correlation was [β = 3.862 95% CI (3.274, 4.450), < 0.00001] in men and [β = 1.067, 95% CI (0.286, 1.849), = 0.00756] in women) [35]. Futhermore, our study suggests that associations between TyG, TyG-WC, TyG-WHtR, and TyG-BMI and CVD and CVD mortality was higher in no diabetes. Laura Sánchez-Íñigo et al. [46] and Liu Li et al. [47] were consistent with our findings. This may be related to glucose-lowering medication in diabetic patients, affecting the blood glucose level and directly influencing the TyG index. In addition, in the diabetic population, traditional cardiovascular risk factors have a more significant impact on cardiovascular events than IR [48].

An essential finding of the study is that glycated hemoglobin has a partial mediating role in the association between TyG, TyG-WC, TyG-WHtR, and TyG-BMI and CVD mortality, total CVD, congestive heart failure, myocardial infarction, angina pectoris, and coronary heart disease. Glycated hemoglobin is the product of combining hemoglobin from red blood cells with sugars from serum. Glycated hemoglobin reflects the average blood glucose level over the last 2–3 months. Some studies have indicated a correlation between increased glycated hemoglobin levels and cardiovascular disease morbidity and mortality [49, 50]. We found a mediating role for insulin in the associations of TyG with total CVD, congestive heart failure, and angina pectoris and TyG-BMI with total CVD and coronary heart disease. Some studies have shown that insulin level increases the risk of cardiovascular disease [43, 51, 52]. Furthermore, the indirect effects of insulin-mediated associations between TyG, TyG-WC, TyG-WHtR, and CVD mortality were -11.2%, -25.1%, and -23.8%. In healthy people, insulin dilates and protects blood vessels. In diabetic populations, insulin is widely used to control blood glucose levels. The relevant literature shows that insulin has a protective effect on the heart and its functions [53–55]. However, further studies are needed to investigate the relationship between insulin and cardiovascular disease. Our findings suggest that effective interventions targeting glycated hemoglobin may be developed to prevent the risk of cardiovascular disease.

The mechanism behind TyG, TyG-WC, TyG-WHtR, and TyG-BMI and increased risk of CVD mortality, total CVD, congestive heart failure, myocardial infarction, angina pectoris, and coronary heart disease may be due to elevated levels of TyG, TyG-WC, TyG-WHtR, and TyG-BMI, which are associated with insulin resistance [56, 57]. Insulin resistance may induce glucose metabolism disorders and lipotoxicity. The release of inflammation factors by macrophages and adipocytes, inactivation of nitric oxide, activation of the sympathetic nervous system and renin–angiotensin–aldosterone system, hemorrhagic disorders, and platelet activation, can lead to cardiac dysfunction and myocardial injury, ultimately resulting in a range of cardiovascular diseases. Experimental research is needed to confirm the specific mechanisms.

## Strengths and limitations

The study's strengths lie in its adjustment for covariates of nutritional data, including total energy intake, AHEI, and dietary supplements. Secondly, this study examines the impact of glycated hemoglobin and insulin on the relationship between TyG, TyG-WC, TyG-WHtR, and TyG-BMI and CVD mortality, total CVD, congestive heart failure, myocardial infarction, angina pectoris, and coronary heart disease. Lastly, the complex multi-stage probability sampling methodology guarantees that the participants in this study accurately represent the non-institutional population, facilitating the generalization of the results to the United States. There are also some limitations to this study. Firstly, data on CVD were collected by self-report, so patients with undiagnosed CVD were omitted. Secondly, the study relied on a single baseline blood sample to gather information on TyG, TyG-WC, TyG-WHtR, and TyG-BMI. Therefore, we could not assess their impact on CVD mortality, total CVD, congestive heart failure, myocardial infarction, angina pectoris, and coronary heart disease over time. Finally, our research is based on data from the United States and excluded people with abnormal energy intake. It remains to be seen whether these findings can be widely applied to other regions, suggesting that further research is warranted.

## Conclusions

TyG-WC and TyG-WtHR enhance CVD mortality prediction, diagnostic efficacy of CVD and its mortality, and some CVD correlation over and above the current hottest TyG. Our study has important implications for identifying people at early risk of cardiovascular disease, improving risk stratification, and reducing the cost of screening, particularly in clinical practice and extensive epidemiological studies.

### Supplementary Information

**Additional file 1.** Mediation analysis of TyG, TyG-WC, TyG-WHtR, and TyG-BMI with CVD mortality, total CVD, congestive heart failure, myocardial infarction, angina pectoris, and coronary heart disease.

**Additional file 2.** Receiver operating characteristic (ROC) curves of TyG, TyG-WC, TyG-WHtR, TyG-BMI, WC, WHtR, and BMI in relation to cardiovascular disease mortality, total CVD, congestive heart failure, myocardial infarction, angina pectoris, and coronary heart disease. A. CVD mortality; B. total CVD; C. congestive heart failure; D. myocardial infarction; E. angina pectoris; F. coronary heart disease.

**Additional file 3.** Supplementary Table 1-31.

## Data Availability

The data were obtained from publicly available sources, as previously stated.

## Abbreviations

TyG: Triglyceride-glucose
CVD: Cardiovascular disease
NHANES: National Health and Nutrition Examination Survey
TyG-WC: Glucose triglyceride-waist circumference
TyG-WHtR: Glucose triglyceride-waist height ratio
TyG-BMI: Glucose triglyceride-body mass index
RCS: Restricted cubic spline
ROC: Receiver operating characteristic
FSG: Fasting blood glucose
OGTT 2 hPG: 2-Hour glucose from an oral glucose tolerance test
HDL-C: High-density lipoprotein-cholesterol
LDL-C: Low-density lipoprotein-cholesterol
SBP: Systolic Blood Pressure
HOMA-IR: Homeostatic Model Assessment of Insulin Resistance
HbA1c: Glycated hemoglobin
AHEI: Alternative healthy eating index
ORs: Odds ratios
CIs: Confidence intervals
