# Supplementary figures and images for: The association between triglyceride-glucose index and its combination with obesity indicators and cardiovascular disease: NHANES 2003–2018
Source: Cardiovasc Diabetol. 2024 Jan 6;23:8. doi: 10.1186/s12933-023-02115-9 (PMC10771672; doi:10.1186/s12933-023-02115-9)

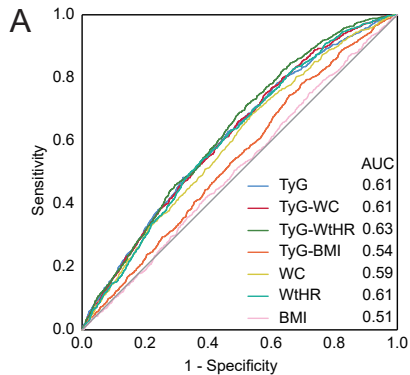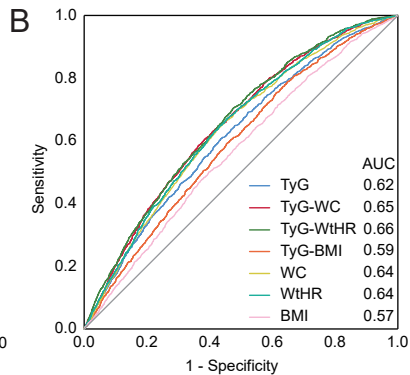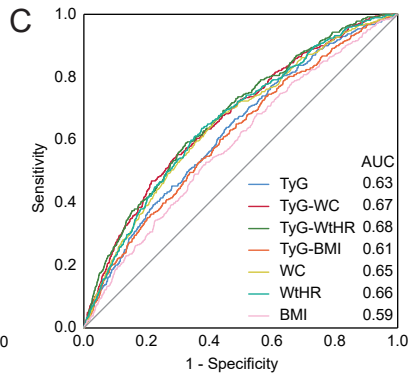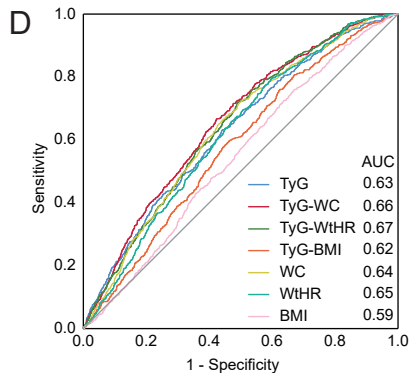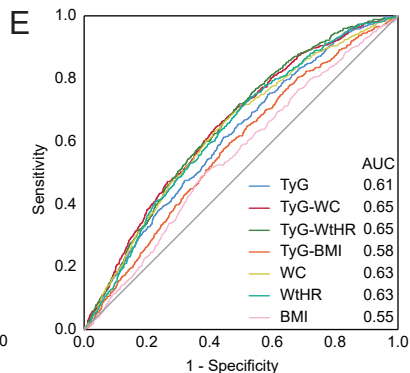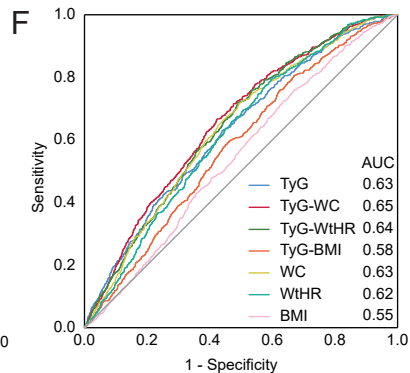

Supplement: Supplementary file 2 — Additional file 2. Receiver operating characteristic (ROC) curves of TyG, TyG-WC, TyG-WHtR, TyG-BMI, WC, WHtR, and BMI in relation to cardiovascular disease mortality, total CVD, congestive heart failure, myocardial infarction, angina pectoris, and coronary heart disease. A. CVD mortality; B. total CVD; C. congestive heart failure; D. myocardial infarction; E. angina pectoris; F. coronary heart disease. [file 12933_2023_2115_MOESM2_ESM.pdf]
