# Supplementary material for: The association between triglyceride-glucose index and its combination with obesity indicators and cardiovascular disease: NHANES 2003–2018
Source: Cardiovasc Diabetol. 2024 Jan 6;23:8. doi: 10.1186/s12933-023-02115-9 (PMC10771672; doi:10.1186/s12933-023-02115-9)
Supplement: Supplementary file 3 — Additional file 3. Supplementary Table 1-31. [file 12933_2023_2115_MOESM3_ESM.docx]

Supplementary Table 1. Baseline characteristics according to triglyceride-glucose-waist circumference (TyG-WC) quartiles: NHANES, 2003 - 2018^acc^

|  | Triglyceride-glucose-waist circumference (TyG-WC) (N = 11,937) | | | | *P* | *P_test_* |
| --- | --- | --- | --- | --- | --- | --- |
|  | ≤ 735.26 | 735.27 - 848.78 | 848.79 - 965.72 | > 965.73 |  |  |
|  | N = 2985 | N = 2984 | N = 2984 | N = 2984 |  |  |
| Age, years | 40.83 (39.82,41.84) | 48.23 (47.41,49.05) | 50.97 (50.21,51.72) | 51.46 (50.63,52.3) | < 0.001 | < 0.001 |
| Male, % | 32.40 (30.20,34.60) | 47.80 (45.60,50.00) | 56.30 (54.20,58.40) | 58.90 (56.80,61.00) | < 0.001 | < 0.001 |
| Non-Hispanic white, % | 69.10 (66.30,71.70) | 68.90 (65.80,71.90) | 71.60 (68.10,74.90) | 75.20 (72.00,78.20) | < 0.001 | < 0.001 |
| BMI, kg/m^2^ | 22.66 (22.51,22.80) | 26.74 (26.60,26.88) | 29.82 (29.66,29.98) | 36.03 (35.73,36.34) | < 0.001 | < 0.001 |
| Smoke, % | 25.30 (22.70,28.10) | 23.10 (21.10,25.30) | 23.00 (21.00,25.10) | 25.30 (23.10,27.60) | < 0.001 | < 0.001 |
| Drink, % | 74.10 (71.90,76.10) | 74.30 (72.00,76.60) | 73.70 (71.50,75.80) | 73.60 (71.10,75.90) | < 0.001 | < 0.001 |
| Regular exercise, % | 31.50 (29.10,34.00) | 39.20 (36.70,41.70) | 45.60 (43.00,48.20) | 53.60 (50.70,56.60) | < 0.001 | < 0.001 |
| College graduate or above, % | 37.30 (34.10,40.60) | 30.70 (28.10,33.40) | 26.80 (24.00,29.70) | 21.20 (18.90,23.80) | < 0.001 | < 0.001 |
| > 100,000 annual household income, % | 25.30 (22.70,28.10) | 23.10 (21.10,25.30) | 23.00 (21.00,25.10) | 25.30 (23.10,27.60) | 0.039 | 0.011 |
| Dietary supplements use, % | 53.50 (51.00,56.00) | 51.40 (49.00,53.80) | 50.40 (48.10,52.70) | 49.50 (47.40,51.70) | < 0.001 | < 0.001 |
| Total energy, kcal/day | 2028 (1997,2060) | 2062 (2023,2102) | 2088 (2053,2124) | 2130 (2092,2167) | < 0.001 | < 0.001 |
| AHEI sore | 51.26 (50.6,51.91) | 50.79 (50.3,51.28) | 49.85 (49.4,50.31) | 48.71 (48.24,49.19) | < 0.001 | < 0.001 |
| Family history of heart disease, % | 13.50 (12.00,15.30) | 15.80 (14.20,17.60) | 17.30 (15.40,19.30) | 19.60 (17.80,21.50) | 0.255 | 0.273 |
| Triglyceride, mmol/L | 0.88 (0.86,0.90) | 1.24 (1.21,1.27) | 1.62 (1.58,1.65) | 2.39 (2.30,2.48) | < 0.001 | < 0.001 |
| Fasting glucose, mmol/L | 5.15 (5.12,5.18) | 5.49 (5.45,5.53) | 5.86 (5.80,5.92) | 6.77 (6.66,6.88) | < 0.001 | < 0.001 |
| Waist circumference, cm | 80.86 (80.54,81.19) | 93.59 (93.35,93.83) | 102.93 (102.66,103.20) | 118.66 (118.05,119.27) | < 0.001 | < 0.001 |
| Glycohemoglobin, % | 5.31 (5.29,5.32) | 5.54 (5.51,5.56) | 5.75 (5.72,5.79) | 6.27 (6.21,6.32) | < 0.001 | < 0.001 |
| Standing height, cm | 165.91 (165.57,166.24) | 167.10 (166.74,167.47) | 168.15 (167.78,168.51) | 169.61 (169.24,169.97) | < 0.001 | < 0.001 |
| Insulin, pmol/L | 40.27 (39.11,41.43) | 58.07 (56.53,59.60) | 81.87 (79.64,84.10) | 133.97 (128.66,139.29) | < 0.001 | < 0.001 |
| Systolic blood pressure, mmHg | 114.10(113.41,114.78) | 120.99(120.23,121.74) | 123.97(123.19,124.74) | 126.21(125.42,127.01) | < 0.001 | < 0.001 |
| Cholesterol, mmol/L | 4.83 (4.79,4.88) | 5.13 (5.08,5.17) | 5.22 (5.17,5.26) | 5.19 (5.13,5.25) | < 0.001 | < 0.001 |
| Self-reported cancer, % | 7.10 (5.90,8.60) | 9.50 (8.00,11.20) | 10.70 (9.40,12.30) | 11.20 (10.00,12.60) | < 0.001 | < 0.001 |
| Self-reported diabetes, % | 1.40 (1.00,2.00) | 4.10 (3.30,5.10) | 9.20 (8.00,10.50) | 20.50 (18.70,22.30) | < 0.001 | < 0.001 |
| Death of cardiovascular diseases, % | 1.60(1.20,2.20) | 3.70(3.10,4.50) | 4.20(3.40,5.10) | 6.70(5.80,7.70) | < 0.001 | < 0.001 |
| Cardiovascular diseases, % | 3.80(2.90,4.90) | 7.30(6.20,8.60) | 9.40(8.00,10.90) | 15.40(13.70,17.30) | < 0.001 | < 0.001 |
| Congestive heart failure, % | 1.10(0.70,1.80) | 1.60(1.10,2.20) | 2.00(1.50,2.60) | 5.30(4.30,6.40) | < 0.001 | < 0.001 |
| Myocardial infarction, % | 1.50(1.00,2.30) | 2.80(2.20,3.50) | 3.70(3.00,4.60) | 6.40(5.40,7.50) | < 0.001 | < 0.001 |
| Angina pectoris, % | 0.90(0.60,1.50) | 1.60(1.20,2.20) | 2.70(2.00,3.50) | 4.40(3.60,5.30) | < 0.001 | < 0.001 |
| Coronary heart disease, % | 1.40(0.90,2.10) | 2.70(2.10,3.60) | 4.10(3.30,5.10) | 6.90(5.80,8.30) | < 0.001 | < 0.001 |

^a^Continuous variables were listed as weighted mean (95%CI). Categorical variables were listed as weighted percentage (95%CI). After adjusting for age, general linear models and chi-square tests were conducted to compare continuous and categorical baseline characteristics, respectively. And *P*_test_ was the result of Bonfreni correction.

Supplementary Table 2. Baseline characteristics according to triglyceride-glucose-waist-to-height ratio (TyG-WtHR) quartiles: NHANES, 2003-2018^a^

|  | Triglyceride-glucose-waist-to-height ratio (TyG-WtHR) (N = 11,937) | | | | *P* | *P_test_* |
| --- | --- | --- | --- | --- | --- | --- |
|  | ≤ 4.39 | 4.40 - 5.06 | 5.07 - 5.79 | > 5.80 |  |  |
|  | N = 2985 | N = 2984 | N = 2984 | N = 2984 |  |  |
| Age, years | 40.35(39.44,41.26) | 48.17(47.34,48.99) | 51.12(50.38,51.85) | 52.68(51.83,53.54) | < 0.001 | < 0.001 |
| Male, % | 44.00(41.90,46.20) | 52.80(50.70,54.90) | 52.60(50.40,54.70) | 44.40(42.30,46.60) | < 0.001 | < 0.001 |
| Non-Hispanic white, % | 71.30(68.50,74.00) | 71.00(68.10,73.80) | 71.00(67.80,74.00) | 71.30(67.50,74.80) | < 0.001 | < 0.001 |
| BMI, kg/m^2^ | 22.76(22.63,22.88) | 26.81(26.66,26.96) | 30.07(29.91,30.23) | 36.58(36.28,36.87) | < 0.001 | < 0.001 |
| Smoke, % | 27.20(24.60,29.90) | 24.20(22.10,26.40) | 22.00(19.80,24.40) | 22.80(20.90,24.80) | < 0.001 | < 0.001 |
| Drink, % | 77.60(75.50,79.50) | 75.70(73.40,77.90) | 72.60(70.30,74.90) | 68.70(66.20,71.10) | < 0.001 | < 0.001 |
| Regular exercise, % | 29.80(27.50,32.10) | 39.50(37.00,42.00) | 45.40(42.60,48.10) | 57.30(54.50,60.10) | < 0.001 | < 0.001 |
| College graduate or above, % | 39.30(36.00,42.70) | 31.00(28.20,33.90) | 25.10(22.80,27.60) | 19.00(17.00,21.10) | < 0.001 | < 0.001 |
| > 100,000 annual household income, % | 19.60(16.70,22.80) | 17.90(15.30,20.90) | 13.40(11.00,16.30) | 8.50(7.00,10.30) | 0.298 | 0.289 |
| Dietary supplements use, % | 51.80(49.20,54.50) | 51.80(49.30,54.30) | 49.40(47.40,51.50) | 51.90(49.60,54.20) | < 0.001 | < 0.001 |
| Total energy, kcal/day | 2134(2105,2162) | 2092(2054,2129) | 2053(2020,2086) | 2009(1964,2055) | < 0.001 | < 0.001 |
| AHEI sore | 50.72(50.11,51.32) | 50.18(49.61,50.76) | 50.00(49.58,50.42) | 49.72(49.23,50.21) | < 0.001 | < 0.001 |
| Family history of heart disease, % | 13.70(12.10,15.40) | 15.30(13.50,17.30) | 17.60(15.70,19.60) | 20.20(18.40,22.00) | 0.015 | 0.007 |
| Triglyceride, mmol/L | 0.88(0.86,0.90) | 1.27(1.24,1.30) | 1.71(1.67,1.75) | 2.37(2.27,2.46) | < 0.001 | < 0.001 |
| Fasting glucose, mmol/L | 5.17(5.13,5.20) | 5.52(5.48,5.55) | 5.86(5.80,5.91) | 6.84(6.72,6.96) | < 0.001 | < 0.001 |
| Waist circumference, cm | 82.12(81.74,82.51) | 94.58(94.24,94.91) | 103.31(102.94,103.69) | 118.39(117.75,119.02) | < 0.001 | < 0.001 |
| Glycohemoglobin, % | 5.30 (5.28,5.32) | 5.52 (5.50,5.55) | 5.74 (5.71,5.77) | 6.30 (6.25,6.36) | < 0.001 | < 0.001 |
| Standing height, cm | 169.35 (169.01,169.70) | 168.49 (168.14,168.85) | 167.31 (166.94,167.67) | 165.61 (165.25,165.98) | < 0.001 | < 0.001 |
| Insulin, pmol/L | 39.61 (38.47,40.74) | 59.02 (57.39,60.65) | 83.27 (80.93,85.61) | 132.48 (127.20,137.77) | < 0.001 | < 0.001 |
| Systolic blood pressure, mmHg | 114.36(113.63,115.09) | 120.87(120.10,121.64) | 123.87(123.13,124.60) | 126.96(126.15,127.78) | < 0.001 | < 0.001 |
| Cholesterol, mmol/L | 4.82(4.77,4.86) | 5.12(5.07,5.17) | 5.24(5.19,5.28) | 5.22(5.16,5.28) | < 0.001 | < 0.001 |
| Self-reported cancer, % | 7.00(5.90,8.30) | 9.30 (7.90,11.00) | 11.00 (9.80,12.40) | 11.50 (10.20,13.00) | < 0.001 | < 0.001 |
| Self-reported diabetes, % | 1.30 (0.90,1.80) | 3.90 (3.20,4.70) | 9.40 (8.00,11.00) | 22.20 (20.20,24.20) | < 0.001 | < 0.001 |
| Death of cardiovascular diseases, % | 1.40(1.10,1.90) | 3.30(2.80,4.00) | 4.70(3.90,5.70) | 7.20(6.30,8.30) | < 0.001 | < 0.001 |
| Cardiovascular diseases, % | 3.80(3.00,4.90) | 6.70(5.70,7.80) | 10.50(9.00,12.10) | 15.70(13.90,17.80) | < 0.001 | < 0.001 |
| Congestive heart failure, % | 1.10(0.70,1.70) | 1.50(1.10,2.00) | 2.50(1.90,3.10) | 5.20(4.30,6.30) | < 0.001 | < 0.001 |
| Myocardial infarction, % | 1.40(1.00,2.10) | 2.60(2.10,3.30) | 4.30(3.50,5.20) | 6.50(5.60,7.60) | < 0.001 | < 0.001 |
| Angina pectoris, % | 1.00(0.60,1.50) | 1.50(1.10,2.00) | 3.10(2.40,4.00) | 4.30(3.50,5.30) | < 0.001 | < 0.001 |
| Coronary heart disease, % | 1.50(1.10,2.10) | 2.80(2.10,3.60) | 4.80(3.90,5.90) | 6.40(5.40,7.70) | < 0.001 | < 0.001 |

^a^Continuous variables were listed as weighted mean (95%CI). Categorical variables were listed as weighted percentage (95%CI). After adjusting for age, general linear models and chi-square tests were conducted to compare continuous and categorical baseline characteristics, respectively. And *P*_test_ was the result of Bonfreni correction.

Supplementary Table 3. Baseline characteristics according to triglyceride-glucose-body mass index (TyG-BMI) quartiles: NHANES, 2003-2018^a^

|  | Triglyceride-glucose-body mass index (TyG-BMI) (N = 11,937) | | | | *P* | *P_test_* |
| --- | --- | --- | --- | --- | --- | --- |
|  | ≤ 204.75 | 204.76 - 242.30 | 242.31 - 285.65 | > 285.66 |  |  |
|  | N = 2985 | N = 2983 | N = 2985 | N = 2984 |  |  |
| Age, years | 43.24 (42.2,44.27) | 49.05 (48.29,49.8) | 49.64 (48.85,50.43) | 49.21 (48.4,50.03) | < 0.001 | < 0.001 |
| Male, % | 39.30 (37.00,41.60) | 52.80 (50.40,55.10) | 55.00 (52.90,57.10) | 47.30 (45.10,49.50) | < 0.001 | < 0.001 |
| Non-Hispanic white, % | 72.20 (69.50,74.80) | 72.10 (69.10,74.90) | 70.00 (66.50,73.30) | 70.10 (66.60,73.40) | < 0.001 | < 0.001 |
| BMI, kg/m^2^ | 22.06 (21.96,22.16) | 26.24 (26.15,26.32) | 29.84 (29.74,29.94) | 37.19 (36.92,37.46) | < 0.001 | < 0.001 |
| Smoke, % | 26.60 (24.00,29.30) | 24.60 (22.40,26.90) | 22.70 (20.70,24.90) | 22.60 (20.60,24.70) | < 0.001 | < 0.001 |
| Drink, % | 74.20 (72.10,76.20) | 76.40 (74.20,78.50) | 74.80 (72.50,76.90) | 70.20 (67.70,72.60) | < 0.001 | < 0.001 |
| Regular exercise, % | 33.50 (31.20,36.00) | 39.20 (36.60,41.90) | 44.50 (41.80,47.20) | 52.50 (49.70,55.20) | < 0.001 | < 0.001 |
| College graduate or above, % | 37.30 (34.20,40.60) | 31.30 (28.40,34.40) | 26.40 (23.70,29.20) | 20.90 (18.80,23.30) | < 0.001 | < 0.001 |
| > 100,000 annual household income, % | 18.90 (16.00,22.30) | 17.00 (14.50,19.90) | 14.00 (11.30,17.10) | 10.50 (8.70,12.50) | 0.084 | 0.021 |
| Dietary supplements use, % | 53.80 (51.00,56.50) | 53.90 (51.50,56.30) | 48.30 (46.10,50.50) | 48.70 (46.60,50.90) | < 0.001 | < 0.001 |
| Total energy, kcal/day | 2067 (203,2097) | 2084 (2047,2120) | 2076 (2039,2112) | 2077 (2039,2115) | < 0.001 | < 0.001 |
| AHEI sore | 51.13 (50.5,51.77) | 50.56 (50.06,51.06) | 49.97 (49.49,50.45) | 48.97 (48.55,49.39) | < 0.001 | < 0.001 |
| Family history of heart disease, % | 18.90 (12.20,15.40) | 17.00 (14.00,17.80) | 14.00 (14.50,18.40) | 10.50 (18.40,22.50) | 0.374 | 0.463 |
| Triglyceride, mmol/L | 0.91 (0.89,0.93) | 1.28 (1.25,1.31) | 1.71 (1.66,1.77) | 2.21 (2.12,2.30) | < 0.001 | < 0.001 |
| Fasting glucose, mmol/L | 5.21 (5.18,5.24) | 5.57 (5.52,5.62) | 5.84 (5.79,5.90) | 6.64 (6.53,6.74) | < 0.001 | < 0.001 |
| Waist circumference, cm | 81.69 (81.31,82.08) | 93.82 (93.52,94.13) | 102.45 (102.08,102.82) | 117.96 (117.37,118.54) | < 0.001 | < 0.001 |
| Glycohemoglobin, % | 5.36 (5.34,5.38) | 5.56 (5.54,5.59) | 5.75 (5.72,5.79) | 6.19 (6.13,6.24) | < 0.001 | < 0.001 |
| Standing height, cm | 167.61 (167.26,167.96) | 168.15 (167.79,168.51) | 167.80 (167.44,168.17) | 167.20 (166.83,167.57) | < 0.001 | < 0.001 |
| Insulin, pmol/L | 39.64 (38.49,40.80) | 57.13 (55.53,58.73) | 82.88 (80.55,85.21) | 134.72 (129.47,139.97) | < 0.001 | < 0.001 |
| Systolic blood pressure, mmHg | 115.57(114.78,116.35) | 120.78(120.06,121.51) | 123.33(122.62,124.05) | 125.39(124.69,126.09) | < 0.001 | < 0.001 |
| Cholesterol, mmol/L | 4.86 (4.81,4.90) | 5.12 (5.07,5.18) | 5.23 (5.18,5.28) | 5.15 (5.10,5.21) | < 0.001 | < 0.001 |
| Self-reported cancer, % | 9.10 (7.80,10.60) | 9.80 (8.60,11.30) | 9.60 (8.40,11.00) | 9.80 (8.70,11.00) | < 0.001 | < 0.001 |
| Self-reported diabetes, % | 2.10 (1.60,2.70) | 5.10 (4.20,6.20) | 8.60 (7.50,9.80) | 19.30 (17.60,21.30) | < 0.001 | < 0.001 |
| Death of cardiovascular diseases, % | 2.70(2.20,3.30) | 3.80(3.10,4.60) | 4.30(3.60,5.00) | 5.40(4.60,6.30) | < 0.001 | < 0.001 |
| Cardiovascular diseases, % | 5.40(4.40,6.70) | 8.10(6.80,9.70) | 9.40(8.20,10.80) | 12.60(11.10,14.30) | < 0.001 | < 0.001 |
| Congestive heart failure, % | 1.50(1.10,2.20) | 1.80(1.30,2.50) | 2.40(1.80,3.20) | 4.10(3.30,5.10) | < 0.001 | < 0.001 |
| Myocardial infarction, % | 2.40(1.80,3.20) | 3.20(2.50,4.10) | 3.60(2.90,4.40) | 5.20(4.30,6.20) | < 0.001 | < 0.001 |
| Angina pectoris, % | 1.40(0.90,2.00) | 1.70(1.30,2.40) | 2.90(2.30,3.70) | 3.50(2.90,4.40) | < 0.001 | < 0.001 |
| Coronary heart disease, % | 2.30(1.70,3.10) | 3.30(2.60,4.30) | 4.30(3.50,5.30) | 5.20(4.10,6.40) | < 0.001 | < 0.001 |

^a^Continuous variables were listed as weighted mean (95%CI). Categorical variables were listed as weighted percentage (95%CI). After adjusting for age, general linear models and chi-square tests were conducted to compare continuous and categorical baseline characteristics, respectively. And *P*_test_ was the result of Bonfreni correction.

Supplementary Table 4. Association of triglyceride-glucose (TyG) with CVD mortality, total cardiovascular disease (Total-CVD), congestive heart failure, myocardial infarction, angina pectoris and coronary heart disease.

|  |  | Triglyceride-glucose (TyG) | | | | | | | *P*_trend_^e^ | *P*_test_^f^ |
| --- | --- | --- | --- | --- | --- | --- | --- | --- | --- | --- |
|  |  | Q1^c^ | Q2 | | Q3 | | Q4 | |  |  |
|  |  |  | HR/OR^b^ | 95%CI | HR/OR | 95%CI | HR/OR | 95%CI |  |  |
| CVD mortality |  |  |  |  |  |  |  |  |  |  |
|  | Model 1^a^ | Ref (1.00)^d^ | 0.98 | (0.98,1.32) | 0.95 | (0.69,1.30) | 1.49 | **(1.10,2.03)*** | 0.002 | 0.034 |
|  | Model 2 | Ref (1.00) | 0.90 | (0.67,1.21) | 0.87 | (0.63,1.19) | 1.29 | (0.95,1.76) | 0.016 | 0.307 |
|  | Model 3 | Ref (1.00) | 0.88 | (0.65,1.19) | 0.84 | (0.61,1.16) | 1.25 | (0.92,1.70) | 0.026 | 0.471 |
|  | Model 4 | Ref (1.00) | 0.89 | (0.65,1.21) | 0.86 | (0.61,1.22) | 1.19 | (0.85,1.67) | 0.096 | 0.930 |
| Total-CVD |  |  |  |  |  |  |  |  |  |  |
|  | Model 1 | Ref (1.00) | 1.14 | (0.89,1.46) | 1.39 | **(1.09,1.77)**** | 1.91 | **(1.56,2.35)***** | < 0.001 | < 0.001 |
|  | Model 2 | Ref (1.00) | 1.10 | (0.85,1.41) | 1.32 | **(1.04,1.69)*** | 1.79 | **(1.45,2.22)***** | < 0.001 | < 0.001 |
|  | Model 3 | Ref (1.00) | 1.09 | (0.85,1.41) | 1.30 | **(1.02,1.67)*** | 1.76 | **(1.43,2.18)***** | < 0.001 | < 0.001 |
|  | Model 4 | Ref (1.00) | 1.21 | (0.93,1.56) | 1.49 | **(1.17,1.91)**** | 2.08 | **(1.66,2.60)***** | < 0.001 | < 0.001 |
| Congestive heart failure |  |  |  |  |  |  |  |  |  |  |
|  | Model 1 | Ref (1.00) | 1.11 | (0.76,1.63) | 1.39 | (0.86,2.23) | 2.30 | **(1.48,3.58)***** | < 0.001 | 0.001 |
|  | Model 2 | Ref (1.00) | 1.05 | (0.72,1.53) | 1.29 | (0.81,2.06) | 2.05 | **(1.32,3.18)**** | < 0.001 | 0.005 |
|  | Model 3 | Ref (1.00) | 1.05 | (0.71,1.53) | 1.28 | (0.80,2.05) | 2.02 | **(1.30,3.12)**** | < 0.001 | 0.006 |
|  | Model 4 | Ref (1.00) | 1.09 | (0.73,1.63) | 1.31 | (0.80,2.15) | 1.90 | **(1.18,3.04)**** | 0.002 | 0.025 |
| Myocardial infarction |  |  |  |  |  |  |  |  |  |  |
|  | Model 1 | Ref (1.00) | 1.32 | (0.83,2.09) | 1.45 | (0.98,2.13) | 1.87 | **(1.32,2.64)*** | < 0.001 | 0.002 |
|  | Model 2 | Ref (1.00) | 1.27 | (0.80,2.03) | 1.38 | (0.94,2.03) | 1.76 | **(1.23,2.50)**** | 0.001 | 0.006 |
|  | Model 3 | Ref (1.00) | 1.25 | (0.78,2.01) | 1.33 | (0.90,1.98) | 1.68 | **(1.17,2.40)**** | 0.002 | 0.016 |
|  | Model 4 | Ref (1.00) | 1.42 | (0.88,2.30) | 1.57 | **(1.06,2.34)*** | 2.13 | **(1.43,3.18)***** | < 0.001 | 0.001 |
| Angina pectoris |  |  |  |  |  |  |  |  |  |  |
|  | Model 1 | Ref (1.00) | 1.00 | (0.61,1.63) | 1.72 | **(1.07,2.76)*** | 1.99 | **(1.32,3.01)**** | < 0.001 | 0.004 |
|  | Model 2 | Ref (1.00) | 0.95 | (0.57,1.57) | 1.62 | **(1.01,2.62)*** | 1.83 | **(1.19,2.81)**** | 0.001 | 0.019 |
|  | Model 3 | Ref (1.00) | 0.95 | (0.57,1.58) | 1.62 | **(1.00,2.61)*** | 1.81 | **(1.17,2.79)**** | 0.001 | 0.023 |
|  | Model 4 | Ref (1.00) | 1.05 | (0.63,1.76) | 1.83 | **(1.11,3.02)*** | 2.14 | **(1.34,3.41)**** | < 0.001 | 0.005 |
| Coronary heart disease |  |  |  |  |  |  |  |  |  |  |
|  | Model 1 | Ref (1.00) | 1.10 | (0.72,1.69) | 1.53 | **(1.02,2.31)*** | 2.12 | **(1.46,3.09)***** | < 0.001 | < 0.001 |
|  | Model 2 | Ref (1.00) | 1.08 | (0.70,1.67) | 1.48 | (0.98,2.23) | 2.05 | **(1.42,2.98)***** | < 0.001 | 0.001 |
|  | Model 3 | Ref (1.00) | 1.08 | (0.70,1.67) | 1.47 | (0.97,2.22) | 2.03 | **(1.39,2.96)***** | < 0.001 | 0.001 |
|  | Model 4 | Ref (1.00) | 1.20 | (0.77,1.86) | 1.70 | **(1.12,2.56)*** | 2.52 | **(1.66,3.83)***** | < 0.001 | < 0.001 |

^a^Model 1 was adjusted for age, sex, ethnicity, and year. Model 2 further adjusted for smoke, drink, exercise, education, income and family history of heart disease. Model 3 further adjusted for energy, AHEI, nutrient supplement use. Model 4 further adjusted for SBP, cholesterol, self-reported cancer, and self-reported diabetes.

^b^Data were listed as the weighted hazard/odd ratio estimates and 95% confidence intervals, with *p < 0.05, **p < 0.01, ***p < 0.001.

^c^Q, quintile.

^d^Ref, reference.

^e^Tests for trends based on the variables containing the median values for each quartile.

^f^*P*_test_ was the result of Bonfreni correction.

Supplementary Table 5. Association of triglyceride-glucose-waist circumference (TyG-WC) with CVD mortality, total cardiovascular disease (Total-CVD), congestive heart failure, myocardial infarction, angina pectoris and coronary heart disease.

|  |  | Triglyceride-glucose-waist circumference (TyG-WC) | | | | | | | *P*_trend_^e^ | *P*_test_^f^ |
| --- | --- | --- | --- | --- | --- | --- | --- | --- | --- | --- |
|  |  | Q1^c^ | Q2 | | Q3 | | Q4 | |  |  |
|  |  |  | HR/OR^b^ | 95%CI | HR/OR | 95%CI | HR/OR | 95%CI |  |  |
| CVD mortality |  |  |  |  |  |  |  |  |  |  |
|  | Model 1^a^ | Ref (1.00)^d^ | 1.21 | (0.89,1.66) | 1.13 | (0.84,1.53) | 2.04 | **(1.48,2.82)***** | < 0.001 | < 0.001 |
|  | Model 2 | Ref (1.00) | 1.20 | (0.89,1.63) | 1.06 | (0.79,1.41) | 1.79 | **(1.31,2.44)***** | < 0.001 | 0.001 |
|  | Model 3 | Ref (1.00) | 1.18 | (0.87,1.61) | 1.03 | (0.77,1.37) | 1.73 | **(1.27,2.36)**** | < 0.001 | 0.002 |
|  | Model 4 | Ref (1.00) | 1.16 | (0.86,1.57) | 0.97 | (0.72,1.30) | 1.58 | **(1.15,2.15)**** | 0.002 | 0.014 |
| Total-CVD |  |  |  |  |  |  |  |  |  |  |
|  | Model 1 | Ref (1.00) | 1.24 | (0.88,1.73) | 1.40 | **(1.05,1.87)*** | 2.67 | **(2.03,3.53)***** | < 0.001 | < 0.001 |
|  | Model 2 | Ref (1.00) | 1.21 | (0.86,1.71) | 1.35 | **(1.01,1.81)*** | 2.51 | **(1.89,3.34)***** | < 0.001 | < 0.001 |
|  | Model 3 | Ref (1.00) | 1.19 | (0.85,1.69) | 1.31 | (0.98,1.77) | 2.45 | **(1.85,3.25)***** | < 0.001 | < 0.001 |
|  | Model 4 | Ref (1.00) | 1.26 | (0.88,1.80) | 1.34 | (0.98,1.83) | 2.37 | **(1.77,3.17)***** | < 0.001 | < 0.001 |
| Congestive heart failure |  |  |  |  |  |  |  |  |  |  |
|  | Model 1 | Ref (1.00) | 0.86 | (0.48,1.54) | 0.97 | (0.66,1.43) | 2.83 | **(1.73,4.64)***** | < 0.001 | < 0.001 |
|  | Model 2 | Ref (1.00) | 0.85 | (0.48,1.51) | 0.92 | (0.63,1.35) | 2.59 | **(1.59,4.23)***** | < 0.001 | 0.001 |
|  | Model 3 | Ref (1.00) | 0.84 | (0.47,1.49) | 0.90 | (0.62,1.33) | 2.55 | **(1.57,4.16)***** | < 0.001 | 0.001 |
|  | Model 4 | Ref (1.00) | 0.86 | (0.48,1.52) | 0.84 | (0.57,1.24) | 2.14 | **(1.31,3.51)**** | < 0.001 | 0.009 |
| Myocardial infarction |  |  |  |  |  |  |  |  |  |  |
|  | Model 1 | Ref (1.00) | 1.07 | (0.69,1.65) | 1.23 | (0.84,1.81) | 2.21 | **(1.49,3.27)***** | < 0.001 | < 0.001 |
|  | Model 2 | Ref (1.00) | 1.05 | (0.67,1.64) | 1.19 | (0.80,1.75) | 2.06 | **(1.38,3.09)**** | < 0.001 | 0.002 |
|  | Model 3 | Ref (1.00) | 1.03 | (0.65,1.62) | 1.14 | (0.76,1.70) | 1.95 | **(1.30,2.93)**** | < 0.001 | 0.005 |
|  | Model 4 | Ref (1.00) | 1.10 | (0.70,1.72) | 1.18 | (0.79,1.76) | 1.92 | **(1.26,2.93)**** | < 0.001 | 0.008 |
| Angina pectoris |  |  |  |  |  |  |  |  |  |  |
|  | Model 1 | Ref (1.00) | 1.08 | (0.61,1.93) | 1.53 | (0.98,2.39) | 2.57 | **(1.52,4.35)**** | < 0.001 | 0.002 |
|  | Model 2 | Ref (1.00) | 1.06 | (0.60,1.89) | 1.46 | (0.94,2.29) | 2.39 | **(1.42,4.02)**** | < 0.001 | 0.004 |
|  | Model 3 | Ref (1.00) | 1.06 | (0.60,1.89) | 1.45 | (0.93,2.27) | 2.38 | **(1.42,3.99)**** | < 0.001 | 0.004 |
|  | Model 4 | Ref (1.00) | 1.15 | (0.65,2.03) | 1.53 | (0.98,2.41) | 2.38 | **(1.43,3.97)**** | < 0.001 | 0.003 |
| Coronary heart disease |  |  |  |  |  |  |  |  |  |  |
|  | Model 1 | Ref (1.00) | 1.03 | (0.64,1.65) | 1.29 | (0.84,1.98) | 2.29 | **(1.51,3.48)***** | < 0.001 | < 0.001 |
|  | Model 2 | Ref (1.00) | 1.00 | (0.62,1.62) | 1.24 | (0.81,1.91) | 2.19 | **(1.45,3.31)***** | < 0.001 | 0.001 |
|  | Model 3 | Ref (1.00) | 1.00 | (0.62,1.62) | 1.23 | (0.80,1.89) | 2.17 | **(1.44,3.26)***** | < 0.001 | 0.001 |
|  | Model 4 | Ref (1.00) | 1.05 | (0.65,1.71) | 1.26 | (0.82,1.93) | 2.10 | **(1.41,3.13)***** | < 0.001 | 0.001 |

^a^Model 1 was adjusted for age, sex, ethnicity, and year. Model 2 further adjusted for smoke, drink, exercise, education, income and family history of heart disease. Model 3 further adjusted for energy, AHEI, nutrient supplement use. Model 4 further adjusted for SBP, cholesterol, self-reported cancer, and self-reported diabetes.

^b^Data were listed as the weighted hazard/odd ratio estimates and 95% confidence intervals, with *p < 0.05, **p < 0.01, ***p < 0.001.

^c^Q, quintile.

^d^Ref, reference.

^e^Tests for trends based on the variables containing the median values for each quartile.

^f^*P*_test_ was the result of Bonfreni correction.

Supplementary Table 6. Association of triglyceride-glucose-waist-to-height ratio (TyG-WtHR) with CVD mortality, total cardiovascular disease (Total-CVD), congestive heart failure, myocardial infarction, angina pectoris and coronary heart disease.

|  |  | Triglyceride-glucose-waist-to-height ratio (TyG-WtHR) | | | | | | | *P*_trend_^e^ | *P*_test_^f^ |
| --- | --- | --- | --- | --- | --- | --- | --- | --- | --- | --- |
|  |  | Q1^c^ | Q2 | | Q3 | | Q4 | |  |  |
|  |  |  | HR/OR^b^ | 95%CI | HR/OR | 95%CI | HR/OR | 95%CI |  |  |
| CVD mortality |  |  |  |  |  |  |  |  |  |  |
|  | Model 1^a^ | Ref (1.00)^d^ | 1.20 | (0.88,1.63) | 1.40 | **(1.05,1.86)*** | 2.26 | **(1.64,3.12)***** | < 0.001 | < 0.001 |
|  | Model 2 | Ref (1.00) | 1.14 | (0.83,1.55) | 1.27 | (0.95,1.69) | 1.93 | **(1.39,2.67)***** | < 0.001 | < 0.001 |
|  | Model 3 | Ref (1.00) | 1.10 | (0.80,1.51) | 1.22 | (0.92,1.62) | 1.83 | **(1.33,2.51)***** | < 0.001 | 0.001 |
|  | Model 4 | Ref (1.00) | 1.09 | (0.81,1.47) | 1.18 | (0.89,1.56) | 1.66 | **(1.21,2.29)**** | < 0.001 | 0.007 |
| Total-CVD |  |  |  |  |  |  |  |  |  |  |
|  | Model 1 | Ref (1.00) | 1.06 | (0.78,1.43) | 1.54 | **(1.19,1.98)**** | 2.57 | **(1.94,3.40)***** | < 0.001 | < 0.001 |
|  | Model 2 | Ref (1.00) | 1.03 | (0.75,1.39) | 1.47 | **(1.13,1.91)**** | 2.40 | **(1.80,3.20)***** | < 0.001 | < 0.001 |
|  | Model 3 | Ref (1.00) | 0.99 | (0.73,1.36) | 1.41 | **(1.09,1.83)*** | 2.30 | **(1.73,3.06)***** | < 0.001 | < 0.001 |
|  | Model 4 | Ref (1.00) | 1.06 | (0.78,1.45) | 1.49 | **(1.12,1.96)**** | 2.27 | **(1.69,3.06)***** | < 0.001 | < 0.001 |
| Congestive heart failure |  |  |  |  |  |  |  |  |  |  |
|  | Model 1 | Ref (1.00) | 0.80 | (0.49,1.33) | 1.16 | (0.74,1.82) | 2.58 | **(1.53,4.34)***** | < 0.001 | 0.001 |
|  | Model 2 | Ref (1.00) | 0.77 | (0.47,1.27) | 1.08 | (0.68,1.69) | 2.28 | **(1.36,3.84)**** | < 0.001 | 0.006 |
|  | Model 3 | Ref (1.00) | 0.76 | (0.46,1.25) | 1.04 | (0.66,1.64) | 2.20 | **(1.31,3.70)**** | < 0.001 | 0.010 |
|  | Model 4 | Ref (1.00) | 0.78 | (0.47,1.29) | 0.99 | (0.62,1.56) | 1.88 | **(1.10,3.21)*** | 0.002 | 0.065 |
| Myocardial infarction |  |  |  |  |  |  |  |  |  |  |
|  | Model 1 | Ref (1.00) | 1.11 | (0.73,1.70) | 1.58 | **(1.12,2.21)**** | 2.55 | **(1.70,3.81)***** | < 0.001 | < 0.001 |
|  | Model 2 | Ref (1.00) | 1.08 | (0.70,1.67) | 1.51 | **(1.07,2.15)*** | 2.38 | **(1.56,3.61)***** | < 0.001 | < 0.001 |
|  | Model 3 | Ref (1.00) | 1.04 | (0.67,1.62) | 1.42 | (0.99,2.04) | 2.20 | **(1.45,3.34)***** | < 0.001 | 0.001 |
|  | Model 4 | Ref (1.00) | 1.13 | (0.73,1.76) | 1.52 | **(1.07,2.16)*** | 2.24 | **(1.45,3.44)***** | < 0.001 | 0.001 |
| Angina pectoris |  |  |  |  |  |  |  |  |  |  |
|  | Model 1 | Ref (1.00) | 0.92 | (0.56,1.52) | 1.72 | **(1.13,2.62)*** | 2.45 | **(1.55,3.88)***** | < 0.001 | 0.001 |
|  | Model 2 | Ref (1.00) | 0.88 | (0.54,1.46) | 1.62 | **(1.06,2.48)*** | 2.25 | **(1.43,3.55)**** | < 0.001 | 0.002 |
|  | Model 3 | Ref (1.00) | 0.87 | (0.53,1.44) | 1.59 | **(1.03,2.46)*** | 2.19 | **(1.40,3.45)**** | < 0.001 | 0.003 |
|  | Model 4 | Ref (1.00) | 0.94 | (0.57,1.55) | 1.70 | **(1.10,2.63)*** | 2.25 | **(1.42,3.57)**** | < 0.001 | 0.002 |
| Coronary heart disease |  |  |  |  |  |  |  |  |  |  |
|  | Model 1 | Ref (1.00) | 1.02 | (0.65,1.58) | 1.58 | **(1.11,2.24)**** | 2.29 | **(1.57,3.35)***** | < 0.001 | < 0.001 |
|  | Model 2 | Ref (1.00) | 0.99 | (0.63,1.54) | 1.52 | **(1.07,2.15)*** | 2.20 | **(1.52,3.17)***** | < 0.001 | < 0.001 |
|  | Model 3 | Ref (1.00) | 0.97 | (0.62,1.52) | 1.49 | **(1.06,2.11)*** | 2.15 | **(1.48,3.11)***** | < 0.001 | < 0.001 |
|  | Model 4 | Ref (1.00) | 1.03 | (0.67,1.61) | 1.55 | **(1.10,2.17)*** | 2.11 | **(1.46,3.04)***** | < 0.001 | < 0.001 |

^a^Model 1 was adjusted for age, sex, ethnicity, and year. Model 2 further adjusted for smoke, drink, exercise, education, income and family history of heart disease. Model 3 further adjusted for energy, AHEI, nutrient supplement use. Model 4 further adjusted for SBP, cholesterol, self-reported cancer, and self-reported diabetes.

^b^Data were listed as the weighted hazard/odd ratio estimates and 95% confidence intervals, with *p < 0.05, **p < 0.01, ***p < 0.001.

^c^Q, quintile.

^d^Ref, reference.

^e^Tests for trends based on the variables containing the median values for each quartile.

^f^*P*_test_ was the result of Bonfreni correction.

Supplementary Table 7. Association of triglyceride-glucose-body mass index (TyG-BMI) with CVD mortality, total cardiovascular disease (Total-CVD), congestive heart failure, myocardial infarction, angina pectoris and coronary heart disease.

|  |  | Triglyceride-glucose-body mass index (TyG-BMI) | | | | | | | *P*_trend_^e^ | *P*_test_^f^ |
| --- | --- | --- | --- | --- | --- | --- | --- | --- | --- | --- |
|  |  | Q1^c^ | Q2 | | Q3 | | Q4 | |  |  |
|  |  |  | HR/OR^b^ | 95%CI | HR/OR | 95%CI | HR/OR | 95%CI |  |  |
| CVD mortality |  |  |  |  |  |  |  |  |  |  |
|  | Model 1^a^ | Ref (1.00) ^d^ | 0.92 | (0.72,1.17) | 1.06 | (0.82,1.38) | 1.77 | **(1.38,2.28)***** | < 0.001 | < 0.001 |
|  | Model 2 | Ref (1.00) | 0.88 | (0.69,1.12) | 1.00 | (0.77,1.30) | 1.55 | **(1.21,1.98)**** | < 0.001 | 0.002 |
|  | Model 3 | Ref (1.00) | 0.87 | (0.68,1.10) | 0.98 | (0.75,1.27) | 1.51 | **(1.18,1.93)**** | < 0.001 | 0.003 |
|  | Model 4 | Ref (1.00) | 0.86 | (0.68,1.10) | 0.92 | (0.71,1.19) | 1.39 | **(1.08,1.78)*** | 0.002 | 0.036 |
| Total-CVD |  |  |  |  |  |  |  |  |  |  |
|  | Model 1 | Ref (1.00) | 1.14 | (0.87,1.48) | 1.39 | **(1.07,1.80)*** | 2.32 | **(1.81,2.99)***** | < 0.001 | < 0.001 |
|  | Model 2 | Ref (1.00) | 1.11 | (0.85,1.45) | 1.34 | **(1.03,1.74)*** | 2.18 | **(1.70,2.81)***** | < 0.001 | < 0.001 |
|  | Model 3 | Ref (1.00) | 1.09 | (0.83,1.43) | 1.31 | (1.00,1.70) | 2.14 | **(1.66,2.75)***** | < 0.001 | < 0.001 |
|  | Model 4 | Ref (1.00) | 1.12 | (0.84,1.50) | 1.31 | (0.99,1.73) | 1.99 | **(1.52,2.61)***** | < 0.001 | < 0.001 |
| Congestive heart failure |  |  |  |  |  |  |  |  |  |  |
|  | Model 1 | Ref (1.00) | 0.89 | (0.59,1.36) | 1.25 | (0.77,2.02) | 2.58 | **(1.60,4.14)***** | < 0.001 | < 0.001 |
|  | Model 2 | Ref (1.00) | 0.87 | (0.57,1.31) | 1.18 | (0.73,1.90) | 2.34 | **(1.46,3.75)**** | < 0.001 | 0.002 |
|  | Model 3 | Ref (1.00) | 0.85 | (0.56,1.29) | 1.16 | (0.72,1.87) | 2.29 | **(1.43,3.67)**** | < 0.001 | 0.002 |
|  | Model 4 | Ref (1.00) | 0.84 | (0.55,1.28) | 1.07 | (0.67,1.71) | 1.89 | **(1.16,3.04)*** | 0.002 | 0.034 |
| Myocardial infarction |  |  |  |  |  |  |  |  |  |  |
|  | Model 1 | Ref (1.00) | 0.96 | (0.71,1.31) | 1.09 | (0.79,1.51) | 1.95 | **(1.41,2.69)***** | < 0.001 | < 0.001 |
|  | Model 2 | Ref (1.00) | 0.94 | (0.69,1.29) | 1.06 | (0.76,1.47) | 1.83 | **(1.31,2.55)**** | < 0.001 | 0.002 |
|  | Model 3 | Ref (1.00) | 0.92 | (0.67,1.27) | 1.02 | (0.72,1.43) | 1.75 | **(1.26,2.43)**** | < 0.001 | 0.003 |
|  | Model 4 | Ref (1.00) | 0.96 | (0.69,1.33) | 1.01 | (0.72,1.42) | 1.64 | **(1.15,2.34)**** | 0.003 | 0.021 |
| Angina pectoris |  |  |  |  |  |  |  |  |  |  |
|  | Model 1 | Ref (1.00) | 0.95 | (0.62,1.47) | 1.68 | **(1.10,2.57)*** | 2.35 | **(1.57,3.54)***** | < 0.001 | < 0.001 |
|  | Model 2 | Ref (1.00) | 0.91 | (0.59,1.41) | 1.60 | **(1.05,2.43)*** | 2.18 | **(1.47,3.25)***** | < 0.001 | 0.001 |
|  | Model 3 | Ref (1.00) | 0.89 | (0.58,1.38) | 1.58 | **(1.04,2.42)*** | 2.15 | **(1.45,3.18)***** | < 0.001 | 0.001 |
|  | Model 4 | Ref (1.00) | 0.93 | (0.60,1.44) | 1.61 | **(1.07,2.44)*** | 2.07 | **(1.37,3.13)**** | < 0.001 | 0.002 |
| Coronary heart disease |  |  |  |  |  |  |  |  |  |  |
|  | Model 1 | Ref (1.00) | 1.01 | (0.67,1.51) | 1.38 | (0.94,2.03) | 2.12 | **(1.51,2.98)***** | < 0.001 | < 0.001 |
|  | Model 2 | Ref (1.00) | 0.97 | (0.65,1.47) | 1.33 | (0.91,1.96) | 2.02 | **(1.44,2.84)***** | < 0.001 | < 0.001 |
|  | Model 3 | Ref (1.00) | 0.96 | (0.64,1.45) | 1.32 | (0.90,1.93) | 1.99 | **(1.42,2.79)***** | < 0.001 | < 0.001 |
|  | Model 4 | Ref (1.00) | 0.99 | (0.65,1.52) | 1.30 | (0.89,1.90) | 1.85 | **(1.30,2.62)**** | < 0.001 | 0.002 |

^a^Model 1 was adjusted for age, sex, ethnicity, and year. Model 2 further adjusted for smoke, drink, exercise, education, income and family history of heart disease. Model 3 further adjusted for energy, AHEI, nutrient supplement use. Model 4 further adjusted for SBP, cholesterol, self-reported cancer, and self-reported diabetes.

^b^Data were listed as the weighted hazard/odd ratio estimates and 95% confidence intervals, with *p < 0.05, **p < 0.01, ***p < 0.001.

^c^Q, quintile.

^d^Ref, reference.

^e^Tests for trends based on the variables containing the median values for each quartile.

^f^*P*_test_ was the result of Bonfreni correction.

Supplementary Table 8. Association of TyG with CVD mortality stratified by age, sex, race, exercise, smoking, drinking, and T2DM status

|  |  |  | TyG | | | | | | | *P*_trend_^e^ | *P*_test_^f^ |
| --- | --- | --- | --- | --- | --- | --- | --- | --- | --- | --- | --- |
|  |  |  | Q1^c^ | Q2 | | Q3 | | Q4 | |  |  |
|  |  |  |  | HR/OR^b^ | 95% CI | HR/OR | 95% CI | HR/OR | 95% CI |  |  |
| Age |  |  |  |  |  |  |  |  |  |  |  |
|  | Model 1^a^ | > 50 | Ref (1.00)^d^ | 1.13 | (0.85,1.49) | 0.98 | (0.72,1.32) | 1.41 | **(1.05,1.89)*** | 0.020 | 0.072 |
|  |  | ≤ 50 | Ref (1.00) | 4.17 | (0.97,17.93) | 13.38 | **(3.52,50.88)***** | 22.77 | **(5.85,88.62)***** | < 0.001 | < 0.001 |
|  | Model 2 | > 50 | Ref (1.00) | 0.96 | (0.72,1.28) | 0.83 | (0.62,1.12) | 1.12 | (0.83,1.52) | 0.298 | 0.667 |
|  |  | ≤ 50 | Ref (1.00) | 3.91 | (0.90,17.02) | 12.09 | **(3.21,45.62)***** | 18.78 | **(4.76,74.16)***** | < 0.001 | < 0.001 |
|  | Model 3 | > 50 | Ref (1.00) | 0.96 | (0.72,1.28) | 0.84 | (0.62,1.13) | 1.11 | (0.82,1.51) | 0.306 | 0.732 |
|  |  | ≤ 50 | Ref (1.00) | 3.90 | (0.89,17.14) | 11.43 | **(3.05,42.81)***** | 17.51 | **(4.46,68.67)***** | < 0.001 | < 0.001 |
|  | Model 4 | > 50 | Ref (1.00) | 0.95 | (0.71,1.27) | 0.86 | (0.63,1.18) | 1.02 | (0.74,1.39) | 0.786 | 1.000 |
|  |  | ≤ 50 | Ref (1.00) | 4.04 | (0.85,19.14) | 12.05 | **(2.89,50.16)***** | 19.58 | **(4.27,89.78)***** | < 0.001 | 0.001 |
| Sex |  |  |  |  |  |  |  |  |  |  |  |
|  | Model l | Male | Ref (1.00) | 1.04 | (0.68,1.60) | 1.08 | (0.70,1.68) | 1.39 | (0.90,2.14) | 0.065 | 0.401 |
|  |  | Female | Ref (1.00) | 0.87 | (0.57,1.33) | 0.76 | (0.49,1.19) | 1.51 | (0.97,2.34) | 0.019 | 0.206 |
|  | Model 2 | Male | Ref (1.00) | 0.96 | (0.63,1.49) | 1.01 | (0.65,1.57) | 1.23 | (0.79,1.91) | 0.192 | 1.000 |
|  |  | Female | Ref (1.00) | 0.78 | (0.51,1.19) | 0.68 | (0.43,1.07) | 1.28 | (0.83,1.98) | 0.062 | 0.274 |
|  | Model 3 | Male | Ref (1.00) | 0.95 | (0.61,1.45) | 0.97 | (0.62,1.51) | 1.18 | (0.76,1.82) | 0.260 | 1.000 |
|  |  | Female | Ref (1.00) | 0.76 | (0.50,1.16) | 0.67 | (0.42,1.05) | 1.25 | (0.80,1.93) | 0.073 | 0.233 |
|  | Model 4 | Male | Ref (1.00) | 0.89 | (0.58,1.37) | 0.92 | (0.58,1.46) | 0.96 | (0.60,1.53) | 0.933 | 1.000 |
|  |  | Female | Ref (1.00) | 0.82 | (0.54,1.24) | 0.72 | (0.46,1.13) | 1.38 | (0.88,2.17) | 0.051 | 0.468 |
| Race |  |  |  |  |  |  |  |  |  |  |  |
|  | Model l | Non-hispanic white | Ref (1.00) | 0.97 | (0.68,1.38) | 0.94 | (0.66,1.34) | 1.32 | (0.93,1.88) | 0.051 | 0.357 |
|  |  | Others | Ref (1.00) | 1.02 | (0.62,1.71) | 0.95 | (0.54,1.67) | 2.07 | **(1.18,3.62)*** | 0.002 | 0.035 |
|  | Model 2 | Non-hispanic white | Ref (1.00) | 0.88 | (0.62,1.24) | 0.83 | (0.57,1.19) | 1.10 | (0.77,1.58) | 0.294 | 0.908 |
|  |  | Others | Ref (1.00) | 1.00 | (0.60,1.66) | 0.91 | (0.51,1.64) | 2.03 | **(1.15,3.57)*** | 0.002 | 0.045 |
|  | Model 3 | Non-hispanic white | Ref (1.00) | 0.85 | (0.60,1.21) | 0.79 | (0.54,1.14) | 1.04 | (0.73,1.50) | 0.435 | 0.617 |
|  |  | Others | Ref (1.00) | 1.01 | (0.61,1.69) | 0.96 | (0.54,1.70) | 2.04 | **(1.16,3.60)*** | 0.002 | 0.041 |
|  | Model 4 | Non-hispanic white | Ref (1.00) | 0.85 | (0.59,1.24) | 0.81 | (0.54,1.20) | 0.98 | (0.67,1.42) | 0.774 | 0.840 |
|  |  | Others | Ref (1.00) | 1.01 | (0.59,1.71) | 0.96 | (0.51,1.80) | 2.00 | **(1.03,3.88)*** | 0.012 | 0.126 |
| Exercise |  |  |  |  |  |  |  |  |  |  |  |
|  | Model l | Yes | Ref (1.00) | 0.92 | (0.60,1.43) | 0.86 | (0.55,1.34) | 1.25 | (0.81,1.93) | 0.088 | 0.907 |
|  |  | No | Ref (1.00) | 0.82 | (0.53,1.28) | 0.87 | (0.57,1.32) | 1.30 | (0.85,1.99) | 0.125 | 0.684 |
|  | Model 2 | Yes | Ref (1.00) | 0.90 | (0.59,1.38) | 0.85 | (0.54,1.32) | 1.22 | (0.80,1.86) | 0.102 | 1.000 |
|  |  | No | Ref (1.00) | 0.84 | (0.54,1.30) | 0.88 | (0.57,1.34) | 1.26 | (0.83,1.91) | 0.167 | 0.827 |
|  | Model 3 | Yes | Ref (1.00) | 0.89 | (0.58,1.35) | 0.83 | (0.53,1.29) | 1.19 | (0.78,1.80) | 0.126 | 1.000 |
|  |  | No | Ref (1.00) | 0.80 | (0.50,1.28) | 0.83 | (0.53,1.32) | 1.17 | (0.76,1.81) | 0.255 | 1.000 |
|  | Model 4 | Yes | Ref (1.00) | 0.90 | (0.58,1.38) | 0.86 | (0.54,1.37) | 1.07 | (0.70,1.65) | 0.413 | 1.000 |
|  |  | No | Ref (1.00) | 0.83 | (0.51,1.37) | 0.88 | (0.53,1.47) | 1.30 | (0.78,2.17) | 0.150 | 0.915 |
| Smoking |  |  |  |  |  |  |  |  |  |  |  |
|  | Model l | Yes | Ref (1.00) | 0.92 | (0.33,2.55) | 1.10 | (0.47,2.59) | 1.60 | (0.68,3.77) | 0.090 | 0.843 |
|  |  | No | Ref (1.00) | 1.02 | (0.74,1.40) | 0.96 | (0.69,1.34) | 1.53 | **(1.10,2.12)*** | 0.002 | 0.036 |
|  | Model 2 | Yes | Ref (1.00) | 0.84 | (0.29,2.44) | 1.11 | (0.46,2.68) | 1.62 | (0.68,3.86) | 0.055 | 0.811 |
|  |  | No | Ref (1.00) | 0.94 | (0.68,1.29) | 0.89 | (0.63,1.24) | 1.32 | (0.96,1.82) | 0.018 | 0.251 |
|  | Model 3 | Yes | Ref (1.00) | 0.92 | (0.30,2.78) | 1.20 | (0.47,3.07) | 1.62 | (0.64,4.11) | 0.092 | 0.926 |
|  |  | No | Ref (1.00) | 0.91 | (0.66,1.26) | 0.86 | (0.61,1.20) | 1.28 | (0.93,1.76) | 0.027 | 0.410 |
|  | Model 4 | Yes | Ref (1.00) | 0.98 | (0.33,2.95) | 1.29 | (0.49,3.37) | 1.56 | (0.58,4.20) | 0.166 | 1.000 |
|  |  | No | Ref (1.00) | 0.92 | (0.66,1.28) | 0.88 | (0.61,1.27) | 1.25 | (0.88,1.78) | 0.065 | 0.607 |
| Drinking |  |  |  |  |  |  |  |  |  |  |  |
|  | Model l | Yes | Ref (1.00) | 1.00 | (0.70,1.43) | 1.16 | (0.81,1.67) | 1.62 | **(1.13,2.32)**** | 0.002 | 0.026 |
|  |  | No | Ref (1.00) | 0.99 | (0.60,1.64) | 0.69 | (0.42,1.14) | 1.37 | (0.84,2.26) | 0.116 | 0.430 |
|  | Model 2 | Yes | Ref (1.00) | 0.93 | (0.64,1.34) | 1.05 | (0.72,1.53) | 1.41 | (0.97,2.03) | 0.014 | 0.207 |
|  |  | No | Ref (1.00) | 0.90 | (0.55,1.48) | 0.64 | (0.39,1.04) | 1.18 | (0.71,1.96) | 0.272 | 0.215 |
|  | Model 3 | Yes | Ref (1.00) | 0.91 | (0.63,1.32) | 1.02 | (0.69,1.49) | 1.36 | (0.94,1.97) | 0.022 | 0.308 |
|  |  | No | Ref (1.00) | 0.88 | (0.53,1.47) | 0.64 | (0.39,1.04) | 1.14 | (0.68,1.90) | 0.324 | 0.121 |
|  | Model 4 | Yes | Ref (1.00) | 0.93 | (0.63,1.38) | 1.06 | (0.70,1.61) | 1.40 | (0.91,2.14) | 0.029 | 0.364 |
|  |  | No | Ref (1.00) | 0.89 | (0.54,1.46) | 0.63 | (0.38,1.02) | 0.99 | (0.60,1.63) | 0.807 | 0.184 |
| T2DM |  |  |  |  |  |  |  |  |  |  |  |
|  | Model l | Yes | Ref (1.00) | 0.54 | (0.21,1.35) | 0.39 | **(0.16,0.97)*** | 0.60 | (0.25,1.45) | 0.972 | 0.128 |
|  |  | No | Ref (1.00) | 1.04 | (0.76,1.42) | 0.99 | (0.71,1.39) | 1.22 | (0.86,1.74) | 0.293 | 0.783 |
|  | Model 2 | Yes | Ref (1.00) | 0.56 | (0.22,1.38) | 0.42 | (0.17,1.03) | 0.61 | (0.26,1.45) | 0.882 | 0.171 |
|  |  | No | Ref (1.00) | 0.95 | (0.69,1.31) | 0.90 | (0.65,1.25) | 1.08 | (0.76,1.54) | 0.618 | 1.000 |
|  | Model 3 | Yes | Ref (1.00) | 0.51 | (0.21,1.25) | 0.40 | **(0.16,0.98)*** | 0.57 | (0.24,1.35) | 0.847 | 0.137 |
|  |  | No | Ref (1.00) | 0.94 | (0.68,1.30) | 0.88 | (0.63,1.23) | 1.05 | (0.73,1.51) | 0.743 | 1.000 |
|  | Model 4 | Yes | Ref (1.00) | 0.51 | (0.21,1.26) | 0.40 | (0.16,1.02) | 0.57 | (0.24,1.37) | 0.817 | 0.166 |
|  |  | No | Ref (1.00) | 0.99 | (0.71,1.38) | 0.96 | (0.69,1.34) | 1.17 | (0.82,1.67) | 0.368 | 1.000 |

^a^The variables adjusted in each model were the factors mentioned above except the stratification variables.

^b^Data were listed as the weighted hazard ratio estimates and 95% confidence intervals, with *p < 0.05, **p < 0.01, ***p < 0.001.

^c^Q, quintile.

^d^Ref, reference.

^e^Tests for trends based on the variables containing the median values for each quartile.

^f^*P*_test_ was the result of Bonfreni correction.

Supplementary Table 9. Association of TyG-WC with CVD mortality stratified by age, sex, race, exercise, smoking, drinking, and T2DM status

|  |  |  | TyG-WC | | | | | | | *P*_trend_^e^ | *P*_test_^f^ |
| --- | --- | --- | --- | --- | --- | --- | --- | --- | --- | --- | --- |
|  |  |  | Q1^c^ | Q2 | | Q3 | | Q4 | |  |  |
|  |  |  |  | HR/OR^b^ | 95% CI | HR/OR | 95% CI | HR/OR | 95% CI |  |  |
| Age |  |  |  |  |  |  |  |  |  |  |  |
|  | Model 1^a^ | > 50 | Ref (1.00)^d^ | 1.20 | (0.85,1.70) | 1.14 | (0.81,1.60) | 1.63 | **(1.16,2.29)**** | 0.002 | 0.017 |
|  |  | ≤ 50 | Ref (1.00) | 5.85 | **(1.23,27.74)*** | 4.97 | **(1.04,23.69)*** | 18.66 | **(4.30,80.96)***** | < 0.001 | < 0.001 |
|  | Model 2 | > 50 | Ref (1.00) | 1.11 | (0.79,1.57) | 1.01 | (0.72,1.41) | 1.30 | (0.93,1.82) | 0.077 | 0.361 |
|  |  | ≤ 50 | Ref (1.00) | 5.55 | **(1.20,25.60)*** | 4.79 | **(1.01,22.70)*** | 15.34 | **(3.62,65.02)***** | < 0.001 | 0.001 |
|  | Model 3 | > 50 | Ref (1.00) | 1.12 | (0.80,1.56) | 1.02 | (0.73,1.42) | 1.37 | (0.99,1.90) | 0.027 | 0.175 |
|  |  | ≤ 50 | Ref (1.00) | 5.59 | **(1.16,26.97)*** | 4.82 | (0.98,23.82) | 15.25 | **(3.59,64.77)***** | < 0.001 | 0.001 |
|  | Model 4 | > 50 | Ref (1.00) | 1.05 | (0.77,1.43) | 0.88 | (0.64,1.22) | 1.11 | (0.80,1.56) | 0.474 | 1.000 |
|  |  | ≤ 50 | Ref (1.00) | 5.30 | **(1.19,23.70)*** | 4.57 | (0.88,23.89) | 14.65 | **(3.16,67.94)***** | < 0.001 | 0.002 |
| Sex |  |  |  |  |  |  |  |  |  |  |  |
|  | Model l | Male | Ref (1.00) | 0.92 | (0.54,1.58) | 0.85 | (0.53,1.34) | 1.61 | (0.98,2.64) | 0.001 | 0.187 |
|  |  | Female | Ref (1.00) | 1.39 | (0.85,2.29) | 1.33 | (0.85,2.09) | 2.29 | **(1.47,3.56)***** | < 0.001 | 0.001 |
|  | Model 2 | Male | Ref (1.00) | 0.93 | (0.55,1.59) | 0.83 | (0.53,1.29) | 1.50 | (0.91,2.47) | 0.004 | 0.322 |
|  |  | Female | Ref (1.00) | 1.40 | (0.87,2.24) | 1.24 | (0.79,1.96) | 1.93 | **(1.24,2.99)**** | 0.003 | 0.011 |
|  | Model 3 | Male | Ref (1.00) | 0.93 | (0.54,1.60) | 0.82 | (0.53,1.28) | 1.47 | (0.91,2.39) | 0.004 | 0.351 |
|  |  | Female | Ref (1.00) | 1.33 | (0.83,2.14) | 1.15 | (0.74,1.80) | 1.83 | **(1.19,2.82)**** | 0.005 | 0.019 |
|  | Model 4 | Male | Ref (1.00) | 0.89 | (0.52,1.54) | 0.74 | (0.47,1.18) | 1.21 | (0.73,2.01) | 0.077 | 0.613 |
|  |  | Female | Ref (1.00) | 1.33 | (0.84,2.11) | 1.14 | (0.72,1.80) | 1.88 | **(1.19,2.96)**** | 0.008 | 0.021 |
| Race |  |  |  |  |  |  |  |  |  |  |  |
|  | Model l | Non-hispanic white | Ref (1.00) | 1.13 | (0.82,1.56) | 1.07 | (0.76,1.51) | 1.99 | **(1.41,2.82)***** | < 0.001 | < 0.001 |
|  |  | Others | Ref (1.00) | 1.38 | (0.77,2.49) | 1.21 | (0.63,2.31) | 2.03 | **(1.10,3.77)*** | 0.024 | 0.075 |
|  | Model 2 | Non-hispanic white | Ref (1.00) | 1.10 | (0.80,1.51) | 0.97 | (0.69,1.36) | 1.69 | **(1.20,2.38)**** | < 0.001 | 0.009 |
|  |  | Others | Ref (1.00) | 1.41 | (0.79,2.52) | 1.20 | (0.62,2.30) | 1.87 | (1.00,3.52) | 0.061 | 0.152 |
|  | Model 3 | Non-hispanic white | Ref (1.00) | 1.08 | (0.79,1.50) | 0.93 | (0.67,1.31) | 1.62 | **(1.16,2.26)**** | 0.001 | 0.015 |
|  |  | Others | Ref (1.00) | 1.41 | (0.79,2.54) | 1.19 | (0.62,2.27) | 1.86 | (0.98,3.52) | 0.070 | 0.172 |
|  | Model 4 | Non-hispanic white | Ref (1.00) | 1.07 | (0.79,1.46) | 0.88 | (0.62,1.23) | 1.43 | **(1.02,2.00)*** | 0.015 | 0.116 |
|  |  | Others | Ref (1.00) | 1.38 | (0.77,2.48) | 1.11 | (0.54,2.29) | 1.83 | (0.84,3.99) | 0.142 | 0.375 |
| Exercise |  |  |  |  |  |  |  |  |  |  |  |
|  | Model l | Yes | Ref (1.00) | 1.00 | (0.67,1.49) | 0.99 | (0.67,1.44) | 1.56 | **(1.07,2.26)*** | 0.001 | 0.060 |
|  |  | No | Ref (1.00) | 1.37 | (0.77,2.45) | 1.14 | (0.66,1.97) | 2.12 | **(1.20,3.76)*** | 0.011 | 0.032 |
|  | Model 2 | Yes | Ref (1.00) | 1.00 | (0.67,1.48) | 0.98 | (0.67,1.43) | 1.54 | **(1.07,2.21)*** | 0.001 | 0.063 |
|  |  | No | Ref (1.00) | 1.34 | (0.75,2.39) | 1.05 | (0.60,1.84) | 1.98 | **(1.12,3.50)*** | 0.019 | 0.057 |
|  | Model 3 | Yes | Ref (1.00) | 0.98 | (0.66,1.46) | 0.96 | (0.66,1.41) | 1.52 | **(1.05,2.20)*** | 0.002 | 0.077 |
|  |  | No | Ref (1.00) | 1.30 | (0.72,2.33) | 0.97 | (0.57,1.68) | 1.77 | **(1.02,3.07)*** | 0.043 | 0.125 |
|  | Model 4 | Yes | Ref (1.00) | 0.98 | (0.67,1.44) | 0.89 | (0.60,1.32) | 1.33 | (0.91,1.95) | 0.033 | 0.422 |
|  |  | No | Ref (1.00) | 1.28 | (0.71,2.30) | 0.99 | (0.58,1.69) | 1.82 | **(1.06,3.13)*** | 0.029 | 0.095 |
| Smoking |  |  |  |  |  |  |  |  |  |  |  |
|  | Model l | Yes | Ref (1.00) | 1.16 | (0.38,3.54) | 1.52 | (0.55,4.21) | 2.79 | **(1.06,7.33)*** | 0.002 | 0.112 |
|  |  | No | Ref (1.00) | 1.23 | (0.89,1.71) | 1.13 | (0.81,1.56) | 1.97 | **(1.41,2.76)***** | < 0.001 | < 0.001 |
|  | Model 2 | Yes | Ref (1.00) | 1.16 | (0.38,3.52) | 1.55 | (0.58,4.18) | 2.70 | **(1.03,7.05)*** | 0.002 | 0.128 |
|  |  | No | Ref (1.00) | 1.23 | (0.90,1.69) | 1.04 | (0.75,1.43) | 1.72 | **(1.23,2.38)**** | 0.001 | 0.005 |
|  | Model 3 | Yes | Ref (1.00) | 1.20 | (0.38,3.78) | 1.70 | (0.58,5.03) | 2.80 | **(1.01,7.73)*** | 0.002 | 0.142 |
|  |  | No | Ref (1.00) | 1.21 | (0.87,1.67) | 1.00 | (0.73,1.37) | 1.64 | **(1.19,2.27)**** | 0.001 | 0.009 |
|  | Model 4 | Yes | Ref (1.00) | 1.28 | (0.42,3.90) | 1.64 | (0.53,5.09) | 2.50 | (0.85,7.34) | 0.017 | 0.281 |
|  |  | No | Ref (1.00) | 1.19 | (0.86,1.63) | 0.95 | (0.69,1.31) | 1.55 | **(1.11,2.16)*** | 0.008 | 0.033 |
| Drinking |  |  |  |  |  |  |  |  |  |  |  |
|  | Model l | Yes | Ref (1.00) | 1.36 | (0.94,1.96) | 1.12 | (0.76,1.63) | 2.30 | **(1.55,3.42)***** | < 0.001 | < 0.001 |
|  |  | No | Ref (1.00) | 1.00 | (0.60,1.67) | 1.23 | (0.80,1.90) | 1.55 | (0.95,2.52) | 0.024 | 0.229 |
|  | Model 2 | Yes | Ref (1.00) | 1.32 | (0.91,1.92) | 1.04 | (0.72,1.51) | 1.96 | **(1.32,2.92)***** | 0.001 | 0.003 |
|  |  | No | Ref (1.00) | 1.04 | (0.63,1.71) | 1.15 | (0.74,1.78) | 1.45 | (0.90,2.34) | 0.066 | 0.367 |
|  | Model 3 | Yes | Ref (1.00) | 1.28 | (0.88,1.88) | 1.01 | (0.69,1.47) | 1.88 | **(1.27,2.78)**** | 0.001 | 0.006 |
|  |  | No | Ref (1.00) | 1.05 | (0.64,1.72) | 1.12 | (0.72,1.72) | 1.41 | (0.87,2.30) | 0.106 | 0.491 |
|  | Model 4 | Yes | Ref (1.00) | 1.27 | (0.88,1.86) | 0.98 | (0.68,1.40) | 1.80 | **(1.23,2.63)**** | 0.001 | 0.009 |
|  |  | No | Ref (1.00) | 1.02 | (0.62,1.67) | 1.02 | (0.66,1.56) | 1.22 | (0.75,1.97) | 0.341 | 1.000 |
| T2DM |  |  |  |  |  |  |  |  |  |  |  |
|  | Model l | Yes | Ref (1.00) | 0.53 | (0.18,1.58) | 0.40 | (0.13,1.28) | 0.57 | (0.21,1.57) | 0.867 | 0.362 |
|  |  | No | Ref (1.00) | 1.21 | (0.89,1.64) | 1.08 | (0.79,1.48) | 1.76 | **(1.21,2.56)**** | 0.006 | 0.010 |
|  | Model 2 | Yes | Ref (1.00) | 0.50 | (0.18,1.45) | 0.38 | (0.12,1.15) | 0.50 | (0.18,1.34) | 0.815 | 0.254 |
|  |  | No | Ref (1.00) | 1.21 | (0.89,1.63) | 1.03 | (0.75,1.40) | 1.57 | **(1.09,2.27)*** | 0.034 | 0.052 |
|  | Model 3 | Yes | Ref (1.00) | 0.50 | (0.18,1.41) | 0.37 | (0.12,1.11) | 0.49 | (0.19,1.27) | 0.765 | 0.225 |
|  |  | No | Ref (1.00) | 1.19 | (0.87,1.62) | 1.00 | (0.73,1.37) | 1.54 | **(1.07,2.22)*** | 0.037 | 0.060 |
|  | Model 4 | Yes | Ref (1.00) | 0.50 | (0.18,1.41) | 0.37 | (0.12,1.10) | 0.49 | (0.18,1.29) | 0.774 | 0.218 |
|  |  | No | Ref (1.00) | 1.20 | (0.89,1.62) | 1.01 | (0.74,1.38) | 1.56 | **(1.09,2.23)*** | 0.031 | 0.049 |

^a^The variables adjusted in each model were the factors mentioned above except the stratification variables.

^b^Data were listed as the weighted hazard ratio estimates and 95% confidence intervals, with *p < 0.05, **p < 0.01, ***p < 0.001.

^c^Q, quintile.

^d^Ref, reference.

^e^Tests for trends based on the variables containing the median values for each quartile.

^f^*P*_test_ was the result of Bonfreni correction.

Supplementary Table 10. Association of TyG-WtHR with CVD mortality stratified by age, sex, race, exercise, smoking, drinking, and T2DM status

|  |  |  | TyG-WtHR | | | | | | | *P*_trend_^e^ | *P*_test_^f^ |
| --- | --- | --- | --- | --- | --- | --- | --- | --- | --- | --- | --- |
|  |  |  | Q1^c^ | Q2 | | Q3 | | Q4 | |  |  |
|  |  |  |  | HR/OR^b^ | 95% CI | HR/OR | 95% CI | HR/OR | 95% CI |  |  |
| Age |  |  |  |  |  |  |  |  |  |  |  |
|  | Model 1^a^ | > 50 | Ref (1.00)^d^ | 1.22 | (0.89,1.69) | 1.63 | (1.21,2.20)** | 2.08 | **(1.50,2.89)***** | < 0.001 | < 0.001 |
|  |  | ≤ 50 | Ref (1.00) | 4.41 | **(1.27,15.24)*** | 2.62 | (0.76,9.01) | 18.03 | **(5.89,55.12)***** | < 0.001 | < 0.001 |
|  | Model 2 | > 50 | Ref (1.00) | 1.11 | (0.80,1.53) | 1.41 | (1.03,1.92)* | 1.60 | **(1.15,2.21)**** | 0.001 | 0.017 |
|  |  | ≤ 50 | Ref (1.00) | 4.27 | **(1.24,14.70)*** | 2.54 | (0.74,8.66) | 14.10 | **(4.55,43.65)***** | < 0.001 | < 0.001 |
|  | Model 3 | > 50 | Ref (1.00) | 1.08 | (0.79,1.48) | 1.36 | (1.01,1.84)* | 1.58 | **(1.15,2.17)**** | 0.002 | 0.017 |
|  |  | ≤ 50 | Ref (1.00) | 4.19 | **(1.16,15.09)*** | 2.52 | (0.72,8.77) | 13.53 | **(4.33,42.28)***** | < 0.001 | < 0.001 |
|  | Model 4 | > 50 | Ref (1.00) | 1.04 | (0.76,1.41) | 1.23 | (0.92,1.65) | 1.30 | (0.94,1.80) | 0.075 | 0.329 |
|  |  | ≤ 50 | Ref (1.00) | 4.09 | **(1.22,13.72)*** | 2.50 | (0.68,9.14) | 13.78 | **(3.77,50.36)***** | < 0.001 | < 0.001 |
| Sex |  |  |  |  |  |  |  |  |  |  |  |
|  | Model l | Male | Ref (1.00) | 0.97 | (0.66,1.41) | 1.21 | (0.86,1.70) | 2.13 | **(1.41,3.21)***** | < 0.001 | 0.001 |
|  |  | Female | Ref (1.00) | 1.55 | (0.91,2.66) | 1.63 | (0.97,2.74) | 2.43 | **(1.45,4.06)***** | < 0.001 | 0.003 |
|  | Model 2 | Male | Ref (1.00) | 0.92 | (0.62,1.36) | 1.11 | (0.78,1.58) | 1.86 | **(1.22,2.85)**** | < 0.001 | 0.014 |
|  |  | Female | Ref (1.00) | 1.49 | (0.88,2.53) | 1.49 | (0.89,2.50) | 2.03 | **(1.22,3.39)**** | 0.004 | 0.021 |
|  | Model 3 | Male | Ref (1.00) | 0.90 | (0.60,1.34) | 1.08 | (0.76,1.54) | 1.78 | **(1.16,2.72)**** | < 0.001 | 0.025 |
|  |  | Female | Ref (1.00) | 1.39 | (0.82,2.35) | 1.38 | (0.83,2.29) | 1.87 | **(1.13,3.08)*** | 0.008 | 0.045 |
|  | Model 4 | Male | Ref (1.00) | 0.86 | (0.58,1.26) | 0.98 | (0.68,1.41) | 1.45 | (0.94,2.24) | 0.010 | 0.270 |
|  |  | Female | Ref (1.00) | 1.40 | (0.84,2.34) | 1.42 | (0.86,2.34) | 1.87 | **(1.10,3.15)*** | 0.015 | 0.061 |
| Race |  |  |  |  |  |  |  |  |  |  |  |
|  | Model l | Non-hispanic white | Ref (1.00) | 1.15 | (0.79,1.67) | 1.29 | (0.95,1.75) | 2.23 | **(1.58,3.17)***** | < 0.001 | < 0.001 |
|  |  | Others | Ref (1.00) | 1.30 | (0.71,2.38) | 1.62 | (0.90,2.91) | 1.99 | **(1.02,3.89)*** | 0.027 | 0.135 |
|  | Model 2 | Non-hispanic white | Ref (1.00) | 1.10 | (0.76,1.59) | 1.15 | (0.84,1.58) | 1.89 | **(1.32,2.70)***** | < 0.001 | 0.002 |
|  |  | Others | Ref (1.00) | 1.28 | (0.69,2.38) | 1.53 | (0.85,2.78) | 1.79 | (0.90,3.57) | 0.075 | 0.283 |
|  | Model 3 | Non-hispanic white | Ref (1.00) | 1.06 | (0.73,1.54) | 1.11 | (0.81,1.51) | 1.78 | **(1.25,2.53)**** | < 0.001 | 0.005 |
|  |  | Others | Ref (1.00) | 1.25 | (0.67,2.32) | 1.52 | (0.83,2.76) | 1.73 | (0.88,3.42) | 0.090 | 0.342 |
|  | Model 4 | Non-hispanic white | Ref (1.00) | 1.05 | (0.73,1.49) | 1.05 | (0.77,1.44) | 1.58 | **(1.11,2.25)*** | 0.002 | 0.034 |
|  |  | Others | Ref (1.00) | 1.21 | (0.64,2.28) | 1.46 | (0.77,2.77) | 1.65 | (0.73,3.74) | 0.175 | 0.685 |
| Exercise |  |  |  |  |  |  |  |  |  |  |  |
|  | Model l | Yes | Ref (1.00) | 1.13 | (0.72,1.76) | 1.24 | (0.79,1.97) | 1.85 | **(1.21,2.81)**** | < 0.001 | 0.014 |
|  |  | No | Ref (1.00) | 1.11 | (0.65,1.87) | 1.30 | (0.83,2.04) | 2.06 | **(1.20,3.54)*** | 0.004 | 0.029 |
|  | Model 2 | Yes | Ref (1.00) | 1.13 | (0.72,1.76) | 1.24 | (0.79,1.97) | 1.85 | **(1.21,2.81)**** | < 0.001 | 0.018 |
|  |  | No | Ref (1.00) | 1.06 | (0.63,1.80) | 1.19 | (0.76,1.86) | 1.87 | **(1.09,3.22)*** | 0.011 | 0.073 |
|  | Model 3 | Yes | Ref (1.00) | 1.09 | (0.70,1.68) | 1.19 | (0.76,1.87) | 1.75 | **(1.15,2.66)**** | < 0.001 | 0.029 |
|  |  | No | Ref (1.00) | 0.99 | (0.57,1.71) | 1.10 | (0.70,1.72) | 1.62 | (0.96,2.75) | 0.028 | 0.213 |
|  | Model 4 | Yes | Ref (1.00) | 1.05 | (0.68,1.60) | 1.12 | (0.70,1.77) | 1.50 | (0.98,2.30) | 0.006 | 0.195 |
|  |  | No | Ref (1.00) | 1.02 | (0.59,1.77) | 1.14 | (0.72,1.81) | 1.71 | (0.99,2.95) | 0.018 | 0.159 |
| Smoking |  |  |  |  |  |  |  |  |  |  |  |
|  | Model l | Yes | Ref (1.00) | 1.30 | (0.51,3.29) | 1.33 | (0.56,3.12) | 3.37 | **(1.44,7.90)**** | 0.001 | 0.017 |
|  |  | No | Ref (1.00) | 1.17 | (0.83,1.66) | 1.42 | **(1.02,1.99)*** | 2.07 | **(1.44,2.99)***** | < 0.001 | < 0.001 |
|  | Model 2 | Yes | Ref (1.00) | 1.33 | (0.55,3.23) | 1.30 | (0.54,3.11) | 3.20 | **(1.40,7.30)**** | 0.001 | 0.019 |
|  |  | No | Ref (1.00) | 1.12 | (0.80,1.58) | 1.28 | (0.91,1.79) | 1.76 | **(1.22,2.55)**** | < 0.001 | 0.009 |
|  | Model 3 | Yes | Ref (1.00) | 1.41 | (0.56,3.54) | 1.38 | (0.54,3.53) | 3.19 | **(1.34,7.62)**** | 0.002 | 0.028 |
|  |  | No | Ref (1.00) | 1.07 | (0.76,1.50) | 1.22 | (0.87,1.70) | 1.64 | **(1.15,2.35)**** | 0.001 | 0.021 |
|  | Model 4 | Yes | Ref (1.00) | 1.50 | (0.61,3.68) | 1.36 | (0.50,3.74) | 2.85 | **(1.08,7.52)*** | 0.013 | 0.103 |
|  |  | No | Ref (1.00) | 1.05 | (0.75,1.47) | 1.18 | (0.85,1.64) | 1.53 | **(1.06,2.21)*** | 0.005 | 0.072 |
| Drinking |  |  |  |  |  |  |  |  |  |  |  |
|  | Model l | Yes | Ref (1.00) | 1.16 | (0.79,1.71) | 1.42 | (0.97,2.06) | 2.43 | **(1.65,3.60)***** | < 0.001 | < 0.001 |
|  |  | No | Ref (1.00) | 1.21 | (0.73,2.01) | 1.31 | (0.75,2.30) | 1.79 | **(1.05,3.05)*** | 0.015 | 0.102 |
|  | Model 2 | Yes | Ref (1.00) | 1.13 | (0.76,1.67) | 1.31 | (0.89,1.93) | 2.06 | **(1.37,3.10)***** | < 0.001 | 0.002 |
|  |  | No | Ref (1.00) | 1.16 | (0.70,1.91) | 1.18 | (0.66,2.11) | 1.57 | (0.91,2.70) | 0.058 | 0.305 |
|  | Model 3 | Yes | Ref (1.00) | 1.09 | (0.73,1.63) | 1.27 | (0.86,1.87) | 1.94 | **(1.30,2.91)**** | < 0.001 | 0.005 |
|  |  | No | Ref (1.00) | 1.08 | (0.65,1.80) | 1.09 | (0.62,1.94) | 1.44 | (0.83,2.50) | 0.104 | 0.569 |
|  | Model 4 | Yes | Ref (1.00) | 1.09 | (0.74,1.61) | 1.24 | (0.85,1.83) | 1.88 | **(1.27,2.81)**** | < 0.001 | 0.006 |
|  |  | No | Ref (1.00) | 1.05 | (0.63,1.74) | 1.05 | (0.59,1.85) | 1.20 | (0.71,2.05) | 0.398 | 1.000 |
| T2DM |  |  |  |  |  |  |  |  |  |  |  |
|  | Model l | Yes | Ref (1.00) | 0.55 | (0.12,2.43) | 0.62 | (0.15,2.61) | 0.70 | (0.17,2.78) | 0.690 | 1.000 |
|  |  | No | Ref (1.00) | 1.16 | (0.83,1.63) | 1.27 | (0.93,1.73) | 1.92 | **(1.33,2.77)***** | < 0.001 | 0.002 |
|  | Model 2 | Yes | Ref (1.00) | 0.49 | (0.11,2.19) | 0.58 | (0.14,2.40) | 0.59 | (0.15,2.40) | 0.983 | 1.000 |
|  |  | No | Ref (1.00) | 1.12 | (0.80,1.57) | 1.14 | (0.82,1.59) | 1.66 | **(1.15,2.41)***** | 0.007 | 0.024 |
|  | Model 3 | Yes | Ref (1.00) | 0.47 | (0.11,2.02) | 0.54 | (0.13,2.18) | 0.55 | (0.14,2.17) | 0.944 | 0.911 |
|  |  | No | Ref (1.00) | 1.08 | (0.77,1.51) | 1.11 | (0.81,1.52) | 1.58 | **(1.10,2.27)*** | 0.012 | 0.044 |
|  | Model 4 | Yes | Ref (1.00) | 0.47 | (0.11,2.00) | 0.54 | (0.13,2.23) | 0.55 | (0.14,2.19) | 0.939 | 0.906 |
|  |  | No | Ref (1.00) | 1.11 | (0.80,1.54) | 1.14 | (0.83,1.56) | 1.60 | **(1.12,2.29)*** | 0.009 | 0.034 |

^a^The variables adjusted in each model were the factors mentioned above except the stratification variables.

^b^Data were listed as the weighted hazard ratio estimates and 95% confidence intervals, with *p < 0.05, **p < 0.01, ***p < 0.001.

^c^Q, quintile.

^d^Ref, reference.

^e^Tests for trends based on the variables containing the median values for each quartile.

^f^*P*_test_ was the result of Bonfreni correction.

Supplementary Table 11. Association of TyG-BMI with CVD mortality stratified by age, sex, race, exercise, smoking, drinking, and T2DM status

|  |  |  | TyG-BMI | | | | | | | *P*_trend_^e^ | *P*_test_^f^ |
| --- | --- | --- | --- | --- | --- | --- | --- | --- | --- | --- | --- |
|  |  |  | Q1^c^ | Q2 | | Q3 | | Q4 | |  |  |
|  |  |  |  | HR/OR^b^ | 95% CI | HR/OR | 95% CI | HR/OR | 95% CI |  |  |
| Age |  |  |  |  |  |  |  |  |  |  |  |
|  | Model 1^a^ | > 50 | Ref (1.00)^d^ | 0.79 | (0.60,1.03) | 0.88 | (0.66,1.16) | 1.04 | (0.79,1.36) | 0.326 | 0.250 |
|  |  | ≤ 50 | Ref (1.00) | 3.91 | **(1.01,15.21)*** | 3.99 | **(1.02,15.62)*** | 10.96 | **(3.18,37.80)***** | < 0.001 | 0.001 |
|  | Model 2 | > 50 | Ref (1.00) | 0.73 | **(0.56,0.96)*** | 0.82 | (0.61,1.09) | 0.87 | (0.68,1.13) | 0.853 | 0.070 |
|  |  | ≤ 50 | Ref (1.00) | 4.08 | **(1.11,15.03)*** | 3.95 | (0.99,15.72) | 9.61 | **(2.86,32.32)***** | < 0.001 | 0.001 |
|  | Model 3 | > 50 | Ref (1.00) | 0.73 | **(0.56,0.95)*** | 0.82 | (0.62,1.08) | 0.91 | (0.71,1.18) | 0.839 | 0.062 |
|  |  | ≤ 50 | Ref (1.00) | 4.11 | **(1.04,16.23)*** | 4.09 | (0.98,17.01) | 9.87 | **(2.86,34.09)***** | < 0.001 | 0.001 |
|  | Model 4 | > 50 | Ref (1.00) | 0.74 | **(0.57,0.95)*** | 0.76 | **(0.58,0.99)*** | 0.80 | (0.62,1.03) | 0.323 | 0.055 |
|  |  | ≤ 50 | Ref (1.00) | 3.86 | (1.10,13.52) | 3.90 | (0.88,17.28) | 9.39 | **(2.44,36.15)***** | 0.001 | 0.004 |
| Sex |  |  |  |  |  |  |  |  |  |  |  |
|  | Model l | Male | Ref (1.00) | 0.78 | (0.55,1.11) | 0.92 | (0.63,1.35) | 1.75 | **(1.21,2.52)**** | < 0.001 | 0.010 |
|  |  | Female | Ref (1.00) | 1.09 | (0.72,1.64) | 1.21 | (0.80,1.84) | 1.74 | **(1.19,2.54)**** | 0.002 | 0.014 |
|  | Model 2 | Male | Ref (1.00) | 0.77 | (0.53,1.12) | 0.91 | (0.61,1.35) | 1.58 | **(1.08,2.31)*** | 0.002 | 0.059 |
|  |  | Female | Ref (1.00) | 1.03 | (0.68,1.56) | 1.13 | (0.75,1.70) | 1.48 | **(1.03,2.14)*** | 0.016 | 0.110 |
|  | Model 3 | Male | Ref (1.00) | 0.76 | (0.52,1.11) | 0.90 | (0.60,1.33) | 1.55 | **(1.06,2.26)*** | 0.002 | 0.077 |
|  |  | Female | Ref (1.00) | 1.00 | (0.66,1.50) | 1.05 | (0.70,1.57) | 1.42 | (0.98,2.06) | 0.033 | 0.197 |
|  | Model 4 | Male | Ref (1.00) | 0.74 | (0.51,1.09) | 0.81 | (0.54,1.23) | 1.28 | (0.85,1.92) | 0.042 | 0.037 |
|  |  | Female | Ref (1.00) | 1.00 | (0.66,1.53) | 1.03 | (0.68,1.57) | 1.45 | (0.97,2.18) | 0.036 | 0.207 |
| Race |  |  |  |  |  |  |  |  |  |  |  |
|  | Model l | Non-hispanic white | Ref (1.00) | 0.79 | (0.58,1.07) | 1.11 | (0.82,1.49) | 1.77 | **(1.34,2.34)***** | < 0.001 | < 0.001 |
|  |  | Others | Ref (1.00) | 1.36 | (0.81,2.27) | 0.76 | (0.46,1.28) | 1.66 | (0.98,2.79) | 0.152 | 0.176 |
|  | Model 2 | Non-hispanic white | Ref (1.00) | 0.73 | (0.54,1.00) | 1.03 | (0.76,1.39) | 1.50 | **(1.13,1.98)**** | < 0.001 | 0.015 |
|  |  | Others | Ref (1.00) | 1.30 | (0.79,2.15) | 0.74 | (0.44,1.23) | 1.52 | (0.90,2.56) | 0.250 | 0.361 |
|  | Model 3 | Non-hispanic white | Ref (1.00) | 0.72 | (0.53,0.98)* | 0.99 | (0.74,1.34) | 1.46 | **(1.11,1.92)**** | < 0.001 | 0.024 |
|  |  | Others | Ref (1.00) | 1.30 | (0.79,2.13) | 0.74 | (0.44,1.23) | 1.47 | (0.87,2.48) | 0.298 | 0.434 |
|  | Model 4 | Non-hispanic white | Ref (1.00) | 0.72 | (0.53,0.97)* | 0.92 | (0.69,1.23) | 1.29 | (0.97,1.71) | 0.008 | 0.086 |
|  |  | Others | Ref (1.00) | 1.30 | (0.78,2.18) | 0.69 | (0.38,1.24) | 1.46 | (0.75,2.82) | 0.411 | 0.641 |
| Exercise |  |  |  |  |  |  |  |  |  |  |  |
|  | Model l | Yes | Ref (1.00) | 0.92 | (0.66,1.30) | 1.04 | (0.73,1.48) | 1.55 | **(1.14,2.12)**** | 0.001 | 0.018 |
|  |  | No | Ref (1.00) | 0.81 | (0.53,1.24) | 1.00 | (0.67,1.51) | 1.62 | (1.04,2.52) | 0.017 | 0.095 |
|  | Model 2 | Yes | Ref (1.00) | 0.92 | (0.66,1.28) | 1.03 | (0.73,1.46) | 1.53 | **(1.12,2.07)**** | 0.001 | 0.023 |
|  |  | No | Ref (1.00) | 0.77 | (0.50,1.19) | 0.93 | (0.61,1.44) | 1.49 | (0.96,2.31) | 0.035 | 0.216 |
|  | Model 3 | Yes | Ref (1.00) | 0.91 | (0.65,1.26) | 1.03 | (0.73,1.46) | 1.52 | **(1.12,2.08)**** | 0.001 | 0.025 |
|  |  | No | Ref (1.00) | 0.73 | (0.47,1.14) | 0.83 | (0.54,1.29) | 1.35 | (0.89,2.05) | 0.077 | 0.483 |
|  | Model 4 | Yes | Ref (1.00) | 0.90 | (0.65,1.25) | 0.95 | (0.67,1.34) | 1.34 | **(0.97,1.86)**** | 0.017 | 0.223 |
|  |  | No | Ref (1.00) | 0.75 | (0.47,1.18) | 0.83 | (0.52,1.31) | 1.39 | (0.90,2.15) | 0.065 | 0.411 |
| Smoking |  |  |  |  |  |  |  |  |  |  |  |
|  | Model l | Yes | Ref (1.00) | 1.27 | (0.62,2.60) | 1.10 | (0.59,2.08) | 2.66 | **(1.32,5.33)**** | 0.004 | 0.020 |
|  |  | No | Ref (1.00) | 0.87 | (0.65,1.15) | 1.07 | (0.80,1.44) | 1.65 | **(1.21,2.23)***** | < 0.001 | 0.005 |
|  | Model 2 | Yes | Ref (1.00) | 1.40 | (0.71,2.74) | 1.12 | (0.60,2.08) | 2.74 | **(1.39,5.38)**** | 0.003 | 0.012 |
|  |  | No | Ref (1.00) | 0.83 | (0.62,1.10) | 0.99 | (0.74,1.34) | 1.41 | **(1.04,1.91)*** | 0.005 | 0.082 |
|  | Model 3 | Yes | Ref (1.00) | 1.38 | (0.67,2.84) | 1.18 | (0.62,2.27) | 2.74 | **(1.32,5.66)**** | 0.004 | 0.022 |
|  |  | No | Ref (1.00) | 0.81 | (0.61,1.07) | 0.96 | (0.71,1.29) | 1.37 | **(1.01,1.85)*** | 0.008 | 0.137 |
|  | Model 4 | Yes | Ref (1.00) | 1.33 | (0.63,2.82) | 1.11 | (0.56,2.18) | 2.32 | **(1.02,5.28)*** | 0.029 | 0.137 |
|  |  | No | Ref (1.00) | 0.80 | (0.61,1.07) | 0.91 | (0.67,1.23) | 1.30 | (0.95,1.77) | 0.024 | 0.293 |
| Drinking |  |  |  |  |  |  |  |  |  |  |  |
|  | Model l | Yes | Ref (1.00) | 0.85 | (0.63,1.16) | 1.04 | (0.77,1.40) | 1.82 | **(1.32,2.52)***** | < 0.001 | 0.001 |
|  |  | No | Ref (1.00) | 1.00 | (0.66,1.50) | 1.05 | (0.66,1.67) | 1.57 | **(1.03,2.40)*** | 0.018 | 0.215 |
|  | Model 2 | Yes | Ref (1.00) | 0.84 | (0.61,1.16) | 1.01 | (0.74,1.37) | 1.57 | **(1.13,2.20)**** | 0.001 | 0.025 |
|  |  | No | Ref (1.00) | 0.97 | (0.64,1.46) | 0.98 | (0.61,1.58) | 1.45 | (0.96,2.21) | 0.042 | 0.241 |
|  | Model 3 | Yes | Ref (1.00) | 0.82 | (0.60,1.13) | 0.98 | (0.73,1.31) | 1.54 | **(1.10,2.14)*** | 0.002 | 0.035 |
|  |  | No | Ref (1.00) | 0.95 | (0.64,1.42) | 0.94 | (0.58,1.52) | 1.38 | (0.89,2.12) | 0.09 | 0.440 |
|  | Model 4 | Yes | Ref (1.00) | 0.82 | (0.60,1.13) | 0.95 | (0.71,1.26) | 1.48 | **(1.06,2.06)*** | 0.003 | 0.061 |
|  |  | No | Ref (1.00) | 0.92 | (0.61,1.38) | 0.83 | (0.52,1.33) | 1.20 | (0.80,1.80) | 0.277 | 1.000 |
| T2DM |  |  |  |  |  |  |  |  |  |  |  |
|  | Model l | Yes | Ref (1.00) | 0.64 | (0.31,1.35) | 0.67 | (0.35,1.26) | 0.75 | (0.38,1.46) | 1.000 | 0.629 |
|  |  | No | Ref (1.00) | 0.89 | (0.67,1.19) | 0.99 | (0.72,1.37) | 1.59 | **(1.14,2.21)**** | 0.007 | 0.019 |
|  | Model 2 | Yes | Ref (1.00) | 0.64 | (0.30,1.38) | 0.64 | (0.32,1.28) | 0.68 | (0.33,1.38) | 0.666 | 0.622 |
|  |  | No | Ref (1.00) | 0.86 | (0.65,1.14) | 0.95 | (0.69,1.31) | 1.41 | **(1.01,1.98)*** | 0.037 | 0.137 |
|  | Model 3 | Yes | Ref (1.00) | 0.64 | (0.30,1.37) | 0.62 | (0.31,1.24) | 0.66 | (0.32,1.35) | 0.623 | 0.515 |
|  |  | No | Ref (1.00) | 0.85 | (0.65,1.12) | 0.93 | (0.68,1.29) | 1.39 | (1.00,1.94) | 0.041 | 0.154 |
|  | Model 4 | Yes | Ref (1.00) | 0.64 | (0.30,1.38) | 0.61 | (0.30,1.23) | 0.66 | (0.32,1.36) | 0.630 | 0.495 |
|  |  | No | Ref (1.00) | 0.87 | (0.66,1.14) | 0.93 | (0.69,1.27) | 1.41 | **(1.02,1.94)*** | 0.035 | 0.115 |

^a^The variables adjusted in each model were the factors mentioned above except the stratification variables.

^b^Data were listed as the weighted hazard ratio estimates and 95% confidence intervals, with *p < 0.05, **p < 0.01, ***p < 0.001.

^c^Q, quintile.

^d^Ref, reference.

^e^Tests for trends based on the variables containing the median values for each quartile.

^f^*P*_test_ was the result of Bonfreni correction.

Supplementary Table 12. Association of TyG with Total-CVD stratified by age, sex, race, exercise, smoking, drinking, and T2DM status

|  |  |  | TyG | | | | | | | *P*_trend_^e^ | *P*_test_^f^ |
| --- | --- | --- | --- | --- | --- | --- | --- | --- | --- | --- | --- |
|  |  |  | Q1^c^ | Q2 | | Q3 | | Q4 | |  |  |
|  |  |  |  | HR/OR^b^ | 95% CI | HR/OR | 95% CI | HR/OR | 95% CI |  |  |
| Age |  |  |  |  |  |  |  |  |  |  |  |
|  | Model 1^a^ | > 50 | Ref (1.00)^d^ | 1.13 | (0.84,1.51) | 1.41 | **(1.10,1.79)**** | 1.85 | **(1.46,2.35)***** | < 0.001 | < 0.001 |
|  |  | ≤ 50 | Ref (1.00) | 2.00 | **(1.20,3.35)**** | 2.29 | **(1.27,4.15)**** | 3.89 | **(2.28,6.61)***** | < 0.001 | < 0.001 |
|  | Model 2 | > 50 | Ref (1.00) | 1.06 | (0.79,1.42) | 1.31 | **(1.03,1.67)*** | 1.68 | **(1.31,2.16)***** | < 0.001 | < 0.001 |
|  |  | ≤ 50 | Ref (1.00) | 1.81 | **(1.08,3.04)*** | 2.05 | **(1.13,3.74)*** | 3.23 | **(1.92,5.46)***** | < 0.001 | < 0.001 |
|  | Model 3 | > 50 | Ref (1.00) | 1.08 | (0.80,1.44) | 1.33 | **(1.04,1.70)*** | 1.70 | **(1.32,2.18)***** | < 0.001 | < 0.001 |
|  |  | ≤ 50 | Ref (1.00) | 1.77 | **(1.06,2.97)*** | 1.97 | **(1.09,3.57)*** | 3.17 | **(1.87,5.37)***** | < 0.001 | < 0.001 |
|  | Model 4 | > 50 | Ref (1.00) | 1.15 | (0.84,1.56) | 1.49 | **(1.16,1.91)**** | 1.94 | **(1.47,2.55)***** | < 0.001 | < 0.001 |
|  |  | ≤ 50 | Ref (1.00) | 1.82 | **(1.08,3.09)*** | 2.10 | **(1.12,3.92)*** | 2.88 | **(1.58,5.27)***** | 0.001 | 0.002 |
| Sex |  |  |  |  |  |  |  |  |  |  |  |
|  | Model l | Male | Ref (1.00) | 1.01 | (0.70,1.46) | 1.25 | (0.86,1.81) | 1.90 | **(1.34,2.72)***** | < 0.001 | 0.002 |
|  |  | Female | Ref (1.00) | 1.38 | (0.96,1.96) | 1.71 | **(1.27,2.31)***** | 2.18 | **(1.61,2.95)***** | < 0.001 | < 0.001 |
|  | Model 2 | Male | Ref (1.00) | 1.00 | (0.68,1.45) | 1.23 | (0.85,1.78) | 1.87 | **(1.30,2.68)***** | < 0.001 | 0.003 |
|  |  | Female | Ref (1.00) | 1.26 | (0.89,1.79) | 1.53 | **(1.12,2.08)**** | 1.84 | **(1.34,2.52)***** | < 0.001 | 0.001 |
|  | Model 3 | Male | Ref (1.00) | 0.99 | (0.68,1.43) | 1.20 | (0.83,1.74) | 1.81 | **(1.27,2.58)***** | < 0.001 | 0.004 |
|  |  | Female | Ref (1.00) | 1.26 | (0.88,1.79) | 1.50 | **(1.11,2.05)**** | 1.83 | **(1.34,2.50)***** | < 0.001 | 0.001 |
|  | Model 4 | Male | Ref (1.00) | 1.03 | (0.71,1.50) | 1.29 | (0.89,1.88) | 2.15 | **(1.47,3.15)***** | < 0.001 | < 0.001 |
|  |  | Female | Ref (1.00) | 1.41 | (0.97,2.03) | 1.72 | **(1.26,2.37)***** | 1.97 | **(1.43,2.71)***** | < 0.001 | < 0.001 |
| Race |  |  |  |  |  |  |  |  |  |  |  |
|  | Model l | Non-hispanic white | Ref (1.00) | 1.16 | (0.84,1.59) | 1.45 | **(1.06,1.99)*** | 1.99 | **(1.55,2.56)***** | < 0.001 | < 0.001 |
|  |  | Others | Ref (1.00) | 1.06 | (0.75,1.51) | 1.17 | (0.81,1.68) | 1.59 | **(1.09,2.33)*** | 0.007 | 0.051 |
|  | Model 2 | Non-hispanic white | Ref (1.00) | 1.10 | (0.80,1.52) | 1.36 | (0.99,1.87) | 1.82 | **(1.40,2.37)***** | < 0.001 | < 0.001 |
|  |  | Others | Ref (1.00) | 1.03 | (0.73,1.47) | 1.14 | (0.80,1.63) | 1.53 | **(1.06,2.22)*** | 0.011 | 0.074 |
|  | Model 3 | Non-hispanic white | Ref (1.00) | 1.10 | (0.79,1.52) | 1.33 | (0.97,1.83) | 1.78 | **(1.37,2.31)***** | < 0.001 | < 0.001 |
|  |  | Others | Ref (1.00) | 1.05 | (0.74,1.49) | 1.17 | (0.82,1.68) | 1.55 | **(1.07,2.25)*** | 0.009 | 0.064 |
|  | Model 4 | Non-hispanic white | Ref (1.00) | 1.22 | (0.87,1.70) | 1.53 | **(1.11,2.12)**** | 2.16 | **(1.63,2.85)***** | < 0.001 | < 0.001 |
|  |  | Others | Ref (1.00) | 1.13 | (0.79,1.62) | 1.30 | (0.89,1.89) | 1.67 | **(1.14,2.45)**** | 0.004 | 0.027 |
| Exercise |  |  |  |  |  |  |  |  |  |  |  |
|  | Model l | Yes | Ref (1.00) | 1.05 | (0.77,1.42) | 1.33 | (0.98,1.80) | 1.89 | **(1.38,2.60)***** | < 0.001 | < 0.001 |
|  |  | No | Ref (1.00) | 1.23 | (0.84,1.79) | 1.43 | (0.99,2.06) | 1.72 | **(1.25,2.36)***** | 0.001 | 0.003 |
|  | Model 2 | Yes | Ref (1.00) | 1.04 | (0.76,1.41) | 1.31 | (0.97,1.78) | 1.88 | **(1.38,2.58)***** | < 0.001 | < 0.001 |
|  |  | No | Ref (1.00) | 1.20 | (0.82,1.75) | 1.38 | (0.95,1.99) | 1.61 | **(1.15,2.23)**** | 0.003 | 0.016 |
|  | Model 3 | Yes | Ref (1.00) | 1.05 | (0.77,1.42) | 1.31 | (0.97,1.78) | 1.91 | **(1.39,2.62)***** | < 0.001 | < 0.001 |
|  |  | No | Ref (1.00) | 1.22 | (0.84,1.78) | 1.38 | (0.95,2.00) | 1.60 | **(1.15,2.23)**** | 0.004 | 0.017 |
|  | Model 4 | Yes | Ref (1.00) | 1.11 | (0.81,1.51) | 1.44 | **(1.06,1.95)*** | 2.01 | **(1.43,2.82)***** | < 0.001 | < 0.001 |
|  |  | No | Ref (1.00) | 1.39 | (0.94,2.05) | 1.66 | **(1.11,2.48)*** | 2.17 | **(1.51,3.12)***** | < 0.001 | < 0.001 |
| Smoking |  |  |  |  |  |  |  |  |  |  |  |
|  | Model l | Yes | Ref (1.00) | 0.77 | (0.44,1.35) | 1.03 | (0.64,1.64) | 1.24 | (0.77,2.00) | 0.196 | 1.000 |
|  |  | No | Ref (1.00) | 1.24 | (0.95,1.63) | 1.46 | **(1.09,1.95)*** | 2.11 | **(1.68,2.64)***** | < 0.001 | < 0.001 |
|  | Model 2 | Yes | Ref (1.00) | 0.72 | (0.41,1.28) | 0.98 | (0.62,1.55) | 1.22 | (0.75,1.98) | 0.201 | 0.775 |
|  |  | No | Ref (1.00) | 1.21 | (0.92,1.60) | 1.42 | **(1.05,1.91)*** | 2.00 | **(1.58,2.55)***** | < 0.001 | < 0.001 |
|  | Model 3 | Yes | Ref (1.00) | 0.73 | (0.41,1.30) | 0.99 | (0.62,1.56) | 1.23 | (0.76,1.99) | 0.201 | 0.851 |
|  |  | No | Ref (1.00) | 1.21 | (0.91,1.59) | 1.39 | **(1.03,1.86)*** | 1.97 | **(1.55,2.49)***** | < 0.001 | < 0.001 |
|  | Model 4 | Yes | Ref (1.00) | 0.85 | (0.48,1.51) | 1.20 | (0.77,1.88) | 1.65 | **(1.03,2.65)*** | 0.022 | 0.113 |
|  |  | No | Ref (1.00) | 1.29 | (0.99,1.70) | 1.53 | **(1.14,2.06)**** | 2.18 | **(1.68,2.83)***** | < 0.001 | < 0.001 |
| Drinking |  |  |  |  |  |  |  |  |  |  |  |
|  | Model l | Yes | Ref (1.00) | 1.12 | (0.83,1.51) | 1.38 | **(1.01,1.89)*** | 1.91 | **(1.48,2.45)***** | < 0.001 | < 0.001 |
|  |  | No | Ref (1.00) | 1.20 | (0.77,1.86) | 1.45 | (0.95,2.20) | 2.08 | **(1.39,3.11)***** | < 0.001 | 0.002 |
|  | Model 2 | Yes | Ref (1.00) | 1.05 | (0.78,1.42) | 1.27 | (0.93,1.74) | 1.72 | **(1.33,2.22)***** | < 0.001 | < 0.001 |
|  |  | No | Ref (1.00) | 1.17 | (0.76,1.81) | 1.35 | (0.89,2.04) | 1.93 | **(1.28,2.91)**** | 0.001 | 0.006 |
|  | Model 3 | Yes | Ref (1.00) | 1.06 | (0.79,1.43) | 1.27 | (0.93,1.74) | 1.72 | **(1.33,2.22)***** | < 0.001 | < 0.001 |
|  |  | No | Ref (1.00) | 1.16 | (0.75,1.79) | 1.31 | (0.86,2.00) | 1.85 | **(1.24,2.76)**** | 0.001 | 0.009 |
|  | Model 4 | Yes | Ref (1.00) | 1.17 | (0.86,1.59) | 1.46 | **(1.07,1.99)*** | 2.11 | **(1.61,2.77)***** | < 0.001 | < 0.001 |
|  |  | No | Ref (1.00) | 1.26 | (0.80,1.97) | 1.43 | (0.92,2.24) | 1.93 | **(1.27,2.92)**** | 0.001 | 0.007 |
| T2DM |  |  |  |  |  |  |  |  |  |  |  |
|  | Model l | Yes | Ref (1.00) | 0.80 | (0.33,1.94) | 0.99 | (0.43,2.29) | 1.30 | (0.57,2.95) | 0.086 | 1.000 |
|  |  | No | Ref (1.00) | 1.14 | (0.88,1.48) | 1.32 | **(1.01,1.72)*** | 1.46 | **(1.17,1.82)***** | 0.001 | 0.003 |
|  | Model 2 | Yes | Ref (1.00) | 0.80 | (0.33,1.94) | 0.97 | (0.43,2.21) | 1.27 | (0.58,2.79) | 0.090 | 1.000 |
|  |  | No | Ref (1.00) | 1.10 | (0.85,1.43) | 1.26 | (0.97,1.65) | 1.38 | **(1.10,1.75)**** | 0.004 | 0.021 |
|  | Model 3 | Yes | Ref (1.00) | 0.76 | (0.32,1.79) | 0.97 | (0.42,2.22) | 1.27 | (0.59,2.76) | 0.078 | 1.000 |
|  |  | No | Ref (1.00) | 1.10 | (0.85,1.44) | 1.25 | (0.96,1.63) | 1.37 | **(1.09,1.73)**** | 0.005 | 0.024 |
|  | Model 4 | Yes | Ref (1.00) | 0.80 | (0.35,1.85) | 1.02 | (0.47,2.25) | 1.44 | (0.69,3.04) | 0.025 | 0.995 |
|  |  | No | Ref (1.00) | 1.28 | (0.97,1.69) | 1.57 | **(1.20,2.06)***** | 2.05 | **(1.61,2.61)***** | < 0.001 | < 0.001 |

^a^The variables adjusted in each model were the factors mentioned above except the stratification variables.

^b^Data were listed as the weighted odd ratio estimates and 95% confidence intervals, with *p < 0.05, **p < 0.01, ***p < 0.001.

^c^Q, quintile.

^d^Ref, reference.

^e^Tests for trends based on the variables containing the median values for each quartile.

^f^*P*_test_ was the result of Bonfreni correction.

Supplementary Table 13. Association of TyG-WC with Total-CVD stratified by age, sex, race, exercise, smoking, drinking, and T2DM status

|  |  |  | TyG-WC | | | | | | | *P*_trend_^e^ | *P*_test_^f^ |
| --- | --- | --- | --- | --- | --- | --- | --- | --- | --- | --- | --- |
|  |  |  | Q1^c^ | Q2 | | Q3 | | Q4 | |  |  |
|  |  |  |  | HR/OR^b^ | 95% CI | HR/OR | 95% CI | HR/OR | 95% CI |  |  |
| Age |  |  |  |  |  |  |  |  |  |  |  |
|  | Model 1^a^ | > 50 | Ref (1.00)^d^ | 1.21 | (0.82,1.79) | 1.37 | (0.98,1.90) | 2.22 | **(1.56,3.18)***** | < 0.001 | < 0.001 |
|  |  | ≤ 50 | Ref (1.00) | 1.70 | (0.90,3.21) | 2.37 | **(1.21,4.62)*** | 5.02 | **(3.13,8.04)***** | < 0.001 | < 0.001 |
|  | Model 2 | > 50 | Ref (1.00) | 1.17 | (0.79,1.74) | 1.29 | (0.92,1.79) | 2.02 | **(1.41,2.89)***** | < 0.001 | 0.001 |
|  |  | ≤ 50 | Ref (1.00) | 1.55 | (0.82,2.92) | 2.08 | **(1.04,4.14)*** | 4.12 | **(2.57,6.61)***** | < 0.001 | < 0.001 |
|  | Model 3 | > 50 | Ref (1.00) | 1.18 | (0.79,1.75) | 1.29 | (0.92,1.81) | 2.09 | **(1.45,3.00)***** | < 0.001 | < 0.001 |
|  |  | ≤ 50 | Ref (1.00) | 1.52 | (0.81,2.87) | 2.10 | **(1.07,4.12)*** | 4.14 | **(2.56,6.71)***** | < 0.001 | < 0.001 |
|  | Model 4 | > 50 | Ref (1.00) | 1.17 | (0.78,1.75) | 1.23 | (0.88,1.73) | 1.88 | **(1.28,2.75)**** | < 0.001 | 0.004 |
|  |  | ≤ 50 | Ref (1.00) | 1.55 | (0.81,2.95) | 1.98 | (0.92,4.25) | 3.60 | **(2.08,6.23)***** | < 0.001 | < 0.001 |
| Sex |  |  |  |  |  |  |  |  |  |  |  |
|  | Model l | Male | Ref (1.00) | 1.21 | (0.69,2.14) | 1.41 | (0.80,2.51) | 2.64 | **(1.61,4.33)***** | < 0.001 | 0.001 |
|  |  | Female | Ref (1.00) | 1.28 | (0.89,1.85) | 1.42 | (0.99,2.04) | 2.74 | **(1.85,4.07)***** | < 0.001 | < 0.001 |
|  | Model 2 | Male | Ref (1.00) | 1.20 | (0.68,2.13) | 1.39 | (0.77,2.49) | 2.57 | **(1.54,4.27)***** | < 0.001 | 0.001 |
|  |  | Female | Ref (1.00) | 1.22 | (0.84,1.77) | 1.30 | (0.90,1.89) | 2.35 | **(1.57,3.52)***** | < 0.001 | < 0.001 |
|  | Model 3 | Male | Ref (1.00) | 1.19 | (0.67,2.13) | 1.36 | (0.76,2.46) | 2.51 | **(1.52,4.12)***** | < 0.001 | 0.001 |
|  |  | Female | Ref (1.00) | 1.16 | (0.79,1.71) | 1.23 | (0.85,1.80) | 2.28 | **(1.51,3.44)***** | < 0.001 | < 0.001 |
|  | Model 4 | Male | Ref (1.00) | 1.26 | (0.72,2.18) | 1.42 | (0.81,2.50) | 2.48 | **(1.56,3.96)***** | < 0.001 | 0.001 |
|  |  | Female | Ref (1.00) | 1.21 | (0.82,1.79) | 1.19 | (0.81,1.75) | 2.12 | **(1.40,3.23)**** | < 0.001 | 0.002 |
| Race |  |  |  |  |  |  |  |  |  |  |  |
|  | Model l | Non-hispanic white | Ref (1.00) | 1.09 | (0.72,1.66) | 1.33 | (0.92,1.92) | 2.47 | **(1.75,3.48)***** | < 0.001 | < 0.001 |
|  |  | Others | Ref (1.00) | 1.59 | **(1.07,2.36)*** | 1.36 | (0.93,1.99) | 2.88 | **(1.93,4.30)***** | < 0.001 | < 0.001 |
|  | Model 2 | Non-hispanic white | Ref (1.00) | 1.07 | (0.70,1.64) | 1.26 | (0.87,1.83) | 2.30 | **(1.62,3.26)***** | < 0.001 | < 0.001 |
|  |  | Others | Ref (1.00) | 1.58 | **(1.06,2.35)*** | 1.33 | (0.91,1.94) | 2.75 | **(1.84,4.10)***** | < 0.001 | < 0.001 |
|  | Model 3 | Non-hispanic white | Ref (1.00) | 1.06 | (0.69,1.63) | 1.23 | (0.85,1.79) | 2.22 | **(1.58,3.14)***** | < 0.001 | < 0.001 |
|  |  | Others | Ref (1.00) | 1.59 | **(1.07,2.36)*** | 1.34 | (0.92,1.94) | 2.75 | **(1.83,4.15)***** | < 0.001 | < 0.001 |
|  | Model 4 | Non-hispanic white | Ref (1.00) | 1.13 | (0.73,1.76) | 1.28 | (0.87,1.90) | 2.20 | **(1.55,3.12)***** | < 0.001 | < 0.001 |
|  |  | Others | Ref (1.00) | 1.65 | **(1.12,2.44)*** | 1.32 | (0.91,1.93) | 2.59 | **(1.72,3.89)***** | < 0.001 | < 0.001 |
| Exercise |  |  |  |  |  |  |  |  |  |  |  |
|  | Model l | Yes | Ref (1.00) | 1.32 | (0.80,2.18) | 1.31 | (0.81,2.11) | 2.58 | **(1.63,4.08)***** | < 0.001 | < 0.001 |
|  |  | No | Ref (1.00) | 1.04 | (0.62,1.74) | 1.39 | (0.91,2.11) | 2.23 | **(1.44,3.45)***** | < 0.001 | 0.001 |
|  | Model 2 | Yes | Ref (1.00) | 1.32 | (0.80,2.19) | 1.33 | (0.83,2.14) | 2.61 | **(1.65,4.13)***** | < 0.001 | < 0.001 |
|  |  | No | Ref (1.00) | 1.02 | (0.61,1.70) | 1.31 | (0.85,2.00) | 2.08 | **(1.35,3.21)**** | < 0.001 | 0.004 |
|  | Model 3 | Yes | Ref (1.00) | 1.32 | (0.79,2.19) | 1.32 | (0.82,2.14) | 2.62 | **(1.64,4.17)***** | < 0.001 | < 0.001 |
|  |  | No | Ref (1.00) | 1.02 | (0.61,1.70) | 1.29 | (0.84,1.98) | 2.06 | **(1.35,3.15)**** | < 0.001 | 0.003 |
|  | Model 4 | Yes | Ref (1.00) | 1.40 | (0.85,2.28) | 1.30 | (0.81,2.08) | 2.38 | **(1.51,3.76)***** | < 0.001 | 0.001 |
|  |  | No | Ref (1.00) | 1.07 | (0.63,1.82) | 1.41 | (0.90,2.20) | 2.18 | **(1.41,3.37)**** | < 0.001 | 0.002 |
| Smoking |  |  |  |  |  |  |  |  |  |  |  |
|  | Model l | Yes | Ref (1.00) | 1.02 | (0.56,1.88) | 0.85 | (0.45,1.58) | 1.97 | **(1.15,3.35)*** | 0.004 | 0.040 |
|  |  | No | Ref (1.00) | 1.39 | (0.95,2.05) | 1.72 | **(1.21,2.45)**** | 3.11 | **(2.28,4.25)***** | < 0.001 | < 0.001 |
|  | Model 2 | Yes | Ref (1.00) | 0.99 | (0.53,1.83) | 0.83 | (0.44,1.60) | 1.94 | **(1.12,3.36)*** | 0.004 | 0.056 |
|  |  | No | Ref (1.00) | 1.37 | (0.93,2.03) | 1.67 | **(1.16,2.41)**** | 2.99 | **(2.17,4.10)***** | < 0.001 | < 0.001 |
|  | Model 3 | Yes | Ref (1.00) | 0.98 | (0.53,1.81) | 0.84 | (0.44,1.58) | 1.94 | **(1.13,3.33)*** | 0.004 | 0.051 |
|  |  | No | Ref (1.00) | 1.35 | (0.91,1.99) | 1.62 | **(1.13,2.32)**** | 2.91 | **(2.14,3.97)***** | < 0.001 | < 0.001 |
|  | Model 4 | Yes | Ref (1.00) | 1.10 | (0.60,2.00) | 0.92 | (0.49,1.75) | 2.17 | **(1.26,3.75)**** | 0.002 | 0.017 |
|  |  | No | Ref (1.00) | 1.37 | (0.91,2.08) | 1.59 | **(1.08,2.34)*** | 2.65 | **(1.85,3.79)***** | < 0.001 | < 0.001 |
| Drinking |  |  |  |  |  |  |  |  |  |  |  |
|  | Model l | Yes | Ref (1.00) | 1.10 | (0.71,1.69) | 1.31 | (0.91,1.89) | 2.67 | **(1.87,3.80)***** | < 0.001 | < 0.001 |
|  |  | No | Ref (1.00) | 1.71 | **(1.07,2.74)*** | 1.73 | **(1.13,2.64)*** | 2.86 | **(1.89,4.32)***** | < 0.001 | < 0.001 |
|  | Model 2 | Yes | Ref (1.00) | 1.08 | (0.70,1.67) | 1.28 | (0.88,1.85) | 2.50 | **(1.75,3.58)***** | < 0.001 | < 0.001 |
|  |  | No | Ref (1.00) | 1.72 | **(1.07,2.77)*** | 1.69 | **(1.11,2.60)*** | 2.76 | **(1.81,4.19)***** | < 0.001 | < 0.001 |
|  | Model 3 | Yes | Ref (1.00) | 1.08 | (0.70,1.66) | 1.27 | (0.88,1.84) | 2.51 | **(1.77,3.57)***** | < 0.001 | < 0.001 |
|  |  | No | Ref (1.00) | 1.67 | **(1.02,2.72)*** | 1.61 | **(1.04,2.48)*** | 2.59 | **(1.69,3.97)***** | < 0.001 | < 0.001 |
|  | Model 4 | Yes | Ref (1.00) | 1.18 | (0.76,1.84) | 1.36 | (0.92,2.00) | 2.57 | **(1.79,3.69)***** | < 0.001 | < 0.001 |
|  |  | No | Ref (1.00) | 1.64 | **(1.01,2.66)*** | 1.52 | **(1.01,2.29)*** | 2.30 | **(1.49,3.55)***** | 0.001 | 0.001 |
| T2DM |  |  |  |  |  |  |  |  |  |  |  |
|  | Model l | Yes | Ref (1.00) | 1.09 | (0.31,3.81) | 1.17 | (0.38,3.62) | 2.36 | (0.73,7.68) | < 0.001 | 0.454 |
|  |  | No | Ref (1.00) | 1.21 | (0.86,1.72) | 1.34 | (0.99,1.80) | 2.11 | **(1.54,2.88)***** | < 0.001 | < 0.001 |
|  | Model 2 | Yes | Ref (1.00) | 1.09 | (0.32,3.75) | 1.19 | (0.40,3.53) | 2.28 | (0.73,7.16) | 0.001 | 0.466 |
|  |  | No | Ref (1.00) | 1.20 | (0.84,1.70) | 1.29 | (0.95,1.76) | 2.01 | **(1.45,2.79)***** | < 0.001 | < 0.001 |
|  | Model 3 | Yes | Ref (1.00) | 1.14 | (0.34,3.86) | 1.26 | (0.43,3.67) | 2.41 | (0.78,7.51) | 0.001 | 0.380 |
|  |  | No | Ref (1.00) | 1.18 | (0.82,1.68) | 1.26 | (0.92,1.71) | 1.97 | **(1.42,2.73)***** | < 0.001 | < 0.001 |
|  | Model 4 | Yes | Ref (1.00) | 1.20 | (0.38,3.75) | 1.28 | (0.48,3.42) | 2.45 | (0.86,6.96) | 0.001 | 0.276 |
|  |  | No | Ref (1.00) | 1.27 | (0.88,1.82) | 1.39 | **(1.01,1.91)*** | 2.22 | **(1.60,3.07)***** | < 0.001 | < 0.001 |

^a^The variables adjusted in each model were the factors mentioned above except the stratification variables.

^b^Data were listed as the weighted odd ratio estimates and 95% confidence intervals, with *p < 0.05, **p < 0.01, ***p < 0.001.

^c^Q, quintile.

^d^Ref, reference.

^e^Tests for trends based on the variables containing the median values for each quartile.

^f^*P*_test_ was the result of Bonfreni correction.

Supplementary Table 14. Association of TyG-WtHR with Total-CVD stratified by age, sex, race, exercise, smoking, drinking, and T2DM status

|  |  |  | TyG-WtHR | | | | | | | *P*_trend_^e^ | *P*_test_^f^ |
| --- | --- | --- | --- | --- | --- | --- | --- | --- | --- | --- | --- |
|  |  |  | Q1^c^ | Q2 | | Q3 | | Q4 | |  |  |
|  |  |  |  | HR/OR^b^ | 95% CI | HR/OR | 95% CI | HR/OR | 95% CI |  |  |
| Age |  |  |  |  |  |  |  |  |  |  |  |
|  | Model 1^a^ | > 50 | Ref (1.00)^d^ | 1.06 | (0.75,1.49) | 1.64 | **(1.22,2.22)**** | 2.38 | **(1.71,3.31)***** | < 0.001 | < 0.001 |
|  |  | ≤ 50 | Ref (1.00) | 1.65 | (0.88,3.12) | 2.54 | **(1.28,5.01)**** | 5.06 | **(3.10,8.27)***** | < 0.001 | < 0.001 |
|  | Model 2 | > 50 | Ref (1.00) | 1.01 | (0.72,1.43) | 1.54 | **(1.13,2.09)**** | 2.14 | **(1.53,2.98)***** | < 0.001 | < 0.001 |
|  |  | ≤ 50 | Ref (1.00) | 1.52 | (0.80,2.88) | 2.20 | **(1.10,4.39)**** | 4.09 | **(2.51,6.68)***** | < 0.001 | < 0.001 |
|  | Model 3 | > 50 | Ref (1.00) | 0.98 | (0.70,1.39) | 1.49 | **(1.09,2.03)*** | 2.10 | **(1.50,2.94)***** | < 0.001 | < 0.001 |
|  |  | ≤ 50 | Ref (1.00) | 1.46 | (0.78,2.76) | 2.18 | **(1.11,4.31)*** | 4.02 | **(2.45,6.61)***** | < 0.001 | < 0.001 |
|  | Model 4 | > 50 | Ref (1.00) | 1.00 | (0.71,1.41) | 1.49 | **(1.09,2.06)*** | 1.95 | **(1.37,2.79)***** | < 0.001 | 0.001 |
|  |  | ≤ 50 | Ref (1.00) | 1.51 | (0.79,2.87) | 2.11 | **(1.01,4.41)*** | 3.44 | **(1.96,6.05)***** | < 0.001 | < 0.001 |
| Sex |  |  |  |  |  |  |  |  |  |  |  |
|  | Model l | Male | Ref (1.00) | 1.01 | (0.65,1.59) | 1.70 | **(1.10,2.64)*** | 2.61 | **(1.71,4.00)***** | < 0.001 | < 0.001 |
|  |  | Female | Ref (1.00) | 1.16 | (0.76,1.76) | 1.40 | (0.99,1.99) | 2.61 | **(1.70,4.01)***** | < 0.001 | < 0.001 |
|  | Model 2 | Male | Ref (1.00) | 1.00 | (0.64,1.57) | 1.67 | **(1.07,2.60)*** | 2.55 | **(1.64,3.96)***** | < 0.001 | < 0.001 |
|  |  | Female | Ref (1.00) | 1.06 | (0.69,1.64) | 1.26 | (0.87,1.82) | 2.18 | **(1.40,3.40)**** | < 0.001 | 0.002 |
|  | Model 3 | Male | Ref (1.00) | 0.98 | (0.63,1.53) | 1.61 | **(1.04,2.50)*** | 2.44 | **(1.59,3.74)***** | < 0.001 | < 0.001 |
|  |  | Female | Ref (1.00) | 1.02 | (0.65,1.58) | 1.18 | (0.80,1.73) | 2.07 | **(1.32,3.26)**** | < 0.001 | 0.006 |
|  | Model 4 | Male | Ref (1.00) | 1.03 | (0.67,1.58) | 1.64 | **(1.06,2.54)*** | 2.42 | **(1.60,3.66)***** | < 0.001 | < 0.001 |
|  |  | Female | Ref (1.00) | 1.08 | (0.70,1.67) | 1.21 | (0.82,1.80) | 1.98 | **(1.25,3.13)**** | 0.001 | 0.012 |
| Race |  |  |  |  |  |  |  |  |  |  |  |
|  | Model l | Non-hispanic white | Ref (1.00) | 0.95 | (0.67,1.34) | 1.46 | **(1.08,1.98)*** | 2.37 | **(1.69,3.33)***** | < 0.001 | < 0.001 |
|  |  | Others | Ref (1.00) | 1.34 | (0.88,2.04) | 1.45 | (0.98,2.15) | 2.63 | **(1.79,3.87)***** | < 0.001 | < 0.001 |
|  | Model 2 | Non-hispanic white | Ref (1.00) | 0.91 | (0.64,1.30) | 1.38 | **(1.01,1.88)*** | 2.18 | **(1.53,3.09)***** | < 0.001 | < 0.001 |
|  |  | Others | Ref (1.00) | 1.32 | (0.87,2.00) | 1.41 | (0.95,2.07) | 2.48 | **(1.70,3.63)***** | < 0.001 | < 0.001 |
|  | Model 3 | Non-hispanic white | Ref (1.00) | 0.88 | (0.62,1.26) | 1.32 | (0.96,1.80) | 2.07 | **(1.46,2.94)***** | < 0.001 | < 0.001 |
|  |  | Others | Ref (1.00) | 1.31 | (0.87,2.00) | 1.41 | (0.96,2.08) | 2.46 | **(1.68,3.61)***** | < 0.001 | < 0.001 |
|  | Model 4 | Non-hispanic white | Ref (1.00) | 0.96 | (0.67,1.37) | 1.40 | (1.00,1.97) | 2.10 | **(1.47,3.00)***** | < 0.001 | < 0.001 |
|  |  | Others | Ref (1.00) | 1.40 | (0.92,2.13) | 1.48 | (1.00,2.19) | 2.37 | **(1.60,3.53)***** | < 0.001 | < 0.001 |
| Exercise |  |  |  |  |  |  |  |  |  |  |  |
|  | Model l | Yes | Ref (1.00) | 1.10 | (0.71,1.72) | 1.36 | (0.88,2.11) | 2.45 | **(1.50,4.02)***** | < 0.001 | 0.001 |
|  |  | No | Ref (1.00) | 0.99 | (0.62,1.56) | 1.65 | **(1.16,2.36)**** | 2.23 | **(1.47,3.38)***** | < 0.001 | 0.001 |
|  | Model 2 | Yes | Ref (1.00) | 1.09 | (0.69,1.71) | 1.36 | (0.88,2.11) | 2.46 | **(1.50,4.04)***** | < 0.001 | 0.001 |
|  |  | No | Ref (1.00) | 0.96 | (0.61,1.51) | 1.53 | **(1.07,2.19)*** | 2.05 | **(1.36,3.08)**** | < 0.001 | 0.002 |
|  | Model 3 | Yes | Ref (1.00) | 1.09 | (0.69,1.72) | 1.34 | (0.87,2.07) | 2.45 | **(1.48,4.04)**** | < 0.001 | 0.002 |
|  |  | No | Ref (1.00) | 0.93 | (0.59,1.46) | 1.50 | **(1.05,2.15)*** | 1.96 | **(1.30,2.94)**** | < 0.001 | 0.004 |
|  | Model 4 | Yes | Ref (1.00) | 1.14 | (0.73,1.79) | 1.36 | (0.87,2.13) | 2.28 | **(1.38,3.76)**** | < 0.001 | 0.005 |
|  |  | No | Ref (1.00) | 1.03 | (0.65,1.62) | 1.65 | **(1.14,2.40)**** | 2.11 | **(1.39,3.19)**** | < 0.001 | 0.005 |
| Smoking |  |  |  |  |  |  |  |  |  |  |  |
|  | Model l | Yes | Ref (1.00) | 0.84 | (0.47,1.51) | 1.10 | (0.65,1.86) | 1.96 | **(1.11,3.47)*** | 0.005 | 0.063 |
|  |  | No | Ref (1.00) | 1.25 | (0.91,1.71) | 1.85 | **(1.36,2.52)***** | 3.07 | **(2.33,4.05)***** | < 0.001 | < 0.001 |
|  | Model 2 | Yes | Ref (1.00) | 0.82 | (0.44,1.52) | 1.10 | (0.65,1.88) | 1.91 | **(1.06,3.43)*** | 0.007 | 0.093 |
|  |  | No | Ref (1.00) | 1.23 | (0.89,1.69) | 1.80 | **(1.30,2.49)**** | 2.95 | **(2.22,3.92)***** | < 0.001 | < 0.001 |
|  | Model 3 | Yes | Ref (1.00) | 0.81 | (0.44,1.50) | 1.09 | (0.64,1.87) | 1.90 | **(1.06,3.39)*** | 0.007 | 0.092 |
|  |  | No | Ref (1.00) | 1.17 | (0.86,1.61) | 1.71 | **(1.25,2.34)**** | 2.80 | **(2.11,3.70)***** | < 0.001 | < 0.001 |
|  | Model 4 | Yes | Ref (1.00) | 0.92 | (0.52,1.63) | 1.24 | (0.71,2.16) | 2.15 | **(1.22,3.77)**** | 0.003 | 0.026 |
|  |  | No | Ref (1.00) | 1.21 | (0.88,1.68) | 1.73 | **(1.24,2.43)**** | 2.61 | **(1.90,3.59)***** | < 0.001 | < 0.001 |
| Drinking |  |  |  |  |  |  |  |  |  |  |  |
|  | Model l | Yes | Ref (1.00) | 1.05 | (0.73,1.52) | 1.50 | **(1.12,2.03)**** | 2.67 | **(1.90,3.75)***** | < 0.001 | < 0.001 |
|  |  | No | Ref (1.00) | 1.18 | (0.74,1.88) | 1.76 | **(1.08,2.86)*** | 2.69 | **(1.73,4.17)***** | < 0.001 | < 0.001 |
|  | Model 2 | Yes | Ref (1.00) | 1.03 | (0.71,1.48) | 1.46 | **(1.08,1.97)*** | 2.47 | **(1.75,3.49)***** | < 0.001 | < 0.001 |
|  |  | No | Ref (1.00) | 1.20 | (0.73,1.98) | 1.78 | **(1.09,2.92)*** | 2.67 | **(1.70,4.18)***** | < 0.001 | < 0.001 |
|  | Model 3 | Yes | Ref (1.00) | 1.01 | (0.70,1.46) | 1.44 | **(1.06,1.94)*** | 2.44 | **(1.74,3.42)***** | < 0.001 | < 0.001 |
|  |  | No | Ref (1.00) | 1.14 | (0.68,1.91) | 1.61 | (0.96,2.68) | 2.45 | **(1.54,3.90)***** | < 0.001 | 0.001 |
|  | Model 4 | Yes | Ref (1.00) | 1.12 | (0.77,1.61) | 1.56 | **(1.14,2.13)**** | 2.53 | **(1.78,3.59)***** | < 0.001 | < 0.001 |
|  |  | No | Ref (1.00) | 1.14 | (0.68,1.91) | 1.58 | (0.94,2.65) | 2.22 | **(1.38,3.56)**** | < 0.001 | 0.004 |
| T2DM |  |  |  |  |  |  |  |  |  |  |  |
|  | Model l | Yes | Ref (1.00) | 0.80 | (0.21,3.10) | 1.48 | (0.37,5.94) | 2.34 | (0.59,9.27) | 0.001 | 0.665 |
|  |  | No | Ref (1.00) | 1.06 | (0.78,1.44) | 1.43 | **(1.09,1.86)*** | 2.03 | **(1.49,2.75)***** | < 0.001 | < 0.001 |
|  | Model 2 | Yes | Ref (1.00) | 0.81 | (0.21,3.08) | 1.48 | (0.39,5.59) | 2.28 | (0.61,8.56) | 0.001 | 0.658 |
|  |  | No | Ref (1.00) | 1.03 | (0.76,1.40) | 1.37 | **(1.04,1.80)*** | 1.91 | **(1.39,2.64)***** | < 0.001 | < 0.001 |
|  | Model 3 | Yes | Ref (1.00) | 0.83 | (0.22,3.09) | 1.50 | (0.40,5.58) | 2.33 | (0.62,8.69) | 0.001 | 0.616 |
|  |  | No | Ref (1.00) | 1.00 | (0.73,1.37) | 1.32 | **(1.01,1.73)*** | 1.85 | **(1.34,2.55)***** | < 0.001 | 0.001 |
|  | Model 4 | Yes | Ref (1.00) | 0.86 | (0.25,2.93) | 1.52 | (0.45,5.13) | 2.40 | (0.71,8.09) | 0.001 | 0.465 |
|  |  | No | Ref (1.00) | 1.10 | (0.80,1.51) | 1.50 | **(1.13,2.00)**** | 2.11 | **(1.52,2.92)***** | < 0.001 | < 0.001 |

^a^The variables adjusted in each model were the factors mentioned above except the stratification variables.

^b^Data were listed as the weighted odd ratio estimates and 95% confidence intervals, with *p < 0.05, **p < 0.01, ***p < 0.001.

^c^Q, quintile.

^d^Ref, reference.

^e^Tests for trends based on the variables containing the median values for each quartile.

^f^*P*_test_ was the result of Bonfreni correction.

Supplementary Table 15. Association of TyG-BMI with Total-CVD stratified by age, sex, race, exercise, smoking, drinking, and T2DM status

|  |  |  | TyG-BMI | | | | | | | *P*_trend_^e^ | *P*_test_^f^ |
| --- | --- | --- | --- | --- | --- | --- | --- | --- | --- | --- | --- |
|  |  |  | Q1^c^ | Q2 | | Q3 | | Q4 | |  |  |
|  |  |  |  | HR/OR^b^ | 95% CI | HR/OR | 95% CI | HR/OR | 95% CI |  |  |
| Age |  |  |  |  |  |  |  |  |  |  |  |
|  | Model 1^a^ | > 50 | Ref (1.00)^d^ | 0.97 | (0.74,1.28) | 1.187 | (0.90,1.57) | 1.598 | **(1.23,2.08)**** | < 0.001 | 0.002 |
|  |  | ≤ 50 | Ref (1.00) | 1.98 | (0.91,4.33) | 2.447 | **(1.39,4.31)**** | 4.062 | **(2.40,6.89)***** | < 0.001 | < 0.001 |
|  | Model 2 | > 50 | Ref (1.00) | 0.94 | (0.71,1.24) | 1.135 | (0.85,1.51) | 1.471 | **(1.13,1.91)**** | < 0.001 | 0.014 |
|  |  | ≤ 50 | Ref (1.00) | 1.89 | (0.87,4.10) | 2.17 | **(1.23,3.82)**** | 3.406 | **(2.01,5.78)***** | < 0.001 | < 0.001 |
|  | Model 3 | > 50 | Ref (1.00) | 0.93 | (0.70,1.24) | 1.125 | (0.84,1.51) | 1.513 | **(1.15,1.99)**** | < 0.001 | 0.011 |
|  |  | ≤ 50 | Ref (1.00) | 1.84 | (0.85,3.97) | 2.173 | **(1.24,3.80)**** | 3.424 | **(1.99,5.87)***** | < 0.001 | < 0.001 |
|  | Model 4 | > 50 | Ref (1.00) | 0.96 | (0.71,1.30) | 1.118 | (0.83,1.51) | 1.389 | **(1.02,1.90)*** | 0.013 | 0.117 |
|  |  | ≤ 50 | Ref (1.00) | 1.85 | (0.85,4.02) | 2.095 | **(1.15,3.81)*** | 2.786 | **(1.54,5.06)**** | < 0.001 | 0.003 |
| Sex |  |  |  |  |  |  |  |  |  |  |  |
|  | Model l | Male | Ref (1.00) | 1.17 | (0.80,1.72) | 1.401 | (0.94,2.09) | 2.582 | **(1.78,3.75)***** | < 0.001 | < 0.001 |
|  |  | Female | Ref (1.00) | 1.13 | (0.77,1.66) | 1.461 | (0.96,2.22) | 2.178 | **(1.47,3.22)***** | < 0.001 | < 0.001 |
|  | Model 2 | Male | Ref (1.00) | 1.15 | (0.78,1.71) | 1.37 | (0.91,2.06) | 2.509 | **(1.72,3.67)***** | < 0.001 | < 0.001 |
|  |  | Female | Ref (1.00) | 1.06 | (0.71,1.57) | 1.325 | (0.86,2.04) | 1.873 | **(1.26,2.80)**** | 0.001 | 0.007 |
|  | Model 3 | Male | Ref (1.00) | 1.14 | (0.76,1.69) | 1.353 | (0.90,2.03) | 2.446 | **(1.68,3.57)***** | < 0.001 | < 0.001 |
|  |  | Female | Ref (1.00) | 1.02 | (0.68,1.53) | 1.257 | (0.80,1.97) | 1.816 | **(1.21,2.72)**** | 0.002 | 0.013 |
|  | Model 4 | Male | Ref (1.00) | 1.17 | (0.79,1.73) | 1.334 | (0.89,1.99) | 2.336 | **(1.62,3.37)***** | < 0.001 | < 0.001 |
|  |  | Female | Ref (1.00) | 1.04 | (0.69,1.57) | 1.239 | (0.80,1.92) | 1.635 | **(1.08,2.48)*** | 0.011 | 0.065 |
| Race |  |  |  |  |  |  |  |  |  |  |  |
|  | Model l | Non-hispanic white | Ref (1.00) | 1.05 | (0.78,1.41) | 1.371 | **(1.01,1.86)*** | 2.22 | **(1.63,3.02)***** | < 0.001 | < 0.001 |
|  |  | Others | Ref (1.00) | 1.37 | (0.89,2.10) | 1.223 | (0.83,1.81) | 2.302 | **(1.63,3.24)***** | < 0.001 | < 0.001 |
|  | Model 2 | Non-hispanic white | Ref (1.00) | 1 | (0.74,1.35) | 1.299 | (0.95,1.78) | 2.047 | **(1.50,2.80)***** | < 0.001 | < 0.001 |
|  |  | Others | Ref (1.00) | 1.35 | (0.88,2.07) | 1.195 | (0.81,1.76) | 2.206 | **(1.58,3.08)***** | < 0.001 | < 0.001 |
|  | Model 3 | Non-hispanic white | Ref (1.00) | 0.98 | (0.72,1.34) | 1.259 | (0.92,1.73) | 1.997 | **(1.47,2.72)***** | < 0.001 | < 0.001 |
|  |  | Others | Ref (1.00) | 1.35 | (0.88,2.07) | 1.207 | (0.82,1.77) | 2.191 | **(1.56,3.08)***** | < 0.001 | < 0.001 |
|  | Model 4 | Non-hispanic white | Ref (1.00) | 1.04 | (0.74,1.44) | 1.283 | (0.92,1.79) | 1.894 | **(1.36,2.63)***** | < 0.001 | 0.001 |
|  |  | Others | Ref (1.00) | 1.36 | (0.89,2.09) | 1.187 | (0.81,1.75) | 2.008 | **(1.42,2.84)***** | 0.001 | < 0.001 |
| Exercise |  |  |  |  |  |  |  |  |  |  |  |
|  | Model l | Yes | Ref (1.00) | 1.10 | (0.74,1.65) | 1.24 | (0.85,1.81) | 2.15 | **(1.42,3.26)***** | < 0.001 | 0.001 |
|  |  | No | Ref (1.00) | 1.14 | (0.74,1.75) | 1.57 | **(1.00,2.45)*** | 2.06 | **(1.34,3.16)**** | < 0.001 | 0.004 |
|  | Model 2 | Yes | Ref (1.00) | 1.09 | (0.72,1.64) | 1.24 | (0.86,1.81) | 2.16 | **(1.43,3.28)***** | < 0.001 | 0.001 |
|  |  | No | Ref (1.00) | 1.09 | (0.71,1.69) | 1.48 | (0.94,2.34) | 1.88 | **(1.23,2.88)**** | 0.001 | 0.012 |
|  | Model 3 | Yes | Ref (1.00) | 1.09 | (0.72,1.65) | 1.25 | (0.85,1.84) | 2.20 | **(1.45,3.35)***** | < 0.001 | 0.001 |
|  |  | No | Ref (1.00) | 1.07 | (0.69,1.66) | 1.45 | (0.93,2.26) | 1.83 | **(1.20,2.78)**** | 0.001 | 0.017 |
|  | Model 4 | Yes | Ref (1.00) | 1.09 | (0.72,1.65) | 1.23 | (0.84,1.80) | 1.91 | **(1.24,2.93)**** | < 0.001 | 0.011 |
|  |  | No | Ref (1.00) | 1.15 | (0.73,1.81) | 1.51 | (0.95,2.41) | 1.84 | **(1.20,2.83)**** | 0.002 | 0.017 |
| Smoking |  |  |  |  |  |  |  |  |  |  |  |
|  | Model l | Yes | Ref (1.00) | 0.94 | (0.52,1.67) | 1.057 | (0.66,1.69) | 1.837 | **(1.13,2.99)*** | 0.009 | 0.045 |
|  |  | No | Ref (1.00) | 1.3 | (0.96,1.77) | 1.658 | **(1.21,2.28)**** | 2.775 | **(2.11,3.65)***** | < 0.001 | < 0.001 |
|  | Model 2 | Yes | Ref (1.00) | 0.94 | (0.51,1.73) | 1.071 | (0.66,1.74) | 1.83 | **(1.11,3.02)*** | 0.010 | 0.056 |
|  |  | No | Ref (1.00) | 1.28 | (0.93,1.75) | 1.613 | **(1.16,2.24)**** | 2.651 | **(1.99,3.53)***** | < 0.001 | < 0.001 |
|  | Model 3 | Yes | Ref (1.00) | 0.93 | (0.51,1.69) | 1.076 | (0.66,1.74) | 1.828 | **(1.12,2.99)*** | 0.010 | 0.051 |
|  |  | No | Ref (1.00) | 1.25 | (0.91,1.71) | 1.553 | **(1.12,2.16)**** | 2.575 | **(1.94,3.43)***** | < 0.001 | < 0.001 |
|  | Model 4 | Yes | Ref (1.00) | 1.01 | (0.57,1.77) | 1.19 | (0.73,1.93) | 1.941 | **(1.19,3.18)**** | 0.006 | 0.027 |
|  |  | No | Ref (1.00) | 1.26 | (0.89,1.77) | 1.504 | **(1.06,2.13)*** | 2.288 | **(1.65,3.19)***** | < 0.001 | < 0.001 |
| Drinking |  |  |  |  |  |  |  |  |  |  |  |
|  | Model l | Yes | Ref (1.00) | 1.02 | (0.76,1.37) | 1.27 | (0.94,1.72) | 2.37 | **(1.76,3.20)***** | < 0.001 | < 0.001 |
|  |  | No | Ref (1.00) | 1.59 | (0.99,2.56) | 1.93 | **(1.31,2.83)**** | 2.38 | **(1.53,3.70)***** | < 0.001 | 0.001 |
|  | Model 2 | Yes | Ref (1.00) | 1.02 | (0.75,1.38) | 1.26 | (0.92,1.72) | 2.26 | **(1.69,3.03)***** | < 0.001 | < 0.001 |
|  |  | No | Ref (1.00) | 1.63 | (1.01,2.63) | 1.94 | **(1.32,2.85)**** | 2.40 | **(1.55,3.72)***** | < 0.001 | < 0.001 |
|  | Model 3 | Yes | Ref (1.00) | 1.00 | (0.74,1.36) | 1.24 | (0.91,1.69) | 2.25 | **(1.69,3.00)***** | < 0.001 | < 0.001 |
|  |  | No | Ref (1.00) | 1.55 | (0.95,2.55) | 1.84 | **(1.23,2.76)**** | 2.23 | **(1.42,3.50)**** | 0.001 | 0.002 |
|  | Model 4 | Yes | Ref (1.00) | 1.06 | (0.77,1.45) | 1.29 | (0.93,1.79) | 2.20 | **(1.62,2.98)***** | < 0.001 | < 0.001 |
|  |  | No | Ref (1.00) | 1.55 | (0.93,2.57) | 1.76 | **(1.19,2.60)**** | 1.97 | **(1.25,3.10)**** | 0.005 | 0.011 |
| T2DM |  |  |  |  |  |  |  |  |  |  |  |
|  | Model l | Yes | Ref (1.00) | 1.23 | (0.55,2.75) | 2.08 | **(1.00,4.32)*** | 2.67 | **(1.22,5.86)**** | 0.002 | 0.044 |
|  |  | No | Ref (1.00) | 1.11 | (0.83,1.48) | 1.19 | (0.90,1.59) | 1.83 | **(1.39,2.41)***** | < 0.001 | < 0.001 |
|  | Model 2 | Yes | Ref (1.00) | 1.17 | (0.52,2.66) | 2.01 | (0.98,4.12) | 2.48 | **(1.14,5.39)*** | 0.003 | 0.069 |
|  |  | No | Ref (1.00) | 1.08 | (0.81,1.45) | 1.16 | (0.87,1.55) | 1.74 | **(1.31,2.31)***** | < 0.001 | 0.001 |
|  | Model 3 | Yes | Ref (1.00) | 1.17 | (0.52,2.64) | 1.99 | (0.98,4.02) | 2.48 | **(1.15,5.35)*** | 0.003 | 0.064 |
|  |  | No | Ref (1.00) | 1.07 | (0.79,1.43) | 1.13 | (0.85,1.52) | 1.72 | **(1.29,2.29)***** | < 0.001 | 0.001 |
|  | Model 4 | Yes | Ref (1.00) | 1.15 | (0.53,2.52) | 1.91 | (0.98,3.73) | 2.42 | **(1.17,4.99)*** | 0.004 | 0.052 |
|  |  | No | Ref (1.00) | 1.15 | (0.84,1.56) | 1.24 | (0.92,1.67) | 1.84 | **(1.39,2.45)***** | < 0.001 | < 0.001 |

^a^The variables adjusted in each model were the factors mentioned above except the stratification variables.

^b^Data were listed as the weighted odd ratio estimates and 95% confidence intervals, with *p < 0.05, **p < 0.01, ***p < 0.001.

^c^Q, quintile.

^d^Ref, reference.

^e^Tests for trends based on the variables containing the median values for each quartile.

^f^*P*_test_ was the result of Bonfreni correction.

Supplementary Table 16. Association of TyG with congestive heart failure stratified by age, sex, race, exercise, smoking, drinking, and T2DM status

|  |  |  | TyG | | | | | | | *P*_trend_^e^ | *P*_test_^f^ |
| --- | --- | --- | --- | --- | --- | --- | --- | --- | --- | --- | --- |
|  |  |  | Q1^c^ | Q2 | | Q3 | | Q4 | |  |  |
|  |  |  |  | HR/OR^b^ | 95% CI | HR/OR | 95% CI | HR/OR | 95% CI |  |  |
| Age |  |  |  |  |  |  |  |  |  |  |  |
|  | Model 1^a^ | > 50 | Ref (1.00)^d^ | 1.29 | (0.81,2.05) | 1.48 | (0.89,2.49) | 2.30 | **(1.37,3.85)**** | 0.001 | 0.005 |
|  |  | ≤ 50 | Ref (1.00) | 0.79 | (0.26,2.44) | 1.81 | (0.69,4.72) | 4.79 | **(1.80,12.79)**** | 0.002 | 0.006 |
|  | Model 2 | > 50 | Ref (1.00) | 1.18 | (0.75,1.87) | 1.33 | (0.80,2.21) | 1.96 | **(1.17,3.29)*** | 0.004 | 0.033 |
|  |  | ≤ 50 | Ref (1.00) | 0.68 | (0.22,2.07) | 1.54 | (0.58,4.10) | 3.61 | **(1.44,9.06)**** | 0.004 | 0.020 |
|  | Model 3 | > 50 | Ref (1.00) | 1.19 | (0.75,1.89) | 1.35 | (0.81,2.25) | 1.97 | **(1.18,3.30)*** | 0.004 | 0.031 |
|  |  | ≤ 50 | Ref (1.00) | 0.63 | (0.20,2.01) | 1.43 | (0.53,3.85) | 3.54 | **(1.39,9.01)**** | 0.005 | 0.026 |
|  | Model 4 | > 50 | Ref (1.00) | 1.22 | (0.76,1.97) | 1.38 | (0.80,2.37) | 1.84 | **(1.06,3.22)*** | 0.019 | 0.096 |
|  |  | ≤ 50 | Ref (1.00) | 0.60 | (0.18,1.96) | 1.43 | (0.48,4.23) | 2.54 | (0.75,8.56) | 0.083 | 0.396 |
| Sex |  |  |  |  |  |  |  |  |  |  |  |
|  | Model l | Male | Ref (1.00) | 0.89 | (0.48,1.62) | 1.08 | (0.56,2.07) | 2.11 | **(1.14,3.93)*** | 0.002 | 0.056 |
|  |  | Female | Ref (1.00) | 1.48 | (0.81,2.69) | 1.87 | (0.94,3.70) | 2.57 | **(1.31,5.06)**** | 0.005 | 0.020 |
|  | Model 2 | Male | Ref (1.00) | 0.85 | (0.47,1.56) | 1.03 | (0.54,1.97) | 1.97 | **(1.06,3.65)*** | 0.004 | 0.094 |
|  |  | Female | Ref (1.00) | 1.35 | (0.73,2.48) | 1.66 | (0.85,3.26) | 2.15 | **(1.11,4.15)*** | 0.017 | 0.070 |
|  | Model 3 | Male | Ref (1.00) | 0.84 | (0.46,1.55) | 1.01 | (0.53,1.92) | 1.91 | **(1.03,3.52)*** | 0.005 | 0.118 |
|  |  | Female | Ref (1.00) | 1.37 | (0.74,2.54) | 1.69 | (0.86,3.32) | 2.17 | **(1.13,4.19)*** | 0.015 | 0.064 |
|  | Model 4 | Male | Ref (1.00) | 0.85 | (0.46,1.57) | 0.99 | (0.52,1.89) | 1.86 | (0.98,3.52) | 0.013 | 0.174 |
|  |  | Female | Ref (1.00) | 1.52 | (0.80,2.90) | 1.80 | (0.87,3.72) | 1.89 | (0.96,3.73) | 0.063 | 0.200 |
| Race |  |  |  |  |  |  |  |  |  |  |  |
|  | Model l | Non-hispanic white | Ref (1.00) | 1.03 | (0.62,1.69) | 1.26 | (0.73,2.20) | 2.23 | **(1.34,3.69)**** | 0.001 | 0.007 |
|  |  | Others | Ref (1.00) | 1.33 | (0.61,2.89) | 1.71 | (0.78,3.74) | 2.20 | **(1.02,4.74)*** | 0.022 | 0.133 |
|  | Model 2 | Non-hispanic white | Ref (1.00) | 0.95 | (0.57,1.58) | 1.15 | (0.66,2.00) | 1.92 | **(1.16,3.18)*** | 0.002 | 0.036 |
|  |  | Others | Ref (1.00) | 1.25 | (0.58,2.71) | 1.62 | (0.75,3.52) | 2.04 | (0.97,4.31) | 0.031 | 0.186 |
|  | Model 3 | Non-hispanic white | Ref (1.00) | 0.95 | (0.57,1.58) | 1.13 | (0.66,1.95) | 1.87 | **(1.14,3.07)*** | 0.003 | 0.042 |
|  |  | Others | Ref (1.00) | 1.30 | (0.61,2.77) | 1.74 | (0.82,3.70) | 2.08 | (0.99,4.40) | 0.029 | 0.163 |
|  | Model 4 | Non-hispanic white | Ref (1.00) | 0.99 | (0.58,1.69) | 1.14 | (0.63,2.06) | 1.74 | **(1.01,3.01)*** | 0.013 | 0.140 |
|  |  | Others | Ref (1.00) | 1.36 | (0.62,2.99) | 1.87 | (0.91,3.86) | 1.93 | (0.92,4.05) | 0.043 | 0.247 |
| Exercise |  |  |  |  |  |  |  |  |  |  |  |
|  | Model l | Yes | Ref (1.00) | 1.23 | (0.74,2.04) | 1.49 | (0.85,2.59) | 2.21 | **(1.32,3.69)**** | < 0.001 | 0.008 |
|  |  | No | Ref (1.00) | 0.88 | (0.42,1.83) | 1.18 | (0.56,2.46) | 2.15 | **(1.01,4.56)*** | 0.034 | 0.141 |
|  | Model 2 | Yes | Ref (1.00) | 1.21 | (0.73,2.01) | 1.45 | (0.83,2.53) | 2.14 | **(1.28,3.59)**** | < 0.001 | 0.012 |
|  |  | No | Ref (1.00) | 0.85 | (0.42,1.70) | 1.11 | (0.54,2.28) | 1.91 | (0.90,4.04) | 0.066 | 0.274 |
|  | Model 3 | Yes | Ref (1.00) | 1.21 | (0.73,2.01) | 1.45 | (0.83,2.56) | 2.15 | **(1.29,3.58)**** | < 0.001 | 0.011 |
|  |  | No | Ref (1.00) | 0.86 | (0.42,1.76) | 1.11 | (0.54,2.26) | 1.89 | (0.90,3.94) | 0.064 | 0.273 |
|  | Model 4 | Yes | Ref (1.00) | 1.23 | (0.73,2.05) | 1.44 | (0.81,2.57) | 1.83 | **(1.11,3.04)*** | 0.008 | 0.058 |
|  |  | No | Ref (1.00) | 0.93 | (0.45,1.94) | 1.19 | (0.57,2.51) | 2.08 | (0.93,4.66) | 0.053 | 0.218 |
| Smoking |  |  |  |  |  |  |  |  |  |  |  |
|  | Model l | Yes | Ref (1.00) | 0.89 | (0.46,1.71) | 0.87 | (0.40,1.90) | 1.62 | (0.78,3.35) | 0.178 | 0.588 |
|  |  | No | Ref (1.00) | 1.14 | (0.69,1.87) | 1.49 | (0.84,2.65) | 2.39 | **(1.36,4.23)**** | < 0.001 | 0.009 |
|  | Model 2 | Yes | Ref (1.00) | 0.86 | (0.44,1.70) | 0.85 | (0.38,1.88) | 1.63 | (0.76,3.47) | 0.182 | 0.614 |
|  |  | No | Ref (1.00) | 1.08 | (0.66,1.78) | 1.42 | (0.80,2.51) | 2.17 | **(1.23,3.84)**** | 0.001 | 0.025 |
|  | Model 3 | Yes | Ref (1.00) | 0.90 | (0.45,1.78) | 0.87 | (0.38,2.02) | 1.67 | (0.74,3.79) | 0.202 | 0.653 |
|  |  | No | Ref (1.00) | 1.08 | (0.65,1.79) | 1.40 | (0.79,2.49) | 2.14 | **(1.20,3.81)**** | 0.001 | 0.030 |
|  | Model 4 | Yes | Ref (1.00) | 1.03 | (0.50,2.12) | 1.02 | (0.45,2.29) | 1.92 | (0.89,4.14) | 0.124 | 0.286 |
|  |  | No | Ref (1.00) | 1.09 | (0.65,1.84) | 1.36 | (0.74,2.49) | 1.85 | **(1.01,3.41)*** | 0.014 | 0.143 |
| Drinking |  |  |  |  |  |  |  |  |  |  |  |
|  | Model l | Yes | Ref (1.00) | 0.96 | (0.59,1.56) | 1.07 | (0.61,1.87) | 2.40 | **(1.46,3.92)***** | < 0.001 | 0.002 |
|  |  | No | Ref (1.00) | 1.51 | (0.65,3.51) | 2.33 | (0.83,6.56) | 2.06 | (0.85,4.97) | 0.058 | 0.139 |
|  | Model 2 | Yes | Ref (1.00) | 0.91 | (0.56,1.46) | 0.98 | (0.56,1.70) | 2.13 | **(1.30,3.50)**** | 0.001 | 0.009 |
|  |  | No | Ref (1.00) | 1.46 | (0.61,3.49) | 2.08 | (0.76,5.69) | 1.83 | (0.75,4.50) | 0.134 | 0.463 |
|  | Model 3 | Yes | Ref (1.00) | 0.92 | (0.57,1.48) | 0.98 | (0.57,1.70) | 2.12 | **(1.30,3.47)**** | 0.001 | 0.009 |
|  |  | No | Ref (1.00) | 1.44 | (0.59,3.49) | 2.10 | (0.76,5.84) | 1.77 | (0.72,4.34) | 0.152 | 0.455 |
|  | Model 4 | Yes | Ref (1.00) | 0.91 | (0.56,1.48) | 0.95 | (0.55,1.66) | 1.84 | **(1.10,3.10)*** | 0.009 | 0.064 |
|  |  | No | Ref (1.00) | 1.72 | (0.69,4.32) | 2.60 | (0.88,7.74) | 1.98 | (0.77,5.09) | 0.150 | 0.253 |
| T2DM |  |  |  |  |  |  |  |  |  |  |  |
|  | Model l | Yes | Ref (1.00) | 0.81 | (0.30,2.20) | 0.85 | (0.30,2.45) | 1.34 | (0.58,3.10) | 0.112 | 1.000 |
|  |  | No | Ref (1.00) | 1.12 | (0.74,1.69) | 1.36 | (0.82,2.27) | 1.88 | **(1.10,3.20)*** | 0.015 | 0.063 |
|  | Model 2 | Yes | Ref (1.00) | 0.79 | (0.28,2.19) | 0.84 | (0.29,2.43) | 1.29 | (0.56,3.00) | 0.140 | 1.000 |
|  |  | No | Ref (1.00) | 1.05 | (0.69,1.58) | 1.26 | (0.75,2.10) | 1.69 | (0.99,2.89) | 0.036 | 0.163 |
|  | Model 3 | Yes | Ref (1.00) | 0.73 | (0.26,2.08) | 0.82 | (0.29,2.30) | 1.23 | (0.53,2.89) | 0.137 | 1.000 |
|  |  | No | Ref (1.00) | 1.06 | (0.70,1.60) | 1.26 | (0.75,2.11) | 1.69 | (0.99,2.89) | 0.037 | 0.158 |
|  | Model 4 | Yes | Ref (1.00) | 0.76 | (0.26,2.18) | 0.84 | (0.29,2.45) | 1.34 | (0.55,3.28) | 0.102 | 1.000 |
|  |  | No | Ref (1.00) | 1.20 | (0.78,1.85) | 1.51 | (0.88,2.58) | 2.33 | **(1.37,3.96)**** | 0.001 | 0.006 |

^a^The variables adjusted in each model were the factors mentioned above except the stratification variables.

^b^Data were listed as the weighted odd ratio estimates and 95% confidence intervals, with *p < 0.05, **p < 0.01, ***p < 0.001.

^c^Q, quintile.

^d^Ref, reference.

^e^Tests for trends based on the variables containing the median values for each quartile.

^f^*P*_test_ was the result of Bonfreni correction.

Supplementary Table 17. Association of TyG-WC with congestive heart failure stratified by age, sex, race, exercise, smoking, drinking, and T2DM status

|  |  |  | TyG-WC | | | | | | | *P*_trend_^e^ | *P*_test_^f^ |
| --- | --- | --- | --- | --- | --- | --- | --- | --- | --- | --- | --- |
|  |  |  | Q1^c^ | Q2 | | Q3 | | Q4 | |  |  |
|  |  |  |  | HR/OR^b^ | 95% CI | HR/OR | 95% CI | HR/OR | 95% CI |  |  |
| Age |  |  |  |  |  |  |  |  |  |  |  |
|  | Model 1^a^ | > 50 | Ref (1.00)^d^ | 0.86 | (0.43,1.71) | 0.94 | (0.56,1.60) | 2.43 | **(1.36,4.35)**** | < 0.001 | 0.010 |
|  |  | ≤ 50 | Ref (1.00) | 1.13 | (0.38,3.41) | 1.89 | (0.41,8.65) | 4.95 | **(1.88,13.06)**** | 0.001 | 0.004 |
|  | Model 2 | > 50 | Ref (1.00) | 0.82 | (0.42,1.62) | 0.86 | (0.52,1.45) | 2.14 | **(1.20,3.81)*** | < 0.001 | 0.032 |
|  |  | ≤ 50 | Ref (1.00) | 1.00 | (0.34,2.90) | 1.57 | (0.32,7.76) | 3.68 | **(1.48,9.15)**** | 0.003 | 0.017 |
|  | Model 3 | > 50 | Ref (1.00) | 0.83 | (0.42,1.63) | 0.87 | (0.52,1.45) | 2.20 | **(1.23,3.95)**** | < 0.001 | 0.025 |
|  |  | ≤ 50 | Ref (1.00) | 0.97 | (0.33,2.87) | 1.54 | (0.31,7.67) | 3.92 | **(1.51,10.17)**** | 0.003 | 0.016 |
|  | Model 4 | > 50 | Ref (1.00) | 0.81 | (0.41,1.62) | 0.78 | (0.47,1.30) | 1.78 | (0.98,3.24) | 0.003 | 0.175 |
|  |  | ≤ 50 | Ref (1.00) | 0.88 | (0.28,2.79) | 1.19 | (0.20,7.13) | 2.49 | (0.80,7.80) | 0.072 | 0.346 |
| Sex |  |  |  |  |  |  |  |  |  |  |  |
|  | Model l | Male | Ref (1.00) | 1.20 | (0.51,2.81) | 1.13 | (0.56,2.26) | 3.17 | **(1.53,6.59)**** | < 0.001 | 0.007 |
|  |  | Female | Ref (1.00) | 0.66 | (0.32,1.36) | 0.92 | (0.53,1.60) | 2.82 | **(1.45,5.50)**** | 0.001 | 0.008 |
|  | Model 2 | Male | Ref (1.00) | 1.25 | (0.54,2.89) | 1.12 | (0.55,2.26) | 3.14 | **(1.52,6.50)**** | < 0.001 | 0.007 |
|  |  | Female | Ref (1.00) | 0.63 | (0.31,1.30) | 0.85 | (0.49,1.47) | 2.43 | **(1.31,4.52)**** | 0.002 | 0.016 |
|  | Model 3 | Male | Ref (1.00) | 1.26 | (0.55,2.90) | 1.11 | (0.55,2.25) | 3.08 | **(1.47,6.44)**** | < 0.001 | 0.009 |
|  |  | Female | Ref (1.00) | 0.63 | (0.31,1.29) | 0.84 | (0.48,1.46) | 2.48 | **(1.37,4.49)**** | 0.001 | 0.010 |
|  | Model 4 | Male | Ref (1.00) | 1.30 | (0.56,3.02) | 1.08 | (0.53,2.20) | 2.78 | **(1.31,5.91)**** | 0.001 | 0.025 |
|  |  | Female | Ref (1.00) | 0.63 | (0.31,1.30) | 0.73 | (0.40,1.31) | 1.91 | **(1.03,3.51)*** | 0.018 | 0.116 |
| Race |  |  |  |  |  |  |  |  |  |  |  |
|  | Model l | Non-hispanic white | Ref (1.00) | 0.77 | (0.38,1.55) | 0.84 | (0.53,1.31) | 2.43 | **(1.34,4.38)**** | < 0.001 | 0.011 |
|  |  | Others | Ref (1.00) | 1.15 | (0.52,2.51) | 1.28 | (0.64,2.58) | 3.80 | **(1.79,8.04)***** | < 0.001 | 0.002 |
|  | Model 2 | Non-hispanic white | Ref (1.00) | 0.77 | (0.39,1.53) | 0.78 | (0.50,1.21) | 2.22 | **(1.25,3.95)**** | < 0.001 | 0.022 |
|  |  | Others | Ref (1.00) | 1.13 | (0.51,2.49) | 1.23 | (0.61,2.48) | 3.45 | **(1.64,7.25)**** | 0.001 | 0.004 |
|  | Model 3 | Non-hispanic white | Ref (1.00) | 0.77 | (0.39,1.52) | 0.77 | (0.49,1.19) | 2.16 | **(1.23,3.81)**** | < 0.001 | 0.025 |
|  |  | Others | Ref (1.00) | 1.16 | (0.53,2.55) | 1.26 | (0.63,2.54) | 3.55 | **(1.67,7.56)**** | 0.001 | 0.004 |
|  | Model 4 | Non-hispanic white | Ref (1.00) | 0.78 | (0.40,1.55) | 0.72 | (0.46,1.13) | 1.83 | **(1.04,3.24)*** | 0.003 | 0.112 |
|  |  | Others | Ref (1.00) | 1.17 | (0.52,2.63) | 1.15 | (0.56,2.37) | 2.95 | **(1.37,6.37)**** | 0.003 | 0.019 |
| Exercise |  |  |  |  |  |  |  |  |  |  |  |
|  | Model l | Yes | Ref (1.00) | 1.04 | (0.57,1.88) | 0.89 | (0.52,1.53) | 2.50 | **(1.51,4.16)**** | < 0.001 | 0.002 |
|  |  | No | Ref (1.00) | 0.68 | (0.25,1.84) | 1.01 | (0.56,1.83) | 2.88 | **(1.35,6.12)**** | 0.001 | 0.020 |
|  | Model 2 | Yes | Ref (1.00) | 1.06 | (0.58,1.93) | 0.91 | (0.53,1.57) | 2.55 | **(1.53,4.24)***** | < 0.001 | 0.001 |
|  |  | No | Ref (1.00) | 0.66 | (0.25,1.74) | 0.92 | (0.54,1.56) | 2.54 | **(1.20,5.37)*** | 0.003 | 0.045 |
|  | Model 3 | Yes | Ref (1.00) | 1.06 | (0.59,1.93) | 0.91 | (0.52,1.57) | 2.57 | **(1.54,4.29)***** | < 0.001 | 0.001 |
|  |  | No | Ref (1.00) | 0.66 | (0.26,1.70) | 0.91 | (0.53,1.55) | 2.48 | **(1.22,5.07)*** | 0.003 | 0.039 |
|  | Model 4 | Yes | Ref (1.00) | 1.08 | (0.60,1.94) | 0.80 | (0.47,1.37) | 2.01 | **(1.19,3.39)**** | 0.001 | 0.027 |
|  |  | No | Ref (1.00) | 0.70 | (0.27,1.77) | 0.91 | (0.54,1.53) | 2.42 | **(1.22,4.82)*** | 0.003 | 0.037 |
| Smoking |  |  |  |  |  |  |  |  |  |  |  |
|  | Model l | Yes | Ref (1.00) | 0.80 | (0.32,1.96) | 0.49 | **(0.27,0.91)*** | 2.08 | (1.00,4.31) | 0.015 | 0.074 |
|  |  | No | Ref (1.00) | 0.94 | (0.44,2.01) | 1.23 | (0.69,2.18) | 3.28 | **(1.78,6.03)***** | < 0.001 | 0.001 |
|  | Model 2 | Yes | Ref (1.00) | 0.79 | (0.34,1.86) | 0.51 | (0.26,1.01) | 2.09 | **(1.06,4.13)*** | 0.010 | 0.103 |
|  |  | No | Ref (1.00) | 0.93 | (0.44,1.97) | 1.17 | (0.67,2.04) | 3.08 | **(1.68,5.65)***** | < 0.001 | 0.001 |
|  | Model 3 | Yes | Ref (1.00) | 0.79 | (0.33,1.89) | 0.51 | **(0.27,0.99)*** | 2.11 | **(1.05,4.24)*** | 0.012 | 0.106 |
|  |  | No | Ref (1.00) | 0.93 | (0.44,1.95) | 1.16 | (0.66,2.03) | 3.06 | **(1.67,5.62)***** | < 0.001 | 0.001 |
|  | Model 4 | Yes | Ref (1.00) | 0.86 | (0.37,1.99) | 0.52 | (0.24,1.10) | 2.02 | **(1.01,4.04)*** | 0.022 | 0.145 |
|  |  | No | Ref (1.00) | 0.92 | (0.44,1.93) | 1.04 | (0.59,1.82) | 2.44 | **(1.30,4.61)**** | < 0.001 | 0.019 |
| Drinking |  |  |  |  |  |  |  |  |  |  |  |
|  | Model l | Yes | Ref (1.00) | 0.98 | (0.46,2.09) | 0.97 | (0.56,1.67) | 3.15 | **(1.68,5.90)***** | < 0.001 | 0.001 |
|  |  | No | Ref (1.00) | 0.67 | (0.30,1.45) | 0.92 | (0.45,1.89) | 2.17 | **(1.16,4.03)*** | 0.002 | 0.046 |
|  | Model 2 | Yes | Ref (1.00) | 1.00 | (0.48,2.07) | 0.97 | (0.58,1.64) | 2.98 | **(1.64,5.43)***** | < 0.001 | 0.001 |
|  |  | No | Ref (1.00) | 0.66 | (0.30,1.46) | 0.89 | (0.43,1.84) | 2.06 | **(1.10,3.87)*** | 0.004 | 0.073 |
|  | Model 3 | Yes | Ref (1.00) | 0.99 | (0.48,2.05) | 0.97 | (0.57,1.63) | 3.00 | **(1.64,5.47)***** | < 0.001 | 0.001 |
|  |  | No | Ref (1.00) | 0.66 | (0.30,1.46) | 0.89 | (0.43,1.82) | 2.01 | **(1.08,3.75)*** | 0.004 | 0.084 |
|  | Model 4 | Yes | Ref (1.00) | 1.03 | (0.50,2.15) | 0.90 | (0.53,1.51) | 2.48 | **(1.36,4.53)**** | < 0.001 | 0.011 |
|  |  | No | Ref (1.00) | 0.62 | (0.28,1.38) | 0.82 | (0.40,1.67) | 1.67 | (0.85,3.28) | 0.032 | 0.400 |
| T2DM |  |  |  |  |  |  |  |  |  |  |  |
|  | Model l | Yes | Ref (1.00) | 1.13 | (0.30,4.23) | 0.75 | (0.21,2.69) | 2.70 | (0.86,8.43) | < 0.001 | 0.262 |
|  |  | No | Ref (1.00) | 0.79 | (0.43,1.46) | 0.96 | (0.63,1.44) | 2.29 | **(1.30,4.05)**** | 0.001 | 0.014 |
|  | Model 2 | Yes | Ref (1.00) | 1.17 | (0.30,4.58) | 0.75 | (0.21,2.77) | 2.64 | (0.82,8.45) | 0.001 | 0.304 |
|  |  | No | Ref (1.00) | 0.78 | (0.42,1.43) | 0.91 | (0.61,1.36) | 2.14 | **(1.21,3.78)**** | 0.001 | 0.028 |
|  | Model 3 | Yes | Ref (1.00) | 1.29 | (0.34,4.83) | 0.83 | (0.23,2.92) | 2.82 | (0.90,8.78) | 0.001 | 0.221 |
|  |  | No | Ref (1.00) | 0.78 | (0.42,1.42) | 0.91 | (0.61,1.36) | 2.16 | **(1.22,3.81)**** | 0.001 | 0.025 |
|  | Model 4 | Yes | Ref (1.00) | 1.33 | (0.36,4.91) | 0.82 | (0.23,2.90) | 2.78 | (0.90,8.58) | 0.001 | 0.222 |
|  |  | No | Ref (1.00) | 0.83 | (0.45,1.52) | 0.97 | (0.64,1.46) | 2.36 | **(1.36,4.10)**** | < 0.001 | 0.008 |

^a^The variables adjusted in each model were the factors mentioned above except the stratification variables.

^b^Data were listed as the weighted odd ratio estimates and 95% confidence intervals, with *p < 0.05, **p < 0.01, ***p < 0.001.

^c^Q, quintile.

^d^Ref, reference.

^e^Tests for trends based on the variables containing the median values for each quartile.

^f^*P*_test_ was the result of Bonfreni correction.

Supplementary Table 18. Association of TyG-WtHR with congestive heart failure stratified by age, sex, race, exercise, smoking, drinking, and T2DM status

|  |  |  | TyG-WtHR | | | | | | | *P*_trend_^e^ | *P*_test_^f^ |
| --- | --- | --- | --- | --- | --- | --- | --- | --- | --- | --- | --- |
|  |  |  | Q1^c^ | Q2 | | Q3 | | Q4 | |  |  |
|  |  |  |  | HR/OR^b^ | 95% CI | HR/OR | 95% CI | HR/OR | 95% CI |  |  |
| Age |  |  |  |  |  |  |  |  |  |  |  |
|  | Model 1^a^ | > 50 | Ref (1.00)^d^ | 0.82 | (0.44,1.52) | 1.33 | (0.74,2.36) | 2.46 | **(1.33,4.53)**** | < 0.001 | 0.013 |
|  |  | ≤ 50 | Ref (1.00) | 1.23 | (0.39,3.90) | 1.17 | (0.34,3.99) | 5.33 | **(2.25,12.63)**** | 0.001 | 0.001 |
|  | Model 2 | > 50 | Ref (1.00) | 0.77 | (0.42,1.41) | 1.19 | (0.67,2.11) | 2.07 | **(1.13,3.80)*** | < 0.001 | 0.059 |
|  |  | ≤ 50 | Ref (1.00) | 1.08 | (0.33,3.51) | 0.96 | (0.27,3.46) | 3.85 | **(1.69,8.77)**** | 0.003 | 0.005 |
|  | Model 3 | > 50 | Ref (1.00) | 0.75 | (0.41,1.37) | 1.15 | (0.65,2.04) | 2.03 | **(1.10,3.73)*** | 0.001 | 0.071 |
|  |  | ≤ 50 | Ref (1.00) | 0.99 | (0.31,3.21) | 0.94 | (0.25,3.46) | 3.86 | **(1.68,8.84)**** | 0.002 | 0.005 |
|  | Model 4 | > 50 | Ref (1.00) | 0.76 | (0.41,1.39) | 1.06 | (0.60,1.89) | 1.68 | (0.90,3.17) | 0.010 | 0.313 |
|  |  | ≤ 50 | Ref (1.00) | 0.91 | (0.25,3.34) | 0.79 | (0.20,3.21) | 2.41 | (0.89,6.51) | 0.059 | 0.246 |
| Sex |  |  |  |  |  |  |  |  |  |  |  |
|  | Model l | Male | Ref (1.00) | 0.79 | (0.42,1.48) | 1.46 | (0.83,2.58) | 2.78 | **(1.44,5.38)**** | < 0.001 | 0.008 |
|  |  | Female | Ref (1.00) | 0.85 | (0.43,1.68) | 0.84 | (0.42,1.66) | 2.36 | **(1.12,4.97)*** | 0.007 | 0.072 |
|  | Model 2 | Male | Ref (1.00) | 0.78 | (0.42,1.45) | 1.37 | (0.77,2.45) | 2.59 | **(1.33,5.05)**** | < 0.001 | 0.017 |
|  |  | Female | Ref (1.00) | 0.78 | (0.40,1.54) | 0.75 | (0.38,1.47) | 1.95 | (0.97,3.93) | 0.015 | 0.183 |
|  | Model 3 | Male | Ref (1.00) | 0.76 | (0.40,1.43) | 1.33 | (0.75,2.37) | 2.47 | **(1.26,4.85)**** | 0.001 | 0.027 |
|  |  | Female | Ref (1.00) | 0.77 | (0.39,1.52) | 0.73 | (0.37,1.43) | 1.93 | (0.97,3.81) | 0.014 | 0.179 |
|  | Model 4 | Male | Ref (1.00) | 0.78 | (0.42,1.45) | 1.26 | (0.71,2.24) | 2.22 | **(1.13,4.33)*** | 0.003 | 0.061 |
|  |  | Female | Ref (1.00) | 0.80 | (0.39,1.63) | 0.68 | (0.34,1.36) | 1.52 | (0.74,3.10) | 0.089 | 0.743 |
| Race |  |  |  |  |  |  |  |  |  |  |  |
|  | Model l | Non-hispanic white | Ref (1.00) | 0.71 | (0.37,1.35) | 1.05 | (0.62,1.77) | 2.23 | **(1.20,4.13)*** | 0.001 | 0.035 |
|  |  | Others | Ref (1.00) | 1.11 | (0.51,2.41) | 1.27 | (0.64,2.53) | 3.15 | **(1.50,6.60)**** | 0.001 | 0.008 |
|  | Model 2 | Non-hispanic white | Ref (1.00) | 0.68 | (0.36,1.29) | 0.97 | (0.57,1.65) | 1.95 | **(1.05,3.60)*** | 0.002 | 0.104 |
|  |  | Others | Ref (1.00) | 1.06 | (0.48,2.34) | 1.18 | (0.58,2.39) | 2.79 | **(1.33,5.84)**** | 0.003 | 0.021 |
|  | Model 3 | Non-hispanic white | Ref (1.00) | 0.67 | (0.35,1.26) | 0.93 | (0.55,1.58) | 1.86 | **(1.01,3.43)*** | 0.003 | 0.137 |
|  |  | Others | Ref (1.00) | 1.04 | (0.47,2.32) | 1.20 | (0.59,2.43) | 2.78 | **(1.33,5.84)**** | 0.003 | 0.022 |
|  | Model 4 | Non-hispanic white | Ref (1.00) | 0.70 | (0.37,1.33) | 0.89 | (0.52,1.53) | 1.63 | (0.86,3.06) | 0.020 | 0.392 |
|  |  | Others | Ref (1.00) | 1.09 | (0.48,2.45) | 1.18 | (0.57,2.43) | 2.32 | **(1.08,4.98)*** | 0.013 | 0.092 |
| Exercise |  |  |  |  |  |  |  |  |  |  |  |
|  | Model l | Yes | Ref (1.00) | 1.05 | (0.52,2.12) | 1.06 | (0.60,1.86) | 2.43 | **(1.24,4.73)*** | < 0.001 | 0.035 |
|  |  | No | Ref (1.00) | 0.55 | (0.22,1.34) | 1.10 | (0.54,2.23) | 2.30 | **(1.08,4.91)*** | 0.008 | 0.093 |
|  | Model 2 | Yes | Ref (1.00) | 1.06 | (0.52,2.15) | 1.07 | (0.61,1.90) | 2.42 | **(1.24,4.75)*** | < 0.001 | 0.032 |
|  |  | No | Ref (1.00) | 0.52 | (0.22,1.22) | 0.96 | (0.48,1.91) | 1.97 | (0.93,4.15) | 0.023 | 0.229 |
|  | Model 3 | Yes | Ref (1.00) | 1.06 | (0.53,2.15) | 1.06 | (0.60,1.86) | 2.41 | **(1.22,4.75)*** | < 0.001 | 0.035 |
|  |  | No | Ref (1.00) | 0.50 | (0.22,1.15) | 0.93 | (0.47,1.82) | 1.82 | (0.88,3.77) | 0.029 | 0.309 |
|  | Model 4 | Yes | Ref (1.00) | 1.07 | (0.53,2.19) | 0.95 | (0.53,1.68) | 1.92 | (0.94,3.91) | 0.011 | 0.218 |
|  |  | No | Ref (1.00) | 0.53 | (0.23,1.20) | 0.93 | (0.47,1.80) | 1.78 | (0.88,3.58) | 0.036 | 0.319 |
| Smoking |  |  |  |  |  |  |  |  |  |  |  |
|  | Model l | Yes | Ref (1.00) | 0.87 | (0.41,1.86) | 0.89 | (0.41,1.94) | 2.18 | (0.90,5.31) | 0.06 | 0.254 |
|  |  | No | Ref (1.00) | 0.84 | (0.41,1.73) | 1.34 | (0.71,2.54) | 2.89 | **(1.52,5.48)**** | < 0.001 | 0.004 |
|  | Model 2 | Yes | Ref (1.00) | 0.88 | (0.42,1.84) | 0.91 | (0.42,1.98) | 2.16 | (0.93,5.02) | 0.055 | 0.222 |
|  |  | No | Ref (1.00) | 0.81 | (0.40,1.65) | 1.26 | (0.67,2.36) | 2.63 | **(1.39,4.97)**** | < 0.001 | 0.100 |
|  | Model 3 | Yes | Ref (1.00) | 0.87 | (0.41,1.86) | 0.90 | (0.41,1.98) | 2.15 | (0.90,5.10) | 0.063 | 0.247 |
|  |  | No | Ref (1.00) | 0.80 | (0.40,1.59) | 1.22 | (0.65,2.29) | 2.54 | **(1.34,4.82)**** | < 0.001 | 0.140 |
|  | Model 4 | Yes | Ref (1.00) | 0.96 | (0.46,2.01) | 0.90 | (0.42,1.93) | 2.01 | (0.84,4.81) | 0.099 | 0.350 |
|  |  | No | Ref (1.00) | 0.80 | (0.40,1.62) | 1.13 | (0.58,2.19) | 2.09 | **(1.07,4.08)*** | 0.001 | 0.094 |
| Drinking |  |  |  |  |  |  |  |  |  |  |  |
|  | Model l | Yes | Ref (1.00) | 0.78 | (0.40,1.52) | 1.15 | (0.63,2.08) | 2.63 | **(1.41,4.90)**** | < 0.001 | 0.008 |
|  |  | No | Ref (1.00) | 0.86 | (0.37,2.01) | 1.14 | (0.43,2.98) | 2.43 | **(1.13,5.25)*** | 0.001 | 0.072 |
|  | Model 2 | Yes | Ref (1.00) | 0.78 | (0.41,1.49) | 1.12 | (0.62,2.02) | 2.40 | **(1.31,4.41)**** | < 0.001 | 0.016 |
|  |  | No | Ref (1.00) | 0.92 | (0.37,2.28) | 1.18 | (0.43,3.30) | 2.47 | **(1.08,5.67)*** | 0.001 | 0.100 |
|  | Model 3 | Yes | Ref (1.00) | 0.77 | (0.40,1.47) | 1.10 | (0.61,1.98) | 2.35 | **(1.28,4.33)**** | < 0.001 | 0.019 |
|  |  | No | Ref (1.00) | 0.88 | (0.35,2.22) | 1.13 | (0.40,3.19) | 2.32 | (0.99,5.47) | 0.002 | 0.016 |
|  | Model 4 | Yes | Ref (1.00) | 0.81 | (0.42,1.54) | 1.03 | (0.58,1.83) | 1.94 | **(1.06,3.57)*** | 0.005 | 0.097 |
|  |  | No | Ref (1.00) | 0.85 | (0.33,2.20) | 1.09 | (0.37,3.20) | 2.03 | (0.79,5.26) | 0.016 | 0.424 |
| T2DM |  |  |  |  |  |  |  |  |  |  |  |
|  | Model l | Yes | Ref (1.00) | 0.66 | (0.17,2.50) | 0.95 | (0.26,3.44) | 1.84 | (0.58,5.83) | 0.009 | 0.894 |
|  |  | No | Ref (1.00) | 0.79 | (0.47,1.32) | 1.07 | (0.66,1.74) | 2.16 | **(1.21,3.84)**** | 0.002 | 0.028 |
|  | Model 2 | Yes | Ref (1.00) | 0.67 | (0.17,2.60) | 0.92 | (0.25,3.40) | 1.75 | (0.54,5.69) | 0.015 | 1.000 |
|  |  | No | Ref (1.00) | 0.76 | (0.46,1.26) | 1.00 | (0.61,1.64) | 1.93 | **(1.08,3.45)*** | 0.006 | 0.081 |
|  | Model 3 | Yes | Ref (1.00) | 0.73 | (0.20,2.66) | 0.96 | (0.27,3.39) | 1.82 | (0.59,5.62) | 0.016 | 0.888 |
|  |  | No | Ref (1.00) | 0.75 | (0.45,1.24) | 0.99 | (0.60,1.62) | 1.91 | **(1.07,3.43)*** | 0.007 | 0.089 |
|  | Model 4 | Yes | Ref (1.00) | 0.75 | (0.20,2.71) | 0.95 | (0.26,3.40) | 1.82 | (0.59,5.60) | 0.016 | 0.882 |
|  |  | No | Ref (1.00) | 0.81 | (0.49,1.36) | 1.09 | (0.66,1.80) | 2.14 | **(1.20,3.79)*** | 0.002 | 0.030 |

^a^The variables adjusted in each model were the factors mentioned above except the stratification variables.

^b^Data were listed as the weighted odd ratio estimates and 95% confidence intervals, with *p < 0.05, **p < 0.01, ***p < 0.001.

^c^Q, quintile.

^d^Ref, reference.

^e^Tests for trends based on the variables containing the median values for each quartile.

^f^*P*_test_ was the result of Bonfreni correction.

Supplementary Table 19. Association of TyG-BMI with congestive heart failure stratified by age, sex, race, exercise, smoking, drinking, and T2DM status

|  |  |  | TyG-BMI | | | | | | | *P*_trend_^e^ | *P*_test_^f^ |
| --- | --- | --- | --- | --- | --- | --- | --- | --- | --- | --- | --- |
|  |  |  | Q1^c^ | Q2 | | Q3 | | Q4 | |  |  |
|  |  |  |  | HR/OR^b^ | 95% CI | HR/OR | 95% CI | HR/OR | 95% CI |  |  |
| Age |  |  |  |  |  |  |  |  |  |  |  |
|  | Model 1^a^ | > 50 | Ref (1.00)^d^ | 0.76 | (0.46,1.27) | 1.23 | (0.73,2.10) | 1.83 | **(1.13,2.98)*** | < 0.001 | 0.045 |
|  |  | ≤ 50 | Ref (1.00) | 1.87 | (0.53,6.60) | 0.31 | (0.06,1.54) | 3.89 | **(1.60,9.43)**** | 0.013 | 0.009 |
|  | Model 2 | > 50 | Ref (1.00) | 0.73 | (0.44,1.21) | 1.17 | (0.69,1.97) | 1.64 | **(1.01,2.65)*** | 0.002 | 0.139 |
|  |  | ≤ 50 | Ref (1.00) | 1.73 | (0.50,6.02) | 0.26 | (0.05,1.24) | 2.96 | **(1.28,6.85)*** | 0.036 | 0.035 |
|  | Model 3 | > 50 | Ref (1.00) | 0.72 | (0.43,1.20) | 1.16 | (0.68,1.97) | 1.68 | **(1.02,2.75)*** | 0.002 | 0.122 |
|  |  | ≤ 50 | Ref (1.00) | 1.64 | (0.47,5.77) | 0.25 | (0.05,1.22) | 3.13 | **(1.30,7.51)*** | 0.027 | 0.034 |
|  | Model 4 | > 50 | Ref (1.00) | 0.72 | (0.43,1.20) | 1.09 | (0.65,1.84) | 1.38 | (0.82,2.33) | 0.038 | 0.608 |
|  |  | ≤ 50 | Ref (1.00) | 1.49 | (0.39,5.72) | 0.20 | (0.04,1.04) | 1.87 | (0.71,4.96) | 0.268 | 0.166 |
| Sex |  |  |  |  |  |  |  |  |  |  |  |
|  | Model l | Male | Ref (1.00) | 1.09 | (0.59,2.01) | 1.21 | (0.70,2.08) | 3.18 | **(1.73,5.86)***** | < 0.001 | 0.001 |
|  |  | Female | Ref (1.00) | 0.71 | (0.41,1.20) | 1.37 | (0.67,2.80) | 2.07 | **(1.11,3.84)*** | 0.009 | 0.065 |
|  | Model 2 | Male | Ref (1.00) | 1.08 | (0.58,2.00) | 1.17 | (0.68,2.01) | 3.03 | **(1.62,5.66)**** | < 0.001 | 0.002 |
|  |  | Female | Ref (1.00) | 0.65 | (0.38,1.12) | 1.23 | (0.61,2.47) | 1.76 | (0.98,3.15) | 0.020 | 0.176 |
|  | Model 3 | Male | Ref (1.00) | 1.07 | (0.59,1.97) | 1.15 | (0.67,1.97) | 2.97 | **(1.59,5.55)**** | < 0.001 | 0.002 |
|  |  | Female | Ref (1.00) | 0.64 | (0.36,1.12) | 1.19 | (0.60,2.36) | 1.76 | (1.00,3.09) | 0.016 | 0.152 |
|  | Model 4 | Male | Ref (1.00) | 1.05 | (0.57,1.92) | 1.08 | (0.63,1.82) | 2.58 | **(1.39,4.82)**** | 0.002 | 0.100 |
|  |  | Female | Ref (1.00) | 0.60 | (0.32,1.12) | 1.04 | (0.53,2.05) | 1.30 | (0.71,2.39) | 0.151 | 0.319 |
| Race |  |  |  |  |  |  |  |  |  |  |  |
|  | Model l | Non-hispanic white | Ref (1.00) | 0.78 | (0.48,1.28) | 1.10 | (0.64,1.90) | 2.25 | **(1.31,3.88)**** | 0.001 | 0.011 |
|  |  | Others | Ref (1.00) | 1.32 | (0.64,2.73) | 1.62 | (0.84,3.12) | 3.44 | **(1.75,6.75)***** | < 0.001 | 0.001 |
|  | Model 2 | Non-hispanic white | Ref (1.00) | 0.75 | (0.46,1.21) | 1.02 | (0.59,1.74) | 2.02 | **(1.17,3.49)**** | 0.003 | 0.037 |
|  |  | Others | Ref (1.00) | 1.27 | (0.60,2.69) | 1.55 | (0.80,2.98) | 3.16 | **(1.62,6.19)**** | 0.001 | 0.003 |
|  | Model 3 | Non-hispanic white | Ref (1.00) | 0.74 | (0.45,1.19) | 0.99 | (0.58,1.69) | 1.97 | **(1.15,3.38)**** | 0.003 | 0.042 |
|  |  | Others | Ref (1.00) | 1.27 | (0.60,2.69) | 1.59 | (0.83,3.06) | 3.15 | **(1.61,6.16)**** | 0.001 | 0.003 |
|  | Model 4 | Non-hispanic white | Ref (1.00) | 0.73 | (0.44,1.21) | 0.92 | (0.55,1.54) | 1.64 | (0.93,2.88) | 0.025 | 0.255 |
|  |  | Others | Ref (1.00) | 1.22 | (0.57,2.64) | 1.44 | (0.73,2.87) | 2.54 | **(1.30,4.96)**** | 0.004 | 0.021 |
| Exercise |  |  |  |  |  |  |  |  |  |  |  |
|  | Model l | Yes | Ref (1.00) | 0.70 | (0.42,1.17) | 1.24 | (0.77,2.01) | 1.95 | **(1.17,3.24)*** | < 0.001 | 0.033 |
|  |  | No | Ref (1.00) | 1.21 | (0.56,2.63) | 1.09 | (0.46,2.56) | 3.17 | **(1.36,7.43)**** | 0.010 | 0.025 |
|  | Model 2 | Yes | Ref (1.00) | 0.71 | (0.42,1.20) | 1.26 | (0.79,2.01) | 1.97 | **(1.18,3.29)*** | < 0.001 | 0.029 |
|  |  | No | Ref (1.00) | 1.13 | (0.54,2.37) | 0.98 | (0.42,2.28) | 2.72 | **(1.15,6.42)*** | 0.028 | 0.070 |
|  | Model 3 | Yes | Ref (1.00) | 0.71 | (0.42,1.19) | 1.26 | (0.79,2.03) | 1.99 | **(1.19,3.33)**** | < 0.001 | 0.027 |
|  |  | No | Ref (1.00) | 1.09 | (0.52,2.30) | 0.93 | (0.40,2.16) | 2.56 | **(1.10,5.94)*** | 0.031 | 0.087 |
|  | Model 4 | Yes | Ref (1.00) | 0.66 | (0.39,1.11) | 1.14 | (0.72,1.81) | 1.48 | (0.87,2.52) | 0.017 | 0.354 |
|  |  | No | Ref (1.00) | 1.12 | (0.55,2.28) | 0.89 | (0.39,2.05) | 2.41 | **(1.07,5.43)*** | 0.048 | 0.100 |
| Smoking |  |  |  |  |  |  |  |  |  |  |  |
|  | Model l | Yes | Ref (1.00) | 0.43 | (0.19,0.95) | 0.85 | (0.40,1.83) | 1.54 | (0.78,3.07) | 0.136 | 0.110 |
|  |  | No | Ref (1.00) | 1.30 | (0.68,2.51) | 1.65 | (0.86,3.15) | 3.58 | **(1.96,6.55)***** | < 0.001 | < 0.001 |
|  | Model 2 | Yes | Ref (1.00) | 0.44 | (0.19,1.00) | 0.88 | (0.41,1.89) | 1.55 | (0.79,3.06) | 0.126 | 0.148 |
|  |  | No | Ref (1.00) | 1.27 | (0.66,2.42) | 1.58 | (0.83,2.99) | 3.31 | **(1.80,6.09)***** | < 0.001 | 0.001 |
|  | Model 3 | Yes | Ref (1.00) | 0.43 | (0.19,0.97) | 0.88 | (0.40,1.93) | 1.54 | (0.76,3.11) | 0.138 | 0.123 |
|  |  | No | Ref (1.00) | 1.24 | (0.65,2.38) | 1.54 | (0.81,2.93) | 3.25 | **(1.77,5.99)***** | < 0.001 | 0.001 |
|  | Model 4 | Yes | Ref (1.00) | 0.45 | (0.20,1.03) | 0.87 | (0.40,1.87) | 1.38 | (0.67,2.85) | 0.224 | 0.180 |
|  |  | No | Ref (1.00) | 1.21 | (0.62,2.35) | 1.40 | (0.72,2.72) | 2.59 | **(1.36,4.92)**** | < 0.001 | 0.012 |
| Drinking |  |  |  |  |  |  |  |  |  |  |  |
|  | Model l | Yes | Ref (1.00) | 0.89 | (0.49,1.63) | 1.03 | (0.57,1.87) | 2.85 | **(1.58,5.14)**** | < 0.001 | 0.002 |
|  |  | No | Ref (1.00) | 0.88 | (0.42,1.87) | 1.74 | (0.88,3.44) | 1.82 | (0.89,3.73) | 0.026 | 0.303 |
|  | Model 2 | Yes | Ref (1.00) | 0.91 | (0.50,1.65) | 1.04 | (0.58,1.86) | 2.73 | **(1.56,4.77)**** | < 0.001 | 0.002 |
|  |  | No | Ref (1.00) | 0.93 | (0.43,2.02) | 1.78 | (0.88,3.63) | 1.85 | (0.89,3.82) | 0.024 | 0.293 |
|  | Model 3 | Yes | Ref (1.00) | 0.90 | (0.50,1.62) | 1.02 | (0.57,1.82) | 2.71 | **(1.55,4.73)**** | < 0.001 | 0.002 |
|  |  | No | Ref (1.00) | 0.90 | (0.41,1.99) | 1.73 | (0.83,3.58) | 1.77 | (0.85,3.67) | 0.031 | 0.377 |
|  | Model 4 | Yes | Ref (1.00) | 0.88 | (0.48,1.60) | 0.94 | (0.53,1.67) | 2.16 | **(1.23,3.81)**** | 0.002 | 0.024 |
|  |  | No | Ref (1.00) | 0.88 | (0.39,2.00) | 1.61 | (0.76,3.42) | 1.47 | (0.66,3.28) | 0.149 | 0.638 |
| T2DM |  |  |  |  |  |  |  |  |  |  |  |
|  | Model l | Yes | Ref (1.00) | 0.48 | (0.16,1.42) | 1.38 | (0.54,3.51) | 1.90 | (0.77,4.72) | 0.006 | 0.490 |
|  |  | No | Ref (1.00) | 0.95 | (0.61,1.46) | 1.03 | (0.61,1.76) | 2.19 | **(1.27,3.80)**** | 0.007 | 0.017 |
|  | Model 2 | Yes | Ref (1.00) | 0.47 | (0.16,1.38) | 1.34 | (0.52,3.41) | 1.77 | (0.71,4.39) | 0.012 | 0.495 |
|  |  | No | Ref (1.00) | 0.92 | (0.60,1.41) | 1.00 | (0.59,1.69) | 2.03 | **(1.17,3.52)*** | 0.013 | 0.038 |
|  | Model 3 | Yes | Ref (1.00) | 0.47 | (0.16,1.39) | 1.29 | (0.51,3.29) | 1.72 | (0.71,4.17) | 0.013 | 0.510 |
|  |  | No | Ref (1.00) | 0.91 | (0.59,1.40) | 0.99 | (0.58,1.68) | 2.04 | **(1.17,3.55)*** | 0.013 | 0.038 |
|  | Model 4 | Yes | Ref (1.00) | 0.46 | (0.15,1.36) | 1.22 | (0.48,3.14) | 1.64 | (0.68,3.95) | 0.016 | 0.469 |
|  |  | No | Ref (1.00) | 0.96 | (0.62,1.47) | 1.06 | (0.63,1.78) | 2.15 | **(1.26,3.68)**** | 0.007 | 0.017 |

^a^The variables adjusted in each model were the factors mentioned above except the stratification variables.

^b^Data were listed as the weighted odd ratio estimates and 95% confidence intervals, with *p < 0.05, **p < 0.01, ***p < 0.001.

^c^Q, quintile.

^d^Ref, reference.

^e^Tests for trends based on the variables containing the median values for each quartile.

^f^*P*_test_ was the result of Bonfreni correction.

Supplementary Table 20. Association of TyG with myocardial infarction stratified by age, sex, race, exercise, smoking, drinking, and T2DM status

|  |  |  | TyG | | | | | | | *P*_trend_^e^ | *P*_test_^f^ |
| --- | --- | --- | --- | --- | --- | --- | --- | --- | --- | --- | --- |
|  |  |  | Q1^c^ | Q2 | | Q3 | | Q4 | |  |  |
|  |  |  |  | HR/OR^b^ | 95% CI | HR/OR | 95% CI | HR/OR | 95% CI |  |  |
| Age |  |  |  |  |  |  |  |  |  |  |  |
|  | Model 1^a^ | > 50 | Ref (1.00)^d^ | 1.22 | (0.76,1.99) | 1.42 | (0.94,2.14) | 1.69 | **(1.15,2.49)**** | 0.003 | 0.025 |
|  |  | ≤ 50 | Ref (1.00) | 3.48 | **(1.16,10.46)*** | 2.57 | (0.80,8.29) | 6.23 | **(2.22,17.45)***** | 0.001 | 0.002 |
|  | Model 2 | > 50 | Ref (1.00) | 1.16 | (0.71,1.88) | 1.34 | (0.90,2.01) | 1.57 | **(1.06,2.32)*** | 0.010 | 0.075 |
|  |  | ≤ 50 | Ref (1.00) | 3.09 | **(1.06,9.05)*** | 2.26 | (0.70,7.31) | 5.10 | **(1.84,14.14)**** | 0.005 | 0.006 |
|  | Model 3 | > 50 | Ref (1.00) | 1.17 | (0.72,1.90) | 1.34 | (0.88,2.02) | 1.54 | **(1.04,2.28)*** | 0.014 | 0.100 |
|  |  | ≤ 50 | Ref (1.00) | 2.99 | **(1.03,8.64)*** | 2.12 | (0.65,6.88) | 4.98 | **(1.78,13.98)**** | 0.007 | 0.008 |
|  | Model 4 | > 50 | Ref (1.00) | 1.27 | (0.77,2.08) | 1.54 | **(1.01,2.33)*** | 1.88 | **(1.24,2.87)**** | 0.001 | 0.011 |
|  |  | ≤ 50 | Ref (1.00) | 3.34 | **(1.15,9.70)*** | 2.59 | (0.74,9.10) | 5.89 | **(2.00,17.36)**** | 0.003 | 0.005 |
| Sex |  |  |  |  |  |  |  |  |  |  |  |
|  | Model l | Male | Ref (1.00) | 0.99 | (0.59,1.69) | 1.46 | (0.83,2.55) | 2.04 | **(1.29,3.23)**** | < 0.001 | 0.008 |
|  |  | Female | Ref (1.00) | 2.04 | (0.99,4.22) | 1.63 | (0.88,3.01) | 1.87 | **(1.06,3.29)*** | 0.075 | 0.094 |
|  | Model 2 | Male | Ref (1.00) | 0.96 | (0.57,1.64) | 1.42 | (0.81,2.49) | 1.96 | **(1.23,3.12)**** | 0.001 | 0.015 |
|  |  | Female | Ref (1.00) | 1.91 | (0.94,3.91) | 1.46 | (0.77,2.78) | 1.63 | (0.89,2.97) | 0.306 | 0.226 |
|  | Model 3 | Male | Ref (1.00) | 0.94 | (0.55,1.61) | 1.35 | (0.76,2.39) | 1.85 | **(1.16,2.96)**** | 0.001 | 0.031 |
|  |  | Female | Ref (1.00) | 1.91 | (0.93,3.92) | 1.43 | (0.76,2.71) | 1.61 | (0.89,2.88) | 0.298 | 0.230 |
|  | Model 4 | Male | Ref (1.00) | 0.98 | (0.57,1.70) | 1.47 | (0.83,2.61) | 2.28 | **(1.41,3.69)***** | < 0.001 | 0.003 |
|  |  | Female | Ref (1.00) | 2.30 | **(1.10,4.83)*** | 1.78 | (0.93,3.40) | 1.91 | **(1.03,3.55)*** | 0.101 | 0.083 |
| Race |  |  |  |  |  |  |  |  |  |  |  |
|  | Model l | Non-hispanic white | Ref (1.00) | 1.46 | (0.85,2.52) | 1.55 | (0.96,2.52) | 1.98 | **(1.28,3.04)**** | 0.001 | 0.007 |
|  |  | Others | Ref (1.00) | 0.84 | (0.47,1.51) | 1.06 | (0.60,1.88) | 1.42 | (0.86,2.35) | 0.067 | 0.511 |
|  | Model 2 | Non-hispanic white | Ref (1.00) | 1.46 | (0.85,2.52) | 1.55 | (0.96,2.52) | 1.98 | **(1.28,3.04)**** | 0.005 | 0.026 |
|  |  | Others | Ref (1.00) | 0.82 | (0.45,1.48) | 1.02 | (0.58,1.81) | 1.34 | (0.82,2.21) | 0.096 | 0.722 |
|  | Model 3 | Non-hispanic white | Ref (1.00) | 1.37 | (0.78,2.39) | 1.38 | (0.84,2.28) | 1.71 | **(1.10,2.66)*** | 0.012 | 0.055 |
|  |  | Others | Ref (1.00) | 0.83 | (0.46,1.51) | 1.06 | (0.60,1.87) | 1.36 | (0.83,2.23) | 0.088 | 0.650 |
|  | Model 4 | Non-hispanic white | Ref (1.00) | 1.57 | (0.89,2.77) | 1.65 | (0.99,2.74) | 2.27 | **(1.39,3.69)***** | 0.001 | 0.004 |
|  |  | Others | Ref (1.00) | 0.87 | (0.48,1.59) | 1.13 | (0.62,2.06) | 1.41 | (0.84,2.37) | 0.075 | 0.557 |
| Exercise |  |  |  |  |  |  |  |  |  |  |  |
|  | Model l | Yes | Ref (1.00) | 1.13 | (0.65,1.97) | 1.33 | (0.85,2.08) | 1.70 | **(1.04,2.78)*** | 0.010 | 0.104 |
|  |  | No | Ref (1.00) | 1.46 | (0.77,2.77) | 1.51 | (0.81,2.81) | 1.77 | **(1.03,3.05)*** | 0.053 | 0.115 |
|  | Model 2 | Yes | Ref (1.00) | 1.14 | (0.65,1.99) | 1.33 | (0.85,2.07) | 1.72 | **(1.05,2.81)*** | 0.008 | 0.096 |
|  |  | No | Ref (1.00) | 1.44 | (0.75,2.76) | 1.45 | (0.78,2.72) | 1.63 | (0.93,2.88) | 0.124 | 0.264 |
|  | Model 3 | Yes | Ref (1.00) | 1.15 | (0.65,2.01) | 1.32 | (0.83,2.10) | 1.73 | **(1.06,2.83)*** | 0.008 | 0.090 |
|  |  | No | Ref (1.00) | 1.42 | (0.73,2.76) | 1.42 | (0.75,2.67) | 1.59 | (0.90,2.81) | 0.154 | 0.339 |
|  | Model 4 | Yes | Ref (1.00) | 1.24 | (0.70,2.18) | 1.46 | (0.90,2.38) | 1.85 | **(1.07,3.19)*** | 0.014 | 0.083 |
|  |  | No | Ref (1.00) | 1.69 | (0.86,3.29) | 1.82 | (0.96,3.47) | 2.50 | **(1.38,4.52)**** | 0.004 | 0.009 |
| Smoking |  |  |  |  |  |  |  |  |  |  |  |
|  | Model l | Yes | Ref (1.00) | 0.99 | (0.35,2.82) | 0.89 | (0.44,1.81) | 1.14 | (0.53,2.47) | 0.722 | 1.000 |
|  |  | No | Ref (1.00) | 1.44 | (0.87,2.40) | 1.62 | (0.99,2.66) | 2.10 | **(1.40,3.16)***** | < 0.001 | 0.001 |
|  | Model 2 | Yes | Ref (1.00) | 0.94 | (0.33,2.73) | 0.88 | (0.42,1.81) | 1.14 | (0.53,2.46) | 0.680 | 1.000 |
|  |  | No | Ref (1.00) | 1.41 | (0.84,2.37) | 1.58 | (0.96,2.60) | 2.00 | **(1.31,3.05)**** | 0.001 | 0.005 |
|  | Model 3 | Yes | Ref (1.00) | 0.97 | (0.33,2.85) | 0.89 | (0.43,1.85) | 1.20 | (0.56,2.56) | 0.617 | 1.000 |
|  |  | No | Ref (1.00) | 1.38 | (0.82,2.32) | 1.50 | (0.91,2.48) | 1.89 | **(1.24,2.87)**** | 0.002 | 0.011 |
|  | Model 4 | Yes | Ref (1.00) | 1.20 | (0.40,3.65) | 1.14 | (0.55,2.34) | 1.89 | (0.87,4.08) | 0.134 | 0.317 |
|  |  | No | Ref (1.00) | 1.49 | (0.89,2.51) | 1.70 | **(1.03,2.80)*** | 2.21 | **(1.38,3.53)***** | 0.001 | 0.003 |
| Drinking |  |  |  |  |  |  |  |  |  |  |  |
|  | Model l | Yes | Ref (1.00) | 1.19 | (0.71,1.99) | 1.34 | (0.86,2.09) | 1.90 | **(1.30,2.77)***** | < 0.001 | 0.003 |
|  |  | No | Ref (1.00) | 2.07 | (0.90,4.77) | 2.18 | **(1.02,4.67)*** | 2.30 | **(1.08,4.93)*** | 0.045 | 0.096 |
|  | Model 2 | Yes | Ref (1.00) | 1.13 | (0.68,1.90) | 1.24 | (0.79,1.94) | 1.72 | **(1.17,2.52)**** | 0.003 | 0.018 |
|  |  | No | Ref (1.00) | 1.99 | (0.86,4.57) | 1.93 | (0.90,4.12) | 2.06 | (0.97,4.36) | 0.115 | 0.181 |
|  | Model 3 | Yes | Ref (1.00) | 1.12 | (0.66,1.89) | 1.22 | (0.77,1.93) | 1.67 | **(1.14,2.47)*** | 0.004 | 0.029 |
|  |  | No | Ref (1.00) | 1.95 | (0.85,4.48) | 1.86 | (0.86,4.03) | 1.91 | (0.91,4.04) | 0.191 | 0.264 |
|  | Model 4 | Yes | Ref (1.00) | 1.27 | (0.74,2.18) | 1.43 | (0.90,2.27) | 2.24 | **(1.50,3.33)***** | < 0.001 | < 0.001 |
|  |  | No | Ref (1.00) | 2.12 | (0.88,5.14) | 2.14 | (0.92,4.98) | 2.15 | (0.91,5.11) | 0.141 | 0.234 |
| T2DM |  |  |  |  |  |  |  |  |  |  |  |
|  | Model l | Yes | Ref (1.00) | 0.50 | (0.18,1.38) | 0.63 | (0.24,1.71) | 0.58 | (0.22,1.56) | 0.587 | 0.532 |
|  |  | No | Ref (1.00) | 1.45 | (0.88,2.37) | 1.44 | (0.91,2.29) | 1.76 | **(1.19,2.60)**** | 0.006 | 0.015 |
|  | Model 2 | Yes | Ref (1.00) | 0.50 | (0.18,1.38) | 0.61 | (0.23,1.62) | 0.56 | (0.22,1.45) | 0.503 | 0.530 |
|  |  | No | Ref (1.00) | 1.40 | (0.85,2.31) | 1.40 | (0.88,2.22) | 1.69 | **(1.14,2.53)**** | 0.013 | 0.031 |
|  | Model 3 | Yes | Ref (1.00) | 0.46 | (0.17,1.25) | 0.56 | (0.22,1.43) | 0.53 | (0.22,1.30) | 0.494 | 0.385 |
|  |  | No | Ref (1.00) | 1.39 | (0.84,2.30) | 1.37 | (0.86,2.17) | 1.64 | **(1.10,2.46)*** | 0.020 | 0.051 |
|  | Model 4 | Yes | Ref (1.00) | 0.49 | (0.19,1.25) | 0.59 | (0.24,1.45) | 0.61 | (0.26,1.41) | 0.710 | 0.404 |
|  |  | No | Ref (1.00) | 1.66 | (0.98,2.83) | 1.79 | **(1.12,2.84)*** | 2.72 | **(1.79,4.14)***** | < 0.001 | < 0.001 |

^a^The variables adjusted in each model were the factors mentioned above except the stratification variables.

^b^Data were listed as the weighted odd ratio estimates and 95% confidence intervals, with *p < 0.05, **p < 0.01, ***p < 0.001.

^c^Q, quintile.

^d^Ref, reference.

^e^Tests for trends based on the variables containing the median values for each quartile.

^f^*P*_test_ was the result of Bonfreni correction.

Supplementary Table 21. Association of TyG-WC with myocardial infarction stratified by age, sex, race, exercise, smoking, drinking, and T2DM status

|  |  |  | TyG-WC | | | | | | | *P*_trend_^e^ | *P*_test_^f^ |
| --- | --- | --- | --- | --- | --- | --- | --- | --- | --- | --- | --- |
|  |  |  | Q1^c^ | Q2 | | Q3 | | Q4 | |  |  |
|  |  |  |  | HR/OR^b^ | 95% CI | HR/OR | 95% CI | HR/OR | 95% CI |  |  |
| Age |  |  |  |  |  |  |  |  |  |  |  |
|  | Model 1^a^ | > 50 | Ref (1.00)^d^ | 0.97 | (0.58,1.64) | 1.11 | (0.73,1.70) | 1.71 | **(1.09,2.66)*** | < 0.001 | 0.056 |
|  |  | ≤ 50 | Ref (1.00) | 1.98 | (0.59,6.62) | 2.86 | (0.95,8.61) | 7.07 | **(2.82,17.73)***** | < 0.001 | < 0.001 |
|  | Model 2 | > 50 | Ref (1.00) | 0.94 | (0.55,1.59) | 1.05 | (0.68,1.61) | 1.54 | (0.98,2.43) | 0.002 | 0.183 |
|  |  | ≤ 50 | Ref (1.00) | 1.79 | (0.55,5.83) | 2.52 | (0.84,7.62) | 5.80 | **(2.29,14.64)***** | < 0.001 | 0.001 |
|  | Model 3 | > 50 | Ref (1.00) | 0.92 | (0.54,1.57) | 1.02 | (0.66,1.58) | 1.51 | (0.96,2.38) | 0.002 | 0.216 |
|  |  | ≤ 50 | Ref (1.00) | 1.72 | (0.54,5.49) | 2.52 | (0.84,7.58) | 5.63 | **(2.22,14.27)***** | < 0.001 | 0.001 |
|  | Model 4 | > 50 | Ref (1.00) | 0.93 | (0.55,1.57) | 1.02 | (0.66,1.55) | 1.39 | (0.87,2.24) | 0.031 | 0.512 |
|  |  | ≤ 50 | Ref (1.00) | 1.89 | (0.58,6.13) | 2.53 | (0.78,8.22) | 5.46 | **(1.93,15.46)**** | 0.002 | 0.005 |
| Sex |  |  |  |  |  |  |  |  |  |  |  |
|  | Model l | Male | Ref (1.00) | 0.87 | (0.43,1.76) | 1.30 | (0.67,2.49) | 2.37 | **(1.23,4.55)*** | < 0.001 | 0.030 |
|  |  | Female | Ref (1.00) | 1.39 | (0.84,2.30) | 1.21 | (0.67,2.19) | 1.96 | **(1.12,3.44)*** | 0.028 | 0.057 |
|  | Model 2 | Male | Ref (1.00) | 0.87 | (0.43,1.77) | 1.28 | (0.66,2.48) | 2.28 | **(1.17,4.42)*** | < 0.001 | 0.047 |
|  |  | Female | Ref (1.00) | 1.34 | (0.79,2.26) | 1.12 | (0.62,2.04) | 1.67 | (0.90,3.11) | 0.137 | 0.310 |
|  | Model 3 | Male | Ref (1.00) | 0.88 | (0.42,1.83) | 1.27 | (0.64,2.51) | 2.19 | **(1.13,4.28)*** | < 0.001 | 0.065 |
|  |  | Female | Ref (1.00) | 1.27 | (0.75,2.17) | 1.05 | (0.57,1.92) | 1.60 | (0.88,2.94) | 0.160 | 0.372 |
|  | Model 4 | Male | Ref (1.00) | 0.93 | (0.46,1.86) | 1.31 | (0.70,2.48) | 2.16 | **(1.17,3.99)*** | < 0.001 | 0.043 |
|  |  | Female | Ref (1.00) | 1.37 | (0.81,2.31) | 1.03 | (0.55,1.96) | 1.53 | (0.81,2.86) | 0.276 | 0.552 |
| Race |  |  |  |  |  |  |  |  |  |  |  |
|  | Model l | Non-hispanic white | Ref (1.00) | 0.97 | (0.58,1.61) | 1.14 | (0.72,1.80) | 2.00 | **(1.26,3.19)**** | < 0.001 | 0.011 |
|  |  | Others | Ref (1.00) | 1.41 | (0.62,3.20) | 1.35 | (0.67,2.72) | 2.51 | **(1.32,4.80)**** | 0.001 | 0.017 |
|  | Model 2 | Non-hispanic white | Ref (1.00) | 0.95 | (0.57,1.60) | 1.08 | (0.68,1.71) | 1.85 | **(1.14,2.99)*** | < 0.001 | 0.040 |
|  |  | Others | Ref (1.00) | 1.41 | (0.63,3.18) | 1.32 | (0.66,2.63) | 2.37 | **(1.25,4.48)**** | 0.002 | 0.026 |
|  | Model 3 | Non-hispanic white | Ref (1.00) | 0.93 | (0.54,1.59) | 1.02 | (0.64,1.64) | 1.71 | **(1.06,2.77)*** | 0.001 | 0.088 |
|  |  | Others | Ref (1.00) | 1.40 | (0.62,3.18) | 1.30 | (0.64,2.63) | 2.36 | **(1.23,4.51)*** | 0.002 | 0.030 |
|  | Model 4 | Non-hispanic white | Ref (1.00) | 1.02 | (0.60,1.74) | 1.10 | (0.68,1.76) | 1.72 | **(1.05,2.83)*** | 0.004 | 0.095 |
|  |  | Others | Ref (1.00) | 1.43 | (0.65,3.15) | 1.29 | (0.65,2.55) | 2.23 | **(1.19,4.19)*** | 0.010 | 0.039 |
| Exercise |  |  |  |  |  |  |  |  |  |  |  |
|  | Model l | Yes | Ref (1.00) | 1.31 | (0.63,2.72) | 1.50 | (0.84,2.70) | 2.38 | **(1.27,4.46)**** | < 0.001 | 0.022 |
|  |  | No | Ref (1.00) | 0.80 | (0.38,1.66) | 0.93 | (0.50,1.71) | 1.78 | (0.92,3.47) | 0.022 | 0.260 |
|  | Model 2 | Yes | Ref (1.00) | 1.33 | (0.64,2.74) | 1.53 | (0.86,2.75) | 2.44 | **(1.31,4.55)**** | < 0.001 | 0.016 |
|  |  | No | Ref (1.00) | 0.78 | (0.37,1.62) | 0.84 | (0.45,1.59) | 1.61 | (0.81,3.20) | 0.054 | 0.503 |
|  | Model 3 | Yes | Ref (1.00) | 1.35 | (0.64,2.82) | 1.56 | (0.86,2.84) | 2.49 | **(1.31,4.72)**** | < 0.001 | 0.017 |
|  |  | No | Ref (1.00) | 0.76 | (0.37,1.59) | 0.81 | (0.43,1.53) | 1.54 | (0.77,3.06) | 0.072 | 0.649 |
|  | Model 4 | Yes | Ref (1.00) | 1.46 | (0.72,2.94) | 1.55 | (0.87,2.77) | 2.26 | **(1.18,4.34)*** | 0.002 | 0.044 |
|  |  | No | Ref (1.00) | 0.82 | (0.38,1.76) | 0.92 | (0.48,1.76) | 1.68 | (0.83,3.42) | 0.045 | 0.442 |
| Smoking |  |  |  |  |  |  |  |  |  |  |  |
|  | Model l | Yes | Ref (1.00) | 0.64 | (0.33,1.25) | 0.59 | (0.29,1.22) | 1.31 | (0.66,2.60) | 0.218 | 0.457 |
|  |  | No | Ref (1.00) | 1.56 | (0.88,2.78) | 1.88 | **(1.09,3.26)*** | 3.10 | **(1.73,5.53)***** | < 0.001 | 0.001 |
|  | Model 2 | Yes | Ref (1.00) | 0.62 | (0.31,1.27) | 0.60 | (0.29,1.27) | 1.33 | (0.68,2.60) | 0.174 | 0.541 |
|  |  | No | Ref (1.00) | 1.55 | (0.87,2.77) | 1.84 | **(1.06,3.18)*** | 2.97 | **(1.66,5.33)***** | < 0.001 | 0.001 |
|  | Model 3 | Yes | Ref (1.00) | 0.62 | (0.31,1.26) | 0.63 | (0.30,1.33) | 1.34 | (0.68,2.63) | 0.161 | 0.561 |
|  |  | No | Ref (1.00) | 1.51 | (0.83,2.74) | 1.75 | (1.00,3.06) | 2.78 | **(1.54,5.00)**** | < 0.001 | 0.003 |
|  | Model 4 | Yes | Ref (1.00) | 0.71 | (0.37,1.37) | 0.72 | (0.37,1.42) | 1.53 | (0.79,2.99) | 0.106 | 0.619 |
|  |  | No | Ref (1.00) | 1.54 | (0.82,2.87) | 1.72 | (0.96,3.08) | 2.56 | **(1.35,4.86)**** | 0.001 | 0.013 |
| Drinking |  |  |  |  |  |  |  |  |  |  |  |
|  | Model l | Yes | Ref (1.00) | 0.93 | (0.54,1.61) | 1.15 | (0.74,1.79) | 2.12 | **(1.35,3.32)**** | < 0.001 | 0.004 |
|  |  | No | Ref (1.00) | 2.01 | (0.82,4.90) | 1.80 | (0.85,3.84) | 3.03 | **(1.36,6.78)**** | 0.003 | 0.022 |
|  | Model 2 | Yes | Ref (1.00) | 0.94 | (0.55,1.62) | 1.16 | (0.75,1.79) | 2.03 | **(1.30,3.16)**** | < 0.001 | 0.006 |
|  |  | No | Ref (1.00) | 2.02 | (0.82,5.00) | 1.75 | (0.80,3.80) | 2.86 | **(1.26,6.53)*** | 0.010 | 0.039 |
|  | Model 3 | Yes | Ref (1.00) | 0.92 | (0.54,1.59) | 1.12 | (0.72,1.75) | 1.95 | **(1.25,3.05)**** | < 0.001 | 0.011 |
|  |  | No | Ref (1.00) | 1.99 | (0.80,4.94) | 1.67 | (0.75,3.73) | 2.69 | **(1.17,6.22)*** | 0.017 | 0.062 |
|  | Model 4 | Yes | Ref (1.00) | 1.02 | (0.60,1.76) | 1.22 | (0.79,1.89) | 2.02 | **(1.27,3.20)**** | < 0.001 | 0.010 |
|  |  | No | Ref (1.00) | 2.01 | (0.81,4.97) | 1.67 | (0.75,3.75) | 2.56 | **(1.06,6.15)*** | 0.045 | 0.108 |
| T2DM |  |  |  |  |  |  |  |  |  |  |  |
|  | Model l | Yes | Ref (1.00) | 0.49 | (0.13,1.81) | 0.42 | (0.12,1.43) | 0.73 | (0.21,2.52) | 0.326 | 0.484 |
|  |  | No | Ref (1.00) | 1.12 | (0.71,1.76) | 1.32 | (0.87,2.01) | 2.05 | **(1.32,3.20)**** | < 0.001 | 0.005 |
|  | Model 2 | Yes | Ref (1.00) | 0.48 | (0.13,1.75) | 0.42 | (0.13,1.37) | 0.68 | (0.21,2.28) | 0.439 | 0.443 |
|  |  | No | Ref (1.00) | 1.11 | (0.70,1.76) | 1.29 | (0.84,1.98) | 1.96 | **(1.23,3.14)**** | 0.001 | 0.016 |
|  | Model 3 | Yes | Ref (1.00) | 0.52 | (0.15,1.78) | 0.46 | (0.15,1.42) | 0.73 | (0.23,2.27) | 0.448 | 0.526 |
|  |  | No | Ref (1.00) | 1.09 | (0.68,1.73) | 1.24 | (0.80,1.90) | 1.88 | **(1.18,3.01)**** | 0.003 | 0.027 |
|  | Model 4 | Yes | Ref (1.00) | 0.56 | (0.18,1.78) | 0.48 | (0.17,1.35) | 0.76 | (0.27,2.14) | 0.466 | 0.482 |
|  |  | No | Ref (1.00) | 1.17 | (0.73,1.88) | 1.37 | (0.88,2.13) | 2.11 | **(1.34,3.31)**** | < 0.001 | 0.004 |

^a^The variables adjusted in each model were the factors mentioned above except the stratification variables.

^b^Data were listed as the weighted odd ratio estimates and 95% confidence intervals, with *p < 0.05, **p < 0.01, ***p < 0.001.

^c^Q, quintile.

^d^Ref, reference.

^e^Tests for trends based on the variables containing the median values for each quartile.

^f^*P*_test_ was the result of Bonfreni correction.

Supplementary Table 22. Association of TyG-WtHR with myocardial infarction stratified by age, sex, race, exercise, smoking, drinking, and T2DM status

|  |  |  | TyG-WtHR | | | | | | | *P*_trend_^e^ | *P*_test_^f^ |
| --- | --- | --- | --- | --- | --- | --- | --- | --- | --- | --- | --- |
|  |  |  | Q1^c^ | Q2 | | Q3 | | Q4 | |  |  |
|  |  |  |  | HR/OR^b^ | 95% CI | HR/OR | 95% CI | HR/OR | 95% CI |  |  |
| Age |  |  |  |  |  |  |  |  |  |  |  |
|  | Model 1^a^ | > 50 | Ref (1.00)^d^ | 1.09 | (0.68,1.74) | 1.60 | **(1.09,2.36)*** | 2.13 | **(1.38,3.29)**** | < 0.001 | 0.002 |
|  |  | ≤ 50 | Ref (1.00) | 1.51 | (0.49,4.68) | 2.99 | (0.82,10.89) | 9.89 | **(3.58,27.30)***** | < 0.001 | < 0.001 |
|  | Model 2 | > 50 | Ref (1.00) | 1.05 | (0.65,1.68) | 1.52 | **(1.02,2.28)*** | 1.92 | **(1.24,3.00)**** | < 0.001 | 0.013 |
|  |  | ≤ 50 | Ref (1.00) | 1.38 | (0.44,4.34) | 2.62 | (0.75,9.21) | 8.06 | **(2.84,22.85)***** | < 0.001 | < 0.001 |
|  | Model 3 | > 50 | Ref (1.00) | 1.00 | (0.62,1.61) | 1.43 | (0.96,2.14) | 1.80 | **(1.16,2.78)**** | < 0.001 | 0.026 |
|  |  | ≤ 50 | Ref (1.00) | 1.28 | (0.41,4.03) | 2.56 | (0.74,8.79) | 7.68 | **(2.71,21.77)***** | < 0.001 | 0.001 |
|  | Model 4 | > 50 | Ref (1.00) | 1.03 | (0.64,1.66) | 1.48 | **(1.01,2.18)*** | 1.71 | **(1.09,2.69)*** | 0.003 | 0.062 |
|  |  | ≤ 50 | Ref (1.00) | 1.47 | (0.47,4.63) | 2.71 | (0.74,9.98) | 8.23 | **(2.55,26.59)**** | < 0.001 | 0.002 |
| Sex |  |  |  |  |  |  |  |  |  |  |  |
|  | Model l | Male | Ref (1.00) | 1.00 | (0.57,1.76) | 1.78 | **(1.04,3.03)*** | 3.01 | **(1.71,5.31)***** | < 0.001 | 0.001 |
|  |  | Female | Ref (1.00) | 1.40 | (0.67,2.96) | 1.40 | (0.78,2.49) | 2.13 | **(1.10,4.16)*** | 0.009 | 0.078 |
|  | Model 2 | Male | Ref (1.00) | 0.98 | (0.55,1.75) | 1.74 | **(1.01,3.00)*** | 2.87 | **(1.60,5.12)**** | < 0.001 | 0.002 |
|  |  | Female | Ref (1.00) | 1.33 | (0.61,2.90) | 1.28 | (0.70,2.35) | 1.82 | (0.87,3.80) | 0.060 | 0.327 |
|  | Model 3 | Male | Ref (1.00) | 0.95 | (0.53,1.71) | 1.67 | (0.96,2.90) | 2.68 | **(1.51,4.78)***** | < 0.001 | 0.003 |
|  |  | Female | Ref (1.00) | 1.25 | (0.58,2.72) | 1.18 | (0.64,2.17) | 1.70 | (0.83,3.47) | 0.081 | 0.436 |
|  | Model 4 | Male | Ref (1.00) | 1.01 | (0.58,1.77) | 1.68 | (1.00,2.83) | 2.68 | **(1.54,4.64)**** | < 0.001 | 0.002 |
|  |  | Female | Ref (1.00) | 1.36 | (0.63,2.96) | 1.28 | (0.69,2.36) | 1.67 | (0.82,3.42) | 0.128 | 0.477 |
| Race |  |  |  |  |  |  |  |  |  |  |  |
|  | Model l | Non-hispanic white | Ref (1.00) | 1.02 | (0.62,1.67) | 1.43 | (0.96,2.12) | 2.40 | **(1.50,3.83)***** | < 0.001 | 0.001 |
|  |  | Others | Ref (1.00) | 1.48 | (0.77,2.85) | 1.96 | (1.00,3.85) | 2.60 | **(1.44,4.70)**** | 0.001 | 0.006 |
|  | Model 2 | Non-hispanic white | Ref (1.00) | 0.98 | (0.59,1.65) | 1.35 | (0.90,2.03) | 2.19 | **(1.33,3.62)**** | < 0.001 | 0.007 |
|  |  | Others | Ref (1.00) | 1.45 | (0.75,2.80) | 1.89 | (0.97,3.69) | 2.41 | **(1.35,4.31)**** | 0.002 | 0.010 |
|  | Model 3 | Non-hispanic white | Ref (1.00) | 0.93 | (0.55,1.58) | 1.25 | (0.82,1.90) | 2.01 | **(1.22,3.30)**** | < 0.001 | 0.019 |
|  |  | Others | Ref (1.00) | 1.45 | (0.75,2.79) | 1.89 | (0.97,3.68) | 2.37 | **(1.32,4.25)**** | 0.003 | 0.013 |
|  | Model 4 | Non-hispanic white | Ref (1.00) | 1.04 | (0.62,1.75) | 1.35 | (0.89,2.05) | 2.09 | **(1.26,3.47)**** | < 0.001 | 0.014 |
|  |  | Others | Ref (1.00) | 1.50 | (0.77,2.91) | 1.94 | (1.01,3.70) | 2.28 | **(1.22,4.24)*** | 0.013 | 0.031 |
| Exercise |  |  |  |  |  |  |  |  |  |  |  |
|  | Model l | Yes | Ref (1.00) | 1.26 | (0.64,2.49) | 1.74 | (0.95,3.18) | 2.53 | **(1.26,5.06)**** | < 0.001 | 0.028 |
|  |  | No | Ref (1.00) | 0.87 | (0.46,1.64) | 1.32 | (0.77,2.28) | 2.22 | **(1.15,4.30)*** | 0.007 | 0.055 |
|  | Model 2 | Yes | Ref (1.00) | 1.25 | (0.62,2.49) | 1.77 | (0.96,3.24) | 2.58 | **(1.29,5.14)**** | < 0.001 | 0.023 |
|  |  | No | Ref (1.00) | 0.83 | (0.44,1.57) | 1.16 | (0.66,2.06) | 1.96 | (0.99,3.89) | 0.021 | 0.158 |
|  | Model 3 | Yes | Ref (1.00) | 1.27 | (0.63,2.57) | 1.76 | (0.95,3.26) | 2.57 | **(1.27,5.20)**** | < 0.001 | 0.028 |
|  |  | No | Ref (1.00) | 0.79 | (0.42,1.51) | 1.11 | (0.62,1.97) | 1.85 | (0.93,3.68) | 0.031 | 0.243 |
|  | Model 4 | Yes | Ref (1.00) | 1.37 | (0.69,2.70) | 1.81 | (0.98,3.36) | 2.41 | **(1.17,4.94)*** | 0.002 | 0.052 |
|  |  | No | Ref (1.00) | 0.87 | (0.45,1.71) | 1.21 | (0.68,2.18) | 2.07 | **(1.03,4.16)*** | 0.018 | 0.126 |
| Smoking |  |  |  |  |  |  |  |  |  |  |  |
|  | Model l | Yes | Ref (1.00) | 0.58 | (0.28,1.22) | 1.14 | (0.62,2.09) | 1.57 | (0.72,3.43) | 0.088 | 0.456 |
|  |  | No | Ref (1.00) | 1.72 | **(1.07,2.77)*** | 2.18 | **(1.31,3.63)**** | 3.67 | **(2.13,6.31)***** | < 0.001 | < 0.001 |
|  | Model 2 | Yes | Ref (1.00) | 0.57 | (0.26,1.25) | 1.16 | (0.64,2.12) | 1.58 | (0.73,3.41) | 0.077 | 0.484 |
|  |  | No | Ref (1.00) | 1.70 | **(1.05,2.75)*** | 2.12 | **(1.27,3.56)**** | 3.51 | **(2.02,6.10)***** | < 0.001 | < 0.001 |
|  | Model 3 | Yes | Ref (1.00) | 0.56 | (0.25,1.25) | 1.15 | (0.63,2.12) | 1.58 | (0.74,3.41) | 0.071 | 0.465 |
|  |  | No | Ref (1.00) | 1.60 | (0.98,2.61) | 1.96 | **(1.16,3.31)*** | 3.18 | **(1.83,5.52)***** | < 0.001 | < 0.001 |
|  | Model 4 | Yes | Ref (1.00) | 0.67 | (0.32,1.40) | 1.40 | (0.80,2.46) | 1.87 | (0.88,3.99) | 0.037 | 0.306 |
|  |  | No | Ref (1.00) | 1.65 | (1.00,2.74) | 1.98 | **(1.17,3.36)*** | 3.02 | **(1.70,5.39)***** | < 0.001 | 0.001 |
| Drinking |  |  |  |  |  |  |  |  |  |  |  |
|  | Model l | Yes | Ref (1.00) | 1.10 | (0.67,1.81) | 1.41 | (0.96,2.09) | 2.54 | **(1.62,3.97)***** | < 0.001 | < 0.001 |
|  |  | No | Ref (1.00) | 1.57 | (0.59,4.16) | 3.06 | **(1.21,7.73)*** | 3.84 | **(1.47,10.00)**** | < 0.001 | 0.019 |
|  | Model 2 | Yes | Ref (1.00) | 1.10 | (0.67,1.79) | 1.39 | (0.94,2.06) | 2.39 | **(1.53,3.74)***** | < 0.001 | 0.001 |
|  |  | No | Ref (1.00) | 1.55 | (0.56,4.31) | 3.06 | **(1.15,8.15)*** | 3.73 | **(1.35,10.32)*** | 0.001 | 0.035 |
|  | Model 3 | Yes | Ref (1.00) | 1.06 | (0.64,1.74) | 1.34 | (0.90,1.99) | 2.27 | **(1.45,3.54)***** | < 0.001 | 0.001 |
|  |  | No | Ref (1.00) | 1.46 | (0.50,4.29) | 2.78 | **(1.01,7.63)*** | 3.38 | **(1.16,9.90)*** | 0.003 | 0.080 |
|  | Model 4 | Yes | Ref (1.00) | 1.19 | (0.73,1.95) | 1.47 | (1.00,2.16) | 2.40 | **(1.51,3.79)***** | < 0.001 | 0.001 |
|  |  | No | Ref (1.00) | 1.53 | (0.53,4.44) | 2.94 | **(1.07,8.04)*** | 3.35 | **(1.13,9.95)*** | 0.007 | 0.091 |
| T2DM |  |  |  |  |  |  |  |  |  |  |  |
|  | Model l | Yes | Ref (1.00) | 0.42 | (0.11,1.59) | 0.64 | (0.16,2.58) | 0.73 | (0.19,2.85) | 0.425 | 0.592 |
|  |  | No | Ref (1.00) | 1.15 | (0.73,1.81) | 1.50 | **(1.05,2.17)*** | 2.43 | **(1.56,3.78)***** | < 0.001 | < 0.001 |
|  | Model 2 | Yes | Ref (1.00) | 0.41 | (0.11,1.51) | 0.63 | (0.17,2.35) | 0.68 | (0.18,2.51) | 0.562 | 0.532 |
|  |  | No | Ref (1.00) | 1.13 | (0.71,1.79) | 1.46 | **(1.00,2.13)*** | 2.33 | **(1.46,3.72)**** | < 0.001 | 0.002 |
|  | Model 3 | Yes | Ref (1.00) | 0.46 | (0.13,1.60) | 0.66 | (0.19,2.32) | 0.71 | (0.20,2.48) | 0.597 | 0.650 |
|  |  | No | Ref (1.00) | 1.08 | (0.67,1.73) | 1.38 | (0.95,2.03) | 2.19 | **(1.37,3.50)**** | < 0.001 | 0.004 |
|  | Model 4 | Yes | Ref (1.00) | 0.49 | (0.15,1.55) | 0.68 | (0.21,2.18) | 0.75 | (0.24,2.36) | 0.556 | 0.665 |
|  |  | No | Ref (1.00) | 1.20 | (0.75,1.94) | 1.59 | **(1.09,2.31)*** | 2.53 | **(1.60,3.98)***** | < 0.001 | < 0.001 |

^a^The variables adjusted in each model were the factors mentioned above except the stratification variables.

^b^Data were listed as the weighted odd ratio estimates and 95% confidence intervals, with *p < 0.05, **p < 0.01, ***p < 0.001.

^c^Q, quintile.

^d^Ref, reference.

^e^Tests for trends based on the variables containing the median values for each quartile.

^f^*P*_test_ was the result of Bonfreni correction.

Supplementary Table 23. Association of TyG-BMI with myocardial infarction stratified by age, sex, race, exercise, smoking, drinking, and T2DM status

|  |  |  | TyG-BMI | | | | | | | *P*_trend_^e^ | *P*_test_^f^ |
| --- | --- | --- | --- | --- | --- | --- | --- | --- | --- | --- | --- |
|  |  |  | Q1^c^ | Q2 | | Q3 | | Q4 | |  |  |
|  |  |  |  | HR/OR^b^ | 95% CI | HR/OR | 95% CI | HR/OR | 95% CI |  |  |
| Age |  |  |  |  |  |  |  |  |  |  |  |
|  | Model 1^a^ | > 50 | Ref (1.00)^d^ | 0.82 | (0.59,1.14) | 0.89 | (0.64,1.25) | 1.32 | (0.96,1.81) | 0.018 | 0.276 |
|  |  | ≤ 50 | Ref (1.00) | 2.01 | (0.51,7.99) | 3.17 | **(1.05,9.58)*** | 4.96 | **(1.77,13.93)**** | 0.001 | 0.008 |
|  | Model 2 | > 50 | Ref (1.00) | 0.79 | (0.56,1.11) | 0.86 | (0.61,1.22) | 1.22 | (0.88,1.69) | 0.054 | 0.533 |
|  |  | ≤ 50 | Ref (1.00) | 1.91 | (0.49,7.47) | 2.80 | (0.94,8.33) | 4.12 | **(1.44,11.78)**** | 0.004 | 0.027 |
|  | Model 3 | > 50 | Ref (1.00) | 0.78 | (0.55,1.10) | 0.84 | (0.59,1.20) | 1.21 | (0.87,1.69) | 0.058 | 0.476 |
|  |  | ≤ 50 | Ref (1.00) | 1.82 | (0.48,6.94) | 2.78 | (0.94,8.21) | 4.03 | **(1.42,11.45)**** | 0.004 | 0.028 |
|  | Model 4 | > 50 | Ref (1.00) | 0.81 | (0.58,1.15) | 0.84 | (0.59,1.20) | 1.11 | (0.77,1.62) | 0.299 | 0.722 |
|  |  | ≤ 50 | Ref (1.00) | 1.98 | (0.51,7.62) | 2.91 | (0.99,8.54) | 3.61 | **(1.10,11.82)*** | 0.029 | 0.103 |
| Sex |  |  |  |  |  |  |  |  |  |  |  |
|  | Model l | Male | Ref (1.00) | 1.03 | (0.65,1.63) | 1.21 | (0.75,1.94) | 2.73 | **(1.66,4.48)***** | < 0.001 | < 0.001 |
|  |  | Female | Ref (1.00) | 0.99 | (0.61,1.61) | 1.09 | (0.65,1.83) | 1.32 | (0.81,2.17) | 0.249 | 0.795 |
|  | Model 2 | Male | Ref (1.00) | 1.02 | (0.63,1.64) | 1.19 | (0.73,1.93) | 2.62 | **(1.58,4.33)***** | < 0.001 | 0.001 |
|  |  | Female | Ref (1.00) | 0.95 | (0.58,1.57) | 0.99 | (0.59,1.67) | 1.13 | (0.66,1.95) | 0.607 | 1.000 |
|  | Model 3 | Male | Ref (1.00) | 1.02 | (0.63,1.67) | 1.16 | (0.70,1.91) | 2.53 | **(1.54,4.18)***** | < 0.001 | 0.001 |
|  |  | Female | Ref (1.00) | 0.91 | (0.55,1.50) | 0.94 | (0.55,1.59) | 1.09 | (0.64,1.85) | 0.682 | 1.000 |
|  | Model 4 | Male | Ref (1.00) | 1.05 | (0.65,1.69) | 1.11 | (0.69,1.78) | 2.39 | **(1.47,3.90)**** | < 0.001 | 0.002 |
|  |  | Female | Ref (1.00) | 0.93 | (0.57,1.54) | 0.93 | (0.54,1.61) | 0.99 | (0.56,1.75) | 0.999 | 1.000 |
| Race |  |  |  |  |  |  |  |  |  |  |  |
|  | Model l | Non-hispanic white | Ref (1.00) | 0.87 | (0.62,1.23) | 1.02 | (0.69,1.50) | 1.89 | **(1.29,2.78)**** | < 0.001 | 0.004 |
|  |  | Others | Ref (1.00) | 1.35 | (0.66,2.78) | 1.25 | (0.71,2.20) | 1.86 | **(1.06,3.28)*** | 0.033 | 0.096 |
|  | Model 2 | Non-hispanic white | Ref (1.00) | 0.84 | (0.59,1.19) | 0.97 | (0.65,1.43) | 1.74 | **(1.16,2.61)**** | 0.002 | 0.023 |
|  |  | Others | Ref (1.00) | 1.35 | (0.66,2.78) | 1.23 | (0.70,2.16) | 1.78 | **(1.03,3.10)*** | 0.046 | 0.122 |
|  | Model 3 | Non-hispanic white | Ref (1.00) | 0.82 | (0.57,1.18) | 0.92 | (0.61,1.39) | 1.66 | **(1.12,2.46)*** | 0.003 | 0.037 |
|  |  | Others | Ref (1.00) | 1.35 | (0.66,2.77) | 1.24 | (0.70,2.18) | 1.76 | **(1.00,3.09)*** | 0.056 | 0.148 |
|  | Model 4 | Non-hispanic white | Ref (1.00) | 0.88 | (0.61,1.28) | 0.92 | (0.61,1.39) | 1.58 | **(1.04,2.40)*** | 0.016 | 0.103 |
|  |  | Others | Ref (1.00) | 1.34 | (0.67,2.71) | 1.20 | (0.69,2.10) | 1.62 | (0.92,2.85) | 0.155 | 0.282 |
| Exercise |  |  |  |  |  |  |  |  |  |  |  |
|  | Model l | Yes | Ref (1.00) | 1.14 | (0.67,1.95) | 1.26 | (0.83,1.92) | 1.90 | **(1.19,3.02)**** | 0.001 | 0.023 |
|  |  | No | Ref (1.00) | 0.71 | (0.42,1.18) | 0.90 | (0.50,1.61) | 1.77 | (0.95,3.30) | 0.039 | 0.221 |
|  | Model 2 | Yes | Ref (1.00) | 1.14 | (0.66,1.95) | 1.28 | (0.84,1.94) | 1.92 | **(1.21,3.06)**** | < 0.001 | 0.019 |
|  |  | No | Ref (1.00) | 0.65 | (0.38,1.12) | 0.81 | (0.44,1.50) | 1.54 | (0.82,2.89) | 0.090 | 0.362 |
|  | Model 3 | Yes | Ref (1.00) | 1.16 | (0.67,2.02) | 1.30 | (0.85,1.99) | 1.98 | **(1.24,3.16)**** | < 0.001 | 0.014 |
|  |  | No | Ref (1.00) | 0.63 | (0.37,1.09) | 0.77 | (0.41,1.44) | 1.46 | (0.77,2.75) | 0.117 | 0.285 |
|  | Model 4 | Yes | Ref (1.00) | 1.17 | (0.68,2.02) | 1.28 | (0.84,1.94) | 1.69 | **(1.01,2.82)*** | 0.020 | 0.135 |
|  |  | No | Ref (1.00) | 0.67 | (0.39,1.17) | 0.78 | (0.41,1.49) | 1.46 | (0.79,2.73) | 0.129 | 0.466 |
| Smoking |  |  |  |  |  |  |  |  |  |  |  |
|  | Model l | Yes | Ref (1.00) | 0.66 | (0.34,1.26) | 0.93 | (0.47,1.84) | 1.45 | (0.74,2.86) | 0.161 | 0.618 |
|  |  | No | Ref (1.00) | 1.24 | (0.82,1.87) | 1.32 | (0.87,2.00) | 2.45 | **(1.55,3.85)***** | < 0.001 | 0.001 |
|  | Model 2 | Yes | Ref (1.00) | 0.66 | (0.33,1.35) | 0.96 | (0.49,1.87) | 1.49 | (0.76,2.90) | 0.131 | 0.723 |
|  |  | No | Ref (1.00) | 1.22 | (0.80,1.86) | 1.29 | (0.84,1.97) | 2.33 | **(1.47,3.70)***** | < 0.001 | 0.001 |
|  | Model 3 | Yes | Ref (1.00) | 0.67 | (0.33,1.36) | 0.99 | (0.50,1.96) | 1.51 | (0.79,2.90) | 0.114 | 0.640 |
|  |  | No | Ref (1.00) | 1.18 | (0.76,1.83) | 1.21 | (0.78,1.87) | 2.19 | **(1.38,3.47)**** | < 0.001 | 0.003 |
|  | Model 4 | Yes | Ref (1.00) | 0.76 | (0.40,1.47) | 1.16 | (0.61,2.21) | 1.63 | (0.84,3.18) | 0.087 | 0.438 |
|  |  | No | Ref (1.00) | 1.18 | (0.76,1.84) | 1.13 | (0.73,1.77) | 1.95 | **(1.19,3.19)**** | 0.006 | 0.026 |
| Drinking |  |  |  |  |  |  |  |  |  |  |  |
|  | Model l | Yes | Ref (1.00) | 0.87 | (0.60,1.26) | 0.98 | (0.65,1.46) | 1.90 | **(1.30,2.78)**** | < 0.001 | 0.003 |
|  |  | No | Ref (1.00) | 1.63 | (0.77,3.43) | 1.91 | **(1.10,3.31)**** | 2.56 | **(1.24,5.29)**** | 0.009 | 0.036 |
|  | Model 2 | Yes | Ref (1.00) | 0.89 | (0.61,1.29) | 0.99 | (0.66,1.48) | 1.84 | **(1.26,2.69)**** | < 0.001 | 0.005 |
|  |  | No | Ref (1.00) | 1.65 | (0.77,3.53) | 1.91 | **(1.07,3.39)**** | 2.53 | **(1.18,5.41)**** | 0.014 | 0.052 |
|  | Model 3 | Yes | Ref (1.00) | 0.87 | (0.60,1.27) | 0.96 | (0.63,1.45) | 1.80 | **(1.24,2.61)**** | 0.001 | 0.007 |
|  |  | No | Ref (1.00) | 1.56 | (0.71,3.46) | 1.81 | (0.98,3.32) | 2.35 | **(1.06,5.18)**** | 0.026 | 0.105 |
|  | Model 4 | Yes | Ref (1.00) | 0.93 | (0.63,1.36) | 0.97 | (0.64,1.49) | 1.75 | **(1.15,2.66)**** | 0.006 | 0.029 |
|  |  | No | Ref (1.00) | 1.62 | (0.72,3.60) | 1.79 | (0.95,3.39) | 2.21 | (0.97,5.03) | 0.061 | 0.177 |
| T2DM |  |  |  |  |  |  |  |  |  |  |  |
|  | Model l | Yes | Ref (1.00) | 0.84 | (0.33,2.13) | 0.82 | (0.32,2.13) | 0.95 | (0.38,2.36) | 0.786 | 1.000 |
|  |  | No | Ref (1.00) | 0.94 | (0.66,1.32) | 1.02 | (0.70,1.50) | 1.87 | **(1.24,2.81)**** | 0.003 | 0.009 |
|  | Model 2 | Yes | Ref (1.00) | 0.79 | (0.31,2.07) | 0.80 | (0.31,2.06) | 0.86 | (0.34,2.15) | 0.990 | 1.000 |
|  |  | No | Ref (1.00) | 0.92 | (0.64,1.31) | 1.00 | (0.67,1.48) | 1.79 | **(1.17,2.75)**** | 0.007 | 0.023 |
|  | Model 3 | Yes | Ref (1.00) | 0.83 | (0.32,2.13) | 0.81 | (0.32,2.02) | 0.85 | (0.35,2.06) | 0.898 | 1.000 |
|  |  | No | Ref (1.00) | 0.90 | (0.63,1.29) | 0.97 | (0.65,1.44) | 1.75 | **(1.14,2.66)*** | 0.009 | 0.031 |
|  | Model 4 | Yes | Ref (1.00) | 0.83 | (0.33,2.07) | 0.77 | (0.32,1.85) | 0.84 | (0.37,1.90) | 0.855 | 1.000 |
|  |  | No | Ref (1.00) | 0.96 | (0.67,1.38) | 1.04 | (0.70,1.55) | 1.83 | **(1.23,2.74)**** | 0.003 | 0.010 |

^a^The variables adjusted in each model were the factors mentioned above except the stratification variables.

^b^Data were listed as the weighted odd ratio estimates and 95% confidence intervals, with *p < 0.05, **p < 0.01, ***p < 0.001.

^c^Q, quintile.

^d^Ref, reference.

^e^Tests for trends based on the variables containing the median values for each quartile.

^f^*P*_test_ was the result of Bonfreni correction.

Supplementary Table 24. Association of TyG with angina pectoris stratified by age, sex, race, exercise, smoking, drinking, and T2DM status

|  |  |  | TyG | | | | | | | *P*_trend_^e^ | *P*_test_^f^ |
| --- | --- | --- | --- | --- | --- | --- | --- | --- | --- | --- | --- |
|  |  |  | Q1^c^ | Q2 | | Q3 | | Q4 | |  |  |
|  |  |  |  | HR/OR^b^ | 95% CI | HR/OR | 95% CI | HR/OR | 95% CI |  |  |
| Age |  |  |  |  |  |  |  |  |  |  |  |
|  | Model 1^a^ | > 50 | Ref (1.00)^d^ | 0.96 | (0.54,1.69) | 1.52 | (0.94,2.44) | 1.76 | **(1.15,2.71)**** | 0.002 | 0.030 |
|  |  | ≤ 50 | Ref (1.00) | 2.08 | (0.76,5.66) | 5.16 | **(1.72,15.49)**** | 5.40 | **(1.77,16.46)**** | 0.001 | 0.010 |
|  | Model 2 | > 50 | Ref (1.00) | 0.87 | (0.49,1.57) | 1.37 | (0.85,2.22) | 1.54 | (0.99,2.39) | 0.010 | 0.159 |
|  |  | ≤ 50 | Ref (1.00) | 1.88 | (0.68,5.21) | 4.67 | **(1.51,14.49)**** | 4.50 | **(1.42,14.27)*** | 0.003 | 0.024 |
|  | Model 3 | > 50 | Ref (1.00) | 0.88 | (0.49,1.58) | 1.40 | (0.86,2.27) | 1.55 | (0.99,2.42) | 0.010 | 0.160 |
|  |  | ≤ 50 | Ref (1.00) | 1.89 | (0.68,5.27) | 4.62 | **(1.51,14.18)**** | 4.35 | **(1.38,13.72)*** | 0.003 | 0.024 |
|  | Model 4 | > 50 | Ref (1.00) | 0.95 | (0.52,1.72) | 1.56 | (0.95,2.59) | 1.80 | **(1.12,2.90)*** | 0.002 | 0.047 |
|  |  | ≤ 50 | Ref (1.00) | 2.11 | (0.73,6.10) | 5.49 | **(1.72,17.56)**** | 4.89 | **(1.32,18.10)*** | 0.005 | 0.014 |
| Sex |  |  |  |  |  |  |  |  |  |  |  |
|  | Model l | Male | Ref (1.00) | 0.98 | (0.54,1.75) | 1.34 | (0.68,2.64) | 1.71 | (0.93,3.14) | 0.029 | 0.246 |
|  |  | Female | Ref (1.00) | 1.05 | (0.40,2.75) | 2.51 | **(1.29,4.91)**** | 2.64 | **(1.32,5.28)**** | 0.002 | 0.020 |
|  | Model 2 | Male | Ref (1.00) | 0.95 | (0.52,1.72) | 1.30 | (0.65,2.58) | 1.63 | (0.87,3.05) | 0.046 | 0.368 |
|  |  | Female | Ref (1.00) | 0.97 | (0.37,2.53) | 2.28 | **(1.15,4.49)*** | 2.26 | **(1.12,4.56)*** | 0.007 | 0.054 |
|  | Model 3 | Male | Ref (1.00) | 0.94 | (0.52,1.71) | 1.28 | (0.65,2.52) | 1.60 | (0.86,2.98) | 0.053 | 0.406 |
|  |  | Female | Ref (1.00) | 0.96 | (0.36,2.53) | 2.29 | **(1.16,4.52)*** | 2.25 | **(1.11,4.57)*** | 0.006 | 0.052 |
|  | Model 4 | Male | Ref (1.00) | 1.04 | (0.57,1.89) | 1.48 | (0.73,3.01) | 2.16 | **(1.10,4.22)*** | 0.010 | 0.077 |
|  |  | Female | Ref (1.00) | 1.02 | (0.38,2.72) | 2.37 | **(1.18,4.77)*** | 2.03 | **(1.00,4.11)*** | 0.012 | 0.048 |
| Race |  |  |  |  |  |  |  |  |  |  |  |
|  | Model l | Non-hispanic white | Ref (1.00) | 0.97 | (0.53,1.80) | 1.73 | (0.96,3.12) | 2.08 | **(1.27,3.40)**** | < 0.001 | 0.012 |
|  |  | Others | Ref (1.00) | 1.09 | (0.47,2.56) | 1.65 | (0.90,3.03) | 1.60 | (0.80,3.19) | 0.098 | 0.321 |
|  | Model 2 | Non-hispanic white | Ref (1.00) | 0.91 | (0.48,1.72) | 1.60 | (0.88,2.92) | 1.85 | **(1.10,3.12)*** | 0.002 | 0.063 |
|  |  | Others | Ref (1.00) | 1.07 | (0.46,2.51) | 1.63 | (0.89,2.98) | 1.57 | (0.80,3.10) | 0.103 | 0.336 |
|  | Model 3 | Non-hispanic white | Ref (1.00) | 0.91 | (0.48,1.73) | 1.58 | (0.86,2.88) | 1.82 | **(1.07,3.08)*** | 0.003 | 0.080 |
|  |  | Others | Ref (1.00) | 1.08 | (0.46,2.55) | 1.66 | (0.91,3.01) | 1.58 | (0.80,3.12) | 0.102 | 0.296 |
|  | Model 4 | Non-hispanic white | Ref (1.00) | 1.00 | (0.52,1.93) | 1.80 | (0.96,3.38) | 2.19 | **(1.24,3.87)**** | < 0.001 | 0.022 |
|  |  | Others | Ref (1.00) | 1.17 | (0.49,2.78) | 1.76 | (0.93,3.32) | 1.63 | (0.82,3.24) | 0.074 | 0.241 |
| Exercise |  |  |  |  |  |  |  |  |  |  |  |
|  | Model l | Yes | Ref (1.00) | 0.65 | (0.35,1.22) | 1.17 | (0.60,2.31) | 1.68 | (0.92,3.07) | 0.004 | 0.281 |
|  |  | No | Ref (1.00) | 1.58 | (0.79,3.14) | 2.62 | **(1.38,4.96)**** | 2.19 | **(1.25,3.84)**** | 0.004 | 0.011 |
|  | Model 2 | Yes | Ref (1.00) | 0.61 | (0.32,1.16) | 1.13 | (0.58,2.22) | 1.62 | (0.89,2.97) | 0.005 | 0.350 |
|  |  | No | Ref (1.00) | 1.57 | (0.79,3.11) | 2.57 | **(1.35,4.88)**** | 2.10 | **(1.18,3.74)*** | 0.010 | 0.013 |
|  | Model 3 | Yes | Ref (1.00) | 0.61 | (0.32,1.15) | 1.13 | (0.57,2.24) | 1.61 | (0.88,2.94) | 0.005 | 0.372 |
|  |  | No | Ref (1.00) | 1.60 | (0.79,3.23) | 2.55 | **(1.34,4.84)**** | 2.08 | **(1.15,3.75)*** | 0.014 | 0.014 |
|  | Model 4 | Yes | Ref (1.00) | 0.66 | (0.34,1.27) | 1.27 | (0.61,2.63) | 1.81 | (0.90,3.64) | 0.006 | 0.287 |
|  |  | No | Ref (1.00) | 1.76 | (0.88,3.52) | 2.87 | **(1.49,5.55)**** | 2.54 | **(1.39,4.62)**** | 0.002 | 0.006 |
| Smoking |  |  |  |  |  |  |  |  |  |  |  |
|  | Model l | Yes | Ref (1.00) | 0.63 | (0.20,2.01) | 1.01 | (0.34,2.97) | 1.00 | (0.38,2.68) | 0.720 | 1.000 |
|  |  | No | Ref (1.00) | 0.65 | (0.19,2.24) | 1.13 | **(0.35,3.66)*** | 1.17 | **(0.38,3.63)***** | < 0.001 | 0.001 |
|  | Model 2 | Yes | Ref (1.00) | 0.58 | (0.17,2.01) | 1.00 | (0.34,2.95) | 1.00 | (0.38,2.66) | 0.669 | 1.000 |
|  |  | No | Ref (1.00) | 1.08 | (0.64,1.82) | 1.74 | **(1.02,2.97)*** | 2.17 | **(1.34,3.52)**** | < 0.001 | 0.006 |
|  | Model 3 | Yes | Ref (1.00) | 0.57 | (0.17,1.97) | 1.00 | (0.34,3.00) | 0.99 | (0.36,2.70) | 0.691 | 1.000 |
|  |  | No | Ref (1.00) | 1.07 | (0.63,1.82) | 1.71 | **(1.00,2.93)*** | 2.13 | **(1.30,3.48)**** | < 0.001 | 0.009 |
|  | Model 4 | Yes | Ref (1.00) | 0.65 | (0.19,2.24) | 1.13 | (0.35,3.66) | 1.17 | (0.38,3.63) | 0.540 | 1.000 |
|  |  | No | Ref (1.00) | 1.18 | (0.69,2.01) | 1.96 | **(1.12,3.43)*** | 2.54 | **(1.48,4.36)***** | < 0.001 | 0.003 |
| Drinking |  |  |  |  |  |  |  |  |  |  |  |
|  | Model l | Yes | Ref (1.00) | 1.09 | (0.60,1.97) | 1.81 | **(1.04,3.15)*** | 1.68 | **(1.02,2.76)*** | 0.015 | 0.113 |
|  |  | No | Ref (1.00) | 0.62 | (0.20,1.86) | 1.19 | (0.39,3.62) | 3.07 | **(1.10,8.55)*** | 0.001 | 0.097 |
|  | Model 2 | Yes | Ref (1.00) | 1.03 | (0.57,1.87) | 1.66 | (0.96,2.87) | 1.52 | (0.92,2.51) | 0.043 | 0.209 |
|  |  | No | Ref (1.00) | 0.59 | (0.19,1.79) | 1.10 | (0.36,3.36) | 2.80 | (0.98,7.97) | 0.001 | 0.161 |
|  | Model 3 | Yes | Ref (1.00) | 1.05 | (0.58,1.91) | 1.67 | (0.97,2.89) | 1.53 | (0.92,2.53) | 0.047 | 0.199 |
|  |  | No | Ref (1.00) | 0.57 | (0.19,1.74) | 1.08 | (0.35,3.33) | 2.74 | (0.99,7.61) | 0.001 | 0.159 |
|  | Model 4 | Yes | Ref (1.00) | 1.19 | (0.65,2.16) | 1.95 | **(1.12,3.42)*** | 1.97 | **(1.15,3.39)*** | 0.005 | 0.043 |
|  |  | No | Ref (1.00) | 0.59 | (0.18,1.89) | 1.08 | (0.33,3.54) | 2.47 | (0.85,7.17) | 0.002 | 0.283 |
| T2DM |  |  |  |  |  |  |  |  |  |  |  |
|  | Model l | Yes | Ref (1.00) | 0.60 | (0.21,1.75) | 0.55 | (0.19,1.61) | 0.54 | (0.19,1.54) | 0.408 | 0.739 |
|  |  | No | Ref (1.00) | 1.02 | (0.59,1.78) | 1.87 | **(1.08,3.22)*** | 1.85 | **(1.16,2.95)**** | 0.001 | 0.030 |
|  | Model 2 | Yes | Ref (1.00) | 0.59 | (0.20,1.75) | 0.54 | (0.19,1.54) | 0.53 | (0.19,1.43) | 0.362 | 0.617 |
|  |  | No | Ref (1.00) | 0.99 | (0.56,1.74) | 1.78 | **(1.04,3.07)*** | 1.75 | **(1.09,2.80)*** | 0.002 | 0.063 |
|  | Model 3 | Yes | Ref (1.00) | 0.56 | (0.18,1.68) | 0.56 | (0.19,1.66) | 0.52 | (0.19,1.47) | 0.370 | 0.647 |
|  |  | No | Ref (1.00) | 0.99 | (0.56,1.77) | 1.77 | **(1.03,3.05)*** | 1.73 | **(1.07,2.78)*** | 0.002 | 0.074 |
|  | Model 4 | Yes | Ref (1.00) | 0.60 | (0.21,1.71) | 0.59 | (0.22,1.59) | 0.60 | (0.23,1.55) | 0.535 | 0.872 |
|  |  | No | Ref (1.00) | 1.12 | (0.63,2.00) | 2.11 | **(1.22,3.63)**** | 2.37 | **(1.44,3.90)***** | < 0.001 | 0.003 |

^a^The variables adjusted in each model were the factors mentioned above except the stratification variables.

^b^Data were listed as the weighted odd ratio estimates and 95% confidence intervals, with *p < 0.05, **p < 0.01, ***p < 0.001.

^c^Q, quintile.

^d^Ref, reference.

^e^Tests for trends based on the variables containing the median values for each quartile.

^f^*P*_test_ was the result of Bonfreni correction.

Supplementary Table 25. Association of TyG-WC with angina pectoris stratified by age, sex, race, exercise, smoking, drinking, and T2DM status

|  |  |  | TyG-WC | | | | | | | *P*_trend_^e^ | *P*_test_^f^ |
| --- | --- | --- | --- | --- | --- | --- | --- | --- | --- | --- | --- |
|  |  |  | Q1^c^ | Q2 | | Q3 | | Q4 | |  |  |
|  |  |  |  |  | HR/OR^b^ | 95% CI | HR/OR | 95% CI | HR/OR |  |  |
| Age |  |  |  |  |  |  |  |  |  |  |  |
|  | Model 1^a^ | > 50 | Ref (1.00)^d^ | 0.93 | (0.49,1.76) | 1.32 | (0.78,2.23) | 1.87 | **(1.06,3.29)*** | 0.002 | 0.093 |
|  |  | ≤ 50 | Ref (1.00) | 3.08 | (0.85,11.17) | 4.90 | **(1.36,17.69)*** | 11.96 | **(4.01,35.64)***** | < 0.001 | < 0.001 |
|  | Model 2 | > 50 | Ref (1.00) | 0.89 | (0.47,1.67) | 1.21 | (0.71,2.06) | 1.66 | (0.95,2.89) | 0.007 | 0.217 |
|  |  | ≤ 50 | Ref (1.00) | 2.87 | (0.79,10.36) | 4.43 | **(1.17,16.83)*** | 9.74 | **(3.14,30.25)***** | < 0.001 | < 0.001 |
|  | Model 3 | > 50 | Ref (1.00) | 0.90 | (0.48,1.70) | 1.22 | (0.72,2.08) | 1.72 | (0.99,2.99) | 0.005 | 0.158 |
|  |  | ≤ 50 | Ref (1.00) | 2.84 | (0.78,10.31) | 4.47 | **(1.21,16.49)*** | 9.84 | **(3.13,30.95)***** | < 0.001 | < 0.001 |
|  | Model 4 | > 50 | Ref (1.00) | 0.93 | (0.50,1.72) | 1.23 | (0.73,2.07) | 1.63 | (0.94,2.84) | 0.016 | 0.252 |
|  |  | ≤ 50 | Ref (1.00) | 3.16 | (0.88,11.41) | 5.16 | **(1.42,18.80)*** | 10.79 | **(3.17,36.76)***** | < 0.001 | 0.001 |
| Sex |  |  |  |  |  |  |  |  |  |  |  |
|  | Model l | Male | Ref (1.00) | 0.94 | (0.36,2.50) | 1.11 | (0.48,2.59) | 2.03 | (0.87,4.74) | 0.002 | 0.299 |
|  |  | Female | Ref (1.00) | 1.13 | (0.53,2.40) | 2.03 | (0.98,4.19) | 3.01 | **(1.29,7.05)*** | 0.004 | 0.035 |
|  | Model 2 | Male | Ref (1.00) | 0.93 | (0.35,2.45) | 1.07 | (0.45,2.53) | 1.94 | (0.82,4.57) | 0.004 | 0.382 |
|  |  | Female | Ref (1.00) | 1.07 | (0.50,2.30) | 1.89 | (0.91,3.91) | 2.62 | **(1.14,6.05)*** | 0.010 | 0.073 |
|  | Model 3 | Male | Ref (1.00) | 0.92 | (0.35,2.40) | 1.05 | (0.45,2.47) | 1.90 | (0.82,4.42) | 0.004 | 0.400 |
|  |  | Female | Ref (1.00) | 1.08 | (0.50,2.30) | 1.88 | (0.93,3.80) | 2.65 | **(1.17,6.01)*** | 0.008 | 0.061 |
|  | Model 4 | Male | Ref (1.00) | 1.03 | (0.40,2.63) | 1.18 | (0.50,2.78) | 2.10 | (0.88,5.04) | 0.006 | 0.284 |
|  |  | Female | Ref (1.00) | 1.10 | (0.52,2.33) | 1.79 | (0.88,3.64) | 2.25 | (0.98,5.14) | 0.031 | 0.165 |
| Race |  |  |  |  |  |  |  |  |  |  |  |
|  | Model l | Non-hispanic white | Ref (1.00) | 0.89 | (0.47,1.69) | 1.39 | (0.86,2.24) | 2.21 | **(1.20,4.05)*** | 0.001 | 0.034 |
|  |  | Others | Ref (1.00) | 2.50 | (0.93,6.71) | 2.36 | (0.97,5.78) | 4.98 | **(2.03,12.23)**** | < 0.001 | 0.002 |
|  | Model 2 | Non-hispanic white | Ref (1.00) | 0.87 | (0.46,1.66) | 1.30 | (0.81,2.11) | 2.01 | **(1.11,3.65)*** | 0.002 | 0.066 |
|  |  | Others | Ref (1.00) | 2.49 | (0.93,6.67) | 2.33 | (0.96,5.68) | 4.81 | **(1.94,11.94)**** | 0.001 | 0.003 |
|  | Model 3 | Non-hispanic white | Ref (1.00) | 0.87 | (0.46,1.65) | 1.28 | (0.79,2.07) | 1.98 | **(1.09,3.57)*** | 0.003 | 0.074 |
|  |  | Others | Ref (1.00) | 2.53 | (0.95,6.72) | 2.37 | (0.98,5.71) | 4.94 | **(2.00,12.24)**** | 0.001 | 0.002 |
|  | Model 4 | Non-hispanic white | Ref (1.00) | 0.95 | (0.50,1.77) | 1.37 | (0.85,2.21) | 2.01 | **(1.14,3.57)*** | 0.003 | 0.051 |
|  |  | Others | Ref (1.00) | 2.70 | (1.05,6.99) | 2.46 | **(1.01,5.98)*** | 4.67 | **(1.85,11.83)**** | 0.003 | 0.004 |
| Exercise |  |  |  |  |  |  |  |  |  |  |  |
|  | Model l | Yes | Ref (1.00) | 1.41 | (0.50,3.95) | 1.56 | (0.72,3.37) | 3.07 | **(1.31,7.20)*** | < 0.001 | 0.031 |
|  |  | No | Ref (1.00) | 0.80 | (0.38,1.70) | 1.48 | (0.83,2.63) | 1.93 | (0.86,4.29) | 0.031 | 0.325 |
|  | Model 2 | Yes | Ref (1.00) | 1.43 | (0.52,3.98) | 1.60 | (0.74,3.45) | 3.13 | **(1.35,7.24)**** | < 0.001 | 0.025 |
|  |  | No | Ref (1.00) | 0.79 | (0.37,1.69) | 1.39 | (0.76,2.54) | 1.83 | (0.80,4.21) | 0.051 | 0.449 |
|  | Model 3 | Yes | Ref (1.00) | 1.44 | (0.52,4.00) | 1.60 | (0.74,3.48) | 3.14 | **(1.36,7.25)**** | < 0.001 | 0.024 |
|  |  | No | Ref (1.00) | 0.78 | (0.36,1.69) | 1.37 | (0.73,2.55) | 1.78 | (0.76,4.15) | 0.064 | 0.538 |
|  | Model 4 | Yes | Ref (1.00) | 1.58 | (0.58,4.30) | 1.66 | (0.77,3.58) | 2.97 | **(1.26,7.00)*** | 0.003 | 0.040 |
|  |  | No | Ref (1.00) | 0.83 | (0.38,1.81) | 1.46 | (0.78,2.74) | 1.90 | (0.83,4.35) | 0.042 | 0.375 |
| Smoking |  |  |  |  |  |  |  |  |  |  |  |
|  | Model l | Yes | Ref (1.00) | 0.60 | (0.20,1.73) | 0.38 | (0.13,1.16) | 1.60 | (0.62,4.18) | 0.166 | 0.263 |
|  |  | No | Ref (1.00) | 1.53 | (0.85,2.75) | 2.43 | **(1.46,4.06)**** | 3.42 | **(2.06,5.68)***** | < 0.001 | < 0.001 |
|  | Model 2 | Yes | Ref (1.00) | 0.57 | (0.21,1.60) | 0.37 | (0.12,1.14) | 1.66 | (0.67,4.10) | 0.129 | 0.247 |
|  |  | No | Ref (1.00) | 1.49 | (0.82,2.69) | 2.28 | **(1.34,3.88)**** | 3.13 | **(1.86,5.26)***** | < 0.001 | < 0.001 |
|  | Model 3 | Yes | Ref (1.00) | 0.59 | (0.21,1.63) | 0.37 | (0.12,1.18) | 1.67 | (0.67,4.17) | 0.128 | 0.275 |
|  |  | No | Ref (1.00) | 1.47 | (0.81,2.67) | 2.23 | **(1.30,3.82)**** | 3.07 | **(1.83,5.15)***** | < 0.001 | < 0.001 |
|  | Model 4 | Yes | Ref (1.00) | 0.65 | (0.24,1.78) | 0.40 | (0.12,1.30) | 1.83 | (0.68,4.92) | 0.136 | 0.382 |
|  |  | No | Ref (1.00) | 1.55 | (0.86,2.81) | 2.29 | **(1.31,4.00)**** | 2.97 | **(1.73,5.12)***** | < 0.001 | < 0.001 |
| Drinking |  |  |  |  |  |  |  |  |  |  |  |
|  | Model l | Yes | Ref (1.00) | 0.97 | (0.50,1.88) | 1.36 | (0.75,2.46) | 2.38 | **(1.22,4.65)*** | 0.001 | 0.036 |
|  |  | No | Ref (1.00) | 1.58 | (0.54,4.65) | 2.11 | (0.83,5.34) | 3.69 | **(1.44,9.43)**** | 0.002 | 0.021 |
|  | Model 2 | Yes | Ref (1.00) | 0.95 | (0.49,1.82) | 1.30 | (0.72,2.35) | 2.22 | **(1.16,4.24)*** | 0.002 | 0.050 |
|  |  | No | Ref (1.00) | 1.62 | (0.54,4.85) | 2.03 | (0.81,5.11) | 3.55 | **(1.39,9.05)**** | 0.003 | 0.025 |
|  | Model 3 | Yes | Ref (1.00) | 0.96 | (0.50,1.84) | 1.32 | (0.73,2.39) | 2.28 | **(1.19,4.33)*** | 0.002 | 0.039 |
|  |  | No | Ref (1.00) | 1.59 | (0.54,4.70) | 2.01 | (0.81,5.02) | 3.39 | **(1.32,8.74)*** | 0.005 | 0.036 |
|  | Model 4 | Yes | Ref (1.00) | 1.09 | (0.57,2.08) | 1.49 | (0.81,2.75) | 2.51 | **(1.31,4.80)**** | 0.001 | 0.018 |
|  |  | No | Ref (1.00) | 1.48 | (0.51,4.28) | 1.80 | (0.73,4.45) | 2.84 | **(1.11,7.22)*** | 0.017 | 0.087 |
| T2DM |  |  |  |  |  |  |  |  |  |  |  |
|  | Model l | Yes | Ref (1.00) | 0.57 | (0.13,2.62) | 0.44 | (0.11,1.77) | 0.70 | (0.17,2.97) | 0.633 | 0.730 |
|  |  | No | Ref (1.00) | 1.05 | (0.59,1.87) | 1.60 | **(1.00,2.54)*** | 2.27 | **(1.28,4.05)**** | 0.001 | 0.017 |
|  | Model 2 | Yes | Ref (1.00) | 0.57 | (0.13,2.43) | 0.44 | (0.12,1.69) | 0.67 | (0.17,2.59) | 0.746 | 0.693 |
|  |  | No | Ref (1.00) | 1.04 | (0.58,1.84) | 1.53 | (0.96,2.44) | 2.17 | **(1.23,3.82)**** | 0.002 | 0.024 |
|  | Model 3 | Yes | Ref (1.00) | 0.59 | (0.13,2.59) | 0.45 | (0.11,1.81) | 0.70 | (0.17,2.79) | 0.709 | 0.780 |
|  |  | No | Ref (1.00) | 1.03 | (0.58,1.84) | 1.51 | (0.95,2.42) | 2.15 | **(1.21,3.82)**** | 0.002 | 0.028 |
|  | Model 4 | Yes | Ref (1.00) | 0.65 | (0.17,2.47) | 0.48 | (0.14,1.61) | 0.71 | (0.21,2.39) | 0.794 | 0.700 |
|  |  | No | Ref (1.00) | 1.11 | (0.62,2.00) | 1.65 | **(1.01,2.68)*** | 2.37 | **(1.32,4.24)**** | 0.001 | 0.013 |

^a^The variables adjusted in each model were the factors mentioned above except the stratification variables.

^b^Data were listed as the weighted odd ratio estimates and 95% confidence intervals, with *p < 0.05, **p < 0.01, ***p < 0.001.

^c^Q, quintile.

^d^Ref, reference.

^e^Tests for trends based on the variables containing the median values for each quartile.

^f^*P*_test_ was the result of Bonfreni correction.

Supplementary Table 26. Association of TyG-WtHR with angina pectoris stratified by age, sex, race, exercise, smoking, drinking, and T2DM status

|  |  |  | TyG-WtHR | | | | | | | *P*_trend_^e^ | *P*_test_^f^ |
| --- | --- | --- | --- | --- | --- | --- | --- | --- | --- | --- | --- |
|  |  |  | Q1^c^ | Q2 | | Q3 | | Q4 | |  |  |
|  |  |  |  | HR/OR^b^ | 95% CI | HR/OR | 95% CI | HR/OR | 95% CI |  |  |
| Age |  |  |  |  |  |  |  |  |  |  |  |
|  | Model 1^a^ | > 50 | Ref (1.00)^d^ | 0.74 | (0.42,1.28) | 1.49 | (0.92,2.42) | 1.87 | **(1.15,3.02)*** | < 0.001 | 0.035 |
|  |  | ≤ 50 | Ref (1.00) | 4.70 | **(1.30,17.01)*** | 8.80 | **(2.42,32.00)**** | 17.01 | **(5.08,56.98)***** | < 0.001 | < 0.001 |
|  | Model 2 | > 50 | Ref (1.00) | 0.69 | (0.39,1.21) | 1.35 | (0.82,2.22) | 1.61 | **(1.00,2.58)*** | 0.001 | 0.150 |
|  |  | ≤ 50 | Ref (1.00) | 4.44 | **(1.21,16.23)*** | 7.93 | **(2.10,30.01)**** | 14.01 | **(3.80,51.70)***** | < 0.001 | < 0.001 |
|  | Model 3 | > 50 | Ref (1.00) | 0.67 | (0.38,1.18) | 1.31 | (0.79,2.17) | 1.58 | (0.99,2.52) | 0.001 | 0.161 |
|  |  | ≤ 50 | Ref (1.00) | 4.31 | **(1.16,15.99)*** | 8.03 | **(2.15,30.02)**** | 13.66 | **(3.73,50.06)***** | < 0.001 | < 0.001 |
|  | Model 4 | > 50 | Ref (1.00) | 0.71 | (0.41,1.23) | 1.36 | (0.83,2.21) | 1.55 | (0.95,2.52) | 0.006 | 0.236 |
|  |  | ≤ 50 | Ref (1.00) | 4.93 | **(1.34,18.16)*** | 9.21 | **(2.51,33.83)**** | 15.07 | **(3.51,64.62)***** | 0.001 | 0.001 |
| Sex |  |  |  |  |  |  |  |  |  |  |  |
|  | Model l | Male | Ref (1.00) | 0.95 | (0.51,1.75) | 1.83 | (1.00,3.35) | 2.26 | **(1.22,4.20)*** | 0.001 | 0.031 |
|  |  | Female | Ref (1.00) | 0.89 | (0.36,2.24) | 1.60 | (0.69,3.70) | 2.74 | **(1.14,6.63)*** | 0.004 | 0.076 |
|  | Model 2 | Male | Ref (1.00) | 0.92 | (0.50,1.71) | 1.75 | (0.94,3.25) | 2.14 | **(1.13,4.06)*** | 0.001 | 0.061 |
|  |  | Female | Ref (1.00) | 0.82 | (0.32,2.07) | 1.46 | (0.63,3.38) | 2.32 | (0.98,5.53) | 0.009 | 0.170 |
|  | Model 3 | Male | Ref (1.00) | 0.90 | (0.49,1.68) | 1.71 | (0.91,3.18) | 2.08 | **(1.09,3.95)*** | 0.002 | 0.078 |
|  |  | Female | Ref (1.00) | 0.81 | (0.32,2.03) | 1.45 | (0.64,3.28) | 2.30 | (1.00,5.31) | 0.007 | 0.153 |
|  | Model 4 | Male | Ref (1.00) | 1.01 | (0.54,1.86) | 1.87 | (0.98,3.58) | 2.30 | **(1.14,4.64)*** | 0.004 | 0.062 |
|  |  | Female | Ref (1.00) | 0.84 | (0.34,2.08) | 1.44 | (0.65,3.20) | 2.05 | (0.89,4.74) | 0.022 | 0.278 |
| Race |  |  |  |  |  |  |  |  |  |  |  |
|  | Model l | Non-hispanic white | Ref (1.00) | 0.79 | (0.46,1.37) | 1.64 | **(1.04,2.60)*** | 2.16 | **(1.27,3.67)**** | < 0.001 | 0.015 |
|  |  | Others | Ref (1.00) | 1.81 | (0.67,4.83) | 2.00 | (0.77,5.23) | 4.16 | **(1.81,9.60)**** | < 0.001 | 0.003 |
|  | Model 2 | Non-hispanic white | Ref (1.00) | 0.75 | (0.43,1.30) | 1.52 | (0.95,2.43) | 1.91 | **(1.13,3.25)*** | 0.001 | 0.050 |
|  |  | Others | Ref (1.00) | 1.77 | (0.67,4.72) | 1.96 | (0.76,5.04) | 4.04 | **(1.77,9.23)**** | < 0.001 | 0.003 |
|  | Model 3 | Non-hispanic white | Ref (1.00) | 0.73 | (0.42,1.28) | 1.47 | (0.91,2.38) | 1.85 | **(1.09,3.12)*** | 0.001 | 0.069 |
|  |  | Others | Ref (1.00) | 1.77 | (0.67,4.66) | 1.99 | (0.78,5.05) | 4.08 | **(1.80,9.27)**** | < 0.001 | 0.003 |
|  | Model 4 | Non-hispanic white | Ref (1.00) | 0.80 | (0.46,1.40) | 1.58 | (0.97,2.57) | 1.92 | **(1.13,3.26)*** | 0.001 | 0.049 |
|  |  | Others | Ref (1.00) | 1.90 | (0.72,5.01) | 2.09 | (0.87,5.01) | 4.04 | **(1.71,9.52)**** | < 0.001 | 0.005 |
| Exercise |  |  |  |  |  |  |  |  |  |  |  |
|  | Model l | Yes | Ref (1.00) | 0.99 | (0.48,2.04) | 1.46 | (0.79,2.70) | 2.76 | **(1.47,5.20)**** | < 0.001 | 0.006 |
|  |  | No | Ref (1.00) | 0.87 | (0.41,1.85) | 1.99 | **(1.14,3.48)*** | 1.85 | (0.83,4.13) | 0.027 | 0.050 |
|  | Model 2 | Yes | Ref (1.00) | 0.94 | (0.45,1.97) | 1.44 | (0.78,2.64) | 2.72 | **(1.44,5.12)**** | < 0.001 | 0.007 |
|  |  | No | Ref (1.00) | 0.85 | (0.40,1.81) | 1.86 | **(1.03,3.34)*** | 1.74 | (0.76,4.01) | 0.054 | 0.118 |
|  | Model 3 | Yes | Ref (1.00) | 0.92 | (0.44,1.94) | 1.42 | (0.77,2.62) | 2.69 | **(1.43,5.04)**** | < 0.001 | 0.007 |
|  |  | No | Ref (1.00) | 0.82 | (0.38,1.78) | 1.80 | (0.98,3.30) | 1.63 | (0.70,3.83) | 0.079 | 0.169 |
|  | Model 4 | Yes | Ref (1.00) | 0.99 | (0.48,2.05) | 1.49 | (0.81,2.75) | 2.60 | **(1.32,5.15)**** | 0.001 | 0.019 |
|  |  | No | Ref (1.00) | 0.88 | (0.40,1.92) | 1.93 | **(1.05,3.54)*** | 1.77 | (0.78,4.04) | 0.046 | 0.102 |
| Smoking |  |  |  |  |  |  |  |  |  |  |  |
|  | Model l | Yes | Ref (1.00) | 0.70 | (0.25,1.97) | 1.02 | (0.34,3.09) | 1.72 | (0.60,4.87) | 0.188 | 0.922 |
|  |  | No | Ref (1.00) | 1.17 | (0.70,1.96) | 2.25 | **(1.27,4.00)**** | 3.11 | **(1.96,4.93)***** | < 0.001 | < 0.001 |
|  | Model 2 | Yes | Ref (1.00) | 0.67 | (0.23,1.94) | 1.05 | (0.37,3.01) | 1.76 | (0.65,4.75) | 0.157 | 0.778 |
|  |  | No | Ref (1.00) | 1.12 | (0.67,1.89) | 2.09 | **(1.16,3.79)*** | 2.82 | **(1.77,4.51)***** | < 0.001 | < 0.001 |
|  | Model 3 | Yes | Ref (1.00) | 0.69 | (0.24,1.98) | 1.08 | (0.38,3.07) | 1.77 | (0.67,4.70) | 0.153 | 0.750 |
|  |  | No | Ref (1.00) | 1.08 | (0.64,1.83) | 2.01 | **(1.10,3.68)*** | 2.70 | **(1.69,4.31)***** | < 0.001 | < 0.001 |
|  | Model 4 | Yes | Ref (1.00) | 0.78 | (0.29,2.16) | 1.21 | (0.41,3.54) | 1.95 | (0.66,5.82) | 0.17 | 0.679 |
|  |  | No | Ref (1.00) | 1.16 | (0.69,1.94) | 2.12 | **(1.15,3.91)*** | 2.69 | **(1.62,4.47)***** | < 0.001 | 0.001 |
| Drinking |  |  |  |  |  |  |  |  |  |  |  |
|  | Model l | Yes | Ref (1.00) | 0.88 | (0.51,1.50) | 1.60 | (0.95,2.71) | 2.42 | **(1.35,4.36)**** | < 0.001 | 0.011 |
|  |  | No | Ref (1.00) | 1.47 | (0.43,4.99) | 2.75 | (0.93,8.13) | 3.77 | **(1.44,9.86)**** | 0.001 | 0.022 |
|  | Model 2 | Yes | Ref (1.00) | 0.85 | (0.50,1.44) | 1.54 | (0.90,2.61) | 2.24 | **(1.25,4.00)**** | 0.001 | 0.021 |
|  |  | No | Ref (1.00) | 1.49 | (0.43,5.16) | 2.70 | (0.92,7.93) | 3.65 | **(1.39,9.60)**** | 0.001 | 0.027 |
|  | Model 3 | Yes | Ref (1.00) | 0.85 | (0.50,1.45) | 1.54 | (0.90,2.65) | 2.25 | **(1.26,4.02)**** | 0.001 | 0.020 |
|  |  | No | Ref (1.00) | 1.42 | (0.40,5.09) | 2.55 | (0.86,7.57) | 3.46 | **(1.30,9.20)*** | 0.002 | 0.041 |
|  | Model 4 | Yes | Ref (1.00) | 0.96 | (0.57,1.63) | 1.76 | **(1.01,3.05)*** | 2.49 | **(1.37,4.55)**** | 0.001 | 0.010 |
|  |  | No | Ref (1.00) | 1.31 | (0.37,4.59) | 2.23 | (0.82,6.08) | 2.86 | **(1.09,7.54)*** | 0.010 | 0.101 |
| T2DM |  |  |  |  |  |  |  |  |  |  |  |
|  | Model l | Yes | Ref (1.00) | 0.35 | (0.09,1.33) | 0.50 | (0.12,2.09) | 0.54 | (0.13,2.19) | 0.850 | 0.360 |
|  |  | No | Ref (1.00) | 0.93 | (0.53,1.64) | 1.70 | **(1.08,2.70)*** | 2.24 | **(1.29,3.91)**** | < 0.001 | 0.014 |
|  | Model 2 | Yes | Ref (1.00) | 0.33 | (0.09,1.28) | 0.50 | (0.13,1.92) | 0.51 | (0.14,1.91) | 0.966 | 0.324 |
|  |  | No | Ref (1.00) | 0.90 | (0.52,1.57) | 1.61 | **(1.02,2.56)*** | 2.11 | **(1.23,3.63)**** | 0.001 | 0.022 |
|  | Model 3 | Yes | Ref (1.00) | 0.32 | (0.08,1.21) | 0.49 | (0.13,1.87) | 0.50 | (0.13,1.90) | 0.922 | 0.272 |
|  |  | No | Ref (1.00) | 0.88 | (0.50,1.56) | 1.58 | (0.99,2.54) | 2.06 | **(1.19,3.56)*** | 0.001 | 0.032 |
|  | Model 4 | Yes | Ref (1.00) | 0.35 | (0.11,1.12) | 0.51 | (0.15,1.70) | 0.53 | (0.17,1.69) | 0.924 | 0.228 |
|  |  | No | Ref (1.00) | 0.96 | (0.54,1.70) | 1.76 | **(1.09,2.84)*** | 2.30 | **(1.32,4.03)**** | < 0.001 | 0.012 |

^a^The variables adjusted in each model were the factors mentioned above except the stratification variables.

^b^Data were listed as the weighted odd ratio estimates and 95% confidence intervals, with *p < 0.05, **p < 0.01, ***p < 0.001.

^c^Q, quintile.

^d^Ref, reference.

^e^Tests for trends based on the variables containing the median values for each quartile.

^f^*P*_test_ was the result of Bonfreni correction.

Supplementary Table 27. Association of TyG-BMI with angina pectoris stratified by age, sex, race, exercise, smoking, drinking, and T2DM status

|  |  |  | TyG-BMI | | | | | | | *P*_trend_^e^ | *P*_test_^f^ |
| --- | --- | --- | --- | --- | --- | --- | --- | --- | --- | --- | --- |
|  |  |  | Q1^c^ | Q2 | | Q3 | | Q4 | |  |  |
|  |  |  |  | HR/OR^b^ | 95% CI | HR/OR | 95% CI | HR/OR | 95% CI |  |  |
| Age |  |  |  |  |  |  |  |  |  |  |  |
|  | Model 1^a^ | > 50 | Ref (1.00)^d^ | 0.76 | (0.48,1.20) | 1.31 | (0.80,2.14) | 1.45 | (0.96,2.19) | 0.009 | 0.238 |
|  |  | ≤ 50 | Ref (1.00) | 3.55 | (0.93,13.60) | 6.49 | **(1.84,22.94)**** | 11.36 | **(3.47,37.18)***** | < 0.001 | < 0.001 |
|  | Model 2 | > 50 | Ref (1.00) | 0.73 | (0.46,1.15) | 1.24 | (0.76,2.02) | 1.32 | (0.88,1.97) | 0.022 | 0.517 |
|  |  | ≤ 50 | Ref (1.00) | 3.44 | (0.89,13.24) | 5.97 | **(1.63,21.89)**** | 9.62 | **(2.88,32.16)***** | < 0.001 | 0.001 |
|  | Model 3 | > 50 | Ref (1.00) | 0.71 | (0.45,1.13) | 1.24 | (0.76,2.03) | 1.35 | (0.91,2.02) | 0.012 | 0.413 |
|  |  | ≤ 50 | Ref (1.00) | 3.39 | (0.88,13.08) | 6.00 | **(1.66,21.75)**** | 9.70 | **(2.90,32.52)***** | < 0.001 | 0.001 |
|  | Model 4 | > 50 | Ref (1.00) | 0.75 | (0.47,1.18) | 1.27 | (0.79,2.05) | 1.29 | (0.83,2.00) | 0.054 | 0.638 |
|  |  | ≤ 50 | Ref (1.00) | 3.61 | (0.95,13.70) | 6.82 | **(1.96,23.74)**** | 9.88 | **(2.62,37.26)**** | 0.001 | 0.003 |
| Sex |  |  |  |  |  |  |  |  |  |  |  |
|  | Model l | Male | Ref (1.00) | 0.89 | (0.50,1.58) | 1.54 | (0.85,2.77) | 2.17 | **(1.30,3.60)**** | < 0.001 | 0.010 |
|  |  | Female | Ref (1.00) | 1.04 | (0.46,2.34) | 1.93 | (0.83,4.47) | 2.62 | **(1.23,5.58)*** | 0.002 | 0.038 |
|  | Model 2 | Male | Ref (1.00) | 0.85 | (0.48,1.53) | 1.48 | (0.82,2.67) | 2.06 | **(1.23,3.43)**** | < 0.001 | 0.018 |
|  |  | Female | Ref (1.00) | 0.98 | (0.44,2.22) | 1.77 | (0.77,4.04) | 2.30 | **(1.10,4.82)*** | 0.006 | 0.083 |
|  | Model 3 | Male | Ref (1.00) | 0.84 | (0.47,1.49) | 1.46 | (0.81,2.65) | 2.01 | **(1.21,3.34)**** | < 0.001 | 0.022 |
|  |  | Female | Ref (1.00) | 0.97 | (0.43,2.15) | 1.73 | (0.78,3.86) | 2.30 | **(1.12,4.73)*** | 0.005 | 0.070 |
|  | Model 4 | Male | Ref (1.00) | 0.90 | (0.51,1.60) | 1.53 | (0.84,2.78) | 2.10 | **(1.19,3.70)*** | 0.001 | 0.032 |
|  |  | Female | Ref (1.00) | 0.96 | (0.44,2.11) | 1.64 | (0.74,3.61) | 1.95 | (0.95,4.02) | 0.022 | 0.209 |
| Race |  |  |  |  |  |  |  |  |  |  |  |
|  | Model l | Non-hispanic white | Ref (1.00) | 0.83 | (0.51,1.33) | 1.74 | **(1.08,2.79)*** | 2.15 | **(1.33,3.46)**** | < 0.001 | 0.006 |
|  |  | Others | Ref (1.00) | 1.69 | (0.64,4.46) | 1.28 | (0.60,2.73) | 3.32 | **(1.52,7.26)**** | 0.002 | 0.009 |
|  | Model 2 | Non-hispanic white | Ref (1.00) | 0.78 | (0.48,1.25) | 1.62 | **(1.02,2.58)*** | 1.93 | **(1.22,3.06)**** | < 0.001 | 0.016 |
|  |  | Others | Ref (1.00) | 1.66 | (0.64,4.34) | 1.24 | (0.58,2.65) | 3.21 | **(1.49,6.92)**** | 0.002 | 0.010 |
|  | Model 3 | Non-hispanic white | Ref (1.00) | 0.76 | (0.47,1.22) | 1.59 | (0.99,2.54) | 1.89 | **(1.20,2.98)**** | < 0.001 | 0.019 |
|  |  | Others | Ref (1.00) | 1.66 | (0.63,4.33) | 1.25 | (0.59,2.65) | 3.24 | **(1.49,7.02)**** | 0.002 | 0.010 |
|  | Model 4 | Non-hispanic white | Ref (1.00) | 0.80 | (0.50,1.29) | 1.63 | **(1.04,2.57)*** | 1.85 | **(1.15,2.95)*** | 0.001 | 0.033 |
|  |  | Others | Ref (1.00) | 1.67 | (0.64,4.37) | 1.25 | (0.58,2.68) | 2.97 | **(1.37,6.47)**** | 0.006 | 0.020 |
| Exercise |  |  |  |  |  |  |  |  |  |  |  |
|  | Model l | Yes | Ref (1.00) | 1.21 | (0.63,2.32) | 1.70 | (0.90,3.20) | 3.01 | **(1.66,5.47)***** | < 0.001 | 0.001 |
|  |  | No | Ref (1.00) | 0.79 | (0.41,1.50) | 1.77 | **(1.01,3.10)*** | 1.60 | (0.75,3.43) | 0.061 | 0.144 |
|  | Model 2 | Yes | Ref (1.00) | 1.17 | (0.60,2.26) | 1.68 | (0.90,3.16) | 3.00 | **(1.65,5.43)***** | < 0.001 | 0.001 |
|  |  | No | Ref (1.00) | 0.75 | (0.39,1.47) | 1.67 | (0.93,3.00) | 1.50 | (0.68,3.30) | 0.100 | 0.262 |
|  | Model 3 | Yes | Ref (1.00) | 1.14 | (0.59,2.22) | 1.70 | (0.90,3.19) | 2.99 | **(1.64,5.45)***** | < 0.001 | 0.001 |
|  |  | No | Ref (1.00) | 0.74 | (0.38,1.44) | 1.61 | (0.88,2.96) | 1.43 | (0.65,3.16) | 0.128 | 0.368 |
|  | Model 4 | Yes | Ref (1.00) | 1.16 | (0.60,2.25) | 1.70 | (0.92,3.17) | 2.66 | **(1.38,5.14)**** | 0.001 | 0.011 |
|  |  | No | Ref (1.00) | 0.77 | (0.39,1.54) | 1.66 | (0.91,3.01) | 1.49 | (0.69,3.23) | 0.097 | 0.290 |
| Smoking |  |  |  |  |  |  |  |  |  |  |  |
|  | Model l | Yes | Ref (1.00) | 0.40 | (0.14,1.13) | 0.91 | (0.37,2.24) | 1.92 | (0.80,4.58) | 0.053 | 0.250 |
|  |  | No | Ref (1.00) | 1.37 | (0.84,2.24) | 2.26 | **(1.47,3.46)***** | 2.84 | **(1.81,4.45)***** | < 0.001 | < 0.001 |
|  | Model 2 | Yes | Ref (1.00) | 0.35 | (0.11,1.11) | 0.96 | (0.41,2.24) | 1.96 | (0.86,4.49) | 0.037 | 0.224 |
|  |  | No | Ref (1.00) | 1.31 | (0.79,2.16) | 2.12 | **(1.37,3.27)**** | 2.58 | **(1.63,4.08)***** | < 0.001 | < 0.001 |
|  | Model 3 | Yes | Ref (1.00) | 0.35 | (0.11,1.10) | 0.96 | (0.40,2.27) | 1.94 | (0.85,4.46) | 0.037 | 0.215 |
|  |  | No | Ref (1.00) | 1.27 | (0.77,2.09) | 2.06 | **(1.33,3.19)**** | 2.51 | **(1.60,3.93)***** | < 0.001 | < 0.001 |
|  | Model 4 | Yes | Ref (1.00) | 0.38 | (0.12,1.18) | 1.06 | (0.45,2.51) | 2.10 | (0.78,5.65) | 0.053 | 0.278 |
|  |  | No | Ref (1.00) | 1.30 | (0.79,2.16) | 2.04 | **(1.32,3.17)**** | 2.34 | **(1.42,3.85)**** | < 0.001 | 0.003 |
| Drinking |  |  |  |  |  |  |  |  |  |  |  |
|  | Model l | Yes | Ref (1.00) | 0.75 | (0.46,1.22) | 1.47 | (0.90,2.43) | 2.10 | **(1.21,3.64)**** | 0.001 | 0.026 |
|  |  | No | Ref (1.00) | 2.41 | (0.88,6.62) | 3.14 | **(1.20,8.19)*** | 4.43 | **(1.80,10.89)**** | 0.001 | 0.004 |
|  | Model 2 | Yes | Ref (1.00) | 0.74 | (0.46,1.20) | 1.47 | (0.90,2.41) | 1.99 | **(1.16,3.41)*** | 0.001 | 0.038 |
|  |  | No | Ref (1.00) | 2.45 | (0.88,6.77) | 3.11 | **(1.19,8.12)*** | 4.42 | **(1.80,10.88)**** | 0.002 | 0.004 |
|  | Model 3 | Yes | Ref (1.00) | 0.72 | (0.45,1.17) | 1.48 | (0.90,2.42) | 1.99 | **(1.17,3.39)*** | 0.001 | 0.035 |
|  |  | No | Ref (1.00) | 2.36 | (0.85,6.59) | 3.06 | **(1.17,8.01)*** | 4.18 | **(1.68,10.38)**** | 0.003 | 0.007 |
|  | Model 4 | Yes | Ref (1.00) | 0.78 | (0.48,1.28) | 1.58 | (0.96,2.61) | 2.04 | **(1.16,3.59)*** | 0.002 | 0.042 |
|  |  | No | Ref (1.00) | 2.26 | (0.83,6.17) | 2.89 | **(1.12,7.49)*** | 3.71 | **(1.46,9.44)**** | 0.009 | 0.019 |
| T2DM |  |  |  |  |  |  |  |  |  |  |  |
|  | Model l | Yes | Ref (1.00) | 0.98 | (0.31,3.14) | 1.35 | (0.48,3.83) | 0.95 | (0.30,3.07) | 0.726 | 1.000 |
|  |  | No | Ref (1.00) | 0.85 | (0.52,1.38) | 1.46 | (0.96,2.23) | 2.34 | **(1.38,3.95)**** | < 0.001 | 0.006 |
|  | Model 2 | Yes | Ref (1.00) | 0.90 | (0.26,3.09) | 1.29 | (0.45,3.70) | 0.86 | (0.27,2.77) | 0.584 | 1.000 |
|  |  | No | Ref (1.00) | 0.82 | (0.51,1.33) | 1.41 | (0.93,2.13) | 2.22 | **(1.33,3.69)**** | < 0.001 | 0.007 |
|  | Model 3 | Yes | Ref (1.00) | 0.89 | (0.25,3.12) | 1.28 | (0.43,3.78) | 0.87 | (0.26,2.91) | 0.639 | 1.000 |
|  |  | No | Ref (1.00) | 0.81 | (0.50,1.32) | 1.39 | (0.92,2.12) | 2.19 | **(1.31,3.66)**** | < 0.001 | 0.009 |
|  | Model 4 | Yes | Ref (1.00) | 0.87 | (0.27,2.83) | 1.22 | (0.45,3.29) | 0.83 | (0.28,2.49) | 0.563 | 1.000 |
|  |  | No | Ref (1.00) | 0.85 | (0.52,1.39) | 1.49 | (0.98,2.27) | 2.31 | **(1.39,3.85)**** | < 0.001 | 0.005 |

^a^The variables adjusted in each model were the factors mentioned above except the stratification variables.

^b^Data were listed as the weighted odd ratio estimates and 95% confidence intervals, with *p < 0.05, **p < 0.01, ***p < 0.001.

^c^Q, quintile.

^d^Ref, reference.

^e^Tests for trends based on the variables containing the median values for each quartile.

^f^*P*_test_ was the result of Bonfreni correction.

Supplementary Table 28. Association of TyG with coronary heart disease stratified by age, sex, race, exercise, smoking, drinking, and T2DM status

|  |  |  | TyG | | | | | | | *P*_trend_^e^ | *P*_test_^f^ |
| --- | --- | --- | --- | --- | --- | --- | --- | --- | --- | --- | --- |
|  |  |  | Q1^c^ | Q2 | | Q3 | | Q4 | |  |  |
|  |  |  |  | HR/OR^b^ | 95% CI | HR/OR | 95% CI | HR/OR | 95% CI |  |  |
| Age |  |  |  |  |  |  |  |  |  |  |  |
|  | Model 1^a^ | > 50 | Ref (1.00)^d^ | 1.17 | (0.75,1.82) | 1.45 | (0.97,2.17) | 1.89 | **(1.28,2.79)***** | 0.001 | 0.005 |
|  |  | ≤ 50 | Ref (1.00) | 0.99 | (0.30,3.26) | 3.22 | (0.84,12.28) | 5.45 | **(1.88,15.75)**** | < 0.001 | 0.006 |
|  | Model 2 | > 50 | Ref (1.00) | 1.12 | (0.71,1.75) | 1.38 | (0.92,2.05) | 1.78 | **(1.21,2.63)**** | 0.002 | 0.013 |
|  |  | ≤ 50 | Ref (1.00) | 0.82 | (0.25,2.73) | 2.79 | (0.72,10.78) | 4.23 | **(1.54,11.65)**** | 0.001 | 0.017 |
|  | Model 3 | > 50 | Ref (1.00) | 1.13 | (0.72,1.77) | 1.39 | (0.93,2.09) | 1.80 | **(1.21,2.67)**** | 0.002 | 0.013 |
|  |  | ≤ 50 | Ref (1.00) | 0.83 | (0.25,2.73) | 2.90 | (0.78,10.85) | 4.16 | **(1.53,11.30)**** | 0.001 | 0.017 |
|  | Model 4 | > 50 | Ref (1.00) | 1.23 | (0.77,1.97) | 1.63 | **(1.07,2.47)*** | 2.24 | **(1.44,3.49)***** | < 0.001 | 0.001 |
|  |  | ≤ 50 | Ref (1.00) | 0.91 | (0.26,3.19) | 3.50 | (0.90,13.63) | 4.91 | **(1.40,17.27)*** | 0.003 | 0.041 |
| Sex |  |  |  |  |  |  |  |  |  |  |  |
|  | Model l | Male | Ref (1.00) | 0.97 | (0.60,1.58) | 1.42 | (0.88,2.30) | 1.74 | **(1.09,2.76)*** | 0.004 | 0.059 |
|  |  | Female | Ref (1.00) | 1.55 | (0.69,3.49) | 1.91 | (0.93,3.92) | 3.52 | **(1.90,6.52)***** | < 0.001 | < 0.001 |
|  | Model 2 | Male | Ref (1.00) | 0.97 | (0.59,1.60) | 1.40 | (0.87,2.26) | 1.72 | **(1.08,2.74)*** | 0.005 | 0.068 |
|  |  | Female | Ref (1.00) | 1.45 | (0.64,3.30) | 1.75 | (0.84,3.63) | 3.15 | **(1.68,5.90)***** | < 0.001 | 0.001 |
|  | Model 3 | Male | Ref (1.00) | 0.97 | (0.59,1.59) | 1.38 | (0.86,2.22) | 1.69 | **(1.06,2.70)*** | 0.007 | 0.088 |
|  |  | Female | Ref (1.00) | 1.44 | (0.64,3.28) | 1.71 | (0.82,3.57) | 3.10 | **(1.66,5.80)***** | < 0.001 | 0.002 |
|  | Model 4 | Male | Ref (1.00) | 1.05 | (0.63,1.74) | 1.61 | (0.99,2.63) | 2.31 | **(1.36,3.91)**** | < 0.001 | 0.007 |
|  |  | Female | Ref (1.00) | 1.59 | (0.70,3.63) | 1.90 | (0.92,3.93) | 3.03 | **(1.56,5.89)***** | < 0.001 | 0.004 |
| Race |  |  |  |  |  |  |  |  |  |  |  |
|  | Model l | Non-hispanic white | Ref (1.00) | 1.06 | (0.64,1.78) | 1.65 | **(1.03,2.64)*** | 2.09 | **(1.34,3.26)***** | < 0.001 | 0.004 |
|  |  | Others | Ref (1.00) | 1.30 | (0.65,2.61) | 0.88 | (0.42,1.84) | 2.26 | **(1.32,3.87)**** | 0.007 | 0.010 |
|  | Model 2 | Non-hispanic white | Ref (1.00) | 1.03 | (0.61,1.75) | 1.57 | (0.98,2.51) | 1.96 | **(1.25,3.06)**** | < 0.001 | 0.011 |
|  |  | Others | Ref (1.00) | 1.28 | (0.64,2.59) | 0.87 | (0.41,1.84) | 2.23 | **(1.29,3.85)**** | 0.008 | 0.014 |
|  | Model 3 | Non-hispanic white | Ref (1.00) | 1.03 | (0.61,1.75) | 1.55 | (0.96,2.50) | 1.93 | **(1.22,3.05)**** | 0.001 | 0.016 |
|  |  | Others | Ref (1.00) | 1.31 | (0.66,2.62) | 0.89 | (0.43,1.87) | 2.25 | **(1.31,3.88)**** | 0.008 | 0.012 |
|  | Model 4 | Non-hispanic white | Ref (1.00) | 1.15 | (0.67,1.98) | 1.83 | **(1.12,2.97)*** | 2.53 | **(1.52,4.19)***** | < 0.001 | 0.001 |
|  |  | Others | Ref (1.00) | 1.32 | (0.65,2.68) | 0.91 | (0.40,2.07) | 2.05 | **(1.11,3.81)*** | 0.028 | 0.070 |
| Exercise |  |  |  |  |  |  |  |  |  |  |  |
|  | Model l | Yes | Ref (1.00) | 0.86 | (0.50,1.47) | 1.42 | (0.83,2.43) | 2.26 | **(1.35,3.79)**** | < 0.001 | 0.007 |
|  |  | No | Ref (1.00) | 1.64 | (0.85,3.16) | 1.85 | (1.00,3.41) | 1.99 | **(1.16,3.42)*** | 0.010 | 0.040 |
|  | Model 2 | Yes | Ref (1.00) | 0.84 | (0.48,1.45) | 1.40 | (0.83,2.39) | 2.27 | **(1.36,3.77)**** | < 0.001 | 0.006 |
|  |  | No | Ref (1.00) | 1.75 | (0.89,3.41) | 1.84 | (0.99,3.42) | 1.98 | **(1.14,3.44)*** | 0.018 | 0.047 |
|  | Model 3 | Yes | Ref (1.00) | 0.84 | (0.48,1.45) | 1.40 | (0.82,2.40) | 2.27 | **(1.37,3.76)**** | < 0.001 | 0.005 |
|  |  | No | Ref (1.00) | 1.81 | (0.93,3.52) | 1.87 | **(1.01,3.48)*** | 2.01 | **(1.15,3.51)*** | 0.022 | 0.047 |
|  | Model 4 | Yes | Ref (1.00) | 0.88 | (0.51,1.53) | 1.55 | (0.89,2.70) | 2.46 | **(1.35,4.47)**** | < 0.001 | 0.011 |
|  |  | No | Ref (1.00) | 2.04 | **(1.05,3.98)*** | 2.26 | **(1.21,4.22)*** | 2.87 | **(1.65,5.00)***** | < 0.001 | 0.001 |
| Smoking |  |  |  |  |  |  |  |  |  |  |  |
|  | Model l | Yes | Ref (1.00) | 0.76 | (0.30,1.93) | 0.83 | (0.37,1.86) | 0.68 | (0.28,1.65) | 0.485 | 1.000 |
|  |  | No | Ref (1.00) | 1.21 | (0.73,2.01) | 1.75 | **(1.05,2.94)*** | 2.77 | **(1.75,4.37)***** | < 0.001 | < 0.001 |
|  | Model 2 | Yes | Ref (1.00) | 0.72 | (0.27,1.92) | 0.81 | (0.35,1.84) | 0.68 | (0.27,1.68) | 0.522 | 1.000 |
|  |  | No | Ref (1.00) | 1.20 | (0.71,2.01) | 1.71 | **(1.01,2.90)*** | 2.68 | **(1.68,4.27)***** | < 0.001 | < 0.001 |
|  | Model 3 | Yes | Ref (1.00) | 0.73 | (0.28,1.92) | 0.81 | (0.35,1.85) | 0.70 | (0.28,1.74) | 0.572 | 1.000 |
|  |  | No | Ref (1.00) | 1.20 | (0.72,2.02) | 1.70 | **(1.01,2.89)*** | 2.67 | **(1.67,4.27)***** | < 0.001 | < 0.001 |
|  | Model 4 | Yes | Ref (1.00) | 0.93 | (0.33,2.61) | 1.04 | (0.43,2.52) | 1.20 | (0.45,3.22) | 0.668 | 1.000 |
|  |  | No | Ref (1.00) | 1.28 | (0.77,2.13) | 1.89 | **(1.13,3.18)*** | 3.09 | **(1.89,5.04)***** | < 0.001 | < 0.001 |
| Drinking |  |  |  |  |  |  |  |  |  |  |  |
|  | Model l | Yes | Ref (1.00) | 1.24 | (0.77,2.01) | 1.61 | (0.99,2.60) | 2.03 | **(1.28,3.23)**** | 0.002 | 0.009 |
|  |  | No | Ref (1.00) | 0.61 | (0.25,1.46) | 1.27 | (0.60,2.72) | 2.40 | **(1.10,5.23)*** | 0.001 | 0.084 |
|  | Model 2 | Yes | Ref (1.00) | 1.20 | (0.73,1.96) | 1.52 | (0.93,2.47) | 1.91 | **(1.20,3.03)**** | 0.004 | 0.019 |
|  |  | No | Ref (1.00) | 0.64 | (0.27,1.52) | 1.26 | (0.59,2.70) | 2.43 | **(1.14,5.20)*** | < 0.001 | 0.067 |
|  | Model 3 | Yes | Ref (1.00) | 1.21 | (0.74,1.99) | 1.52 | (0.94,2.47) | 1.91 | **(1.20,3.06)**** | 0.004 | 0.021 |
|  |  | No | Ref (1.00) | 0.63 | (0.26,1.50) | 1.25 | (0.58,2.68) | 2.43 | **(1.14,5.15)*** | < 0.001 | 0.064 |
|  | Model 4 | Yes | Ref (1.00) | 1.36 | (0.82,2.28) | 1.81 | **(1.13,2.90)*** | 2.62 | **(1.62,4.26)***** | < 0.001 | < 0.001 |
|  |  | No | Ref (1.00) | 0.64 | (0.27,1.55) | 1.28 | (0.58,2.80) | 2.31 | **(1.07,5.00)*** | 0.001 | 0.099 |
| T2DM |  |  |  |  |  |  |  |  |  |  |  |
|  | Model l | Yes | Ref (1.00) | 0.61 | (0.21,1.77) | 0.97 | (0.32,2.89) | 1.13 | (0.41,3.08) | 0.266 | 1.000 |
|  |  | No | Ref (1.00) | 1.13 | (0.73,1.75) | 1.44 | (0.94,2.22) | 1.65 | **(1.13,2.42)*** | 0.004 | 0.033 |
|  | Model 2 | Yes | Ref (1.00) | 0.61 | (0.21,1.82) | 0.94 | (0.32,2.78) | 1.10 | (0.42,2.93) | 0.274 | 1.000 |
|  |  | No | Ref (1.00) | 1.13 | (0.73,1.77) | 1.42 | (0.92,2.20) | 1.63 | **(1.11,2.40)*** | 0.005 | 0.040 |
|  | Model 3 | Yes | Ref (1.00) | 0.61 | (0.21,1.82) | 0.94 | (0.32,2.78) | 1.10 | (0.42,2.93) | 0.256 | 1.000 |
|  |  | No | Ref (1.00) | 1.13 | (0.72,1.77) | 1.41 | (0.91,2.19) | 1.61 | **(1.09,2.39)*** | 0.007 | 0.051 |
|  | Model 4 | Yes | Ref (1.00) | 0.58 | (0.20,1.68) | 1.02 | (0.35,2.95) | 1.27 | (0.50,3.24) | 0.103 | 0.942 |
|  |  | No | Ref (1.00) | 1.31 | (0.83,2.09) | 1.77 | **(1.15,2.72)**** | 2.53 | **(1.67,3.83)***** | < 0.001 | < 0.001 |

^a^The variables adjusted in each model were the factors mentioned above except the stratification variables.

^b^Data were listed as the weighted odd ratio estimates and 95% confidence intervals, with *p < 0.05, **p < 0.01, ***p < 0.001.

^c^Q, quintile.

^d^Ref, reference.

^e^Tests for trends based on the variables containing the median values for each quartile.

^f^*P*_test_ was the result of Bonfreni correction.

Supplementary Table 29. Association of TyG-WC with coronary heart disease stratified by age, sex, race, exercise, smoking, drinking, and T2DM status

|  |  |  | TyG-WC | | | | | | | *P*_trend_^e^ | *P*_test_^f^ |
| --- | --- | --- | --- | --- | --- | --- | --- | --- | --- | --- | --- |
|  |  |  | Q1^c^ | Q2 | | Q3 | | Q4 | |  |  |
|  |  |  |  | HR/OR^b^ | 95% CI | HR/OR | 95% CI | HR/OR | 95% CI |  |  |
| Age |  |  |  |  |  |  |  |  |  |  |  |
|  | Model 1^a^ | > 50 | Ref (1.00)^d^ | 1.06 | (0.63,1.78) | 1.28 | (0.80,2.05) | 1.88 | **(1.16,3.06)*** | 0.001 | 0.034 |
|  |  | ≤ 50 | Ref (1.00) | 0.62 | (0.16,2.40) | 1.55 | (0.30,8.03) | 5.60 | **(2.24,13.98)***** | < 0.001 | 0.001 |
|  | Model 2 | > 50 | Ref (1.00) | 1.02 | (0.60,1.72) | 1.21 | (0.75,1.94) | 1.75 | **(1.08,2.83)*** | 0.001 | 0.074 |
|  |  | ≤ 50 | Ref (1.00) | 0.56 | (0.15,2.12) | 1.28 | (0.23,7.12) | 4.39 | **(1.89,10.23)**** | 0.001 | 0.002 |
|  | Model 3 | > 50 | Ref (1.00) | 1.03 | (0.61,1.74) | 1.22 | (0.76,1.97) | 1.81 | **(1.12,2.92)*** | 0.001 | 0.047 |
|  |  | ≤ 50 | Ref (1.00) | 0.55 | (0.15,2.01) | 1.31 | (0.25,6.78) | 4.53 | **(1.93,10.62)**** | < 0.001 | 0.002 |
|  | Model 4 | > 50 | Ref (1.00) | 1.03 | (0.62,1.73) | 1.20 | (0.75,1.91) | 1.65 | **(1.03,2.64)*** | 0.006 | 0.112 |
|  |  | ≤ 50 | Ref (1.00) | 0.58 | (0.15,2.21) | 1.41 | (0.27,7.41) | 4.52 | **(1.61,12.68)**** | 0.001 | 0.014 |
| Sex |  |  |  |  |  |  |  |  |  |  |  |
|  | Model l | Male | Ref (1.00) | 1.11 | (0.55,2.26) | 1.39 | (0.73,2.65) | 2.41 | **(1.32,4.42)**** | < 0.001 | 0.014 |
|  |  | Female | Ref (1.00) | 0.94 | (0.49,1.80) | 1.18 | (0.66,2.12) | 2.23 | **(1.13,4.41)*** | 0.011 | 0.064 |
|  | Model 2 | Male | Ref (1.00) | 1.09 | (0.53,2.22) | 1.35 | (0.70,2.60) | 2.36 | **(1.27,4.39)**** | < 0.001 | 0.021 |
|  |  | Female | Ref (1.00) | 0.91 | (0.48,1.74) | 1.10 | (0.61,1.98) | 1.93 | (0.98,3.79) | 0.032 | 0.168 |
|  | Model 3 | Male | Ref (1.00) | 1.08 | (0.53,2.19) | 1.33 | (0.70,2.54) | 2.32 | **(1.27,4.25)**** | < 0.001 | 0.020 |
|  |  | Female | Ref (1.00) | 0.88 | (0.46,1.69) | 1.06 | (0.59,1.90) | 1.87 | (0.98,3.58) | 0.032 | 0.176 |
|  | Model 4 | Male | Ref (1.00) | 1.18 | (0.59,2.35) | 1.47 | (0.78,2.78) | 2.49 | **(1.36,4.56)**** | < 0.001 | 0.010 |
|  |  | Female | Ref (1.00) | 0.88 | (0.45,1.70) | 0.93 | (0.52,1.67) | 1.44 | (0.76,2.75) | 0.192 | 0.774 |
| Race |  |  |  |  |  |  |  |  |  |  |  |
|  | Model l | Non-hispanic white | Ref (1.00) | 1.05 | (0.59,1.84) | 1.26 | (0.76,2.10) | 2.20 | **(1.32,3.64)**** | < 0.001 | 0.008 |
|  |  | Others | Ref (1.00) | 0.90 | (0.42,1.91) | 1.20 | (0.71,2.02) | 2.26 | **(1.37,3.75)**** | < 0.001 | 0.005 |
|  | Model 2 | Non-hispanic white | Ref (1.00) | 1.03 | (0.58,1.82) | 1.19 | (0.71,2.00) | 2.06 | **(1.25,3.39)**** | < 0.001 | 0.016 |
|  |  | Others | Ref (1.00) | 0.89 | (0.42,1.90) | 1.17 | (0.69,2.00) | 2.17 | **(1.29,3.63)**** | 0.001 | 0.011 |
|  | Model 3 | Non-hispanic white | Ref (1.00) | 1.03 | (0.58,1.81) | 1.18 | (0.71,1.97) | 2.03 | **(1.24,3.32)**** | < 0.001 | 0.016 |
|  |  | Others | Ref (1.00) | 0.90 | (0.42,1.91) | 1.19 | (0.70,2.00) | 2.22 | **(1.32,3.73)**** | 0.001 | 0.009 |
|  | Model 4 | Non-hispanic white | Ref (1.00) | 1.12 | (0.62,2.00) | 1.26 | (0.76,2.10) | 2.07 | **(1.29,3.32)**** | < 0.001 | 0.009 |
|  |  | Others | Ref (1.00) | 0.87 | (0.43,1.78) | 1.04 | (0.62,1.74) | 1.85 | **(1.07,3.21)*** | 0.016 | 0.084 |
| Exercise |  |  |  |  |  |  |  |  |  |  |  |
|  | Model l | Yes | Ref (1.00) | 1.62 | (0.83,3.18) | 1.45 | (0.82,2.57) | 3.33 | **(1.78,6.25)***** | < 0.001 | 0.001 |
|  |  | No | Ref (1.00) | 0.54 | (0.27,1.09) | 1.09 | (0.61,1.95) | 1.29 | (0.70,2.39) | 0.043 | 0.259 |
|  | Model 2 | Yes | Ref (1.00) | 1.62 | (0.83,3.14) | 1.45 | (0.82,2.58) | 3.36 | **(1.81,6.25)***** | < 0.001 | 0.001 |
|  |  | No | Ref (1.00) | 0.53 | (0.26,1.07) | 1.00 | (0.56,1.80) | 1.21 | (0.65,2.27) | 0.077 | 0.229 |
|  | Model 3 | Yes | Ref (1.00) | 1.63 | (0.84,3.16) | 1.46 | (0.83,2.59) | 3.37 | **(1.82,6.25)***** | < 0.001 | 0.001 |
|  |  | No | Ref (1.00) | 0.53 | (0.26,1.09) | 1.02 | (0.56,1.86) | 1.23 | (0.64,2.36) | 0.083 | 0.249 |
|  | Model 4 | Yes | Ref (1.00) | 1.73 | (0.90,3.34) | 1.43 | (0.81,2.53) | 3.02 | **(1.57,5.81)**** | < 0.001 | 0.003 |
|  |  | No | Ref (1.00) | 0.55 | (0.26,1.15) | 1.10 | (0.61,1.98) | 1.32 | (0.70,2.49) | 0.042 | 0.336 |
| Smoking |  |  |  |  |  |  |  |  |  |  |  |
|  | Model l | Yes | Ref (1.00) | 0.58 | (0.23,1.49) | 0.39 | (0.13,1.17) | 1.04 | (0.44,2.44) | 0.562 | 0.274 |
|  |  | No | Ref (1.00) | 1.36 | (0.78,2.37) | 1.96 | **(1.10,3.47)*** | 3.16 | **(1.93,5.20)***** | < 0.001 | < 0.001 |
|  | Model 2 | Yes | Ref (1.00) | 0.53 | (0.21,1.33) | 0.36 | (0.11,1.15) | 1.00 | (0.44,2.31) | 0.542 | 0.256 |
|  |  | No | Ref (1.00) | 1.33 | (0.76,2.34) | 1.88 | **(1.04,3.39)*** | 3.05 | **(1.84,5.04)***** | < 0.001 | < 0.001 |
|  | Model 3 | Yes | Ref (1.00) | 0.54 | (0.22,1.37) | 0.38 | (0.12,1.21) | 1.02 | (0.44,2.35) | 0.522 | 0.298 |
|  |  | No | Ref (1.00) | 1.34 | (0.76,2.34) | 1.89 | **(1.05,3.38)*** | 3.07 | **(1.89,5.01)***** | < 0.001 | < 0.001 |
|  | Model 4 | Yes | Ref (1.00) | 0.62 | (0.26,1.52) | 0.44 | (0.14,1.38) | 1.24 | (0.53,2.90) | 0.359 | 0.474 |
|  |  | No | Ref (1.00) | 1.36 | (0.76,2.42) | 1.84 | **(1.01,3.37)*** | 2.80 | **(1.66,4.73)***** | < 0.001 | 0.001 |
| Drinking |  |  |  |  |  |  |  |  |  |  |  |
|  | Model l | Yes | Ref (1.00) | 0.77 | (0.44,1.35) | 1.09 | (0.67,1.77) | 1.88 | **(1.16,3.07)*** | < 0.001 | 0.034 |
|  |  | No | Ref (1.00) | 2.67 | **(1.06,6.68)*** | 2.66 | **(1.14,6.20)*** | 4.96 | **(2.20,11.18)***** | < 0.001 | 0.001 |
|  | Model 2 | Yes | Ref (1.00) | 0.75 | (0.42,1.33) | 1.04 | (0.63,1.70) | 1.76 | **(1.09,2.85)*** | < 0.001 | 0.067 |
|  |  | No | Ref (1.00) | 2.68 | **(1.04,6.86)*** | 2.72 | **(1.14,6.49)*** | 5.03 | **(2.20,11.49)***** | < 0.001 | 0.001 |
|  | Model 3 | Yes | Ref (1.00) | 0.75 | (0.43,1.33) | 1.04 | (0.64,1.69) | 1.79 | **(1.11,2.88)*** | < 0.001 | 0.053 |
|  |  | No | Ref (1.00) | 2.66 | **(1.03,6.88)*** | 2.72 | **(1.13,6.54)*** | 4.94 | **(2.16,11.28)***** | < 0.001 | 0.001 |
|  | Model 4 | Yes | Ref (1.00) | 0.83 | (0.47,1.47) | 1.14 | (0.70,1.85) | 1.88 | **(1.20,2.97)**** | < 0.001 | 0.020 |
|  |  | No | Ref (1.00) | 2.51 | (0.97,6.48) | 2.46 | **(1.03,5.88)*** | 4.16 | **(1.86,9.35)**** | 0.001 | 0.002 |
| T2DM |  |  |  |  |  |  |  |  |  |  |  |
|  | Model l | Yes | Ref (1.00) | 0.55 | (0.11,2.70) | 0.69 | (0.18,2.69) | 1.40 | (0.31,6.28) | 0.025 | 1.000 |
|  |  | No | Ref (1.00) | 1.03 | (0.63,1.67) | 1.25 | (0.79,1.96) | 1.65 | **(1.07,2.54)*** | 0.006 | 0.070 |
|  | Model 2 | Yes | Ref (1.00) | 0.54 | (0.11,2.62) | 0.70 | (0.19,2.63) | 1.35 | (0.31,5.82) | 0.034 | 1.000 |
|  |  | No | Ref (1.00) | 1.02 | (0.62,1.66) | 1.21 | (0.77,1.91) | 1.62 | **(1.05,2.49)*** | 0.007 | 0.089 |
|  | Model 3 | Yes | Ref (1.00) | 0.56 | (0.12,2.73) | 0.72 | (0.19,2.74) | 1.40 | (0.32,6.07) | 0.030 | 1.000 |
|  |  | No | Ref (1.00) | 1.01 | (0.62,1.65) | 1.20 | (0.76,1.90) | 1.60 | **(1.04,2.46)*** | 0.008 | 0.098 |
|  | Model 4 | Yes | Ref (1.00) | 0.59 | (0.13,2.73) | 0.75 | (0.21,2.69) | 1.47 | (0.36,5.93) | 0.023 | 1.000 |
|  |  | No | Ref (1.00) | 1.09 | (0.66,1.80) | 1.32 | (0.83,2.08) | 1.78 | **(1.15,2.74)*** | 0.002 | 0.030 |

^a^The variables adjusted in each model were the factors mentioned above except the stratification variables.

^b^Data were listed as the weighted odd ratio estimates and 95% confidence intervals, with *p < 0.05, **p < 0.01, ***p < 0.001.

^c^Q, quintile.

^d^Ref, reference.

^e^Tests for trends based on the variables containing the median values for each quartile.

^f^*P*_test_ was the result of Bonfreni correction.

Supplementary Table 30. Association of TyG-WtHR with coronary heart disease stratified by age, sex, race, exercise, smoking, drinking, and T2DM status

|  |  |  | TyG-WtHR | | | | | | | *P*_trend_^e^ | *P*_test_^f^ |
| --- | --- | --- | --- | --- | --- | --- | --- | --- | --- | --- | --- |
|  |  |  | Q1^c^ | Q2 | | Q3 | | Q4 | |  |  |
|  |  |  |  | HR/OR^b^ | 95% CI | HR/OR | 95% CI | HR/OR | 95% CI |  |  |
| Age |  |  |  |  |  |  |  |  |  |  |  |
|  | Model 1^a^ | > 50 | Ref (1.00)^d^ | 1.01 | (0.65,1.59) | 1.69 | **(1.15,2.50)**** | 2.02 | **(1.35,3.02)**** | < 0.001 | 0.003 |
|  |  | ≤ 50 | Ref (1.00) | 1.52 | (0.33,6.97) | 2.12 | (0.49,9.12) | 8.28 | **(2.99,22.90)***** | < 0.001 | < 0.001 |
|  | Model 2 | > 50 | Ref (1.00) | 0.98 | (0.62,1.53) | 1.61 | **(1.08,2.38)*** | 1.87 | **(1.25,2.80)**** | < 0.001 | 0.008 |
|  |  | ≤ 50 | Ref (1.00) | 1.38 | (0.31,6.20) | 1.75 | (0.40,7.70) | 6.24 | **(2.37,16.44)***** | < 0.001 | 0.001 |
|  | Model 3 | > 50 | Ref (1.00) | 0.95 | (0.61,1.50) | 1.56 | **(1.05,2.32)*** | 1.84 | **(1.23,2.75)**** | < 0.001 | 0.010 |
|  |  | ≤ 50 | Ref (1.00) | 1.36 | (0.31,6.01) | 1.85 | (0.44,7.75) | 6.26 | **(2.37,16.58)***** | < 0.001 | 0.001 |
|  | Model 4 | > 50 | Ref (1.00) | 0.98 | (0.63,1.53) | 1.59 | **(1.09,2.32)*** | 1.72 | **(1.14,2.58)*** | 0.002 | 0.029 |
|  |  | ≤ 50 | Ref (1.00) | 1.48 | (0.31,6.96) | 1.98 | (0.44,8.83) | 6.28 | **(2.08,19.02)**** | < 0.001 | 0.004 |
| Sex |  |  |  |  |  |  |  |  |  |  |  |
|  | Model l | Male | Ref (1.00) | 0.98 | (0.57,1.70) | 1.79 | **(1.14,2.81)*** | 2.28 | **(1.40,3.72)**** | < 0.001 | 0.004 |
|  |  | Female | Ref (1.00) | 1.12 | (0.51,2.45) | 1.14 | (0.54,2.43) | 2.30 | **(1.05,5.07)*** | 0.009 | 0.116 |
|  | Model 2 | Male | Ref (1.00) | 0.96 | (0.56,1.67) | 1.74 | **(1.09,2.76)*** | 2.25 | **(1.37,3.71)**** | < 0.001 | 0.005 |
|  |  | Female | Ref (1.00) | 1.07 | (0.48,2.36) | 1.05 | (0.49,2.24) | 1.99 | (0.91,4.36) | 0.023 | 0.256 |
|  | Model 3 | Male | Ref (1.00) | 0.95 | (0.55,1.64) | 1.70 | **(1.09,2.66)*** | 2.19 | **(1.33,3.61)**** | < 0.001 | 0.007 |
|  |  | Female | Ref (1.00) | 1.04 | (0.47,2.29) | 1.00 | (0.47,2.11) | 1.91 | (0.90,4.07) | 0.023 | 0.275 |
|  | Model 4 | Male | Ref (1.00) | 1.04 | (0.60,1.78) | 1.83 | **(1.15,2.89)*** | 2.35 | **(1.38,3.99)**** | < 0.001 | 0.006 |
|  |  | Female | Ref (1.00) | 1.05 | (0.48,2.31) | 0.93 | (0.44,1.94) | 1.52 | (0.73,3.18) | 0.120 | 0.782 |
| Race |  |  |  |  |  |  |  |  |  |  |  |
|  | Model l | Non-hispanic white | Ref (1.00) | 1.01 | (0.63,1.62) | 1.58 | **(1.08,2.31)*** | 2.18 | **(1.42,3.36)**** | < 0.001 | 0.002 |
|  |  | Others | Ref (1.00) | 0.95 | (0.43,2.09) | 1.31 | (0.64,2.67) | 2.50 | **(1.42,4.39)**** | < 0.001 | 0.005 |
|  | Model 2 | Non-hispanic white | Ref (1.00) | 0.98 | (0.61,1.56) | 1.48 | **(1.01,2.17)*** | 2.02 | **(1.32,3.08)**** | < 0.001 | 0.004 |
|  |  | Others | Ref (1.00) | 0.92 | (0.42,1.99) | 1.25 | (0.63,2.48) | 2.37 | **(1.36,4.12)**** | < 0.001 | 0.008 |
|  | Model 3 | Non-hispanic white | Ref (1.00) | 0.97 | (0.60,1.56) | 1.46 | (0.99,2.14) | 1.98 | **(1.29,3.04)**** | < 0.001 | 0.007 |
|  |  | Others | Ref (1.00) | 0.90 | (0.41,1.98) | 1.26 | (0.64,2.49) | 2.36 | **(1.36,4.11)**** | < 0.001 | 0.008 |
|  | Model 4 | Non-hispanic white | Ref (1.00) | 1.06 | (0.66,1.70) | 1.56 | **(1.07,2.27)*** | 2.03 | **(1.33,3.09)**** | < 0.001 | 0.004 |
|  |  | Others | Ref (1.00) | 0.89 | (0.40,2.01) | 1.18 | (0.61,2.31) | 1.97 | **(1.01,3.84)*** | 0.005 | 0.141 |
| Exercise |  |  |  |  |  |  |  |  |  |  |  |
|  | Model l | Yes | Ref (1.00) | 1.25 | (0.73,2.16) | 1.61 | (0.96,2.68) | 2.97 | **(1.71,5.15)***** | < 0.001 | < 0.001 |
|  |  | No | Ref (1.00) | 0.88 | (0.47,1.62) | 1.70 | **(1.06,2.74)*** | 1.51 | (0.82,2.81) | 0.029 | 0.089 |
|  | Model 2 | Yes | Ref (1.00) | 1.21 | (0.70,2.11) | 1.59 | (0.95,2.66) | 2.97 | **(1.71,5.14)***** | < 0.001 | < 0.001 |
|  |  | No | Ref (1.00) | 0.83 | (0.44,1.56) | 1.52 | (0.94,2.46) | 1.41 | (0.74,2.66) | 0.067 | 0.267 |
|  | Model 3 | Yes | Ref (1.00) | 1.22 | (0.70,2.11) | 1.59 | (0.96,2.63) | 2.95 | **(1.71,5.08)***** | < 0.001 | < 0.001 |
|  |  | No | Ref (1.00) | 0.83 | (0.44,1.59) | 1.53 | (0.93,2.53) | 1.39 | (0.70,2.73) | 0.091 | 0.281 |
|  | Model 4 | Yes | Ref (1.00) | 1.24 | (0.71,2.16) | 1.57 | (0.95,2.58) | 2.64 | **(1.49,4.69)**** | < 0.001 | 0.003 |
|  |  | No | Ref (1.00) | 0.91 | (0.48,1.74) | 1.66 | **(1.01,2.72)*** | 1.52 | (0.79,2.93) | 0.047 | 0.131 |
| Smoking |  |  |  |  |  |  |  |  |  |  |  |
|  | Model l | Yes | Ref (1.00) | 0.60 | (0.25,1.45) | 0.67 | (0.28,1.64) | 0.88 | (0.35,2.16) | 0.961 | 0.758 |
|  |  | No | Ref (1.00) | 1.37 | (0.86,2.19) | 2.33 | **(1.51,3.60)***** | 3.40 | **(2.26,5.11)***** | < 0.001 | < 0.001 |
|  | Model 2 | Yes | Ref (1.00) | 0.57 | (0.23,1.42) | 0.69 | (0.28,1.67) | 0.84 | (0.34,2.11) | 0.933 | 0.669 |
|  |  | No | Ref (1.00) | 1.34 | (0.84,2.16) | 2.25 | **(1.44,3.52)**** | 3.29 | **(2.16,5.01)***** | < 0.001 | < 0.001 |
|  | Model 3 | Yes | Ref (1.00) | 0.57 | (0.23,1.42) | 0.68 | (0.28,1.67) | 0.85 | (0.34,2.12) | 0.950 | 0.665 |
|  |  | No | Ref (1.00) | 1.34 | (0.84,2.15) | 2.24 | **(1.45,3.48)**** | 3.28 | **(2.17,4.96)***** | < 0.001 | < 0.001 |
|  | Model 4 | Yes | Ref (1.00) | 0.68 | (0.29,1.62) | 0.83 | (0.35,1.97) | 1.02 | (0.41,2.58) | 0.825 | 1.000 |
|  |  | No | Ref (1.00) | 1.37 | (0.85,2.20) | 2.25 | **(1.42,3.54)***** | 3.05 | **(1.97,4.74)***** | < 0.001 | < 0.001 |
| Drinking |  |  |  |  |  |  |  |  |  |  |  |
|  | Model l | Yes | Ref (1.00) | 0.96 | (0.60,1.55) | 1.52 | **(1.06,2.16)*** | 2.18 | **(1.42,3.34)***** | < 0.001 | 0.001 |
|  |  | No | Ref (1.00) | 1.25 | (0.43,3.65) | 2.08 | (0.78,5.53) | 3.02 | **(1.15,7.94)*** | 0.002 | 0.077 |
|  | Model 2 | Yes | Ref (1.00) | 0.93 | (0.57,1.51) | 1.45 | **(1.01,2.07)*** | 2.01 | **(1.32,3.07)**** | < 0.001 | 0.004 |
|  |  | No | Ref (1.00) | 1.40 | (0.49,4.00) | 2.31 | (0.88,6.05) | 3.31 | **(1.31,8.39)*** | 0.001 | 0.036 |
|  | Model 3 | Yes | Ref (1.00) | 0.93 | (0.57,1.51) | 1.45 | **(1.02,2.06)*** | 2.02 | **(1.32,3.09)**** | < 0.001 | 0.004 |
|  |  | No | Ref (1.00) | 1.40 | (0.48,4.09) | 2.26 | (0.83,6.13) | 3.28 | **(1.27,8.46)*** | 0.001 | 0.045 |
|  | Model 4 | Yes | Ref (1.00) | 1.02 | (0.64,1.64) | 1.57 | **(1.13,2.20)**** | 2.12 | **(1.42,3.17)***** | < 0.001 | 0.001 |
|  |  | No | Ref (1.00) | 1.33 | (0.47,3.80) | 2.07 | (0.79,5.46) | 2.73 | **(1.07,6.95)*** | 0.006 | 0.107 |
| T2DM |  |  |  |  |  |  |  |  |  |  |  |
|  | Model l | Yes | Ref (1.00) | 0.32 | (0.08,1.31) | 0.85 | (0.18,3.99) | 1.19 | (0.27,5.20) | 0.016 | 0.339 |
|  |  | No | Ref (1.00) | 1.08 | (0.68,1.72) | 1.46 | **(1.03,2.06)*** | 1.62 | **(1.10,2.39)*** | 0.003 | 0.045 |
|  | Model 2 | Yes | Ref (1.00) | 0.31 | (0.07,1.35) | 0.85 | (0.19,3.70) | 1.15 | (0.28,4.69) | 0.023 | 0.354 |
|  |  | No | Ref (1.00) | 1.05 | (0.66,1.68) | 1.41 | (1.00,2.00) | 1.59 | **(1.09,2.34)*** | 0.005 | 0.052 |
|  | Model 3 | Yes | Ref (1.00) | 0.31 | (0.07,1.36) | 0.85 | (0.19,3.78) | 1.16 | (0.28,4.80) | 0.022 | 0.357 |
|  |  | No | Ref (1.00) | 1.04 | (0.65,1.67) | 1.39 | (0.98,1.98) | 1.57 | **(1.06,2.32)*** | 0.008 | 0.078 |
|  | Model 4 | Yes | Ref (1.00) | 0.31 | (0.07,1.28) | 0.87 | (0.21,3.60) | 1.19 | (0.31,4.59) | 0.017 | 0.310 |
|  |  | No | Ref (1.00) | 1.14 | (0.71,1.84) | 1.56 | **(1.09,2.22)*** | 1.77 | **(1.18,2.64)**** | 0.002 | 0.018 |

^a^The variables adjusted in each model were the factors mentioned above except the stratification variables.

^b^Data were listed as the weighted odd ratio estimates and 95% confidence intervals, with *p < 0.05, **p < 0.01, ***p < 0.001.

^c^Q, quintile.

^d^Ref, reference.

^e^Tests for trends based on the variables containing the median values for each quartile.

^f^*P*_test_ was the result of Bonfreni correction.

Supplementary Table 31. Association of TyG-BMI with coronary heart disease stratified by age, sex, race, exercise, smoking, drinking, and T2DM status

|  |  |  | TyG-BMI | | | | | | | *P*_trend_^e^ | *P*_test_^f^ |
| --- | --- | --- | --- | --- | --- | --- | --- | --- | --- | --- | --- |
|  |  |  | Q1^c^ | Q2 | | Q3 | | Q4 | |  |  |
|  |  |  |  | HR/OR^b^ | 95% CI | HR/OR | 95% CI | HR/OR | 95% CI |  |  |
| Age |  |  |  |  |  |  |  |  |  |  |  |
|  | Model 1^a^ | > 50 | Ref (1.00)^d^ | 0.89 | (0.61,1.30) | 1.21 | (0.81,1.82) | 1.37 | (0.97,1.93) | 0.019 | 0.212 |
|  |  | ≤ 50 | Ref (1.00) | 1.32 | (0.29,6.00) | 1.53 | (0.36,6.51) | 4.89 | **(1.81,13.20)**** | 0.001 | 0.006 |
|  | Model 2 | > 50 | Ref (1.00) | 0.86 | (0.58,1.27) | 1.16 | (0.77,1.76) | 1.29 | (0.91,1.81) | 0.038 | 0.439 |
|  |  | ≤ 50 | Ref (1.00) | 1.27 | (0.28,5.79) | 1.32 | (0.30,5.75) | 3.97 | **(1.52,10.33)**** | 0.002 | 0.016 |
|  | Model 3 | > 50 | Ref (1.00) | 0.85 | (0.57,1.26) | 1.17 | (0.77,1.77) | 1.33 | (0.93,1.88) | 0.023 | 0.340 |
|  |  | ≤ 50 | Ref (1.00) | 1.26 | (0.28,5.68) | 1.34 | (0.32,5.62) | 4.06 | **(1.55,10.66)**** | 0.002 | 0.014 |
|  | Model 4 | > 50 | Ref (1.00) | 0.90 | (0.61,1.35) | 1.18 | (0.78,1.79) | 1.24 | (0.85,1.80) | 0.134 | 0.812 |
|  |  | ≤ 50 | Ref (1.00) | 1.28 | (0.27,6.01) | 1.37 | (0.31,5.97) | 3.69 | **(1.26,10.84)*** | 0.004 | 0.054 |
| Sex |  |  |  |  |  |  |  |  |  |  |  |
|  | Model l | Male | Ref (1.00) | 1.18 | (0.73,1.88) | 1.55 | (0.94,2.57) | 2.53 | **(1.61,3.98)***** | < 0.001 | < 0.001 |
|  |  | Female | Ref (1.00) | 0.77 | (0.41,1.47) | 1.18 | (0.63,2.23) | 1.59 | (0.90,2.80) | 0.047 | 0.324 |
|  | Model 2 | Male | Ref (1.00) | 1.14 | (0.70,1.84) | 1.50 | (0.90,2.51) | 2.47 | **(1.56,3.92)***** | < 0.001 | 0.001 |
|  |  | Female | Ref (1.00) | 0.73 | (0.39,1.38) | 1.08 | (0.57,2.06) | 1.38 | (0.77,2.46) | 0.135 | 0.826 |
|  | Model 3 | Male | Ref (1.00) | 1.11 | (0.69,1.79) | 1.49 | (0.90,2.48) | 2.42 | **(1.53,3.82)***** | < 0.001 | 0.001 |
|  |  | Female | Ref (1.00) | 0.72 | (0.38,1.35) | 1.05 | (0.57,1.96) | 1.34 | (0.77,2.34) | 0.144 | 0.885 |
|  | Model 4 | Male | Ref (1.00) | 1.20 | (0.74,1.96) | 1.52 | (0.91,2.54) | 2.48 | **(1.52,4.03)***** | < 0.001 | 0.001 |
|  |  | Female | Ref (1.00) | 0.67 | (0.35,1.29) | 0.91 | (0.49,1.69) | 1.01 | (0.57,1.77) | 0.647 | 0.695 |
| Race |  |  |  |  |  |  |  |  |  |  |  |
|  | Model l | Non-hispanic white | Ref (1.00) | 0.91 | (0.59,1.41) | 1.36 | (0.89,2.09) | 2.03 | **(1.39,2.98)***** | < 0.001 | 0.001 |
|  |  | Others | Ref (1.00) | 1.58 | (0.79,3.19) | 1.28 | (0.75,2.17) | 2.38 | **(1.38,4.10)**** | 0.005 | 0.007 |
|  | Model 2 | Non-hispanic white | Ref (1.00) | 0.87 | (0.55,1.35) | 1.29 | (0.84,1.97) | 1.88 | **(1.28,2.76)**** | < 0.001 | 0.004 |
|  |  | Others | Ref (1.00) | 1.52 | (0.76,3.04) | 1.22 | (0.72,2.07) | 2.25 | **(1.34,3.79)**** | 0.007 | 0.008 |
|  | Model 3 | Non-hispanic white | Ref (1.00) | 0.86 | (0.55,1.35) | 1.27 | (0.83,1.95) | 1.86 | **(1.27,2.72)**** | < 0.001 | 0.005 |
|  |  | Others | Ref (1.00) | 1.50 | (0.75,3.00) | 1.23 | (0.74,2.07) | 2.23 | **(1.31,3.80)**** | 0.008 | 0.010 |
|  | Model 4 | Non-hispanic white | Ref (1.00) | 0.92 | (0.57,1.47) | 1.28 | (0.83,1.97) | 1.79 | **(1.21,2.63)**** | 0.001 | 0.011 |
|  |  | Others | Ref (1.00) | 1.44 | (0.73,2.84) | 1.06 | (0.62,1.81) | 1.85 | **(1.01,3.39)*** | 0.010 | 0.140 |
| Exercise |  |  |  |  |  |  |  |  |  |  |  |
|  | Model l | Yes | Ref (1.00) | 1.10 | (0.65,1.88) | 1.21 | (0.71,2.04) | 2.31 | **(1.45,3.69)**** | < 0.001 | 0.002 |
|  |  | No | Ref (1.00) | 0.92 | (0.48,1.78) | 1.67 | (0.97,2.88) | 1.61 | (0.85,3.08) | 0.025 | 0.187 |
|  | Model 2 | Yes | Ref (1.00) | 1.08 | (0.62,1.85) | 1.20 | (0.71,2.03) | 2.30 | **(1.44,3.68)**** | < 0.001 | 0.002 |
|  |  | No | Ref (1.00) | 0.84 | (0.43,1.66) | 1.51 | (0.88,2.59) | 1.47 | (0.76,2.84) | 0.053 | 0.405 |
|  | Model 3 | Yes | Ref (1.00) | 1.08 | (0.64,1.84) | 1.22 | (0.72,2.05) | 2.32 | **(1.46,3.68)***** | < 0.001 | 0.001 |
|  |  | No | Ref (1.00) | 0.84 | (0.43,1.67) | 1.51 | (0.87,2.63) | 1.47 | (0.75,2.89) | 0.061 | 0.417 |
|  | Model 4 | Yes | Ref (1.00) | 1.08 | (0.62,1.86) | 1.17 | (0.69,1.98) | 1.95 | **(1.17,3.26)*** | 0.050 | 0.032 |
|  |  | No | Ref (1.00) | 0.90 | (0.45,1.80) | 1.56 | (0.91,2.68) | 1.49 | (0.77,2.89) | 0.056 | 0.324 |
| Smoking |  |  |  |  |  |  |  |  |  |  |  |
|  | Model l | Yes | Ref (1.00) | 0.47 | (0.20,1.12) | 0.38 | **(0.16,0.90)*** | 0.99 | (0.48,2.04) | 0.835 | 0.086 |
|  |  | No | Ref (1.00) | 1.39 | (0.85,2.27) | 2.09 | **(1.33,3.30)**** | 2.93 | **(1.93,4.44)***** | < 0.001 | < 0.001 |
|  | Model 2 | Yes | Ref (1.00) | 0.45 | (0.18,1.16) | 0.39 | **(0.16,0.94)*** | 0.98 | (0.46,2.06) | 0.841 | 0.111 |
|  |  | No | Ref (1.00) | 1.35 | (0.81,2.23) | 2.02 | **(1.27,3.21)**** | 2.80 | **(1.81,4.33)***** | < 0.001 | < 0.001 |
|  | Model 3 | Yes | Ref (1.00) | 0.47 | (0.18,1.19) | 0.40 | **(0.16,0.99)*** | 0.99 | (0.48,2.06) | 0.818 | 0.142 |
|  |  | No | Ref (1.00) | 1.34 | (0.81,2.21) | 2.01 | **(1.28,3.16)**** | 2.79 | **(1.83,4.27)***** | < 0.001 | < 0.001 |
|  | Model 4 | Yes | Ref (1.00) | 0.53 | (0.21,1.30) | 0.46 | (0.19,1.15) | 1.11 | (0.52,2.36) | 0.658 | 0.287 |
|  |  | No | Ref (1.00) | 1.34 | (0.79,2.26) | 1.91 | **(1.20,3.05)**** | 2.47 | **(1.55,3.95)***** | < 0.001 | 0.001 |
| Drinking |  |  |  |  |  |  |  |  |  |  |  |
|  | Model l | Yes | Ref (1.00) | 0.89 | (0.58,1.36) | 1.27 | (0.82,1.96) | 1.97 | **(1.27,3.04)**** | 0.001 | 0.008 |
|  |  | No | Ref (1.00) | 1.42 | (0.63,3.22) | 1.85 | (0.87,3.93) | 2.72 | **(1.22,6.08)*** | 0.011 | 0.046 |
|  | Model 2 | Yes | Ref (1.00) | 0.87 | (0.56,1.35) | 1.24 | (0.81,1.91) | 1.84 | **(1.19,2.82)**** | 0.001 | 0.018 |
|  |  | No | Ref (1.00) | 1.49 | (0.66,3.36) | 1.85 | (0.88,3.90) | 2.82 | **(1.29,6.18)*** | 0.009 | 0.031 |
|  | Model 3 | Yes | Ref (1.00) | 0.85 | (0.55,1.33) | 1.24 | (0.81,1.90) | 1.83 | **(1.20,2.81)**** | 0.001 | 0.018 |
|  |  | No | Ref (1.00) | 1.48 | (0.66,3.34) | 1.85 | (0.88,3.88) | 2.75 | **(1.24,6.08)*** | 0.011 | 0.039 |
|  | Model 4 | Yes | Ref (1.00) | 0.90 | (0.57,1.42) | 1.27 | (0.83,1.93) | 1.81 | **(1.20,2.73)**** | 0.002 | 0.016 |
|  |  | No | Ref (1.00) | 1.45 | (0.63,3.32) | 1.68 | (0.79,3.56) | 2.35 | **(1.05,5.26)*** | 0.044 | 0.115 |
| T2DM |  |  |  |  |  |  |  |  |  |  |  |
|  | Model l | Yes | Ref (1.00) | 1.26 | (0.39,4.07) | 2.34 | (0.77,7.15) | 2.61 | (0.78,8.72) | 0.031 | 0.351 |
|  |  | No | Ref (1.00) | 0.95 | (0.63,1.45) | 1.11 | (0.75,1.65) | 1.50 | **(1.05,2.14)*** | 0.016 | 0.075 |
|  | Model 2 | Yes | Ref (1.00) | 1.17 | (0.34,4.02) | 2.26 | (0.74,6.90) | 2.39 | (0.71,8.10) | 0.049 | 0.447 |
|  |  | No | Ref (1.00) | 0.93 | (0.61,1.43) | 1.08 | (0.73,1.61) | 1.47 | **(1.03,2.10)*** | 0.022 | 0.104 |
|  | Model 3 | Yes | Ref (1.00) | 1.14 | (0.33,4.00) | 2.22 | (0.73,6.78) | 2.38 | (0.70,8.13) | 0.047 | 0.479 |
|  |  | No | Ref (1.00) | 0.93 | (0.60,1.42) | 1.07 | (0.72,1.60) | 1.45 | **(1.02,2.08)*** | 0.025 | 0.122 |
|  | Model 4 | Yes | Ref (1.00) | 1.22 | (0.35,4.26) | 2.26 | (0.75,6.85) | 2.48 | (0.74,8.27) | 0.041 | 0.414 |
|  |  | No | Ref (1.00) | 0.99 | (0.64,1.53) | 1.14 | (0.77,1.69) | 1.52 | **(1.06,2.18)*** | 0.015 | 0.072 |

^a^The variables adjusted in each model were the factors mentioned above except the stratification variables.

^b^Data were listed as the weighted odd ratio estimates and 95% confidence intervals, with *p < 0.05, **p < 0.01, ***p < 0.001.

^c^Q, quintile.

^d^Ref, reference.

^e^Tests for trends based on the variables containing the median values for each quartile.

^f^*P*_test_ was the result of Bonfreni correction.
