# Supplementary material for: The association between triglyceride-glucose index and its combination with obesity indicators and cardiovascular disease: NHANES 2003–2018
Source: Cardiovasc Diabetol. 2024 Jan 6;23:8. doi: 10.1186/s12933-023-02115-9 (PMC10771672; doi:10.1186/s12933-023-02115-9)
Supplement: Supplementary file 1 — Additional file 1. Mediation analysis of TyG, TyG-WC, TyG-WHtR, and TyG-BMI with CVD mortality, total CVD, congestive heart failure, myocardial infarction, angina pectoris, and coronary heart disease. [file 12933_2023_2115_MOESM1_ESM.pdf]

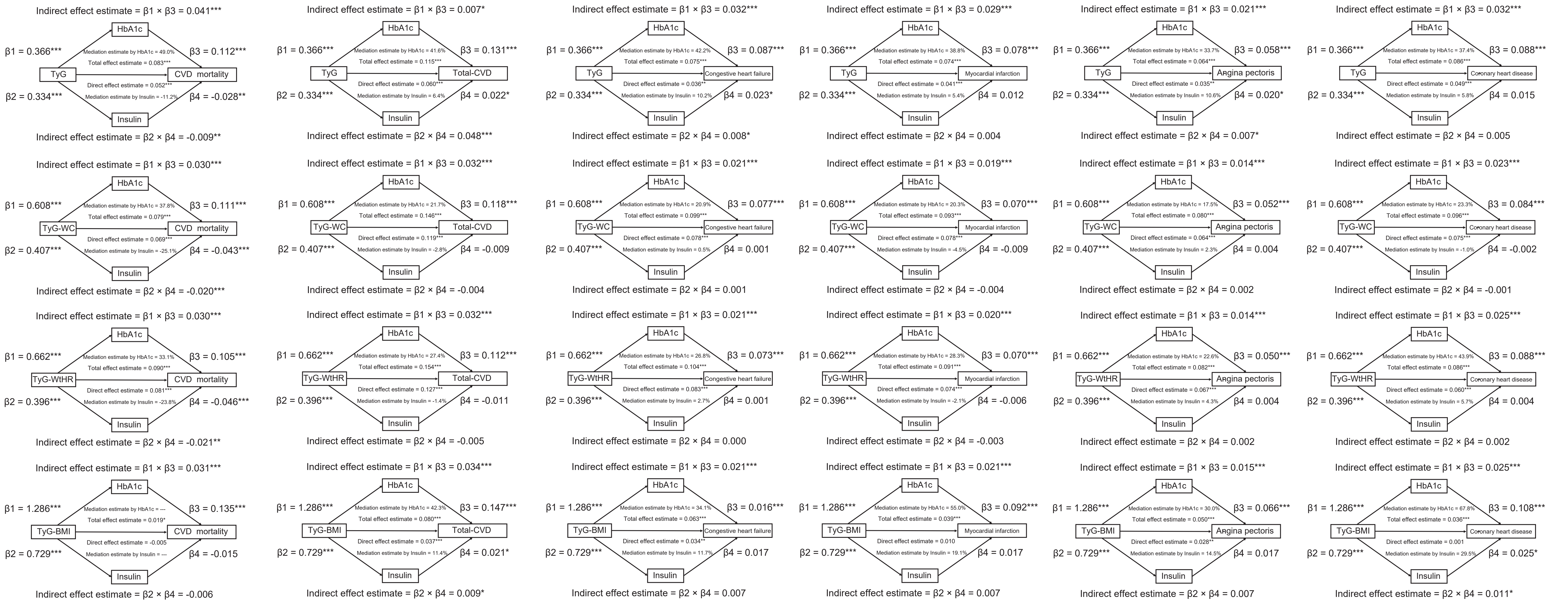
Indirect effect estimate = β1 × β3 = 0.041***
HbA1c
β1 = 0.366***
Mediation estimate by HbA1c = 49.0%
β3 = 0.112***
Total effect estimate = 0.083***
TyG
CVD mortality
Direct effect estimate = 0.052***
β2 = 0.334***
Mediation estimate by Insulin = -11.2%
β4 = -0.028**
Insulin
Indirect effect estimate = β2 × β4 = -0.009**
Indirect effect estimate = β1 × β3 = 0.007*
HbA1c
β1 = 0.366***
Mediation estimate by HbA1c = 41.6%
β3 = 0.131***
Total effect estimate = 0.115***
TyG
Total-CVD
Direct effect estimate = 0.060***
β2 = 0.334***
Mediation estimate by Insulin = 6.4%
β4 = 0.022*
Insulin
Indirect effect estimate = β2 × β4 = 0.048***
Indirect effect estimate = β1 × β3 = 0.032***
HbA1c
β1 = 0.366***
Mediation estimate by HbA1c = 42.2%
β3 = 0.087***
Total effect estimate = 0.075***
TyG
Congestive heart failure
Direct effect estimate = 0.036**
β2 = 0.334***
Mediation estimate by Insulin = 10.2%
β4 = 0.023*
Insulin
Indirect effect estimate = β2 × β4 = 0.008*
Indirect effect estimate = β1 × β3 = 0.029***
HbA1c
β1 = 0.366***
Mediation estimate by HbA1c = 38.8%
β3 = 0.078***
Total effect estimate = 0.074***
TyG
Myocardial infarction
Direct effect estimate = 0.041***
β2 = 0.334***
Mediation estimate by Insulin = 5.4%
β4 = 0.012
Insulin
Indirect effect estimate = β2 × β4 = 0.004
Indirect effect estimate = β1 × β3 = 0.021***
HbA1c
β1 = 0.366***
Mediation estimate by HbA1c = 33.7%
β3 = 0.058***
Total effect estimate = 0.064***
TyG
Angina pectoris
Direct effect estimate = 0.035**
β2 = 0.334***
Mediation estimate by Insulin = 10.6%
β4 = 0.020*
Insulin
Indirect effect estimate = β2 × β4 = 0.007*
Indirect effect estimate = β1 × β3 = 0.032***
HbA1c
β1 = 0.366***
Mediation estimate by HbA1c = 37.4%
β3 = 0.088***
Total effect estimate = 0.086***
TyG
Coronary heart disease
Direct effect estimate = 0.049***
β2 = 0.334***
Mediation estimate by Insulin = 5.8%
β4 = 0.015
Insulin
Indirect effect estimate = β2 × β4 = 0.005
Indirect effect estimate = β1 × β3 = 0.030***
HbA1c
β1 = 0.608***
Mediation estimate by HbA1c = 37.8%
β3 = 0.111***
Total effect estimate = 0.079***
TyG-WC
CVD mortality
Direct effect estimate = 0.069***
β2 = 0.407***
Mediation estimate by Insulin = -25.1%
β4 = -0.043***
Insulin
Indirect effect estimate = β2 × β4 = -0.020***
Indirect effect estimate = β1 × β3 = 0.032***
HbA1c
β1 = 0.608***
Mediation estimate by HbA1c = 21.7%
β3 = 0.118***
Total effect estimate = 0.146***
TyG-WC
Total-CVD
Direct effect estimate = 0.119***
β2 = 0.407***
Mediation estimate by Insulin = -2.8%
β4 = -0.009
Insulin
Indirect effect estimate = β2 × β4 = -0.004
Indirect effect estimate = β1 × β3 = 0.021***
HbA1c
β1 = 0.608***
Mediation estimate by HbA1c = 20.9%
β3 = 0.077***
Total effect estimate = 0.099***
TyG-WC
Congestive heart failure
Direct effect estimate = 0.078***
β2 = 0.407***
Mediation estimate by Insulin = 0.5%
β4 = 0.001
Insulin
Indirect effect estimate = β2 × β4 = 0.001
Indirect effect estimate = β1 × β3 = 0.019***
HbA1c
β1 = 0.608***
Mediation estimate by HbA1c = 20.3%
β3 = 0.070***
Total effect estimate = 0.093***
TyG-WC
Myocardial infarction
Direct effect estimate = 0.078***
β2 = 0.407***
Mediation estimate by Insulin = -4.5%
β4 = -0.009
Insulin
Indirect effect estimate = β2 × β4 = -0.004
Indirect effect estimate = β1 × β3 = 0.014***
HbA1c
β1 = 0.608***
Mediation estimate by HbA1c = 17.5%
β3 = 0.052***
Total effect estimate = 0.080***
TyG-WC
Angina pectoris
Direct effect estimate = 0.064***
β2 = 0.407***
Mediation estimate by Insulin = 2.3%
β4 = 0.004
Insulin
Indirect effect estimate = β2 × β4 = 0.002
Indirect effect estimate = β1 × β3 = 0.023***
HbA1c
β1 = 0.608***
Mediation estimate by HbA1c = 23.3%
β3 = 0.084***
Total effect estimate = 0.096***
TyG-WC
Coronary heart disease
Direct effect estimate = 0.075***
β2 = 0.407***
Mediation estimate by Insulin = -1.0%
β4 = -0.002
Insulin
Indirect effect estimate = β2 × β4 = -0.001
Indirect effect estimate = β1 × β3 = 0.030***
HbA1c
β1 = 0.662***
Mediation estimate by HbA1c = 33.1%
β3 = 0.105***
Total effect estimate = 0.090***
TyG-WtHR
CVD mortality
Direct effect estimate = 0.081***
β2 = 0.396***
Mediation estimate by Insulin = -23.8%
β4 = -0.046***
Insulin
Indirect effect estimate = β2 × β4 = -0.021**
Indirect effect estimate = β1 × β3 = 0.032***
HbA1c
β1 = 0.662***
Mediation estimate by HbA1c = 27.4%
β3 = 0.112***
Total effect estimate = 0.154***
TyG-WtHR
Total-CVD
Direct effect estimate = 0.127***
β2 = 0.396***
Mediation estimate by Insulin = -1.4%
β4 = -0.011
Insulin
Indirect effect estimate = β2 × β4 = -0.005
Indirect effect estimate = β1 × β3 = 0.021***
HbA1c
β1 = 0.662***
Mediation estimate by HbA1c = 26.8%
β3 = 0.073***
Total effect estimate = 0.104***
TyG-WtHR
Congestive heart failure
Direct effect estimate = 0.083***
β2 = 0.396***
Mediation estimate by Insulin = 2.7%
β4 = 0.001
Insulin
Indirect effect estimate = β2 × β4 = 0.000
Indirect effect estimate = β1 × β3 = 0.020***
HbA1c
β1 = 0.662***
Mediation estimate by HbA1c = 28.3%
β3 = 0.070***
Total effect estimate = 0.091***
TyG-WtHR
Myocardial infarction
Direct effect estimate = 0.074***
β2 = 0.396***
Mediation estimate by Insulin = -2.1%
β4 = -0.006
Insulin
Indirect effect estimate = β2 × β4 = -0.003
Indirect effect estimate = β1 × β3 = 0.014***
HbA1c
β1 = 0.662***
Mediation estimate by HbA1c = 22.6%
β3 = 0.050***
Total effect estimate = 0.082***
TyG-WtHR
Angina pectoris
Direct effect estimate = 0.067***
β2 = 0.396***
Mediation estimate by Insulin = 4.3%
β4 = 0.004
Insulin
Indirect effect estimate = β2 × β4 = 0.002
Indirect effect estimate = β1 × β3 = 0.025***
HbA1c
β1 = 0.662***
Mediation estimate by HbA1c = 43.9%
β3 = 0.088***
Total effect estimate = 0.086***
TyG-WtHR
Coronary heart disease
Direct effect estimate = 0.060***
β2 = 0.396***
Mediation estimate by Insulin = 5.7%
β4 = 0.004
Insulin
Indirect effect estimate = β2 × β4 = 0.002
Indirect effect estimate = β1 × β3 = 0.031***
HbA1c
β1 = 1.286***
Mediation estimate by HbA1c = ---
β3 = 0.135***
Total effect estimate = 0.019*
TyG-BMI
CVD mortality
Direct effect estimate = -0.005
β2 = 0.729***
Mediation estimate by Insulin = ---
β4 = -0.015
Insulin
Indirect effect estimate = β2 × β4 = -0.006
Indirect effect estimate = β1 × β3 = 0.034***
HbA1c
β1 = 1.286***
Mediation estimate by HbA1c = 42.3%
β3 = 0.147***
Total effect estimate = 0.080***
TyG-BMI
Total-CVD
Direct effect estimate = 0.037***
β2 = 0.729***
Mediation estimate by Insulin = 11.4%
β4 = 0.021*
Insulin
Indirect effect estimate = β2 × β4 = 0.009*
Indirect effect estimate = β1 × β3 = 0.021***
HbA1c
β1 = 1.286***
Mediation estimate by HbA1c = 34.1%
β3 = 0.016***
Total effect estimate = 0.063***
TyG-BMI
Congestive heart failure
Direct effect estimate = 0.034**
β2 = 0.729***
Mediation estimate by Insulin = 11.7%
β4 = 0.017
Insulin
Indirect effect estimate = β2 × β4 = 0.007
Indirect effect estimate = β1 × β3 = 0.021***
HbA1c
β1 = 1.286***
Mediation estimate by HbA1c = 55.0%
β3 = 0.092***
Total effect estimate = 0.039***
TyG-BMI
Myocardial infarction
Direct effect estimate = 0.010
β2 = 0.729***
Mediation estimate by Insulin = 19.1%
β4 = 0.017
Insulin
Indirect effect estimate = β2 × β4 = 0.007
Indirect effect estimate = β1 × β3 = 0.015***
HbA1c
β1 = 1.286***
Mediation estimate by HbA1c = 30.0%
β3 = 0.066***
Total effect estimate = 0.050***
TyG-BMI
Angina pectoris
Direct effect estimate = 0.028**
β2 = 0.729***
Mediation estimate by Insulin = 14.5%
β4 = 0.017
Insulin
Indirect effect estimate = β2 × β4 = 0.007
Indirect effect estimate = β1 × β3 = 0.025***
HbA1c
β1 = 1.286***
Mediation estimate by HbA1c = 67.8%
β3 = 0.108***
Total effect estimate = 0.036***
TyG-BMI
Coronary heart disease
Direct effect estimate = 0.001
β2 = 0.729***
Mediation estimate by Insulin = 29.5%
β4 = 0.025*
Insulin
Indirect effect estimate = β2 × β4 = 0.011*
